# Projections of the global, regional and national stroke burden by 2050: a systematic analysis for the Global Burden of Disease Study 2021

**DOI:** 10.1007/s10654-026-01376-4

**Published:** 2026-02-21

**Authors:** Minghong Yao, Mingqi Wang, Yu Ma, Jason W. Busse, Xin Hu, Yanmei Liu, Xiaochao Luo, Qigao Liang, Xiao Liang, Kang Zou, Ling Li, Xin Sun

**Affiliations:** 1 Department of Neurosurgery and Clinical Epidemiology and Evidence-based Medicine Center, West China Hospital, West China Hospital, Sichuan University, Chengdu, China; 2 NHC Key Laboratory of Clinical Epidemiology and Evidence-Based Medicine, West China Hospital, Sichuan University, Chengdu, China; 3 Sichuan Center of Technology Innovation for Real World Data, Sichuan University, West China Hospital, Chengdu, China; 4 Michael G DeGroote National Pain Centre, McMaster University, Hamilton, ON L8S 4K1 Canada; 5 Department of Health Research Methods, Evidence, and Impact, McMaster University, Hamilton, ON L8S 4K1 Canada; 6 Department of Anaesthesia, McMaster University, Hamilton, ON L8S 4K1 Canada; 7 Department of Neurosurgery, West China Hospital, Sichuan University, Chengdu, China; 8 Department of Encephalopathy, Xiyuan Hospital, China Academy of Chinese Medical Sciences, Beijing, China; 9 Department of Epidemiology and Biostatistics, West China School of Public Health, West China Fourth Hospital, Sichuan University, Chengdu, China; 10 Chinese Evidence-Based Medicine Center, West China Hospital, Sichuan University, 37 Guo Xue Xiang, Chengdu, Sichuan China

**Keywords:** Global burden of disease, Incidence, Prevalence, Deaths, Disability-adjusted life years, Stroke

## Abstract

This study projected the incidence, prevalence, death and disability-adjusted life-years (DALYs) attributable to total strokes and pathological types in people aged ≥ 15 years for 204 countries and territories to 2050. Age- and sex-specific trends in rates were developed using XGBoost models incorporating national human development index, gross domestic product per capita, and demographic data from the Global Burden of Disease study, the World Bank, and the United Nations. Uncertainty intervals were calculated as the 2.5th and 97.5th percentiles of the distribution using a bootstrap-like method. From 2021 to 2050, the global absolute number of incident strokes is anticipated to increase by 31.64% (27.78–35.61), prevalent strokes by 58.00% (54.68–60.62), deaths from stroke by 11.88% (5.83–19.28), and DALYs due to stroke by 17.86% (10.63–25.18). Meanwhile, the age-standardised rate of stroke prevalence will increase by 2.67% (1.47–4.23). However, the age-standardised rates of stroke incidence, death and DALYs are projected to decrease by 8.54% (6.87–10.07), 21.51% (19.18–23.92) and 18.44% (15.83–20.95), respectively. In 2050, males are projected to exceed females across all age-standardised metrics. Ischaemic stroke will account for the largest proportion of the total-stroke burden, the burden of stroke in low to middle SDI countries will account for more than half of the total global burden of stroke, and Southeast Asia, East Asia, and Oceania are projected to have the highest stroke burden in absolute numbers. Our projections suggested the global burden of stroke will increase substantially from 2021 to 2050, despite reductions in age-standardised rates.

**Supplementary Information:**

The online version contains supplementary material available at 10.1007/s10654-026-01376-4.

## Introduction

Globally, stroke was the second leading cause of death and the third leading cause of disability-adjusted life years (DALYs), according to the Global Burden of Disease, Injuries, and Risk Factors Study (GBD) 2021 report [1, 2]. There were estimated 11.9 million incident strokes, 93.8 million prevalent strokes, 7.3 million deaths from stroke, and 160.5 million DALYs lost due to stroke worldwide in 2021 [1]. More people are surviving stroke, and the age-standardized incidence rate of stroke in younger adults (< 55 years) has increased in high-income countries [3].

Mitigating the burden of stroke requires a thorough understanding of risk factors and anticipated trends in epidemiology. The estimated global lifetime risk of stroke in people aged over 25 years is 24.9% [4]. Important risk factors for stroke include high body-mass index, high fasting plasma glucose, elevated systolic blood pressure and low-density lipoprotein cholesterol level [5]. Several studies have shown that the number of people with these risk factors will increase significantly in the future [6, 7]. Furthermore, older age is a major risk factor for stroke [8], a burden that is further compounded by the progressively ageing populations in many countries [9]. Environmental risk factors, such as air pollution and rising temperatures, may also increase the burden of stroke [10]. It is, therefore, imperative that policymakers, health care providers, and researchers determine the distribution and magnitude of the expected increase in the global, regional, and national burden of stroke and its pathological types to support evidence-based resource allocation and health care planning.

Several previous studies have projected the burden of stroke at regional [9, 11, 12], national [13–16], and international levels [17–19], but these studies have focussed on stroke mortality or a specific pathological type of stroke. Further, advances in treatment suggest the likelihood of surviving a stroke will continue to increase. Stroke-related long-term sequelae and the corresponding costs are expected to increase dramatically [20]. In addition, there is evidence that the age at which stroke occurs in the population is decreasing [16]. Predicting trends in stroke incidence in the working-age population, particularly those aged 15–59 years, is essential for developing targeted prevention strategies.

The global distribution of the burden of stroke is not uniform, with significant variations between regions [5]. Taking these regional differences into account can provide insights into the future burden of stroke and guide country-specific allocation of prevention and health care services. However, information on trends in incidence, deaths, prevalence, and DALYs for stroke and its pathological type is lacking in many countries worldwide, particularly in low- and middle-income countries [21], where the impact of stroke is compounded by limited resources and inadequate health infrastructure [22]. This study therefore aimed to estimate the crude and age-standardized rates and absolute numbers for stroke incidence, prevalence, deaths, and DALYs up to 2050. Projections were made at global, regional, and national levels, stratified by pathological type, based on GBD 1990–2021 data.

## Methods

### Study population

We focussed on forecasting the incidence, prevalence, deaths and DALYs of total strokes and subtypes (ischaemic stroke, intracerebral haemorrhage, and subarachnoid haemorrhage) among individuals aged ≥ 15 years in 204 nations and territories. In addition, we examined the potential impact of stroke on working-age individuals (15–59 years). Our models used publicly available data from the GBD, the World Bank and the United Nations.

### Model development and evaluation

We developed age-, sex-, and country-specific eXtreme Gradient Boosting (XGBoost) models to predict incidence, prevalence, mortality, and DALY rates for total stroke and subtypes. The XGBoost model was selected over alternatives (e.g., ARIMA, linear regression, and Bayesian hierarchical models) due to its superior ability to model complex, non-linear relationships and to handle multicollinearity without relying on strong prior assumptions [23]. All outcome measures were modelled as rates using historical data from 1990 to 2021. As country-specific data on stroke-specific risk factors were not available, we constructed our prediction model using the following set of predictors: year, sex- and age-specific population size, national Human Development Index (HDI), and Gross Domestic Product (GDP) per capita (see Appendix 1.1). These macro-level indicators served as robust proxies for capturing stages of epidemiological transition and health system capacity across countries [9, 24, 25]. For outcomes with relatively low event rates (e.g., subarachnoid haemorrhage incidence and mortality), we incorporated a year-over-year rate difference feature to capture dynamic trends. Consequently, projections employed a recursive strategy, where predicted rates dynamically updated this lag feature for subsequent years to ensure temporal continuity.

Models were trained on data from 1990 to 2010, with a random 80% of the data allocated to the training set and the remaining 20% held out for validation. Internal model training employed 10-fold cross‑validation repeated over 10 iterations, with hyperparameter optimisation performed within each validation fold. To estimate uncertainty, the 95% uncertainty intervals (UIs) of the predicted rates were generated by propagating 500 draws through a multi‑stage computational pipeline using a bootstrap‑like approach, with the final intervals defined by the 2.5th and 97.5th percentiles of the resulting distributions. Model performance was then evaluated on the out‑of‑sample period from 2011 to 2021 using R‑squared, deviance, mean absolute error, and root mean squared error.

### Forecasting analysis

Subtype-specific rates for mortality, incidence, and DALYs were projected by country, age, and sex through 2050. The corresponding absolute numbers were then derived by multiplying these rates by the estimated size of the eligible population for each year. The absolute numbers for total stroke were obtained by aggregating the subtype-specific absolute numbers. Finally, total stroke rates were calculated by dividing these aggregated absolute numbers by the population. The total stroke prevalence rate was modelled directly from the original GBD data (Appendix 1.3). These estimates were subsequently aggregated to generate results at the global level and for the seven GBD super-regions: Central Europe, Eastern Europe and Central Asia; Latin America and Caribbean; South Asia; Sub-Saharan Africa; North Africa and the Middle East; Southeast Asia, East Asia, and Oceania; High-income countries. We also grouped all countries into 5 income levels: high, high-middle, middle, lower-middle and low according to a socio-demographic index (SDI) [26]. The age-standardised rates were calculated based on the World 2000–2025 standard population (https://seer.cancer.gov/stdpopulations/world.who.html). All projections are presented in absolute numbers (in millions) and rates per 100 000 population (with 95% UIs), stratified by age group (15–59 years and ≥ 60 years) and sex. Data processing and XGBoost modelling were performed in R version 4.3.1.

### Data sources

In this study, age-, sex-, year- and subtype-specific rates for incidence, prevalence, deaths and DALYs, as well as the prevalence rate of total stroke, were obtained from the GBD 2021 study (http://ghdx.healthdata.org/gbd-results-tool) [27]. Historical and future GDP per capita data, sourced from the Institute for Health Metrics and Evaluation, were expressed in constant 2021 US dollars adjusted for purchasing power parity [28]. Population estimates from 2000 to 2050, stratified by country, sex, and age, were sourced from the World Bank Databank (https://databank.worldbank.org/source/population-estimates-and-projections). The HDI, a comprehensive measure of human development achievements, was obtained from the United Nations Development Programme [29], while future HDI projections to 2050 were derived from an open-source tool by the Frederick S. Pardee Center for International Futures [30].

## Results

### Global trends in stroke burden by geographical region

The XGBoost models demonstrated satisfactory prediction accuracy (Fig.S1). The absolute number of incident cases is expected to increase by 31.64% (95% UI: 27.78 to 35.61) to 11.94 million (11.59 to 12.30), prevalent cases by 58.00% (54.68 to 60.62) to 144.51 million (141.47 to 146.90), deaths by 11.88% (5.83 to 19.28) to 4.99 million (4.72 to 5.32), and DALYs by 17.86% (10.63 to 25.18) to 149.62 million (140.45 to 158.91). In contrast, age-standardized rates are projected to decrease for incidence (–8.54%; -10.07 to -6.87), mortality (-21.51%; -23.92 to -19.18), and DALYs (-18.44%; -20.95 to -15.83), while the prevalence rate is expected to rise slightly (2.67%; 1.47 to 4.23) (Table 1).

**Table 1:** Absolute number, crude rates, and age-standardised rates per year of incident strokes, prevalent strokes, deaths from stroke and dalys due to stroke in 2050, and percentage change globally for 2021–2050, by pathological types of stroke

	Incidence (95% UI)		Prevalence (95% UI)		Deaths (95%UI)		DALYs (95%UI)	
	2050	Percentage change, 2021–2050	2050	Percentage change, 2021–2050	2050	Percentage change, 2021–2050	2050	Percentage change, 2021–2050
Intracerebral haemorrhage								
Absolute number	2·96 (2·85 to 3·08)	12·27 (8.00 to 16·66)	19·54 (19·25 to 19·89)	29·25 (27·33 to 31·6)	2·38 (2·26 to 2·53)	-2·28 (-7·29 to 3·87)	68·97 (64·32 to 73·82)	-0·08 (-6·81 to 6·95)
Crude rate	39·97 (38·45, 41·53)	-11·73 (-15·08 to -8·28)	263·56 (259·64 to 268·35)	1·63 (0·12 to 3·47)	32·15 (30·51 to 34·18)	-23·15 (-27·1 to -18·33))	930·46 (867·72 to 995·86)	-21·43 (-26·73 to -15·91)
Age-standardised rate	35·20 (34·39 to 36·13)	-17·06 (-18·97 to -14·87)	223·12 (219·73 to 227·36)	-5·17 (-6·61 to -3·37)	31·94 (30·82 to 33·01)	-24·15 (-26·81 to -21·61)	908·82 (880·21 to 937·30)	-24·45 (-26·82 to -22·08)
Ischaemic stroke								
Absolute number	8·22 (8·01 to 8·45)	40·5 (36·88 to 44·4)	88·60 (86·74 to 90·68)	52·56 (49·34 to 56·13)	2·23 (2·10 to 2·39)	27·49 (19·80 to 36·72)	68·20 (64·15 to 72·16)	40·41 (32·08 to 48·55)
Crude rate	110·86 (108·01 to 113·94)	10·47 (7·63 to 13·54)	1195·37 (1170·16 to 1223·4)	19·95 (17·42 to 22·77)	30·1 (28·28 to 32·27)	0·27 (-5·80 to 7·5)	920·11 (865·51 to 973·47)	10·4 (3·85 to 16·81)
Age-standardised rate	80·96 (79·77 to 82·25)	-4·70 (-6·10 to -3·18)	935·22 (921·47 to 949·10)	1·70 (0·20 to 3·21)	22·30 (21·71 to 22·89)	-19·20 (-21·34 to -17·07)	667·10 (645·91 to 689·99)	-11·98 (-14·78 to -8·96)
Subarachnoid haemorrhage								
Absolute number, millions	0·76 (0·74 to 0·78)	30·11 (27·17 to 34·31)	10·77 (10·49 to 11·11)	51·34 (47·42 to 56·21)	0·38 (0·36 to 0·39)	41·75 (35·73 to 47·88)	12·45 (11·97 to 12·94)	33·12 (27·99 to 38·3)
Crude rate	10·19 (9·96 to 10·52)	2·31 (0 to 5·62)	145·27 (141·51 to 149·95)	19·00 (15·92 to 22·83)	5·09 (4·87 to 5·31)	11·38 (6·56 to 16·19)	168·00 (161·52 to 174·53)	4·67 (0·64 to 8·74)
Age-standardised rate	9·3 (9·21 to 9·37)	-5·01 (-5·92 to -4·29)	134·85 (133·54 to 136·39)	2·37 (1·37 to 3·54)	3·71 (3·64 to 3·77)	-9·95 (-11·65 to -8·5)	150·21 (146·88 to 154·04)	-3·51 (-5·65 to -1·05)
Total stroke								
Absolute number	11·94 (11·59 to 12·30)	31·64 (27·78 to 35·61)	144·51 (141·47 to 146·90)	58·00 (54·68 to 60·62)	4·99 (4·72 to 5·32)	11·88 (5·83 to 19·28)	149·62 (140·45 to 158·91)	17·86 (10·63 to 25·18)
Crude rate	161·02 (156·42 to 165·99)	3·49 (0·53 to 6·68)	1949·52 (1908·63 to 1981·83)	24·23 (21·62 to 26·29)	67·34 (63·66 to 71·76)	-11·90 (-16·72 to -6·12)	2018·57 (1894·75 to 2143·86)	-7·33 (-13·01 to -1·57)
Age-standardised rate	125·46 (123·37 to 127·75)	-8·54 (-10·07 to -6·87)	1465·76 (1448·67 to 1488·06)	2·67 (1·47 to 4·23)	57·95 (56·17 to 59·67)	-21·51 (-23·92 to -19·18)	1726·14 (1673·00 to 1781·34)	-18·44 (-20·95 to -15·83)

Absolute numbers are in millions

The denominator of crude incidence rate and age standardized incidence rate is per 100 000 people

Absolute numbers in millions, crude rates per 100 000 people, age-standardised rates per 100 000 people, and percentage change are presented to two decimal places

*UI* uncertainty interval, *DALY* disability adjusted life-year

Regionally, Southeast Asia, East Asia, and Oceania are projected to bear the highest stroke burden in absolute number of cases in 2050, with 3.94 million (3.70 to 4.19) new stroke cases, 50.05 million (47.66 to 51.87) stroke survivors, 1.66 million (1.49 to 1.88) deaths from stroke, and 50.87 million (45.21 to 57.02) DALYs lost due to stroke. Southeast Asia, East Asia and Oceania are also projected to have the highest age-standardised rates for incidence (170.31 per 100 000), deaths (105.13 per 100 000) and DALYs (2923.34 per 100 000), as well as the highest crude death rate (91.86 per 100 000) (Table S1). In comparison, Central Europe, Eastern Europe, and Central Asia are projected to have the highest crude incidence (225.74 per 100 000) and DALYs rates (3175.60 per 100 000). Sub-Saharan Africa is projected to have the highest age-standardized prevalence rate (866.36 per 100 000) (Table S1).

**Fig. 1:**
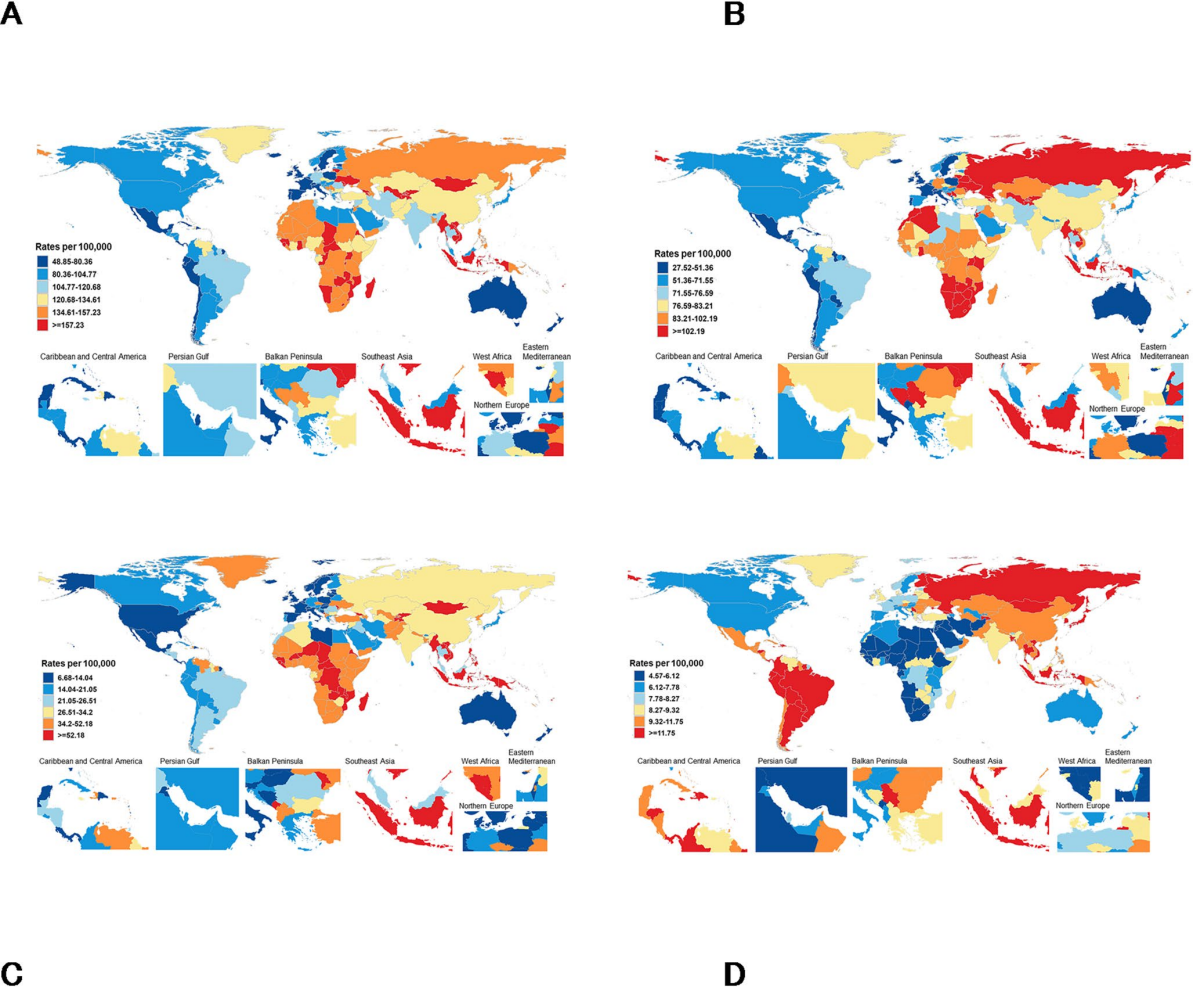
Age-standardised incidence rates per 100 000 people by stroke type and country, for both sexes, 2050. **A**: total stroke, **B**: Ischaemic stroke, **C**: Intracerebral haemorrhage, **D**: Subarachnoid haemorrhage

Substantial differences among countries were observed in age-standardised stroke incidence rate, with the lowest in Malta (46.52 [30.91 to 58.75] per 100 000) and the highest in Georgia (333.57 [294.49 to 370.76] per 100 000) (Fig. 1A). We also observed large geographical disparities in age-standardised stroke prevalence rates, with the lowest in Cyprus (500.44 [338.50 to 646.65] per 100 000) and the highest in Georgia (5051.13 [4662.98–5555.14] per 100 000); death rates, with the lowest in Malta (9.82 [8.81 to 10.90] per 100 000) and the highest in Kiribati (280.54 [239.70 to 391.91] per 100 000); and DALYs rates, with the lowest in Malta (220.91 [110.67 to 412.57] per 100 000) and the highest in Kiribati (7819.83 [6774.77 to 8712.42] per 100 000) (Table S2; Fig.S2A & S3A & S4A).

### Trends in stroke burden by pathological types

By 2050, ischaemic stroke is projected to account for the largest proportion of total strokes in terms of incidence (8.22 million [95% UI 8.01 to 8.45]; 68.84% of total strokes), prevalence (88.60 million [86.74 to 90.68]; 61.31% of total strokes), and deaths (2.23 million [2.10 to 2.39]; 44.69% of total strokes). For the three pathological types of strokes, age-standardised rates of incidence, prevalence, deaths and DALYs are projected to decrease from 2021 to 2050 (except for ischaemic stroke, where age-standardised prevalence rate is projected to increase by 1.70% [0.20 to 3.21]; and for subarachnoid haemorrhage, where age-standardised prevalence rate is projected to increase by 2.37% [1.37 to 3.54]) (Table 1).

**Fig. 2:**
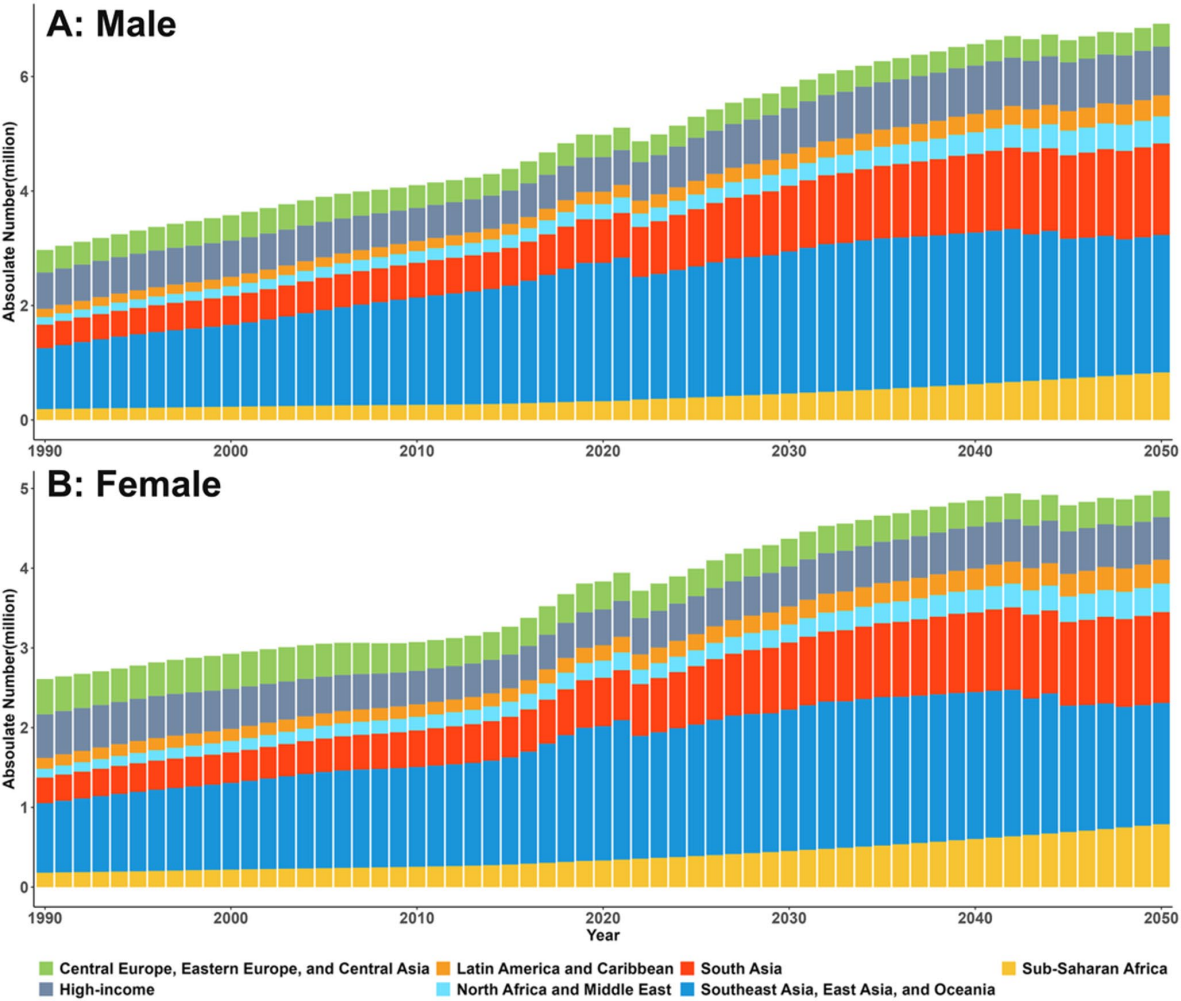
Global time trend in the number of stroke incident for all ages in seven GBD super-regions, by sex, 2020–2050

Southeast Asia, East Asia, and Oceania are projected to have the highest age-standardised rates for incidence, prevalence, deaths, and DALYs across all pathological types. However, for ischaemic stroke, Central Europe, Eastern Europe and Central Asia are projected to have the highest age-standardised rates for incidence (106.08 per 100 000), prevalence (1105.54 per 100 000) and DALYs rate (892.54 per 100 000); Sub-Saharan Africa is projected to have the highest age-standardised death rate (34.98 per 100 000) (Table S1 & Fig. 2B-D & Fig.S2B-D & S3B-D & S4B-D).

### Trends in stroke burden by sex

In 2050, males are projected to bear a higher stroke burden than females across all metrics. Specifically, males are projected to account for 58.20% of incident cases (6.95 million [6.71 to 7.20]), 53.07% of prevalent cases (76.65 million [75.31 to 78.11]), 58.31% of deaths (2.91 million [2.71 to 3.14]), 60.80% of DALYs lost (90.72 million [85.62 to 95.97]). Global age-standardised rates for all metrics are projected to be higher in males (Table S3).

Analysis by pathological type showed a male predominance in ischemic stroke and intracerebral haemorrhage. Conversely, subarachnoid haemorrhage is projected to exhibit a reverse pattern, with lower incidence, prevalence, and deaths in males compared to females, although males retain higher DALYs (Table S3). Ischemic stroke is projected to constitute the largest proportion of incident and prevalent cases globally and across super-regions for both sexes (Fig.S5). Southeast Asia, East Asia, and Oceania are projected to have the highest proportion of incident strokes for both males and females in 2050 (Fig. 2).

### Trends in stroke burden by age

Globally, among individuals aged 15–59 years, the absolute number is projected to decrease for total stroke incidents (-6.23% [-10.48 to -1.98]), deaths (-13.46% [-18.27 to -7.69]) and DALYs (-20.20% [-29.66 to -10.43]). Conversely, prevalent cases are projected to increase by 10.46% (7.31 to 14.70), resulting in an additional 3.89 million prevalent cases in 2050. Both crude and age-standardised rates of total strokes are projected to decline from 2021 to 2050. Specifically, crude rates will decrease by 16.83% (12.89 to 20.64) for incidence, 1.99% (-1.78 to 4.77) for prevalence, 23.03% (17.92 to 27.88) for deaths, and 29.19% (20.53 to 37.58) for DALYs. Age-standardised total stroke rates are projected to decrease by 12.23% (8.22 to 15.27) for incidence, 0.86% (-2.01 to 3.38) for prevalence, 15.26% (11.66 to 18.56) for deaths, and 18.52% (13.11 to 23.49) for DALYs (Table S4).

Among individuals aged ≥ 60 years, the absolute total stroke burden is projected to rise substantially, with incidence increasing by 55.23% (50.18 to 61.37), prevalence by 89.75% (86.88 to 93.16), deaths by 19.65% (12.02 to 28.74), and DALYs by 40.68% (32.48 to 49.44). The total stroke crude rates are projected to decrease by 18.95% (15.81 to 21.61) for incidence, 0.96% (-0.81 to 2.47) for prevalence, 37.65% (32.85 to 41.67) for death, and 26.57% (22.00 to 30.86) for DALYs. Age-standardised rates are projected to decrease for incidence (-5.95% [-7.17 to -4.65]), deaths (-23.44% [-26.04 to -20.85]), and DALYs (-18.42% [-20.32 to -16.55], but increase for prevalence (6.01% [5.04 to 6.85]) (Table S4).

**Fig. 3:**
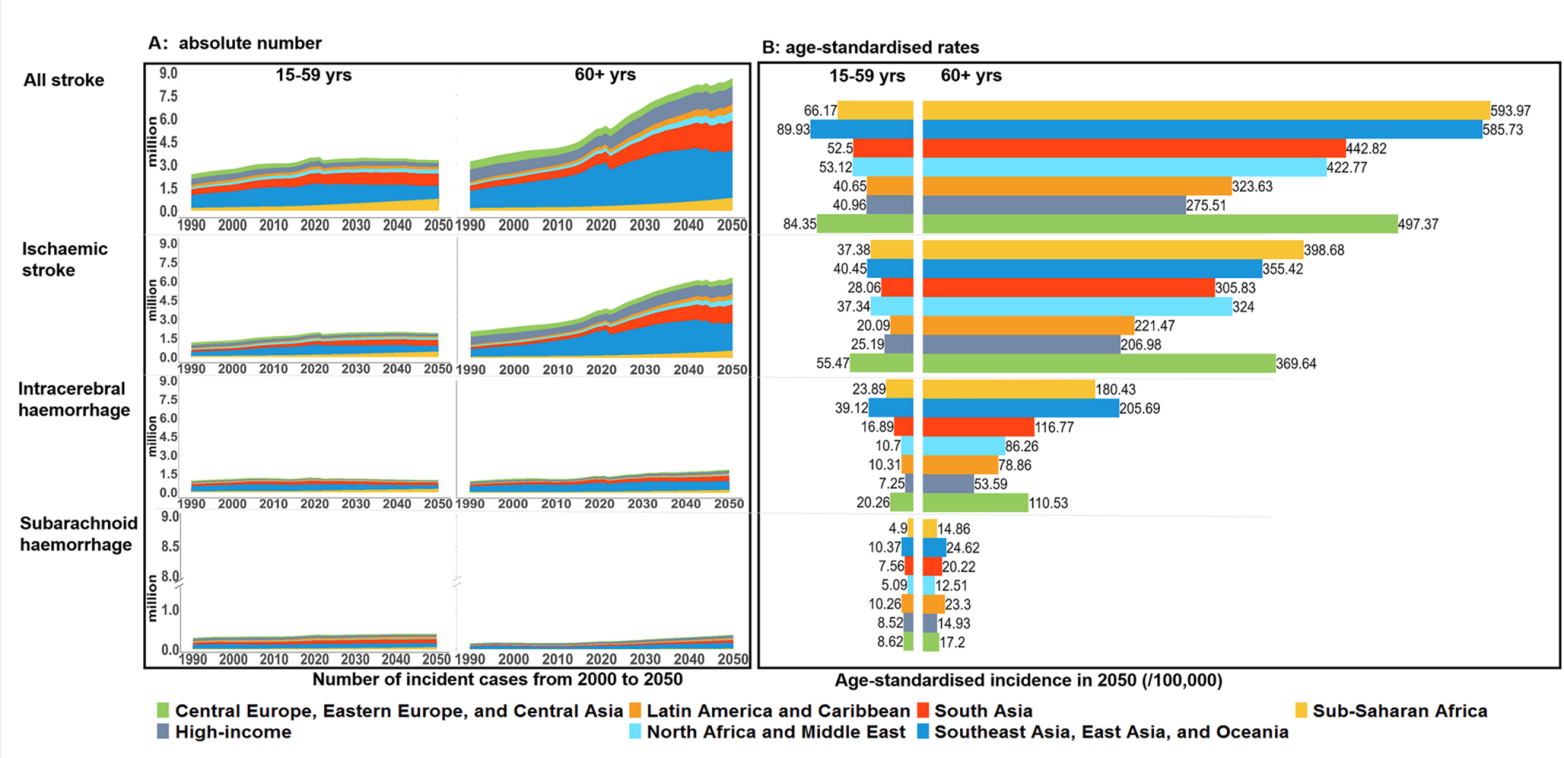
Predicted incident cases and age-standardised incidence rates for total strokes by age group in seven GBD super-regions. **A**: Temporal trends in incident cases by age group (15–59 and 60+) in seven GBD super-regions from 2000 to 2050; **B**: Age-standardised incidence rates in the seven GBD super-regions in 2050

Regionally, for individuals aged 15–59 years, Southeast Asia, East Asia, and Oceania are projected to have the highest age-standardised rates of total stroke incidence (89.93 per 100 000, 0.84 million cases), prevalence (995.36 per 100 000, 11.00 million cases), deaths (36.11 per 100 000, 0.25 million deaths), and DALYs (1492.61 per 100 000, 11.25 million years) in 2050. For those aged ≥ 60 years, Sub-Saharan Africa is projected to have the highest age-standardised rates of total stroke incidence (593.97 per 100 000, 0.83 million cases), prevalence (5828.24 per 100 000, 8.31 million cases), deaths (461.73 per 100 000, 0.59 million deaths), and DALYs (10366.48 per 100 000, 13.75 million years) in 2050, but Southeast Asia, East Asia, and Oceania are projected to have the highest absolute number of new stroke events (3.08 million), stroke survivors (38.72 million), deaths (1.41 million), and DALYs lost (39.35 million) (Fig. 3, Fig.S6 & S7 & S8).

### Projected trends by income

From 2021 to 2050, the absolute number of incidents, prevalent cases, deaths and DALYs from total stroke and its pathological types are all projected to increase in countries with a low- to middle-SDI. As a result, in 2050, 60.91% of incidents, 50.78% of prevalent cases, 70.45% of deaths, and 67.81% of DALYs from total stroke are projected to occur in low to middle SDI countries (Table 2; Table S5 & S6 & S7). These trends are similar across individuals aged 15–59, and those aged ≥ 60 years (Tables S5, S8 to S15).

**Table 2:** Number of cases, crude rates and age-standardised rates per 100 000 population in 2050 and percentage change between 2021 and 2050 for stroke incidence, by SDI quintile, for both sexes (95% uncertainty interval)

		Intracerebral haemorrhage		Ischaemic stroke		Subarachnoid haemorrhage		All stroke	
		2050	Percentage change, 2021–2050	2050	Percentage change, 2021–2050	2050	Percentage change, 2021–2050	2050	Percentage change, 2021–2050
Low SDI	Absolute number	0·40 (0·38 to 0·42)	121·68 (111·93 to 130·39)	0·69 (0·67 to 0·71)	156·82 (148·67 to 166·38)	0·06 (0·06 to 0·06) ^#1^	108·79 (105·22 to 111·95)	1·15 (1·11 to 1·19)	140·57 (132·09 to 149·41)
	Crude rate	41·18 (39·37 to 42·8)	2·75 (-1·77 to 6·79)	70·54 (68·31 to 73·17)	19·04 (15·26 to 23·47)	6·29 (6·19 to 6·39)	-3·22 (-4·88 to -1·76)	118·02 (113·86 to 122·35)	11·5 (7·58 to 15·6)
	Age-standardized rate	55·80 (54·19 to 57·21)	-11·94 (-14·48 to -9·71)	89·52 (87·28 to 91·71)	3·24 (0·65 to 5·77)	7·78 (7·67 to 7·92)	-1·99 (-3·5 to -0·32)	153·1 (149·13 to 156·84)	-3·11 (-5·62 to -0·74)
Low-middle SDI	Absolute number	1·00 (0·94 to 1·06)	24·4 (17·24 to 31·71)	2·57 (2·42 to 2·71)	143·92 (129·36 to 156·98)	0·23 (0·22 to 0·23) ^#2^	42·68 (39·25 to 45·88)	3·80 (3·59 to 4·00)	88·3 (77·56 to 98·28)
	Crude rate	38·17 (35·97 to 40·41)	-13·77 (-18·74 to -8·71)	98·25 (92·38 to 103·51)	69·08 (58·98 to 78·12)	8·76 (8·55 to 8·96)	-1·10 (-3·48 to 1·12)	145·18 (136·91 to 152·88)	30·52 (23·08 to 37·44)
	Age-standardized rate	51·53 (50·03 to 52·99)	-19·61 (-21·95 to -17·33)	95·39 (93·49 to 97·36)	1·59 (-0·43 to 3·68)	9·67 (9·55 to 9·75)	-12·84 (-13·89 to -12·06)	156·59 (153·07 to 160·1)	-7·39 (-9·47 to -5·32)
Middle SDI	Absolute number	0·59 (0·57 to 0·62)	25·82 (20·27 to 31·51)	1·55 (1·51 to 1·60)	69·26 (64·88 to 75·11)	0·17 (0·16 to 0·17) ^#3^	38·4 (35·72 to 42·73)	2·31 (2·24 to 2·40)	53·2 (48·59 to 58·88)
	Crude rate	44·12 (42·17 to 46·11)	-2·09 (-6·41 to 2·34)	115·03 (112·06 to 119·00)	31·72 (28·31 to 36·26)	12·27 (12·04 to 12·66)	7·7 (5·62 to 11·06)	171·42 (166·26 to 177·77)	19·22 (15·63 to 23·64)
	Age-standardized rate	30·25 (28·82 to 31·71)	-18·49 (-22·34 to -14·55)	73·74 (71·43 to 76·41)	-4·32 (-7·32 to -0·86)	10·06 (9·94 to 10·17)	5·68 (4·4 to 6·83)	114·05 (110·18 to 118·29)	-7·80 (-10·93 to -4·37)
High-middle SDI	Absolute number	0·75 (0·68 to 0·83)	-24·44 (-31·84 to -16·71)	2·41 (2·28 to 2·58)	-14·17 (-18·92 to -8·28)	0·20 (0·19 to 0·22)	21·04 (12·91 to 31·3)	3·37 (3·15 to 3·63)	-15·25 (-20·8 to -8·71)
	Crude rate	47·24 (42·61 to 52·07)	-23·67 (-31·14 to -15·86)	151·23 (142·87 to 161·61)	-13·29 (-18·09 to -7·34)	12·81 (11·95 to 13·90)	22·28 (14·06 to 32·64)	211·28 (197·43 to 227·58)	-14·39 (-19·99 to -7·78)
	Age-standardized rate	25·92 (24·45 to 27·54)	-26·26 (-30·45 to -21·66)	82·41 (79·43 to 85·09)	-14·42 (-17·52 to -11·64)	9·96 (9·75 to 10·17)	-6·69 (-8·66 to -4·73)	118·29 (113·62 to 122·8)	-16·77 (-20·05 to -13·6)
High SDI	Absolute number	0·21 (0·21 to 0·23)	24·39 (18·94 to 30·87)	0·98 (0·92 to 1·07)	29·41 (20·48 to 40·49)	0·10 (0·09 to 0·10) ^#4^	-1·49 (-3·24 to 0·58)	1·29 (1·22 to 1·39)	25·63 (17·97 to 35·08)
	Crude rate	24·42 (23·35 to 25·69)	18·82 (13·62 to 25·01)	111·87 (104·15 to 121·45)	23·62 (15·09 to 34·2)	10·99 (10·8 to 11·23)	-5·90 (-7·58 to -3·92)	147·29 (138·3 to 158·37)	20·00 (12·68 to 29·03)
	Age-standardized rate	15·91 (15·21 to 16·77)	-14·59 (-18·36 to -9·96)	67·46 (64·98 to 70·3)	-10·74 (-14·02 to -6·98)	9·47 (9·26 to 9·62)	-7·26 (-9·35 to -5·74)	92·84 (89·45 to 96·7)	-11·09 (-14·34 to -7·39)

Absolute numbers are in millions

The denominator of crude incidence rate and age standardized incidence rate is per 100 000 people

Absolute numbers in millions, crude rates per 100 000 people, age-standardised rates per 100 000 people, and percentage change are presented to two decimal places

*UI* uncertainty interval, *DALY* disability adjusted life-year

^#1^: Absolute number of subarachnoid haemorrhage incident cases in low SDI countries in 2050 is 61,296 (60247, 62224)

^#2^: Absolute number of subarachnoid haemorrhage incident cases in low-middle SDI countries in 2050 is 229,442(223922, 234578)

^#3^: Absolute number of subarachnoid haemorrhage incident cases in middle SDI countries in 2050 is 165,509 (162313, 170687)

^#4^: Absolute number of subarachnoid haemorrhage incident cases in high SDI countries in 2050 is 96,614 (94896, 98649)

## Discussion

The absolute numbers of incidents, prevalent cases, stroke deaths, and DALYs due to total stroke are projected to increase substantially by 2050; however, these trends differ by age group and country SDI. Working-age individuals (15–59 years) show decreased absolute numbers of incidence, deaths and DALYs, and increased prevalence, whereas older individuals (aged ≥ 60 years) show increased incidence, prevalence, deaths and DALYs. Ischaemic stroke will account for the largest proportion of the total stroke burden. In 2050, the highest absolute numbers and age-standardised rate of incidents due to total strokes are anticipated to occur in Southeast Asia, East Asia, and Oceania. Males are projected to have higher age-standardised rates of total stroke incidence, prevalence, deaths, and DALYs than females. In the 15–59 age group, the highest absolute numbers and age-standardised rates of total stroke incidence, prevalence, deaths, and DALYs are expected in Southeast Asia, East Asia, and Oceania. For those aged 60 and over, Sub-Saharan Africa is projected to have the highest age-standardised rates of total stroke incidence, prevalence, deaths, and DALYs, whereas Southeast Asia, East Asia, and Oceania are projected to have the highest absolute numbers across all measures. The burden of total stroke in low to middle SDI countries will account for more than half of the total global burden of stroke.

The concurrent increase in both the absolute number of the total stroke incidents and the crude incidence rate globally is primarily driven by demographic shifts. Our projections indicated a shift in the age distribution of new total stroke cases. While incidents among individuals aged 15–59 years are projected to decrease from 3.53 to 3.31 million, cases among those aged ≥ 60 years are expected to rise from 5.54 to 8.60 million by 2050. This trend is driven by contrasting demographic shifts, the elderly population (≥ 60 years) is projected to nearly double (from 1.07 to 2.05 billion), compared to a more modest increase in the younger population (from 4.76 to 5.37 billion). This divergent trend is clearly exemplified in China, which bears the world’s heaviest total stroke burden. A previous study projected that, from 2019 to 2050, new total strokes among the elderly (≥ 65 years) would more than double, from 2.34 to 4.79 million, while cases among those under 65 would decline from 1.60 to 1.34 million. This pattern aligns directly with the projected increase in China’s elderly population proportion from 18.06% to 32.69% [16]. On the other hand, the increasing number of new total stroke incidents may be because of increased population exposure to major risk factors, high body-mass index, hypertension, diabetes, tobacco use, harmful alcohol consumption, and environmental hazards [4, 31–34]. In contrast, the observed declines in age-standardised incidence, death, and DALY rates signal a reduction in individual-level risk, likely attributable to successful prevention strategies and health-system improvements. Key contributing factors include global tobacco control policies, the expansion of hypertension management, and the establishment of acute stroke care units [35]. Furthermore, the increase in stroke age-standardised prevalence rates and its absolute numbers may be due both to an increase in new cases and to improvements in medical care that result in higher survival rates among stroke patients. The increase in the absolute number of DALYs due to stroke highlights the need for greater attention to the socio-economic impact of stroke, particularly disability, in the working age population. Our projections show a higher burden of stroke in men than women, which is consistent with the World Stroke Organisation’s global stroke facts [36]. Men tend to have a higher prevalence of major risk factors such as high blood pressure, diabetes, atrial fibrillation and atrial flutter [36, 37].

Geographically, our projections indicate that the burden of stroke in 2050 will fall disproportionately on low- to middle‑SDI countries, particularly those in Southeast Asia, East Asia, Oceania, and sub‑Saharan Africa. Constraints in healthcare delivery and greater exposure to environmental risk factors in these regions are likely to adversely affect stroke prevention and treatment [4]. Previous reviews have highlighted that in Southeast Asia, factors such as inadequate healthcare, inequalities in standards of care, lifestyle and dietary habits, and socioeconomic disparities among large marginalized populations contribute to an elevated stroke burden [38]. Consistent with this, other GBD studies have identified air pollution and low fruit intake as leading attributable risks for stroke in low‑income settings [4]. In contrast, high‑middle‑ and high‑SDI countries possess greater capacity to invest in stroke prevention, treatment, and public health strategies, which can mitigate future burden [39–42]. The disproportionate stroke burden projected for low‑ and middle‑SDI regions thus underscores a critical gap in health‑system preparedness [43]. In these settings, primary care infrastructure is often fragmented, limiting widespread screening for key risk factors such as hypertension and atrial fibrillation. There is also a severe shortage of specialised clinical workforce (e.g., neurologists, rehabilitation therapists), leading to heavy reliance on non‑physician health workers. Furthermore, the “double burden” of persistent infectious diseases and rising non‑communicable diseases stretches already limited financial and human resources, creating substantial barriers to implementing comprehensive stroke care plans [44]. Compounding these systemic challenges is the inconsistent availability and affordability of essential preventive medications, such as statins and antihypertensives [44].

Factors associated with increased stroke burden in general include early development of vascular risk factors (e.g., high blood pressure, diabetes), unhealthy lifestyle, and a lack of stroke awareness [31, 45]. High blood pressure contributes to approximately half of total strokes; however, a 2024 cross-sectional study of 3129 US adults with uncontrolled hypertension found more than half were unaware they had high blood pressure and were untreated, including 93% of those aged 18 to 44 [46]. Every millimetre reduction in systolic blood pressure is associated with a 15% to 20% reduction in stroke incidence [47]. These trends highlight the need for greater attention to early detection, lifestyle changes and culturally sensitive stroke care [38]. Such policies, particularly in developing countries, are critical to reducing health inequities, raising awareness [41, 48, 49], and fostering a collective commitment to reducing stroke-related morbidity and mortality. Our findings indicate rising stroke prevalence in both crude and age-standardized rates, as well as in absolute case numbers. This growing burden signals a profound public health crisis, one that extends beyond individual health to strain healthcare systems and economies worldwide. Effective responses must be context-specific, addressing the distinct epidemiological and resource realities in different settings. In countries with an upper-middle and high-SDI, the primary challenge is managing a high-prevalence population of survivors within aging societies. Telemedicine networks, stroke service monitoring, and structures that link hospital care with community services show promise for improving the quality of life of stroke survivors [50]. In low- and middle-SDI regions, the primary challenge is to curb the rising incidence of disease with limited resources and overburdened health systems. High-impact population-wide interventions are paramount. Governments should prioritize legislative actions to control tobacco use and reduce salt intake [51], as these measures offer substantial preventive benefits at low cost. Furthermore, to address the shortage of specialists, health systems should adopt task-shifting strategies, such as training non-physician health workers to manage uncomplicated hypertension, and promote the use of polypills to improve medication adherence and affordability [52].

There have been several previous studies describing the global epidemiology of stroke and some stroke projection studies specific to China and Europe [9, 11, 16]. Only one study projected the global burden of ischaemic stroke from 2020 to 2030 [19]. Another study projected the trends associated with the burden of stroke in China from 2019 to 2050 [16]. A study conducted in Europe found that mortality would be halved between 2005 and 2030 [9, 11]. Another study projected stroke incidence, prevalence, deaths and DALYs in Europe [9]. Our estimates are consistent with these results and extend projections for the burden of total stroke and its pathological types for all countries in the world. Our findings suggest that more attention should be paid to changes in the burden of stroke in countries with a low- to middle- SDI, and particularly to the increasing burden among working-age populations in these countries.

This study has several limitations. First, our models did not incorporate stroke specific risk factors (e.g., smoking, hypertension, ) and other risk factors (e.g., air pollution, climate change) due to the lack of age-stratified exposure data at the national level, which may affect the accuracy of long-term projections. Second, projections are based on forecasted future values for population, HDI, and GDP. Since these input projections may themselves deviate from actual future values, this uncertainty consequently affects the accuracy of our final estimates. Third, our projections for 2050 depend on the assumption that the historical relationships between the predictors and the outcomes will remain stable over time. While this study projects the stroke-related disease burden over the next 30 years, it is important to note that, due to data availability constraints, the model’s predictive performance was externally validated only using data from 2011 to 2021. Consequently, the projections, particularly long-term ones, may deviate from future observed outcomes. Fourth, population and HDI data were unavailable for eight small-population countries (e.g., Tokelau, Niue, South Sudan) in Africa and Oceania, potentially reducing the precision of estimates for these settings. Finally, as with all trend-based projections, our model does not account for the impact of potential major public health events that may occur in the future on the prediction results; for instance, given the link between COVID-19 and stroke risk [53, 54], our estimates may underestimate the future burden.

In conclusion, our projections show that the global burden of stroke will increase substantially in the coming decades as the population ages, with a particularly pronounced impact in countries with a low to middle SDI. Targeted interventions and policies designed to alleviate the burden of stroke are urgently needed, particularly in people aged 15–59 years and in countries with a low- and low-middle SDI. Our findings underscore the need to adopt tailored public health policies and interventions that address the specific challenges faced by at-risk populations which vary by geographical location. Such tailored approaches are essential to effectively manage and reduce the growing burden of stroke in the coming decades.

## Supplementary Information

Below is the link to the electronic supplementary material.

Supplementary Material 1

## Data Availability

No additional data available.
