# Supplementary material for: Projections of the global, regional and national stroke burden by 2050: a systematic analysis for the Global Burden of Disease Study 2021
Source: Eur J Epidemiol. 2026 Feb 21;41(3):337–49. doi: 10.1007/s10654-026-01376-4 (PMC13222217; doi:10.1007/s10654-026-01376-4)
Supplement: Supplementary file 1 — Supplementary Material 1 [file 10654_2026_1376_MOESM1_ESM.docx]

**Supplement to: Projections of the global, regional and national stroke burden by 2050: a systematic analysis for the Global Burden of Disease Study 2021**

**Tables of Contents**

[Appendix 1. Model for XGBoost 3](#_Toc220060854)

[Appendix 2. Burden of all strokes and their pathological types in different super-regions in 2050 6](#_Toc220060855)

[Appendix 3. Projected age-standardised rates for prevalence, deaths, and DALYs by stroke type and country in 2050 16](#_Toc220060856)

[Appendix 4. Burden of all strokes and their pathological types by sex in 2050 61](#_Toc220060857)

[Appendix 5. Burden of all strokes and their pathological types by age in different super-regions in 2050 65](#_Toc220060858)

[Appendix 6. Burden of all strokes and their pathological types by socio-demographic index level and ages in 2050 70](#_Toc220060859)

# Appendix 1. Model for XGBoost

**1.1 Model construction**

We modelled different XGBoost models for incidence, prevalence, deaths and DALY rates. Sex- and age-specific population size was included as a proxy variable of the base to measure comprehensive national strength. HDI was included as a measure of social development level. And GDP per capita was included as the economy level, which may reflect the medical cost. In addition, the model also includes the incidence, prevalence, deaths and DALYs rates of the previous year into the modelling, so when making predictions, the model also makes predictions year by year to ensure the continuity of prediction results in time.

The outcome was a transformed population-based rate. Specifically, for each location 𝑖 and year 𝑡, the event rate was defined as the ratio of the observed number of events 𝑉_𝑖, 𝑡_ to the corresponding total population 𝑁_𝑖, 𝑡_, and a square root transformation was applied to stabilize variance:

$$\text{y}_{i,t}=\sqrt{\frac{V_{i,t}}{N_{i,t}}}$$

The covariates included location, total population size, Human Development Index (HDI), gross domestic product per capita adjusted for purchasing power parity (GDP-PPP), age group (15-59 years old, 60+ years old), calendar year, sex, World Bank income group, and geographic region. To account for temporal dependency and historical trends, the lagged value of the transformed outcome from the previous time point was additionally included as a predictor. Formally, the model can be expressed as an additive ensemble of regression trees:

$\hat{\text{y}}_{i,t}=\int(X_{i,t})=\sum_{k=1}^{K} g_{k}(X_{i,t})$,

where 𝑋_𝑖, 𝑡​_ denotes the vector of covariates for location 𝑖i at year𝑡t, 𝑔_𝑘_ (⋅) represents the 𝑘-th regression tree, each 𝑔_𝑘_ (⋅) corresponds to an independent tree structure and leaf weights, and 𝐾 is the total number of trees in the ensemble. Model parameters were estimated by minimizing a regularized objective function that combines a loss term with penalties on model complexity, as implemented in the XGBoost framework:

.

Here the second term Ω penalizes the complexity of the model according to the number T of leaves in the k-th tree and leaf weights *w*. This approach allows flexible modelling of nonlinear effects and high-order interactions among predictors without prespecifying functional forms.

**1.2 Model performance**

Root Mean Squared Error (RMSE), R-squared (R^2^) and Mean Absolute Error (MAE) were used to select the optimal model using the smallest value. We evaluated the performance of the final trained models in test sets. The final XGBoost models demonstrated robust predictive performance across all indicators and stroke subtypes in the test sets, with R^2^ ranging from 0·933 to 0·997 for all models. RMSE and MAE remain consistently low across all models. Results of evaluation was illustrated in Fig.S1.


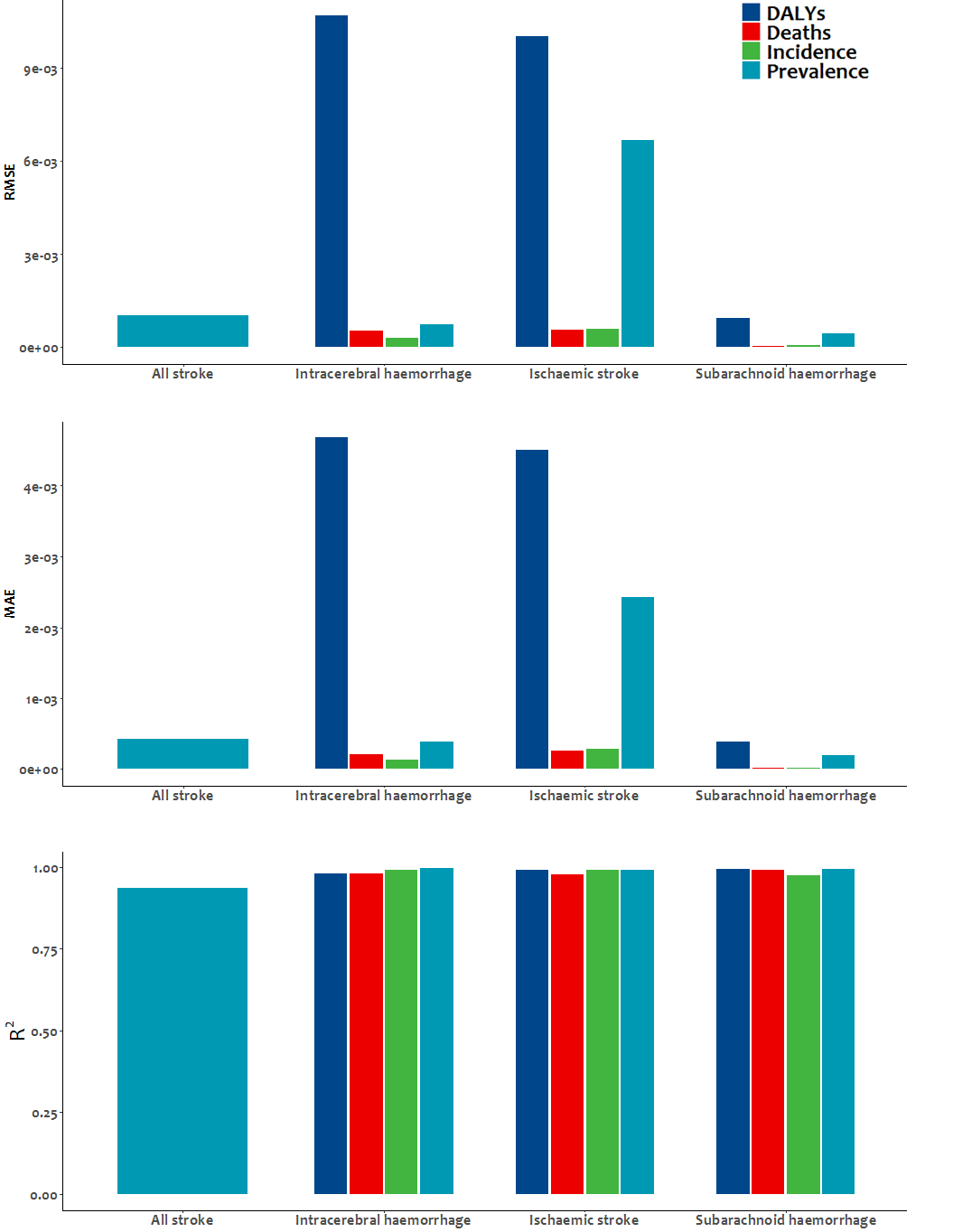


**Fig.S1 Performance of all XGBoost models**

**1.3 Estimation of overall stroke rates across subtypes**

Stroke outcomes were first modelled separately for three major stroke subtypes. For each sex *g*, age *a*, location 𝑖 and year 𝑡, subtype-specific rates for mortality, incidence, and DALY rate, denoted as $r_{g,a,i,t,s}^{(k)}$, were predicted using XGBoost models, where 𝑠 indexes stroke subtype and 𝑘 denotes the outcome type. Subtype-specific predicted rates were converted into predicted event counts by multiplying by the corresponding population size 𝑁*_g, a_,* _𝑖, 𝑡_. Overall stroke rates for mortality, incidence, and DALYs were then obtained by summing predicted event counts across subtypes and dividing by the total population:

$$\hat{R}_{i,t}^{(k)}=\frac{\sum_{s=1}^{3} r_{g,a,i,t,s}^{(k)}*N_{g,a,i,t}}{N_{i,t}}$$

This aggregation is appropriate for event-based outcomes such as deaths, incident cases, and DALYs.

In contrast, overall stroke prevalence cannot be derived by summing subtype-specific prevalence rates, as individual patients may experience more than one stroke subtype, violating the assumption of mutual exclusivity. Accordingly, overall stroke prevalence was modelled separately using an independent XGBoost model, with the prevalence rate of total stroke serving as the target variable. The predicted prevalence rate was defined as:

$$\hat{p}rev_{i,t}=f_{prev}(X_{i,t})$$

where 𝑓_prev_ (⋅) denotes the prevalence-specific model and 𝑋_𝑖, 𝑡_ represents the set of covariates.

# Appendix 2. Burden of all strokes and their pathological types in different super-regions in 2050

**Table S1. Absolute number, crude rates, and age-standardised rates per 100 000 people per year of incident and prevalent strokes, deaths from stroke and DALYs due to stroke in 2050 and percentage change in seven GBD super-regions for 2021–2050, by pathological type of stroke.**

|  | **2021** | | | **2050** | | | **Relative change** | | |
| --- | --- | --- | --- | --- | --- | --- | --- | --- | --- |
|  | **Number** | **Crude Rate** | **Age-standardised Rate** | **Number** | **Crude Rate** | **Age-standardised Rate** | **Number** | **Crude Rate** | **Age-standardised Rate** |
| **Southeast Asia, East Asia, and Oceania** | | | | | | | | | |
| **Incidence** | | | | | | | | | |
| Intracerebral haemorrhage | 1.26 | 73.88 | 73.58 | 1.04  (0.96, 1.13) | 57.62  (53.17, 62.32) | 66.00  (63.42, 69.09) | -16.86  (-23.29, -10.08) | -21.33  (-27.4, -14.91) | -10.67  (-14.16, -6.48) |
| Ischaemic stroke | 2.81 | 90.97 | 90.21 | 2.65  (2.51, 2.8) | 146.47  (138.35, 154.49) | 91.62  (87.95, 95.1) | -5.48  (-10.72, -0.3) | -10.55  (-15.51, -5.65) | 0.71  (-3.32, 4.54) |
| Subarachnoid haemorrhage | 0.19 | 13.35 | 13.32 | 0.24  (0.23, 0.26) ^#1^ | 13.37  (12.61, 14.43) | 12.69  (12.46, 12.95) | 28.75  (21.43, 38.97) | 21.88  (14.95, 31.54) | -4.94  (-6.67, -3) |
| All stroke | 4.25 | 178.19 | 177.11 | 3.94  (3.7, 4.19) | 217.47  (204.13, 231.24) | 170.31  (163.83, 177.14) | -7.29  (-12.94, -1.41) | -12.3  (-17.68, -6.74) | -4.42  (-8.06, -0.59) |
|  |  |  |  |  |  |  |  |  |  |
| **Prevalence** |  |  |  |  |  |  |  |  |  |
| Intracerebral haemorrhage | 6.2 | 361.78 | 374.62 | 6.85  (6.62, 7.09) | 378.53  (365.59, 391.5) | 357.33  (346.95, 370.22) | 10.56  (6.78, 14.35) | 4.63  (1.05, 8.21) | -4.62  (-7.39, -1.17) |
| Ischaemic stroke | 23.25 | 1356.85 | 961.6 | 28.44  (27.24, 29.65) | 1570.40  (1504.44, 1637.59) | 1038.91  (1004.27, 1093) | 22.3  (17.16, 27.53) | 15.74  (10.88, 20.69) | 8.04  (4.44, 13.66) |
| Subarachnoid haemorrhage | 2.08 | 121.18 | 170.99 | 3.23  (3, 3.58) | 178.11  (165.66, 197.57) | 161.12  (157.72, 164.82) | 55.32  (44.46, 72.28) | 46.98  (36.71, 63.04) | -5.77  (-7.76, -3.61) |
| All stroke | 35.86 | 2092.51 | 808.22 | 50.05  (47.66, 51.87) | 2763.86  (2632.06, 2864.27) | 858.59  (828.83, 881.3) | 39.57  (32.91, 44.65) | 32.08  (25.78, 36.88) | 6.23  (2.55, 9.04) |
|  |  |  |  |  |  |  |  |  |  |
| **Death** |  |  |  |  |  |  |  |  |  |
| Intracerebral haemorrhage | 1.32 | 76.78 | 78.12 | 0.80  (0.73, 0.89) | 44.15  (40.08, 49.09) | 68.36  (64.02, 74.6) | -39.24  (-44.83, -32.44) | -42.5  (-47.8, -36.06) | -12.49  (-18.05, -4.51) |
| Ischaemic stroke | 0.82 | 47.72 | 28.09 | 0.72  (0.64, 0.84) | 39.89  (35.08, 46.52) | 30.81  (28.16, 33.95) | -11.66  (-22.32, 3.01) | -16.41  (-26.49, -2.51) | 9.68  (0.25, 20.86) |
| Subarachnoid haemorrhage | 0.1 | 5.73 | 6.31 | 0.14  (0.13, 0.15) ^#2^ | 7.81  (7.03, 8.48) | 5.97  (5.72, 6.3) | 44.01  (29.53, 56.3) | 36.3  (22.69, 47.99) | -5.39  (-9.35, -0.16) |
| All stroke | 2.23 | 130.24 | 112.52 | 1.66  (1.49, 1.88) | 91.86  (82.19, 104.08) | 105.13  (97.9, 114.86) | -25.56  (-33.18, -15.7) | -29.47  (-36.89, -20.09) | -6.57  (-12.99, 2.08) |
|  |  |  |  |  |  |  |  |  |  |
| **DALYs** | | | | | | | | | |
| Intracerebral haemorrhage | 36.07 | 2104.8 | 2320.82 | 24.69  (22.2, 27.9) | 1363.65  (1226.13, 1540.85) | 1919.24  (1822.45, 2011.28) | -31.54  (-38.44, -22.64) | -35.21  (-41.75, -26.79) | -17.3  (-21.47, -13.34) |
| Ischaemic stroke | 22.64 | 1321.04 | 829.51 | 21.64  (18.82, 24.17) | 1194.98  (1039.59, 1334.84) | 752.34  (699.92, 823.64) | -4.41  (-16.84, 6.77) | -9.54  (-21.31, 1.04) | -9.3  (-15.62, -0.71) |
| Subarachnoid haemorrhage | 3.25 | 189.61 | 252.48 | 4.54  (4.19, 4.95) | 250.80  (231.24, 273.32) | 251.75  (241.52, 265.34) | 39.77  (28.87, 52.32) | 32.27  (21.96, 44.15) | -0.29  (-4.34, 5.09) |
| All stroke | 61.95 | 3615.46 | 3402.81 | 50.87  (45.21, 57.02) | 2809.43  (2496.96, 3149.01) | 2923.34  (2763.89, 3100.26) | -17.89  (-27.02, -7.96) | -22.29  (-30.94, -12.9) | -14.09  (-18.78, -8.89) |
|  |  |  |  |  |  |  |  |  |  |
| **Central Europe, Eastern Europe, and Central Asia** | | | | | | | | | |
| **Incidence** |  |  |  |  |  |  |  |  |  |
| Intracerebral haemorrhage | 0.14 | 43.29 | 45.34 | 0.15  (0.14, 0.16) | 46.5  (43.88, 49.44) | 34.97  (33.00, 37.59) | 3.9  (-1.95, 10.48) | 7.42  (1.36, 14.21) | -22.87  (-27.22, -17.09) |
| Ischaemic stroke | 0.56 | 168.36 | 130.37 | 0.54  (0.52, 0.55) | 167.23  (162.07, 172.08) | 106.08  (101.61, 110.39) | -3.92  (-6.89, -1.14) | -0.67  (-3.74, 2.21) | -18.63  (-22.06, -15.33) |
| Subarachnoid haemorrhage | 0.04 | 11.59 | 10.94 | 0.04  (0.04, 0.04) ^#3^ | 12.01  (11.7, 12.25) | 10.02  (9.78, 10.20) | 0.2  (-2.32, 2.27) | 3.62  (0.95, 5.69) | -8.41  (-10.6, -6.76) |
| All stroke | 0.74 | 223.24 | 186.66 | 0.73  (0.70, 0.76) | 225.74  (217.65, 233.77) | 151.07  (144.39, 158.18) | -1.35  (-5.41, 1.35) | 1.12  (-2.5, 4.72) | -19.07  (-22.65, -15.26) |
|  |  |  |  |  |  |  |  |  |  |
| **Prevalence** |  |  |  |  |  |  |  |  |  |
| Intracerebral haemorrhage | 0.66 | 198.79 | 224.58 | 0.68  (0.65, 0.70) | 210.02  (202.23, 217.03) | 198.33  (188.8, 206.1) | 2.18  (-1.61, 5.6) | 5.65  (1.73, 9.18) | -11.69  (-15.93, -8.23) |
| Ischaemic stroke | 4.94 | 1484.24 | 1268.58 | 5.05  (4.94, 5.19) | 1568.43  (1534.59, 1612.69) | 1105.54  (1066.90, 1144.55) | 2.21  (0.01, 5.1) | 5.67  (3.39, 8.65) | -12.85  (-15.9, -9.78) |
| Subarachnoid haemorrhage | 0.42 | 127.15 | 130.05 | 0.46  (0.45, 0.47) | 142.15  (139.26, 145.26) | 129.97  (126.8, 133.98) | 8.14  (5.94, 10.51) | 11.8  (9.52, 14.24) | -0.06  (-2.5, 3.02) |
| All stroke | 6.78 | 2035.05 | 893.88 | 7.41  (7.25, 7.62) | 2300.81  (2251.77, 2364.46) | 857.1  (761.96, 887.5) | 9.29  (6.93, 12.39) | 13.06  (10.65, 16.19) | -4.11  (-14.76, -0.71) |
|  |  |  |  |  |  |  |  |  |  |
| **Death** |  |  |  |  |  |  |  |  |  |
| Intracerebral haemorrhage | 0.12 | 34.77 | 35.16 | 0.11  (0.10, 0.11)^#4^ | 32.86  (31.39, 34.25) | 19.99  (18.88, 21.19) | -8.58  (-12.68, -4.72) | -5.49  (-9.72, -1.5) | -43.15  (-46.3, -39.73) |
| Ischaemic stroke | 0.22 | 67.17 | 42.97 | 0.15  (0.13, 0.18) | 47.72  (41.49, 55.69) | 20.90  (19.45, 22.36) | -31.28  (-40.26, -19.8) | -28.96  (-38.23, -17.09) | -51.36  (-54.74, -47.96) |
| Subarachnoid haemorrhage | 0.02 | 6.37 | 5.27 | 0.02  (0.02, 0.02)^#5^ | 6.07  (5.78, 6.34) | 4.19  (4.05, 4.35) | -7.91  (-12.28, -3.74) | -4.71  (-9.26, -0.47) | -20.49  (-23.15, -17.46) |
| All stroke | 0.36 | 108.31 | 83.39 | 0.28  (0.25, 0.31) | 86.65  (78.65, 96.29) | 45.08  (42.38, 47.9) | -22.22  (-30.56, -13.89) | -20  (-27.38, -11.1) | -45.94  (-49.18, -42.56) |
|  |  |  |  |  |  |  |  |  |  |
| **DALYs** |  |  |  |  |  |  |  |  |  |
| Intracerebral haemorrhage | 3.28 | 986.2 | 1003.06 | 3.29  (3.10, 3.50) | 1021.49  (962.03, 1088.13) | 778.19  (725, 826.15) | 0.19  (-5.65, 6.72) | 3.58  (-2.45, 10.34) | -22.42  (-27.72, -17.64) |
| Ischaemic stroke | 5.86 | 1760.61 | 1196.08 | 6.21  (5.77, 6.66) | 1928.59  (1792.27, 2067.31) | 892.54  (829.23, 964.49) | 5.95  (-1.54, 13.57) | 9.54  (1.8, 17.42) | -25.38  (-30.67, -19.36) |
| Subarachnoid haemorrhage | 0.73 | 217.89 | 192.94 | 0.73  (0.70, 0.75) | 225.52  (217.85, 233.24) | 174.34  (168.84, 181.02) | 0.11  (-3.29, 3.54) | 3.5  (-0.02, 7.04) | -9.64  (-12.49, -6.18) |
| All stroke | 9.87 | 2964.7 | 2392.08 | 10.23  (9.57, 10.92) | 3175.60  (2972.15, 3388.68) | 1845.07  (1723.07, 1971.66) | 3.65  (-3.04, 10.64) | 7.11  (0.25, 14.3) | -22.87  (-27.97, -17.58) |
|  |  |  |  |  |  |  |  |  |  |
| **Latin America and Caribbean** | | | | | | | | | |
| **Incidence** |  |  |  |  |  |  |  |  |  |
| Intracerebral haemorrhage | 0.10 | 22.01 | 26.08 | 0.14  (0.12, 0.15) | 26.09  (23.28, 28.49) | 21.60  (19.94, 23.14) | 39.6  (24.54, 52.42) | 18.54  (5.77, 29.44) | -17.18  (-23.54, -11.27) |
| Ischaemic stroke | 0.26 | 57.33 | 52.88 | 0.45  (0.43, 0.47) | 85.49  (81.83, 89.71) | 52.59  (50.09, 54.76) | 75.6  (68.09, 84.26) | 49.12  (42.74, 56.48) | -0.55  (-5.28, 3.56) |
| Subarachnoid haemorrhage | 0.06 | 13.14 | 11.35 | 0.08  (0.07, 0.08)^#6^ | 14.49  (14.18, 14.97) | 12.35  (12.18, 12.5) | 29.87  (27.12, 34.15) | 10.27  (7.91, 13.93) | 8.81  (7.31, 10.13) |
| All stroke | 0.41 | 92.47 | 90.31 | 0.66  (0.63, 0.7) | 126.07  (119.29, 133.16) | 86.53  (82.21, 90.41) | 60.98  (53.66, 70.73) | 36.34  (29, 44) | -4.19  (-8.97, 0.11) |
|  |  |  |  |  |  |  |  |  |  |
| **Prevalence** |  |  |  |  |  |  |  |  |  |
| Intracerebral haemorrhage | 0.71 | 159.67 | 164.98 | 0.96  (0.92, 1.01) | 182.53  (174.64, 191.84) | 176.94  (168.98, 187.09) | 34.62  (28.79, 41.48) | 14.32  (9.38, 20.15) | 7.25  (2.42, 13.4) |
| Ischaemic stroke | 2.97 | 664.69 | 601.3 | 5.09  (4.96, 5.24) | 968.9  (943.8, 996.2) | 675.69  (648.68, 707.49) | 71.65  (67.2, 76.49) | 45.77  (41.99, 49.87) | 12.37  (7.88, 17.66) |
| Subarachnoid haemorrhage | 0.76 | 170.21 | 158.72 | 1.02  (1.00, 1.04) | 193.85  (190.47, 197.23) | 172.82  (170.07, 176.16) | 34.11  (31.77, 36.45) | 13.89  (11.9, 15.87) | 8.88  (7.15, 10.99) |
| All stroke | 5.07 | 1134.98 | 518.38 | 8.32  (8.11, 8.5) | 1583.88  (1543.96, 1618.07) | 604.27  (537.96, 631.54) | 64.1  (59.96, 67.65) | 39.55  (36.03, 42.56) | 16.57  (3.78, 21.83) |
|  |  |  |  |  |  |  |  |  |  |
| **Death** |  |  |  |  |  |  |  |  |  |
| Intracerebral haemorrhage | 0.08 | 18.40 | 24.69 | 0.12  (0.11, 0.13)^#7^ | 22.67  (21.21, 24.02) | 19.82  (18.59, 21.25) | 45.05  (35.72, 53.7) | 23.21  (15.27, 30.54) | -19.72  (-24.71, -13.93) |
| Ischaemic stroke | 0.07 | 14.82 | 16.51 | 0.11  (0.11, 0.12)^#8^ | 21.84  (20.01, 23.6) | 13.66  (12.83, 14.39) | 73.52  (58.92, 87.51) | 47.37  (35.02, 59.24) | -17.26  (-22.29, -12.84) |
| Subarachnoid haemorrhage | 0.03 | 6.01 | 5 | 0.04  (0.04, 0.04)^#9^ | 7.36  (7.04, 7.72) | 5.47  (5.34, 5.58) | 44.31  (38.13, 51.43) | 22.46  (17.14, 28.45) | 9.4  (6.8, 11.6) |
| All stroke | 0.18 | 39.23 | 46.2 | 0.27  (0.25, 0.29) | 51.87  (48.26, 55.35) | 38.94  (36.75, 41.22) | 50  (38.89, 61.11) | 32.22  (23.02, 41.09) | -15.71  (-20.45, -10.78) |
|  |  |  |  |  |  |  |  |  |  |
| **DALYs** |  |  |  |  |  |  |  |  |  |
| Intracerebral haemorrhage | 2.44 | 545.88 | 698.48 | 2.97  (2.64, 3.33) | 564.62  (501.45, 634.34) | 490.91  (444.83, 535.34) | 21.8  (8.17, 36.84) | 3.43  (-8.14, 16.21) | -29.72  (-36.31, -23.36) |
| Ischaemic stroke | 1.74 | 388.87 | 417.67 | 3.29  (2.81, 3.84) | 626.64  (533.82, 730.21) | 339.53  (292.28, 399.1) | 89.76  (61.65, 121.12) | 61.14  (37.27, 87.78) | -18.71  (-30.02, -4.45) |
| Subarachnoid haemorrhage | 0.98 | 218.75 | 180.92 | 1.29  (1.23, 1.35) | 245.07  (234.7, 256.24) | 200.47  (193.6, 210.89) | 31.92  (26.34, 37.94) | 12.03  (7.29, 17.14) | 10.81  (7.01, 16.57) |
| All stroke | 5.15 | 1153.5 | 1297.07 | 7.55  (6.67, 8.52) | 1436.33  (1269.96, 1620.79) | 1030.90  (930.71, 1145.33) | 46.60  (29.51, 65.44) | 24.52  (10.10, 40.51) | -20.52  (-28.25, -11.70) |
|  |  |  |  |  |  |  |  |  |  |
| **North Africa and Middle East** | | | | | | | | | |
| **Incidence** |  |  |  |  |  |  |  |  |  |
| Intracerebral haemorrhage | 0.10 | 21.88 | 23.35 | 0.17  (0.16, 0.18) | 27.43  (26.28, 28.66) | 22.88  (21.99, 23.82) | 74.39  (67.03, 82.16) | 25.37  (20.11, 30.99) | -2.01  (-5.82, 2.01) |
| Ischaemic stroke | 0.37 | 83.96 | 91.31 | 0.62  (0.61, 0.64) | 101.06  (99.28, 103.93) | 83.77  (81.57, 86.01) | 67.42  (64.48, 72.18) | 20.37  (18.25, 23.79) | -8.26  (-10.67, -5.8) |
| Subarachnoid haemorrhage | 0.03 | 5.93 | 6.34 | 0.04  (0.04, 0.04) ^#10^ | 6.13  (6.06, 6.2) | 6.27  (6.19, 6.36) | 43.76  (42.21, 45.39) | 3.37  (2.19, 4.55) | -1.1  (-2.37, 0.32) |
| All stroke | 0.5 | 111.77 | 120.99 | 0.83  (0.81, 0.86) | 134.62  (131.62, 138.78) | 112.92  (109.75, 116.19) | 66.00  (62.00, 72.00) | 20.44  (17.76, 24.17) | -6.67  (-9.29, -3.97) |
|  |  |  |  |  |  |  |  |  |  |
| **Prevalence** |  |  |  |  |  |  |  |  |  |
| Intracerebral haemorrhage | 0.73 | 165.61 | 174.33 | 1.06  (1.03, 1.08) | 171.24  (167.13, 175.68) | 167.80  (164.21, 171.57) | 43.82  (40.37, 47.55) | 3.4  (0.92, 6.08) | -3.75  (-5.81, -1.58) |
| Ischaemic stroke | 3.96 | 894.93 | 979.54 | 6.56  (6.45, 6.69) | 1064.12  (1046.88, 1085) | 960.28  (938.66, 981.69) | 65.39  (62.71, 68.63) | 18.91  (16.98, 21.24) | -1.97  (-4.17, 0.22) |
| Subarachnoid haemorrhage | 0.39 | 86.91 | 93.45 | 0.60  (0.58, 0.62) | 96.92  (94.83, 99.82) | 97.96  (95.87, 99.66) | 55.12  (51.77, 59.76) | 11.52  (9.11, 14.85) | 4.83  (2.59, 6.65) |
| All stroke | 4.47 | 1008.99 | 628.18 | 9.17  (9.02, 9.34) | 1488.05  (1463.31, 1515.29) | 683.38  (643.55, 707.43) | 105.15  (101.79, 108.95) | 47.48  (45.03, 50.18) | 8.79  (2.45, 12.62) |
|  |  |  |  |  |  |  |  |  |  |
| **Death** |  |  |  |  |  |  |  |  |  |
| Intracerebral haemorrhage | 0.08 | 18.91 | 21.05 | 0.14  (0.13, 0.14)^#11^ | 22.6  (21.64, 23.35) | 17.59  (17.06, 18.41) | 66.31  (59.24, 71.83) | 19.51  (14.44, 23.48) | -16.44  (-18.95, -12.54) |
| Ischaemic stroke | 0.15 | 33.38 | 36.61 | 0.23  (0.22, 0.24) | 37.05  (34.94, 39.33) | 25.76  (24.49, 27.1) | 54.39  (45.58, 63.87) | 10.99  (4.67, 17.83) | -29.64  (-33.11, -25.98) |
| Subarachnoid haemorrhage | 0.01 | 2.13 | 2.12 | 0.02  (0.01, 0.02)^#12^ | 2.45  (2.37, 2.53) | 1.99  (1.93, 2.05) | 59.62  (54.59, 65.43) | 15.02  (11.27, 18.78) | -6.13  (-8.96, -3.3) |
| All stroke | 0.24 | 54.42 | 59.78 | 0.38  (0.36, 0.40) | 62.1  (58.95, 65.22) | 45.34  (43.48, 47.56) | 58.33  (50, 66.67) | 14.11  (8.32, 19.85) | -24.16  (-27.27, -20.44) |
|  |  |  |  |  |  |  |  |  |  |
| **DALYs** |  |  |  |  |  |  |  |  |  |
| Intracerebral haemorrhage | 2.67 | 602.74 | 623.19 | 4.78  (4.54, 5.02) | 776.44  (736.95, 815.16) | 611.62  (585.72, 638.5) | 79.18  (70.07, 88.11) | 28.82  (22.27, 35.24) | -1.86  (-6.01, 2.46) |
| Ischaemic stroke | 4.16 | 939.86 | 989.33 | 7.51  (7.12, 7.85) | 1217.97  (1156.13, 1274.24) | 856.14  (809.59, 894.59) | 80.25  (71.1, 88.58) | 29.59  (23.01, 35.58) | -13.46  (-18.17, -9.58) |
| Subarachnoid haemorrhage | 0.36 | 82.29 | 78.43 | 0.69  (0.64, 0.73) | 111.85  (104.55, 117.81) | 89.89  (84.88, 93.37) | 89.07  (76.73, 99.14) | 35.92  (27.05, 43.16) | 14.61  (8.22, 19.05) |
| All stroke | 7.2 | 1624.88 | 1690.95 | 12.98  (12.31, 13.6) | 2106.26  (1997.63, 2207.21) | 1557.65  (1480.18, 1626.46) | 80.28  (70.97, 88.89) | 29.63  (22.94, 35.84) | -7.88  (-12.46, -3.81) |
| **South Asia** | | | | | | | | | |
| **Incidence** |  |  |  |  |  |  |  |  |  |
| Intracerebral haemorrhage | 0.61 | 45.47 | 51.45 | 0.70  (0.65, 0.75) | 37.95  (35.27, 41.05) | 33.15  (31.09, 35.43) | 14.38  (6.32, 23.74) | -16.54  (-22.43, -9.72) | -35.57  (-39.57, -31.14) |
| Ischaemic stroke | 0.67 | 50.21 | 63.33 | 1.86  (1.73, 2.01) | 101.47  (94.35, 109.47) | 72.75  (68.88, 77) | 176.98  (157.52, 198.81) | 102.09  (87.91, 118.02) | 14.87  (8.76, 21.59) |
| Subarachnoid haemorrhage | 0.13 | 9.45 | 10.66 | 0.18  (0.17, 0.18) ^#13^ | 9.68  (9.38, 9.93) | 9.58  (9.37, 9.84) | 40.32  (35.96, 44.06) | 2.43  (-0.74, 5.08) | -10.13  (-12.1, -7.69) |
| All stroke | 1.41 | 105.13 | 125.44 | 2.73  (2.55, 2.94) | 149.1  (139, 160.46) | 115.48  (109.34, 122.26) | 93.62  (80.85, 108.51) | 41.82  (32.22, 52.63) | -7.94  (-12.83, -2.54) |
|  |  |  |  |  |  |  |  |  |  |
| **Prevalence** |  |  |  |  |  |  |  |  |  |
| Intracerebral haemorrhage | 3.29 | 245.5 | 258.23 | 4.27  (4.10, 4.49) | 232.87  (223.77, 244.78) | 228.19  (218.89, 235.1) | 30  (24.91, 36.64) | -5.14  (-8.85, -0.29) | -11.63  (-15.23, -8.96) |
| Ischaemic stroke | 7.13 | 532.43 | 631.68 | 16.72  (15.91, 17.54) | 911.63  (867.39, 956.53) | 742.58  (705.5, 775.43) | 134.65  (123.26, 146.21) | 71.22  (62.91, 79.65) | 17.56  (11.69, 22.76) |
| Subarachnoid haemorrhage | 1.29 | 96.1 | 111.28 | 2.18  (2.07, 2.31) | 118.86  (112.7, 125.92) | 118.68  (113.76, 122.59) | 69.51  (60.72, 79.57) | 23.68  (17.27, 31.03) | 6.65  (2.23, 10.16) |
| All stroke | 12.33 | 921.66 | 537.45 | 25.45  (24.5, 26.65) | 1387.87  (1335.67, 1453.03) | 612.62  (523.09, 650) | 106.41  (98.7, 116.14) | 50.58  (44.92, 57.65) | 13.99  (-2.67, 20.94) |
|  |  |  |  |  |  |  |  |  |  |
| **Death** |  |  |  |  |  |  |  |  |  |
| Intracerebral haemorrhage | 0.49 | 36.34 | 43.29 | 0.57  (0.49, 0.67) | 30.93  (26.69, 36.26) | 28.40  (25.67, 31.53) | 16.64  (0.66, 36.73) | -14.89  (-26.55, -0.22) | -34.4  (-40.7, -27.17) |
| Ischaemic stroke | 0.28 | 20.77 | 28.07 | 0.57  (0.53, 0.62) | 30.81  (28.82, 33.56) | 25.72  (24.35, 27.03) | 103.35  (90.22, 121.47) | 48.34  (38.76, 61.58) | -8.37  (-13.25, -3.71) |
| Subarachnoid haemorrhage | 0.06 | 4.52 | 5.35 | 0.09  (0.08, 0.1)^#14^ | 4.92  (4.49, 5.31) | 4.02  (3.66, 4.32) | 49.27  (36.17, 60.81) | 8.85  (-0.66, 17.48) | -24.86  (-31.59, -19.25) |
| All stroke | 0.82 | 61.63 | 76.71 | 1.22  (1.1, 1.38) | 66.67  (60.01, 75.12) | 58.15  (53.69, 62.88) | 48.78  (34.15, 68.29) | 8.18  (-2.63, 21.89) | -24.2  (-30.01, -18.03) |
|  |  |  |  |  |  |  |  |  |  |
| **DALYs** |  |  |  |  |  |  |  |  |  |
| Intracerebral haemorrhage | 14.07 | 1051.16 | 1193.35 | 15.32  (12.85, 17.30) | 835.28  (700.73, 943.51) | 827.82  (732.37, 908.07) | 8.9  (-8.64, 23.01) | -20.54  (-33.34, -10.24) | -30.63  (-38.63, -23.91) |
| Ischaemic stroke | 7.26 | 542.64 | 701.95 | 15.64  (14, 17.32) | 852.58  (763.32, 944.15) | 719.76  (651.74, 788.03) | 115.32  (92.78, 138.45) | 57.12  (40.67, 73.99) | 2.54  (-7.15, 12.26) |
| Subarachnoid haemorrhage | 2.2 | 164.3 | 187.47 | 2.28  (1.91, 2.54) | 124.07  (104.09, 138.49) | 133.72  (123.68, 145.85) | 3.49  (-13.18, 15.51) | -24.49  (-36.65, -15.71) | -28.67  (-34.03, -22.2) |
| All stroke | 23.53 | 1758.11 | 2082.77 | 33.23  (28.76, 37.16) | 1811.92  (1568.14, 2026.14) | 1681.30  (1507.80, 1841.95) | 41.22  (22.23, 57.93) | 3.06  (-10.81, 15.25) | -19.28  (-27.61, -11.56) |
| **Sub-Saharan Africa** | | | | | | | | | |
| **Incidence** |  |  |  |  |  |  |  |  |  |
| Intracerebral haemorrhage | 0.24 | 38.03 | 59.74 | 0.53  (0.51, 0.55) | 39.45  (37.59, 41.13) | 49.36  (47.85, 50.79) | 117.56  (107.33, 126.82) | 3.73  (-1.16, 8.15) | -17.38  (-19.9, -14.98) |
| Ischaemic stroke | 0.4 | 62.62 | 97.31 | 1.02  (0.98, 1.05) | 75.62  (72.94, 78.02) | 95.81  (93.54, 98.26) | 153.27  (144.31, 161.32) | 20.76  (16.48, 24.59) | -1.54  (-3.87, 0.98) |
| Subarachnoid haemorrhage | 0.04 | 5.53 | 6.85 | 0.08  (0.08, 0.08) ^#15^ | 5.70  (5.59, 5.81) | 6.50  (6.43, 6.57) | 116.4  (112.2, 120.53) | 3.07  (1.08, 5.06) | -5.11  (-6.13, -4.09) |
| All stroke | 0.68 | 106.18 | 163.9 | 1.62  (1.56, 1.68) | 120.77  (116.13, 124.96) | 151.67  (147.81, 155.62) | 138.24  (129.41, 147.06) | 13.74  (9.37, 17.69) | -7.46  (-9.82, -5.05) |
|  |  |  |  |  |  |  |  |  |  |
| **Prevalence** |  |  |  |  |  |  |  |  |  |
| Intracerebral haemorrhage | 1.4 | 218.49 | 271.44 | 3.14  (3.05, 3.22) | 233.37  (226.82, 240.03) | 260.77  (254.84, 266.26) | 124.03  (117.74, 130.42) | 6.81  (3.81, 9.86) | -3.93  (-6.12, -1.91) |
| Ischaemic stroke | 4.71 | 735.45 | 1060.9 | 11.23  (10.82, 11.63) | 835.66  (805.51, 865.96) | 1059.11  (1035.59, 1081.94) | 138.32  (129.73, 146.97) | 13.63  (9.53, 17.75) | -0.17  (-2.39, 1.98) |
| Subarachnoid haemorrhage | 0.5 | 78.82 | 100.52 | 1.33  (1.28, 1.39) | 99.10  (95.37, 103.2) | 111.6  (108.83, 114.76) | 163.71  (153.77, 174.62) | 25.73  (21, 30.93) | 11.02  (8.27, 14.17) |
| All stroke | 7.28 | 1136.67 | 803 | 16.97  (16.5, 17.65) | 1263.20  (1228.45, 1313.66) | 866.36  (658.42, 942.95) | 133.1  (126.65, 142.45) | 11.13  (8.07, 15.57) | 7.89  (-18, 17.43) |
|  |  |  |  |  |  |  |  |  |  |
| **Death** |  |  |  |  |  |  |  |  |  |
| Intracerebral haemorrhage | 0.24 | 37.14 | 72.10 | 0.51  (0.48, 0.54) | 37.77  (35.9, 39.95) | 54.8  (53, 57.66) | 113.31  (102.76, 125.66) | 1.7  (-3.34, 7.57) | -23.99  (-26.49, -20.03) |
| Ischaemic stroke | 0.11 | 17.73 | 38.50 | 0.29  (0.28, 0.30) | 21.83  (21.21, 22.49) | 34.98  (34.18, 35.91) | 158.31  (150.99, 166.14) | 23.12  (19.63, 26.85) | -9.14  (-11.22, -6.73) |
| Subarachnoid haemorrhage | 0.01 | 1.85 | 2.98 | 0.03  (0.03, 0.03)^#16^ | 2.07  (1.98, 2.21) | 2.73  (2.66, 2.81) | 134.9  (124.36, 151.15) | 11.89  (7.03, 19.46) | -8.39  (-10.74, -5.7) |
| All stroke | 0.36 | 56.71 | 113.58 | 0.83  (0.79, 0.87) | 61.67  (59.09, 64.66) | 92.52  (89.84, 96.38) | 130.56  (119.44, 141.67) | 8.75  (4.2, 14.02) | -18.54  (-20.9, -15.14) |
|  |  |  |  |  |  |  |  |  |  |
| **DALYs** |  |  |  |  |  |  |  |  |  |
| Intracerebral haemorrhage | 7.28 | 1137 | 1982.59 | 13.86  (12.8, 14.94) | 1031.81  (952.51, 1112.22) | 1429.58  (1358.57, 1487.28) | 90.34  (75.71, 105.17) | -9.25  (-16.23, -2.18) | -27.89  (-31.47, -24.98) |
| Ischaemic stroke | 3.27 | 511.2 | 998.35 | 8.46  (7.64, 9.07) | 630.06  (568.45, 674.93) | 926.82  (881.2, 967.37) | 158.51  (133.24, 176.93) | 23.25  (11.2, 32.03) | -7.16  (-11.73, -3.1) |
| Subarachnoid haemorrhage | 0.5 | 78.24 | 110.52 | 1.24  (1.14, 1.34) | 92.60  (85.03, 99.67) | 107.04  (100.07, 111.54) | 148.26  (127.95, 167.21) | 18.35  (8.68, 27.39) | -3.15  (-9.46, 0.92) |
| All stroke | 11.06 | 1726.44 | 3091.46 | 23.57  (21.57, 25.35) | 1754.48  (1605.99, 1886.83) | 2463.44  (2339.85, 2566.19) | 113.11  (95.03, 129.2) | 1.62  (-6.98, 9.29) | -20.31  (-24.31, -16.99) |
| **High-income** | | | | | | | | | |
| **Incidence** |  |  |  |  |  |  |  |  |  |
| Intracerebral haemorrhage | 0.19 | 20.29 | 15.92 | 0.24  (0.23, 0.25) | 24.69  (23.7, 25.9) | 14.68  (14, 15.42) | 27.85  (22.76, 34.16) | 21.69  (16.81, 27.65) | -7.79  (-12.06, -3.14) |
| Ischaemic stroke | 0.77 | 84.43 | 58.56 | 1.06  (0.99, 1.14) | 110.18  (102.85, 119.2) | 54.54  (52.22, 56.59) | 37.16  (28.04, 48.4) | 30.5  (21.82, 41.18) | -6.86  (-10.83, -3.36) |
| Subarachnoid haemorrhage | 0.11 | 11.63 | 10.03 | 0.11  (0.10, 0.11) ^#17^ | 11.13  (10.94, 11.35) | 9.55  (9.42, 9.66) | 0.65  (-1.12, 2.59) | -4.3  (-5.93, -2.41) | -4.79  (-6.08, -3.69) |
| All stroke | 1.06 | 116.35 | 84.51 | 1.40  (1.32, 1.50) | 146  (137.49, 156.45) | 78.78  (75.64, 81.66) | 32.08  (24.53, 41.51) | 25.48  (18.17, 34.46) | -6.78  (-10.5, -3.37) |
|  |  |  |  |  |  |  |  |  |  |
| **Prevalence** |  |  |  |  |  |  |  |  |  |
| Intracerebral haemorrhage | 2.07 | 226.76 | 167.87 | 2.55  (2.48, 2.61) | 265.45  (258.78, 271.76) | 170.52  (167.18, 173.75) | 23.04  (19.95, 25.96) | 17.06  (14.12, 19.84) | 1.58  (-0.41, 3.5) |
| Ischaemic stroke | 10.98 | 1202.53 | 734.71 | 15.46  (14.73, 16.31) | 1611.56  (1534.73, 1699.93) | 753.77  (734.6, 781.26) | 40.86  (34.14, 48.58) | 34.01  (27.63, 41.36) | 2.59  (-0.01, 6.34) |
| Subarachnoid haemorrhage | 1.67 | 182.55 | 139.67 | 1.93  (1.88, 1.96) | 200.87  (196.36, 204.06) | 146.35  (144.32, 148.11) | 15.66  (13.06, 17.49) | 10.04  (7.57, 11.78) | 4.78  (3.33, 6.04) |
| All stroke | 19.67 | 2154.44 | 633.88 | 26.67  (25.87, 27.75) | 2779.2  (2696.42, 2892) | 693.78  (540.15, 774.11) | 35.59  (31.52, 41.08) | 29  (25.16, 34.23) | 9.45  (-14.79, 22.12) |
|  |  |  |  |  |  |  |  |  |  |
| **Death** |  |  |  |  |  |  |  |  |  |
| Intracerebral haemorrhage | 0.11 | 12.22 | 8.13 | 0.12  (0.11, 0.13) | 12.64  (11.84, 13.46) | 6.13  (5.86, 6.39) | 8.72  (1.82, 15.77) | 3.44  (-3.11, 10.15) | -24.6  (-27.92, -21.4) |
| Ischaemic stroke | 0.10 | 10.82 | 6.61 | 0.12  (0.12, 0.14) ^#18^ | 12.8  (12.12, 14.31) | 5.47  (5.26, 5.7) | 24.3  (17.74, 38.97) | 18.3  (12.01, 32.26) | -17.25  (-20.42, -13.77) |
| Subarachnoid haemorrhage | 0.04 | 4.14 | 2.98 | 0.04  (0.04, 0.04)^#19^ | 4.49  (4.35, 4.65) | 2.69  (2.64, 2.75) | 13.94  (10.55, 18.12) | 8.45  (5.07, 12.32) | -9.73  (-11.41, -7.72) |
| All stroke | 0.25 | 27.18 | 17.72 | 0.29  (0.27, 0.31) | 29.92  (28.31, 32.42) | 14.30  (13.76, 14.84) | 16  (8, 24) | 10.08  (4.16, 19.28) | -19.3  (-22.35, -16.25) |
|  |  |  |  |  |  |  |  |  |  |
| **DALYs** |  |  |  |  |  |  |  |  |  |
| Intracerebral haemorrhage | 3.01 | 330.11 | 240.31 | 3.77  (3.43, 4.2) | 392.55  (357.74, 438.17) | 210.56  (193.92, 225.94) | 24.99  (13.91, 39.51) | 18.91  (8.37, 32.73) | -12.38  (-19.3, -5.98) |
| Ischaemic stroke | 3.52 | 385.05 | 237.66 | 5.27  (4.59, 6.62) | 549.15  (478.21, 690.31) | 222.52  (200.14, 247.19) | 49.9  (30.54, 88.43) | 42.62  (24.19, 79.28) | -6.37  (-15.79, 4.01) |
| Subarachnoid haemorrhage | 1.32 | 145.12 | 116.33 | 1.69  (1.6, 1.79) | 176.01  (167.06, 186.52) | 119.2  (117.02, 122.13) | 27.49  (21.01, 35.09) | 21.29  (15.12, 28.53) | 2.47  (0.59, 4.99) |
| All stroke | 7.85 | 860.28 | 594.31 | 10.73  (9.62, 12.62) | 1117.72  (1003.01, 1314.99) | 552.28  (511.07, 595.27) | 36.69  (22.55, 60.76) | 29.93  (16.59, 52.86) | -7.07  (-14.01, 0.16) |
|  |  |  |  |  |  |  |  |  |  |

Notes: Absolute number are in millions. The denominator of crude incidence rate and age standardized incidence rate is per 100 000 people. Absolute numbers in millions, crude rates per 100 000 people, age-standardised rates per 100 000 people, and percentage change are presented to two decimal places. UI=uncertainty interval. DALY=dis ability adjusted life-year.

#1: Absolute number of subarachnoid haemorrhage incident cases in Southeast Asia, East Asia, and Oceania in 2050 is 242112(228345, 261333);

#2: Absolute number of subarachnoid haemorrhage dead cases in Southeast Asia, East Asia, and Oceania in 2050 is 141467(127244, 153534);

#3: Absolute number of subarachnoid haemorrhage incident cases in Central Europe, Eastern Europe, and Central Asia in 2050 is 38670(37670, 39469);

#4: Absolute number of intracerebral haemorrhage dead in Central Europe, Eastern Europe, and Central Asia in 2050 is 105851 (101103, 110323);

#5: Absolute number of subarachnoid haemorrhage dead in Central Europe, Eastern Europe, and Central Asia in 2050 is 19539(18613, 20425);

#6: Absolute number of subarachnoid haemorrhage incident cases in Latin America and Caribbean in 2050 is 76145(74533, 78657);

#7: Absolute number of intracerebral haemorrhage dead cases in Latin America and Caribbean in 2050 is 119155(111487, 126257);

#8: Absolute number of ischemic haemorrhage dead cases in Latin America and Caribbean in 2050 is 114805(105144,124060);

#9: Absolute number of subarachnoid haemorrhage dead cases in Latin America and Caribbean in 2050 is 38681(37026, 40591);

#10: Absolute number of subarachnoid haemorrhage incident cases in North Africa and Middle East in 2050 is 38670(37700, 39469);

#11: Absolute number of intracerebral haemorrhage dead in North Africa and Middle East in 2050 is 139296(133373, 143919);

#12: Absolute number of ischemic haemorrhage dead in North Africa and Middle East in 2050 is 228337(215310, 242348);

#13: Absolute number of subarachnoid haemorrhage incident cases in South Asia in 2050 is 177472(171957, 182197);

#14: Absolute number of subarachnoid haemorrhage dead cases in South Asia in 2050 is 90324(82399, 97307);

#15: Absolute number of subarachnoid haemorrhage incident cases in Sub-Saharan Africa in 2050 is 76614(75127, 78078);

#16: Absolute number of subarachnoid haemorrhage dead cases in Sub-Saharan Africa in 2050 is 27816(26568, 29741);

#17: Absolute number of subarachnoid haemorrhage incident cases in high-income countries in 2050 is 106838(104957, 108899);

#18: Absolute number of ischemic haemorrhage dead cases in high-income countries in 2050 is 122796(116319, 137298);

#19: Absolute number of subarachnoid haemorrhage dead cases in high-income countries in 2050 is 43051(41770, 44630).

# Appendix 3. Projected age-standardised rates for prevalence, deaths, and DALYs by stroke type and country in 2050


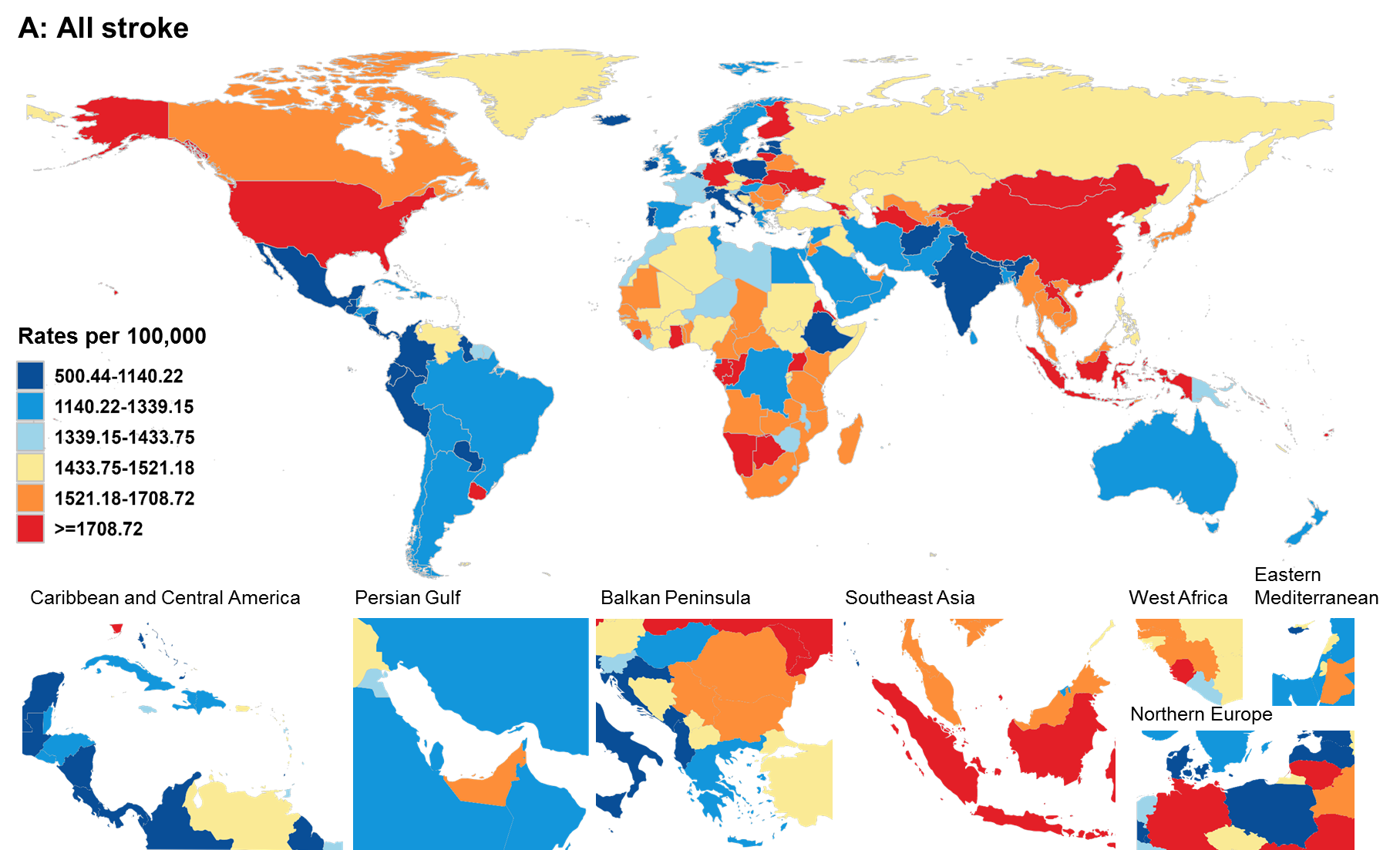

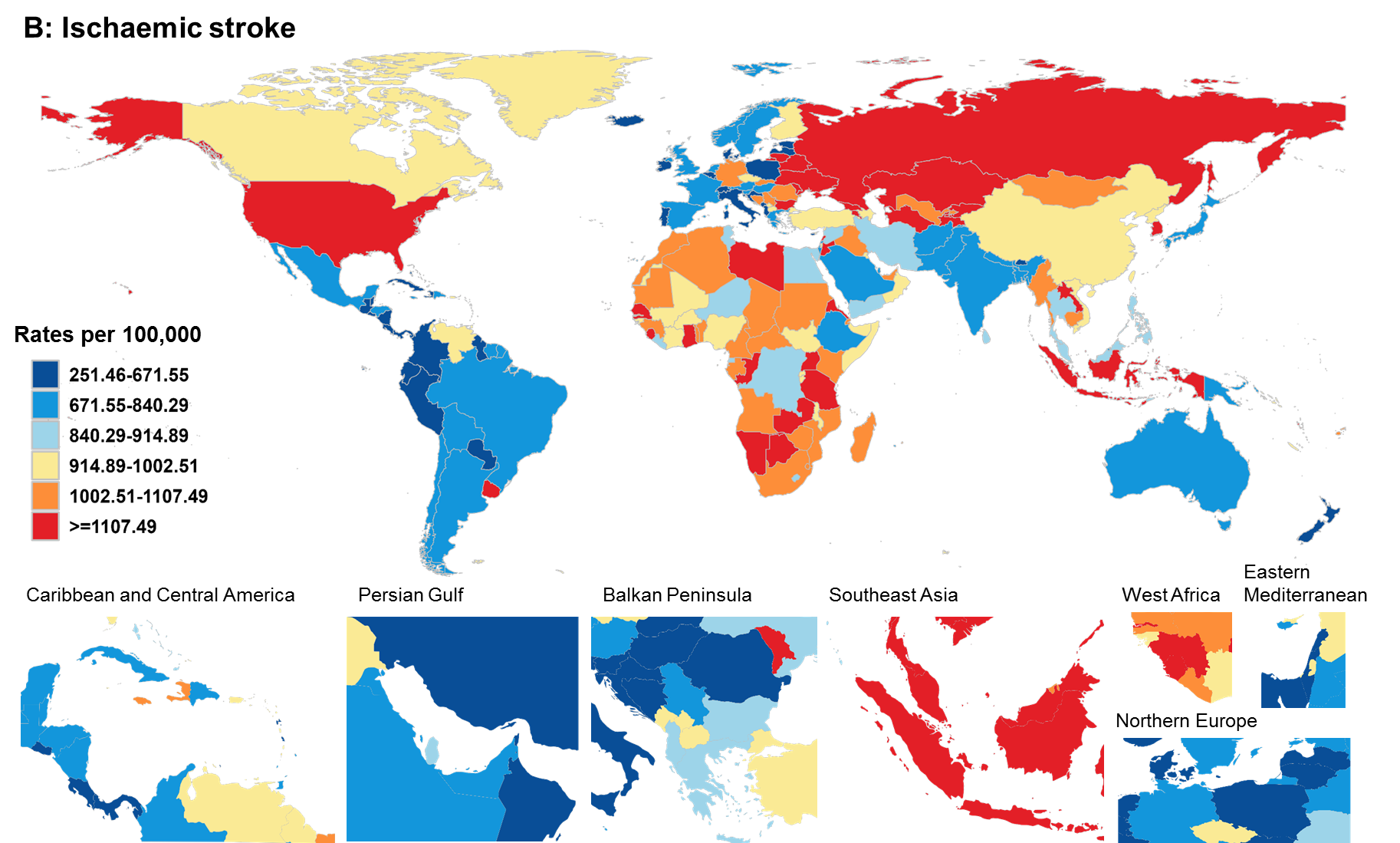

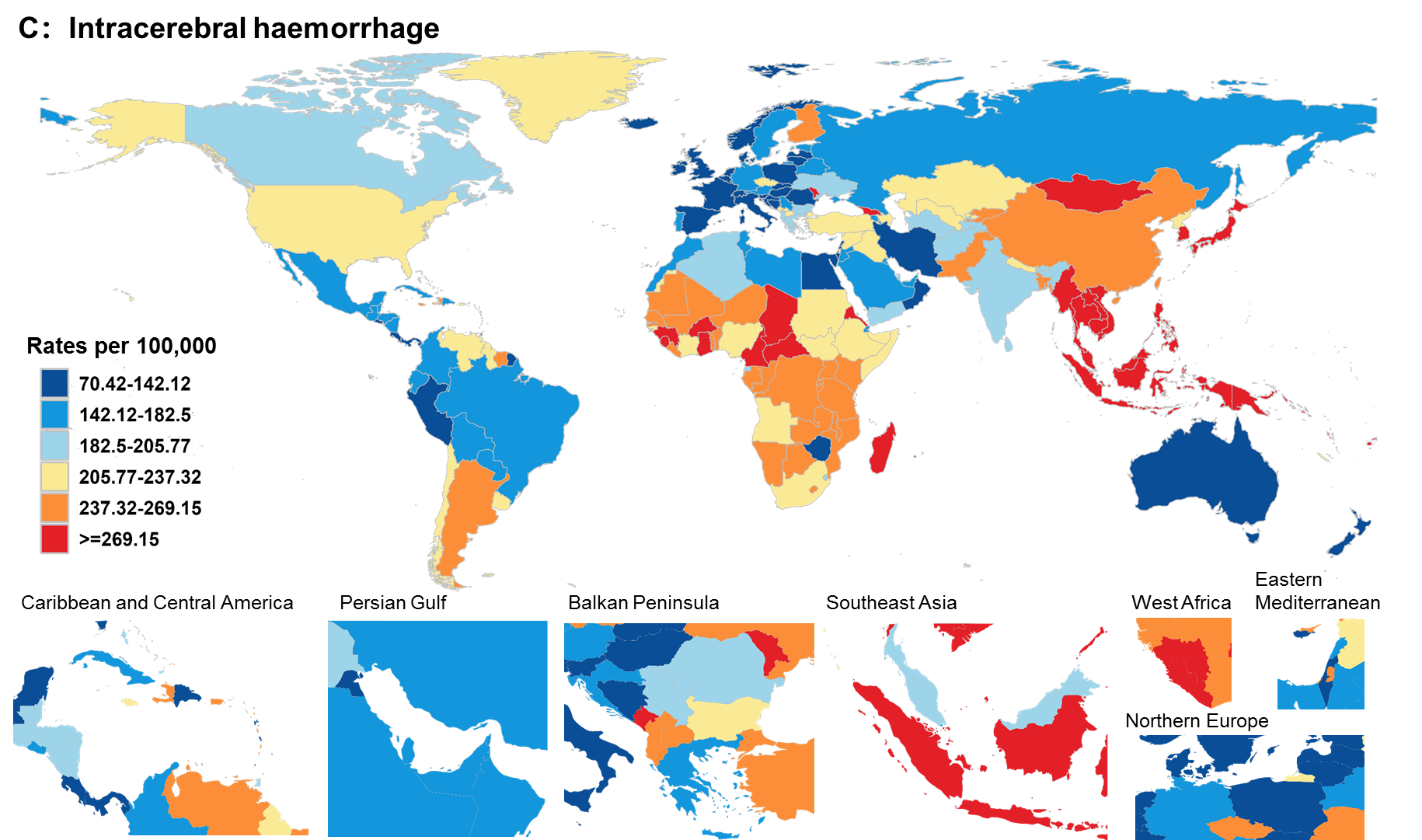

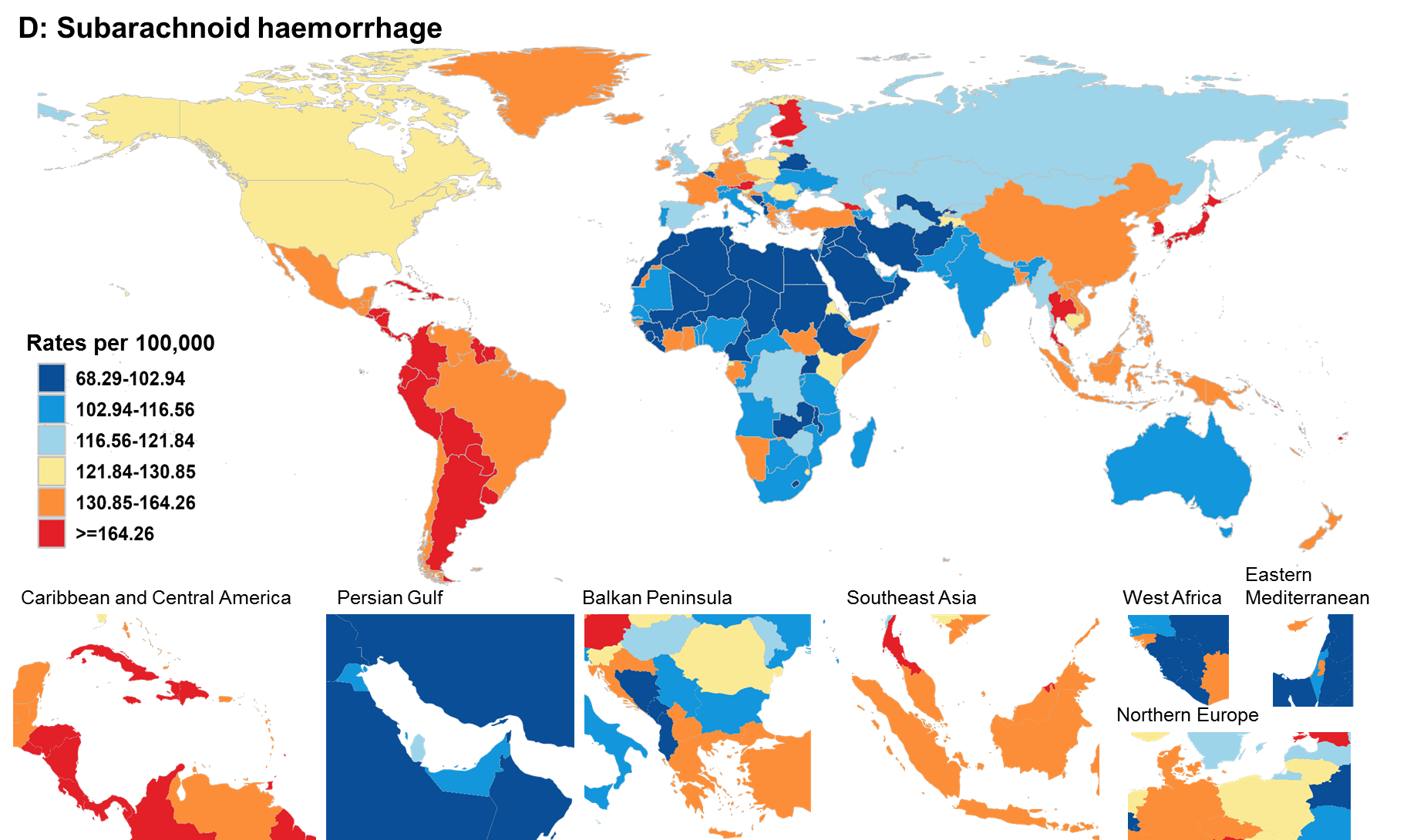


**Fig.S2 Age-standardised prevalence rates per 100 000 people by stroke type and country, for both sexes, 2050.**


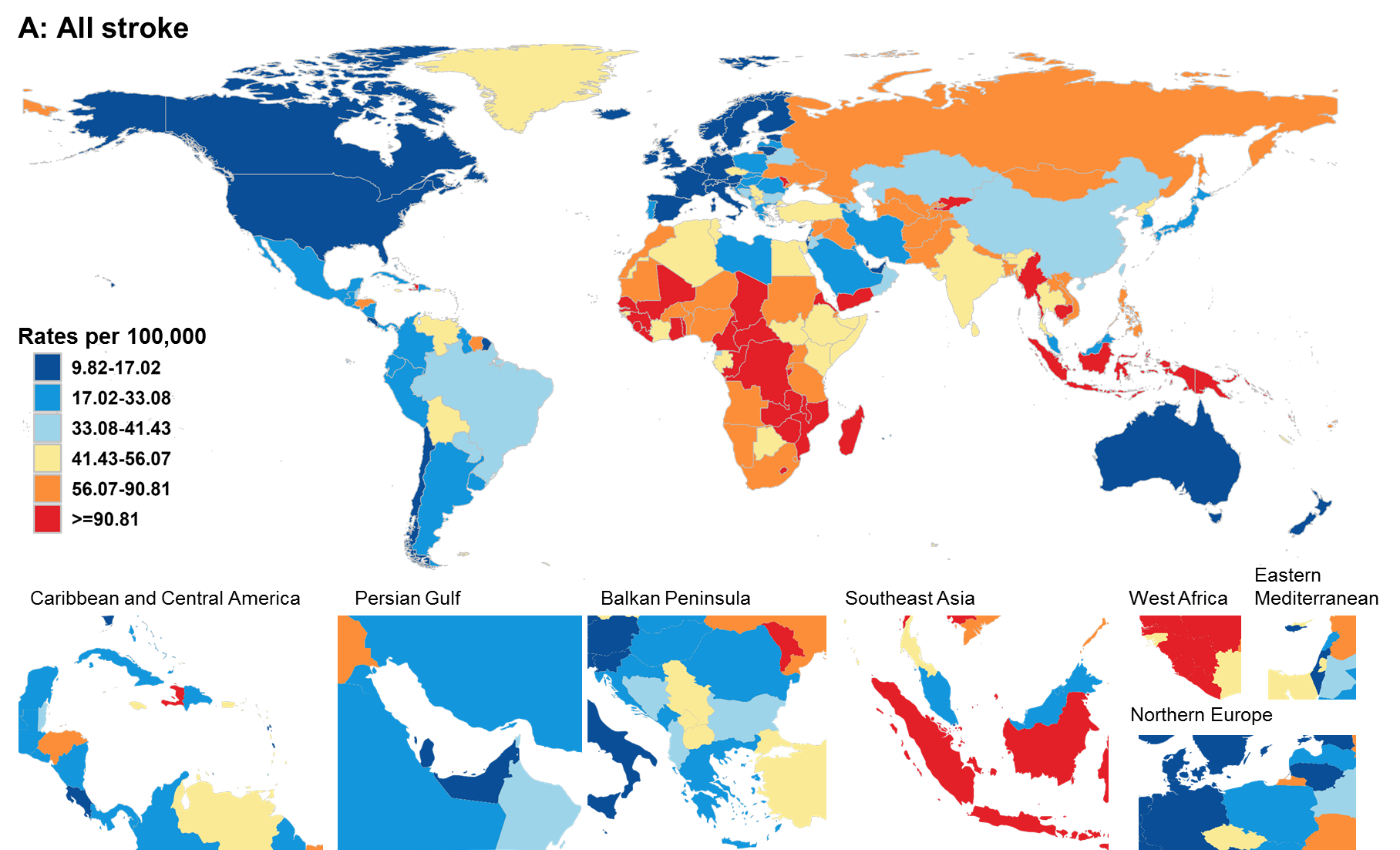

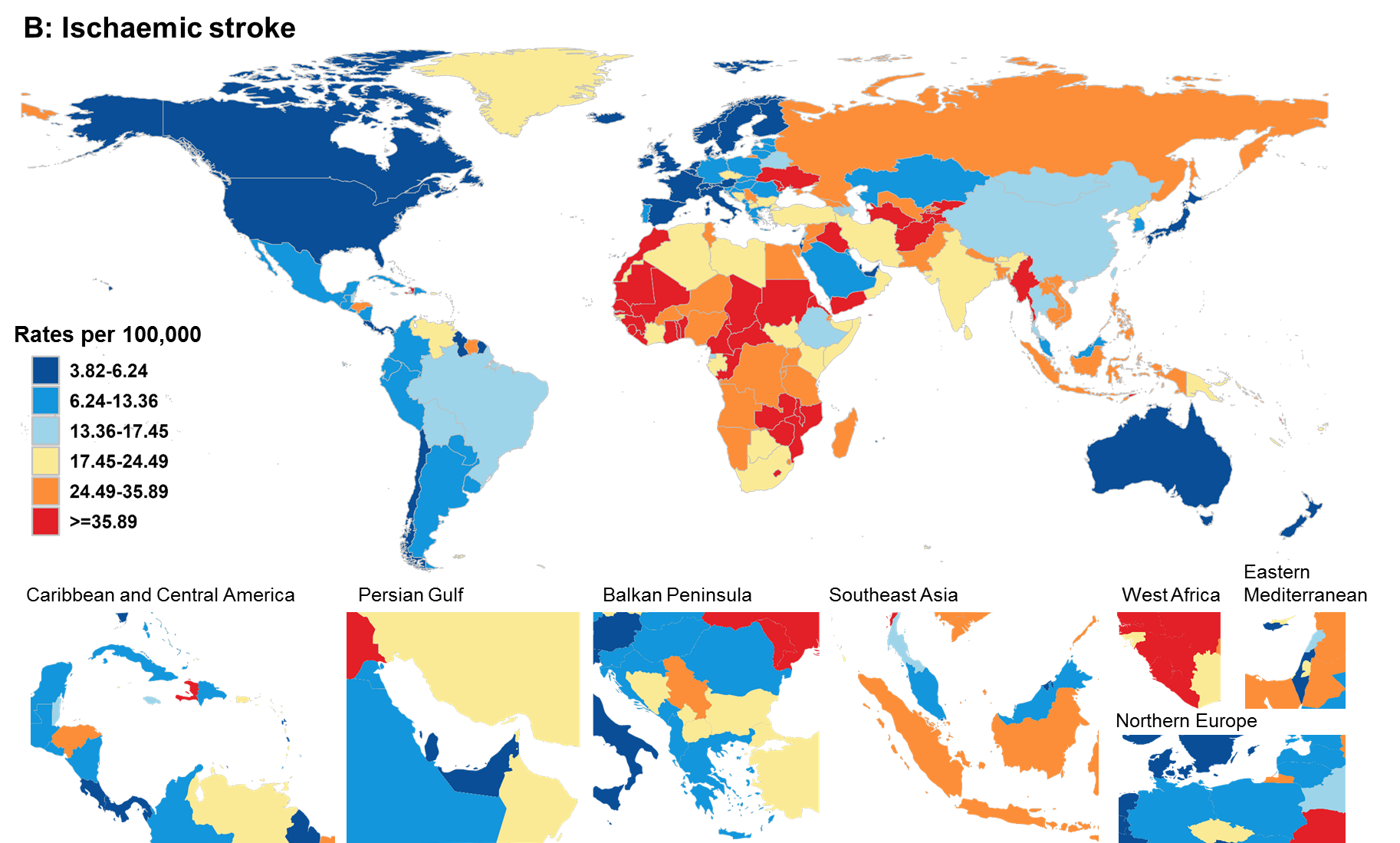

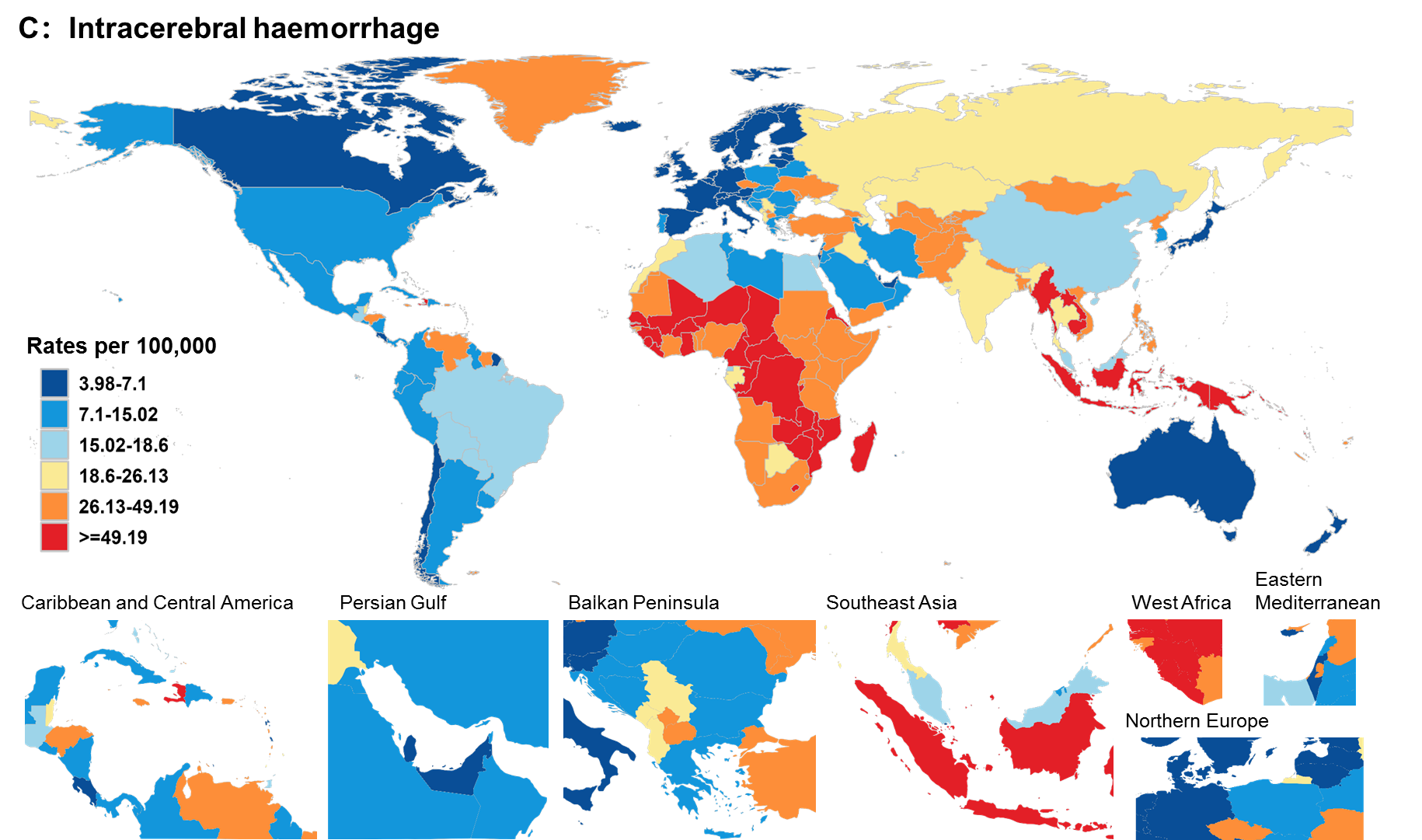

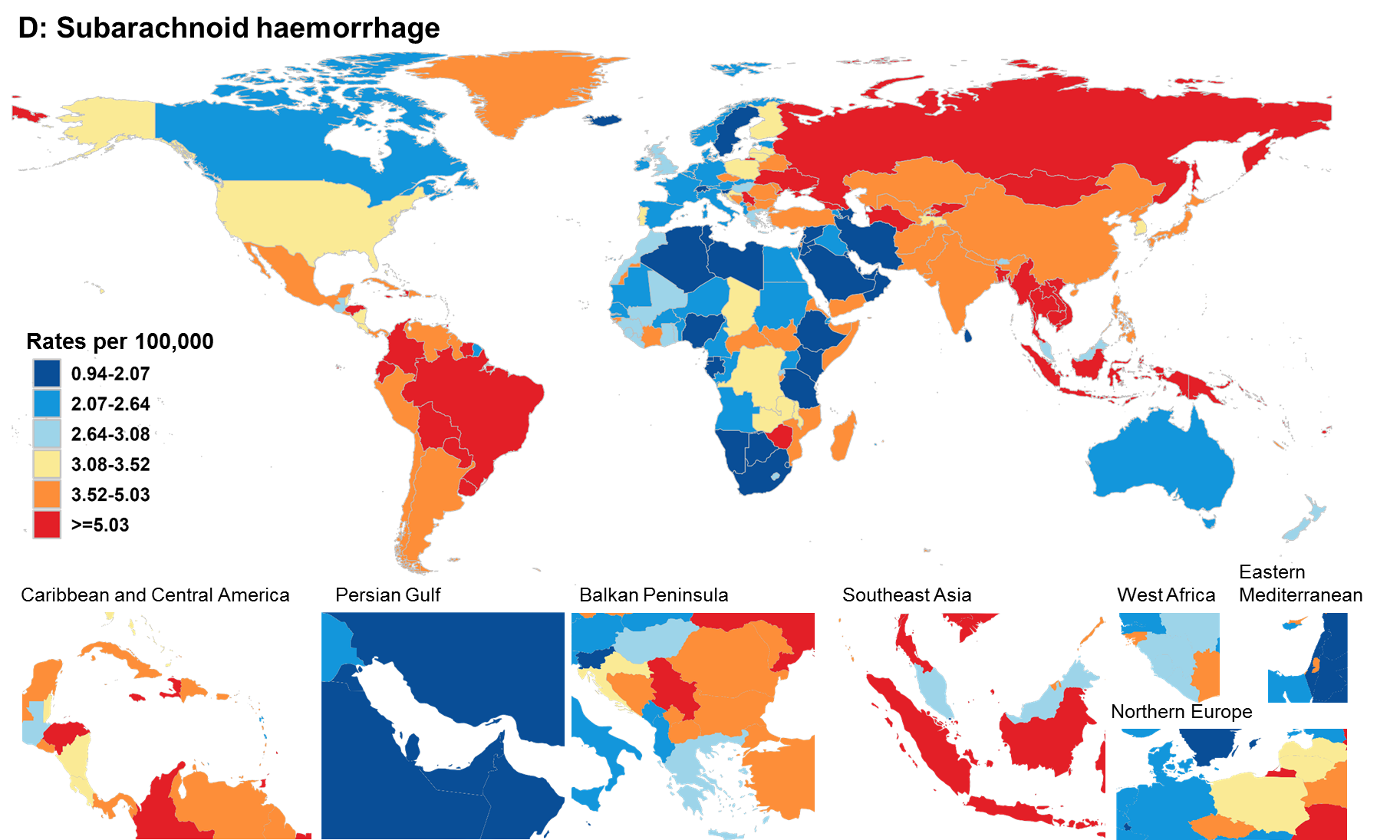


**Fig.S3 Age-standardised deaths rates per 100 000 people by stroke type and country, for both sexes, 2050.**


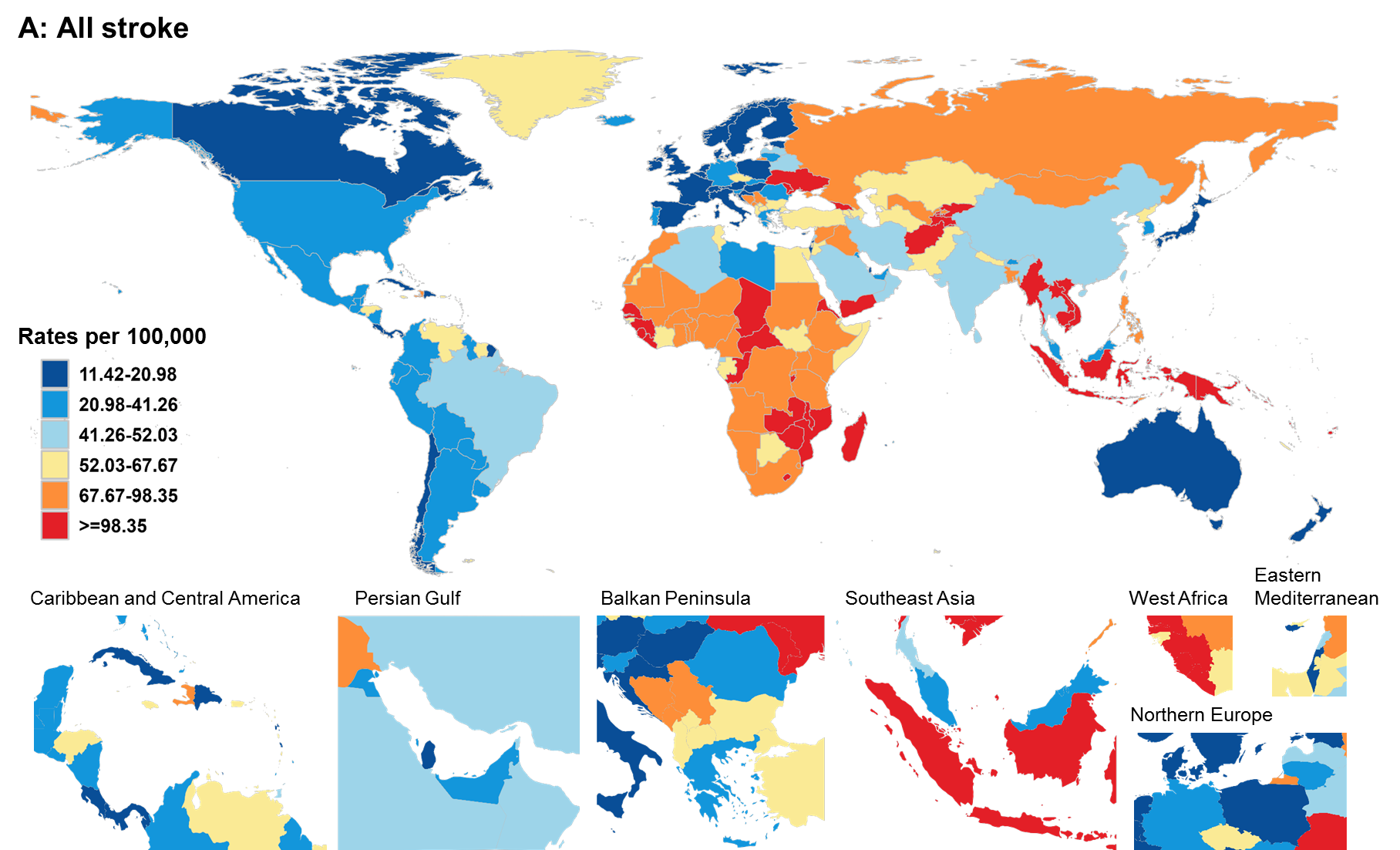

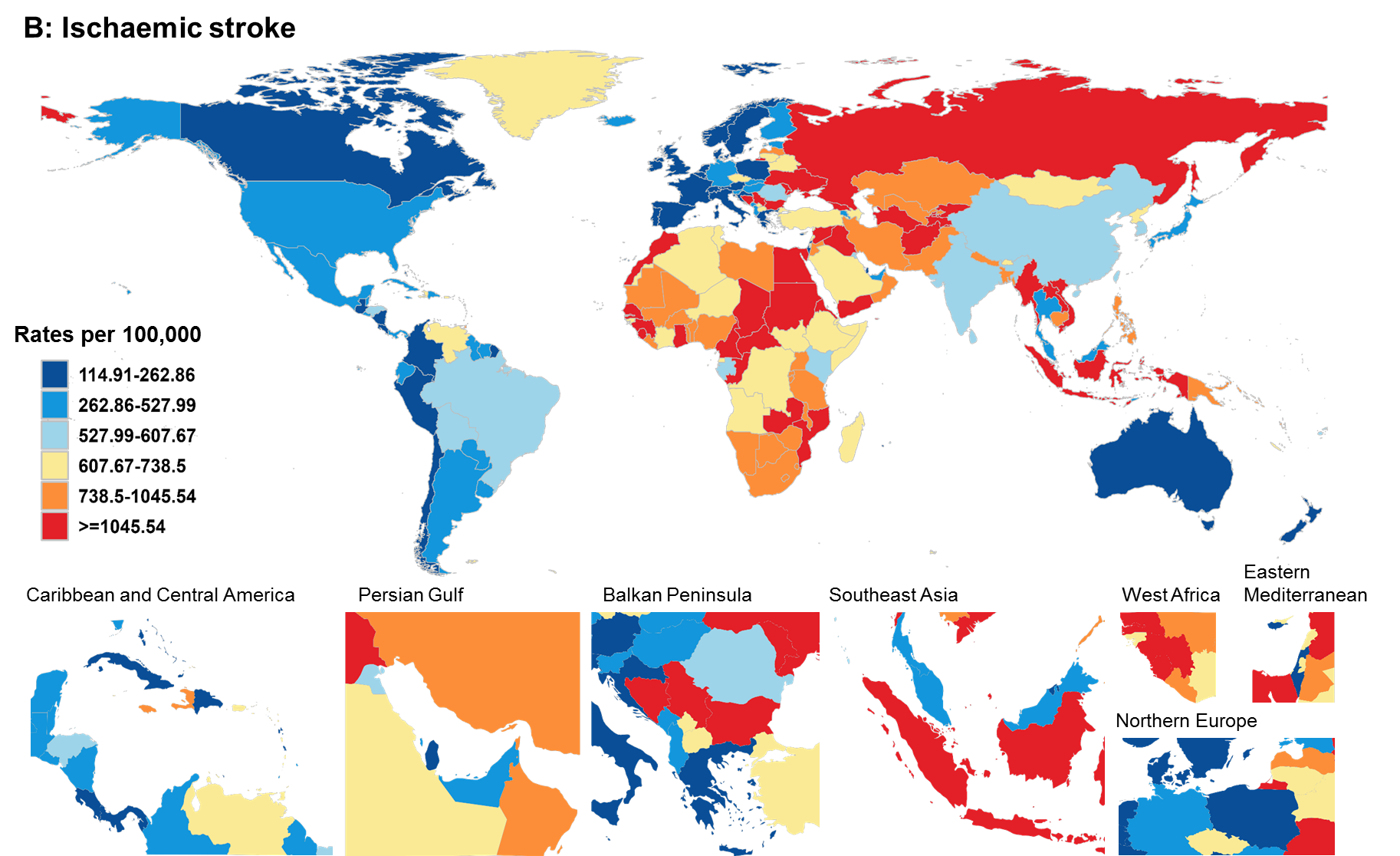

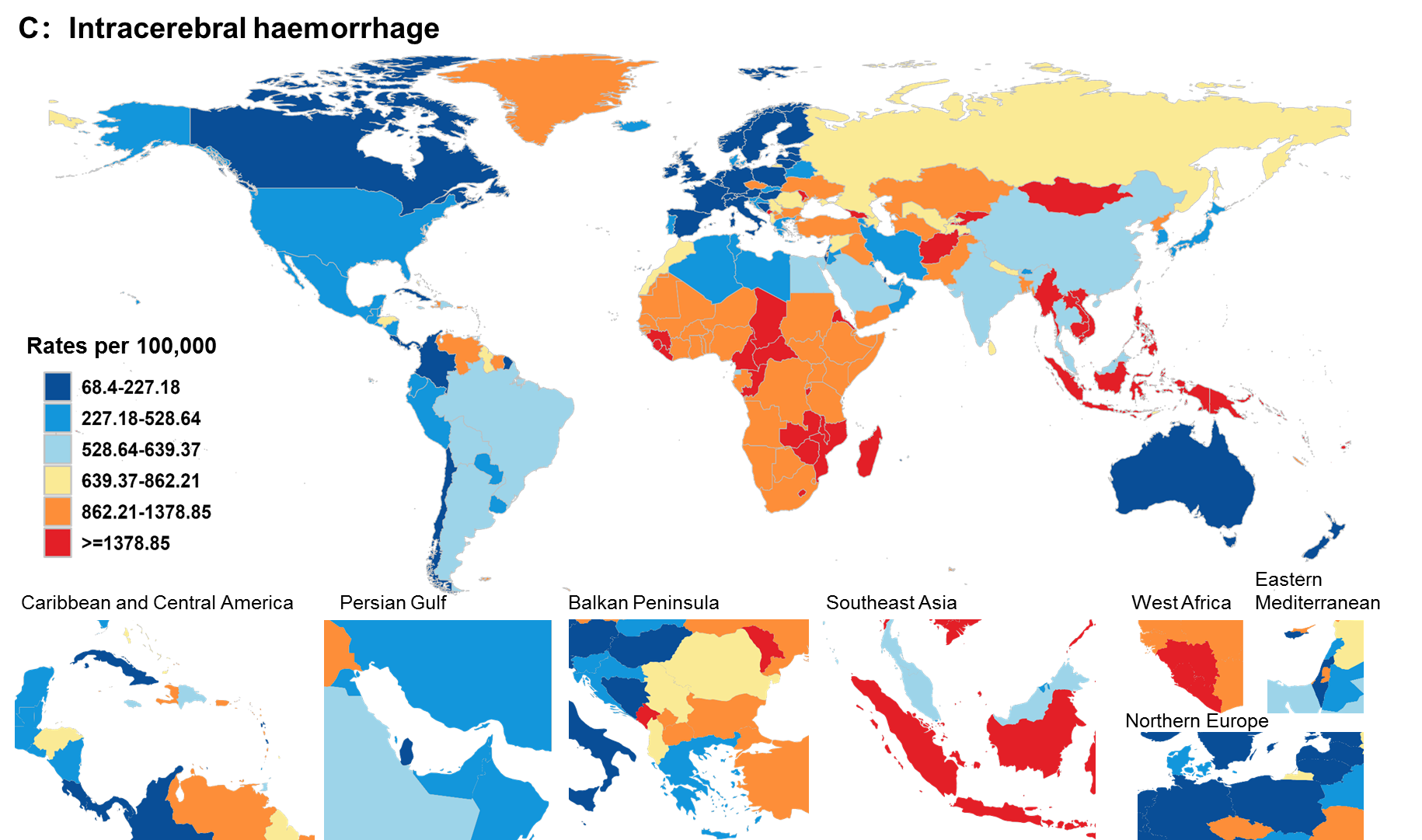

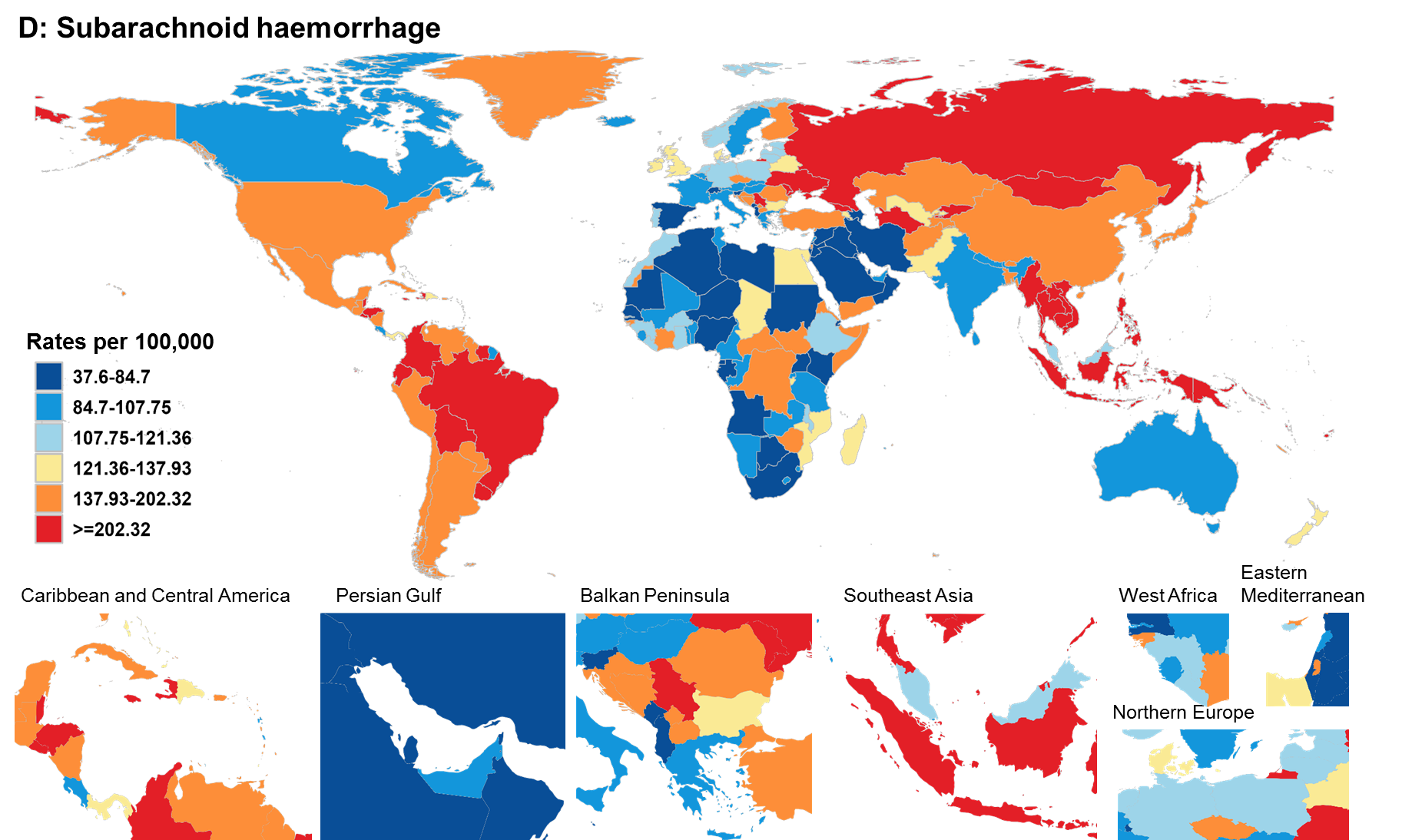


**Fig.S4 Age-standardised DALYs rates per 100 000 people by stroke type and country, for both sexes, 2050.**

**Table S2. Incident cases, prevalent cases, deaths, and DALYs for stroke in 2050 and percentage change in age-standardised rates for 2021–2050, by location, for both sexes**

| **Location** | **Disease** | **Prevalent cases** | | **Incident cases** | | **Deaths** | | **DALY** | |
| --- | --- | --- | --- | --- | --- | --- | --- | --- | --- |
|  |  | **Absolute number**  **(95%UI)** | **Change of age-standardised prevalence rates (%)** | **Absolute number**  **(95%UI)** | **Change of age-standardised incidence rates (%)** | **Absolute number**  **(95%UI)** | **Change of age-standardised deaths rates (%)** | **Absolute number**  **(95%UI)** | **Change of age-standardised DALY rates (%)** |
| Afghanistan | All stroke | 429731  (402546, 461624) | 21.07  (13.42, 30.06) | 49420  (44999, 54717) | 23.66  (12.6, 36.91) | 27729  (23705, 34060) | 2.14  (-12.69, 25.46) | 1153732  (1021458, 1280284) | 30.17  (15.25, 44.45) |
| Afghanistan | Intracerebral hemorrhage | 91339  (84845, 99249) | 31.3  (22.29, 41.77) | 18361  (16738, 20546) | 51.68  (40.27, 66.86) | 14037  (12225, 16029) | -7.1  (-20.24, 9.32) | 617386  (562090, 673738) | 28.74  (21.68, 40.05) |
| Afghanistan | Ischemic stroke | 277828  (257405, 296425) | 13.75  (5.51, 20.87) | 28366  (25630, 31362) | 12.12  (4.37, 21.14) | 12220  (10087, 16436) | -23.93  (-39.29, 6.37) | 467939  (396601, 529185) | 16.93  (0.01, 30.6) |
| Afghanistan | Subarachnoid hemorrhage | 39986  (36820, 42425) | 41.82  (31.73, 48.62) | 2693  (2631, 2809) | -1.85  (-4.72, 3.04) | 1472  (1393, 1596) | -2.88  (-7.59, 7.07) | 68407  (62768, 77360) | 36.08  (25.19, 48.39) |
| Albania | All stroke | 42289  (36286, 48631) | 52.9  (31.2, 75.83) | 3632  (2989, 4444) | 18.57  (-2.42, 45.07) | 1587  (1218, 2128) | -24.7  (-42.23, 0.96) | 45449  (32445, 56686) | -6.5  (-33.25, 16.62) |
| Albania | Intracerebral hemorrhage | 4960  (4121, 5780) | 6.84  (-13.19, 27.81) | 1360  (1118, 1599) | -10.58  (-31.44, 15.94) | 931  (699, 1338) | -50.62  (-61.65, -33.47) | 31937  (25522, 38074) | -28.7  (-48.68, -5.03) |
| Albania | Ischemic stroke | 22131  (18644, 27917) | -11.24  (-34.83, 22.67) | 2107  (1712, 2675) | 9.46  (-26.31, 43.98) | 585  (451, 715) | -21.08  (-39.55, -3.91) | 11894  (5886, 16430) | -14.79  (-65.71, 49.77) |
| Albania | Subarachnoid hemorrhage | 2347  (1958, 2876) | 3.75  (-17.35, 30.03) | 165  (159, 169) | -2.36  (-4.72, -0.16) | 71  (68, 75) | 5.05  (-0.51, 12.12) | 1619  (1037, 2183) | -19.3  (-48.5, 15.24) |
| Algeria | All stroke | 952005  (915010, 975623) | 38.4  (33.02, 41.83) | 87969  (83317, 92616) | 40.25  (32.84, 47.66) | 29810  (26744, 33060) | 44.41  (29.55, 60.16) | 919627  (820828, 1029969) | 45.17  (29.57, 62.59) |
| Algeria | Intracerebral hemorrhage | 97013  (91614, 104183) | 3.86  (-2.56, 10.13) | 16609  (15123, 17991) | 14.32  (2.07, 26.09) | 10583  (9805, 11502) | -9.03  (-16.17, -2.59) | 325944  (289312, 369434) | -1.22  (-14.36, 12.07) |
| Algeria | Ischemic stroke | 644008  (617159, 672071) | -9.1  (-13.01, -5.86) | 67776  (64744, 70951) | -10.38  (-15.76, -6.29) | 18025  (15838, 20292) | -21.28  (-30.48, -12.92) | 543584  (486818, 602911) | -16.37  (-27.13, -4.98) |
| Algeria | Subarachnoid hemorrhage | 55517  (53411, 57662) | 1.87  (-1.63, 6.6) | 3584  (3450, 3674) | -4.99  (-7.26, -2.99) | 1202  (1101, 1266) | -5.5  (-12.5, -1.5) | 50099  (44698, 57624) | 9.36  (-4.37, 24.48) |
| Angola | All stroke | 572657  (533583, 616145) | 21.45  (13.16, 30.67) | 56365  (52126, 60051) | 19.09  (10.14, 26.89) | 23917  (21184, 26555) | -1.86  (-13.09, 8.96) | 646765  (524455, 756417) | -12.93  (-29.4, 1.83) |
| Angola | Intracerebral hemorrhage | 96777  (90636, 104938) | 6.84  (1.01, 15.63) | 16120  (14566, 17523) | -7.98  (-15.05, -0.91) | 15347  (13316, 17204) | -17.9  (-29.99, -8.08) | 389151  (320745, 452976) | -31.22  (-41.77, -21.95) |
| Angola | Ischemic stroke | 388008  (363640, 415544) | 9.41  (3.63, 15.82) | 37730  (35100, 39954) | 15.1  (7.81, 21.18) | 7769  (7117, 8508) | -12.01  (-19.59, -5.56) | 224787  (176936, 263644) | -9.05  (-20.84, 4.98) |
| Angola | Subarachnoid hemorrhage | 48768  (44439, 53696) | 36.89  (26.02, 48.73) | 2514  (2460, 2574) | -2.3  (-4.28, 0.16) | 802  (752, 843) | -8.94  (-15.32, -3.83) | 32827  (26774, 39796) | -2.34  (-18.11, 15.21) |
| Argentina | All stroke | 768570  (735569, 795144) | 21.16  (15.96, 25.35) | 58320  (53612, 62911) | 18.4  (8.84, 27.73) | 16705  (14109, 19355) | -3.36  (-18.39, 11.97) | 593102  (489541, 686265) | 15.63  (-4.56, 33.79) |
| Argentina | Intracerebral hemorrhage | 120210  (113779, 126458) | -2.47  (-8.55, 3.28) | 13929  (12565, 15241) | -16.88  (-27.6, -6.19) | 9150  (7620, 10231) | -23.84  (-35.57, -15) | 307087  (272034, 331232) | -1.29  (-16.13, 8.38) |
| Argentina | Ischemic stroke | 448346  (422915, 476237) | 1.78  (-4.95, 9.25) | 38302  (35236, 41414) | 1.75  (-9.22, 10.24) | 5587  (4685, 6952) | -31.43  (-43.3, -15.31) | 199574  (138366, 263794) | -15.96  (-50.33, 22.09) |
| Argentina | Subarachnoid hemorrhage | 85556  (83337, 88511) | 2.83  (-0.38, 6.61) | 6088  (5811, 6256) | -2.97  (-6.84, -0.67) | 1968  (1804, 2172) | -23.17  (-28.81, -17.95) | 86441  (79140, 91239) | -1.74  (-10.17, 4.24) |
| Armenia | All stroke | 57227  (51539, 64655) | 45.08  (30.66, 63.9) | 4695  (3976, 5477) | 42.88  (21, 66.68) | 958  (762, 1164) | -28.22  (-42.94, -12.83) | 31554  (21328, 45499) | -17.73  (-44.39, 18.63) |
| Armenia | Intracerebral hemorrhage | 3640  (2843, 4715) | -0.32  (-25.09, 33.95) | 837  (569, 1084) | 32.82  (-20.83, 78.06) | 335  (272, 409) | -30.32  (-45.29, -17.05) | 11881  (8228, 18872) | -11.12  (-50.31, 46.22) |
| Armenia | Ischemic stroke | 35568  (31722, 40548) | 0.74  (-13.37, 20.88) | 3669  (3224, 4199) | 16.64  (-2.61, 41.9) | 543  (415, 669) | -57.6  (-67.43, -49.13) | 16272  (10344, 22764) | -51  (-73.05, -28.47) |
| Armenia | Subarachnoid hemorrhage | 2910  (2485, 3469) | 3.24  (-12.27, 24.75) | 189  (183, 193) | -2.99  (-6.09, -0.5) | 80  (75, 85) | -1.87  (-10.45, 2.99) | 3401  (2755, 3863) | 21.75  (-2.39, 42.92) |
| Australia | All stroke | 525030  (495928, 551273) | 33.18  (25.8, 39.84) | 28906  (26509, 31332) | 23.83  (13.56, 34.22) | 5180  (4871, 5667) | 35.1  (27.06, 47.85) | 173477  (130067, 221314) | 32.7  (-0.51, 69.29) |
| Australia | Intracerebral hemorrhage | 48456  (42558, 55351) | 12.23  (-0.43, 26.44) | 5006  (4250, 5657) | 6.48  (-19.86, 33.54) | 2155  (1985, 2319) | -4.64  (-12.14, 3.97) | 61926  (44896, 80164) | 21.87  (-18.33, 72.65) |
| Australia | Ischemic stroke | 298855  (277594, 320927) | 10.07  (1.77, 19.66) | 21579  (19984, 23305) | -3.53  (-13.12, 5.21) | 2122  (2016, 2392) | 20.5  (13.56, 33.75) | 77099  (53699, 102801) | 13.33  (-24.12, 65.73) |
| Australia | Subarachnoid hemorrhage | 40859  (38349, 45065) | 4.64  (-0.34, 12.47) | 2321  (2275, 2371) | 1.51  (-0.69, 3.43) | 903  (871, 956) | 2.64  (-0.88, 7.05) | 34452  (31472, 38349) | 7.48  (-3.13, 21.22) |
| Austria | All stroke | 205809  (189717, 219447) | -1.78  (-9.46, 4.72) | 11850  (10790, 12750) | 11.68  (1.69, 20.17) | 1940  (1823, 2044) | 27.37  (19.73, 34.24) | 58092  (49917, 67391) | 11.72  (-4, 29.61) |
| Austria | Intracerebral hemorrhage | 18796  (17596, 19846) | -3.04  (-10.17, 4.51) | 2351  (2179, 2485) | 15.34  (2.36, 28.58) | 802  (744, 849) | -0.82  (-8.02, 5.14) | 20485  (17152, 24076) | 8.9  (-17.5, 35.15) |
| Austria | Ischemic stroke | 103516  (95051, 110550) | -22.14  (-30.12, -15.86) | 8471  (7626, 9207) | -23.45  (-35.8, -10.83) | 869  (819, 921) | -12.69  (-18.08, -7.12) | 28231  (24379, 33261) | -15.86  (-37.05, 11.85) |
| Austria | Subarachnoid hemorrhage | 21104  (19654, 22317) | -8.18  (-14.19, -3.68) | 1028  (986, 1059) | -4.64  (-8.86, -1.74) | 270  (260, 275) | -4.85  (-7.49, -3.08) | 9375  (8386, 10054) | -5.33  (-18.28, 2.7) |
| Azerbaijan | All stroke | 186615  (177138, 196019) | 48.78  (41.23, 56.28) | 17310  (15700, 19310) | 19.5  (8.38, 33.3) | 5356  (4529, 6384) | -11.03  (-24.75, 6.06) | 200178  (165502, 236696) | 13.59  (-6.09, 34.31) |
| Azerbaijan | Intracerebral hemorrhage | 23590  (22255, 24918) | -11.23  (-16.3, -5.41) | 5311  (4605, 6155) | -36.78  (-45.34, -25.5) | 2631  (2331, 3006) | -56.66  (-62.02, -50.34) | 104970  (90225, 122557) | -35.55  (-45.11, -26.64) |
| Azerbaijan | Ischemic stroke | 114956  (108583, 121723) | -5.5  (-11.58, 2.32) | 11333  (10442, 12473) | -7.48  (-16.68, 2.4) | 2513  (1998, 3152) | -23.03  (-38.98, -6.35) | 86195  (67967, 103745) | -9.39  (-30.47, 11.86) |
| Azerbaijan | Subarachnoid hemorrhage | 11040  (10081, 12478) | 9.04  (0.94, 19.23) | 665  (653, 682) | -2.3  (-4.17, -0.43) | 212  (200, 226) | 36.75  (29.06, 47.86) | 9013  (7311, 10394) | 55.32  (21.57, 81.9) |
| Bahamas | All stroke | 5089  (4697, 5561) | 19.44  (10.24, 30.51) | 381  (321, 440) | 2.2  (-13.79, 18.13) | 140  (122, 164) | -12.41  (-23.38, 2.8) | 4878  (4077, 5779) | 0.47  (-16.03, 19.02) |
| Bahamas | Intracerebral hemorrhage | 829  (780, 869) | -7.6  (-12.37, -2.26) | 125  (112, 141) | -28.57  (-39.14, -16.47) | 77  (67, 91) | -39.63  (-48.55, -30.62) | 3088  (2752, 3487) | -17.16  (-26.6, -7.22) |
| Bahamas | Ischemic stroke | 2928  (2615, 3273) | -15.76  (-26.98, -4.21) | 216  (170, 257) | -31.55  (-51.05, -13.76) | 47  (40, 56) | -44.58  (-53.37, -33.59) | 1226  (796, 1688) | -44.97  (-65.89, -17.19) |
| Bahamas | Subarachnoid hemorrhage | 688  (647, 733) | 1.19  (-6.12, 8.93) | 40  (39, 42) | -4.8  (-7.49, -2) | 16  (15, 17) | -9.02  (-13.53, -6.1) | 565  (529, 605) | -11.57  (-18.19, -5.17) |
| Bahrain | All stroke | 24876  (22074, 27637) | 104.37  (81.35, 127.05) | 1848  (1585, 2140) | 137.54  (103.73, 174.98) | 342  (310, 380) | 11.93  (1.5, 24.21) | 14667  (11065, 18447) | 37.98  (4.1, 73.53) |
| Bahrain | Intracerebral hemorrhage | 3026  (2807, 3265) | -5.92  (-12.88, 1.67) | 330  (281, 385) | -0.58  (-18.87, 22.31) | 169  (152, 189) | -43.42  (-50.58, -36.34) | 6476  (4916, 8245) | -9  (-33.85, 14.54) |
| Bahrain | Ischemic stroke | 16128  (14803, 18101) | 24.33  (12.87, 41.57) | 1435  (1223, 1670) | 52.07  (22.17, 83.92) | 145  (131, 161) | -69.41  (-72.59, -65.47) | 6894  (4979, 8792) | -47.42  (-65.49, -27.97) |
| Bahrain | Subarachnoid hemorrhage | 1813  (1601, 2085) | 30.87  (14.59, 53.97) | 83  (82, 85) | 3.71  (1.86, 5.36) | 28  (27, 30) | -6.16  (-8.9, -1.37) | 1298  (1170, 1410) | 24.31  (8.51, 36.49) |
| Bangladesh | All stroke | 2690105  (2549050, 2860648) | 42.73  (35.24, 51.77) | 297832  (275833, 322735) | 28.67  (19.17, 39.43) | 174193  (146456, 199970) | 14.83  (-3.45, 31.83) | 4847813  (4215292, 5492644) | 13.27  (-1.5, 28.34) |
| Bangladesh | Intracerebral hemorrhage | 503636  (474588, 531190) | -20.41  (-25, -15.67) | 93426  (85527, 104159) | -38.16  (-44.34, -30.33) | 83418  (66751, 98347) | -44.45  (-53.57, -35.5) | 2570486  (2241622, 2944417) | -33.36  (-42.78, -23.31) |
| Bangladesh | Ischemic stroke | 1654470  (1555657, 1759154) | 9.73  (2.77, 17.56) | 180324  (167564, 192920) | 9.37  (0.21, 17.31) | 78520  (69486, 87395) | -18.03  (-27.61, -9.65) | 1892943  (1641095, 2113669) | -17.03  (-30.84, -4.43) |
| Bangladesh | Subarachnoid hemorrhage | 262145  (244996, 280573) | -8.09  (-14.49, -0.79) | 24081  (22741, 25655) | -14.51  (-20.6, -7.39) | 12255  (10220, 14227) | -30.98  (-42.11, -21.89) | 384384  (332575, 434559) | -31.13  (-41.38, -19.87) |
| Barbados | All stroke | 4500  (4081, 5071) | 12.93  (2.41, 27.25) | 309  (246, 364) | -7.71  (-26.49, 8.83) | 161  (137, 185) | -13.76  (-26.71, -1.24) | 4630  (3587, 5962) | -3.09  (-24.91, 24.79) |
| Barbados | Intracerebral hemorrhage | 464  (425, 506) | -19.83  (-28.24, -10.13) | 79  (63, 91) | -28.69  (-50.98, -5.96) | 73  (63, 84) | -26.03  (-34.61, -14.4) | 2109  (1715, 2673) | 3.4  (-24.02, 36.53) |
| Barbados | Ischemic stroke | 2558  (2192, 3020) | -13.96  (-33.04, 8.8) | 195  (149, 237) | -41.17  (-63.03, -18.62) | 69  (57, 81) | -36.1  (-46.04, -23.72) | 2014  (1393, 2741) | -16.1  (-48.61, 37.89) |
| Barbados | Subarachnoid hemorrhage | 486  (462, 509) | -19.69  (-24.81, -15.02) | 35  (33, 36) | -6.04  (-10.16, -3.34) | 19  (18, 20) | -9.86  (-15.25, -6.93) | 507  (479, 548) | -18.64  (-25.32, -11.3) |
| Belarus | All stroke | 180172  (172641, 187998) | 10.79  (6.16, 15.61) | 14836  (13695, 16160) | -14.36  (-20.95, -6.72) | 4049  (3326, 5075) | -45.34  (-55.1, -31.49) | 140412  (114103, 170076) | -33.37  (-45.85, -19.29) |
| Belarus | Intracerebral hemorrhage | 12334  (11336, 13472) | -27.51  (-35.93, -19.75) | 1938  (1549, 2393) | -46.55  (-59.98, -34.87) | 1546  (1344, 1841) | -46.14  (-55.52, -36.8) | 41681  (30794, 53977) | -36.01  (-53.15, -18.94) |
| Belarus | Ischemic stroke | 123447  (116212, 128292) | -16.58  (-22.16, -9.8) | 12236  (11506, 13087) | -28.34  (-34.06, -22.37) | 2135  (1641, 2837) | -66.87  (-73.32, -58.41) | 87634  (73150, 103941) | -48.23  (-62.24, -36.52) |
| Belarus | Subarachnoid hemorrhage | 8394  (7659, 9282) | -5.68  (-15.19, 2.95) | 662  (641, 680) | -6.51  (-10.67, -3.63) | 368  (341, 397) | -18.5  (-26.02, -11.18) | 11097  (10159, 12157) | -28.63  (-35.73, -20.52) |
| Belgium | All stroke | 185609  (176271, 195391) | 17.06  (11.18, 23.23) | 11686  (10887, 12443) | 17.47  (9.44, 25.08) | 2691  (2493, 2864) | 15.47  (7.03, 22.93) | 91774  (82734, 103858) | 38.25  (24.63, 56.45) |
| Belgium | Intracerebral hemorrhage | 15796  (14678, 16828) | 3.79  (-4.05, 9.99) | 2134  (1902, 2364) | 4.15  (-12.53, 20.56) | 1133  (1012, 1227) | -11.64  (-22.96, -2.83) | 34659  (31210, 40038) | 25.88  (5.55, 46.54) |
| Belgium | Ischemic stroke | 101313  (94583, 106464) | -1.32  (-8.47, 5.91) | 8730  (8175, 9243) | -7.72  (-15.87, -0.1) | 1184  (1114, 1256) | -4.63  (-10.74, 2.04) | 45023  (39995, 51021) | 27.45  (3.17, 54.85) |
| Belgium | Subarachnoid hemorrhage | 12975  (12191, 13749) | 4.79  (-0.84, 10.91) | 822  (810, 836) | -2.37  (-3.63, -0.7) | 374  (367, 381) | 8.07  (5.38, 9.87) | 12092  (11529, 12799) | 15.67  (8.35, 22.85) |
| Belize | All stroke | 6303  (5586, 7336) | 71.34  (51.83, 99.39) | 411  (347, 493) | 29.61  (9.41, 55.52) | 221  (178, 324) | 61.48  (30.12, 136.63) | 5265  (3147, 8010) | 25.46  (-25.02, 90.86) |
| Belize | Intracerebral hemorrhage | 866  (736, 1239) | 7.67  (-8.74, 57.7) | 127  (100, 166) | -22.3  (-42.83, 6.74) | 126  (95, 215) | -10.67  (-33.14, 48.27) | 2527  (1468, 3765) | -44.83  (-68.01, -9.89) |
| Belize | Ischemic stroke | 3377  (2836, 4163) | 14.24  (-8.69, 44.78) | 239  (203, 281) | -14.64  (-32.5, 4.73) | 79  (68, 93) | 4.02  (-11, 21.85) | 1694  (963, 2681) | -12.42  (-57.11, 42.03) |
| Belize | Subarachnoid hemorrhage | 746  (697, 811) | 9.32  (1.3, 20.5) | 45  (44, 46) | -0.1  (-2.07, 1.56) | 16  (15, 17) | 6.73  (2.36, 13.13) | 1044  (715, 1564) | 98.54  (32, 204) |
| Benin | All stroke | 222401  (209784, 235441) | 18.25  (11.54, 25.18) | 18861  (17329, 20450) | 12.32  (3.2, 21.79) | 10938  (9674, 12021) | 7.04  (-5.33, 17.64) | 283113  (231477, 331552) | -4.74  (-22.11, 11.56) |
| Benin | Intracerebral hemorrhage | 39876  (37568, 42394) | -14.92  (-19.33, -9.33) | 6131  (5497, 6652) | -22.08  (-28.51, -15.04) | 6195  (5216, 6993) | -15.07  (-28.7, -3.66) | 145859  (115423, 171824) | -29.62  (-43.33, -18.74) |
| Benin | Ischemic stroke | 139675  (129067, 149980) | 9.37  (1.78, 16.02) | 11943  (11057, 12987) | 19.32  (11.53, 28.18) | 4426  (4155, 4690) | -7.25  (-13.29, -2.15) | 123287  (105054, 142656) | -1.12  (-12.14, 11.88) |
| Benin | Subarachnoid hemorrhage | 16073  (14536, 17713) | 20.73  (10.1, 31.41) | 788  (775, 810) | 0.59  (-0.99, 3.16) | 317  (303, 338) | -0.44  (-4.8, 5.24) | 13967  (11000, 17072) | 10.85  (-11.48, 31.85) |
| Bhutan | All stroke | 11050  (9967, 12343) | 78.55  (61.04, 99.44) | 956  (844, 1084) | 48.22  (30.85, 68.09) | 482  (415, 552) | 46.16  (25.68, 67.3) | 14228  (11324, 17766) | 56.34  (24.43, 95.21) |
| Bhutan | Intracerebral hemorrhage | 1810  (1685, 1927) | 4.68  (-4.26, 13.14) | 210  (174, 258) | -46.63  (-62.06, -29.11) | 195  (167, 232) | -37.23  (-47.27, -26.67) | 5287  (3721, 7118) | -35.09  (-57.47, -10.6) |
| Bhutan | Ischemic stroke | 6928  (5984, 7769) | 12.42  (-10.18, 33.62) | 679  (606, 756) | -0.86  (-16.81, 14.97) | 259  (223, 287) | -11.77  (-23.68, -2.88) | 7680  (6456, 9221) | 4.75  (-18.42, 36.22) |
| Bhutan | Subarachnoid hemorrhage | 993  (934, 1053) | 24.04  (15.8, 33.37) | 68  (64, 70) | -4.62  (-8.99, -1.94) | 28  (24, 32) | -25.92  (-34.29, -16.23) | 1261  (1146, 1427) | 11.39  (-1.25, 28.27) |
| Bolivia  (Plurinational State of) | All stroke | 165933  (153152, 179486) | 47.79  (36.41, 59.86) | 12650  (11435, 14009) | 29.07  (16.66, 42.94) | 5888  (5314, 6605) | -3.48  (-12.88, 8.28) | 188369  (152956, 227144) | 7.04  (-13.08, 29.07) |
| Bolivia  (Plurinational State of) | Intracerebral hemorrhage | 20788  (18620, 23890) | 10.87  (-1.61, 26.96) | 2466  (2009, 2964) | -28.01  (-41.31, -12.71) | 2228  (1983, 2582) | -47.6  (-53.58, -38.98) | 72468  (56727, 91131) | -34.79  (-49.09, -17.63) |
| Bolivia  (Plurinational State of) | Ischemic stroke | 98287  (86955, 108756) | 28.38  (13.48, 42.68) | 7944  (7236, 8758) | 17.88  (6.69, 30.29) | 2104  (1843, 2379) | -23.03  (-33.38, -13.6) | 71746  (56259, 88433) | 11.98  (-12.02, 40.27) |
| Bolivia  (Plurinational State of) | Subarachnoid hemorrhage | 25273  (24139, 26434) | -13.07  (-16.83, -8.59) | 2240  (2190, 2287) | -1.58  (-3.86, 0.47) | 1556  (1488, 1644) | -1.15  (-5.32, 4.26) | 44155  (39970, 47581) | -10.2  (-18.28, -3.07) |
| Bosnia and Herzegovina | All stroke | 66574  (62273, 71732) | 10.15  (3.03, 18.68) | 6002  (5213, 6867) | 2.94  (-10.59, 17.78) | 1776  (1475, 2202) | -42.07  (-51.88, -28.18) | 64041  (53869, 79929) | -15.4  (-28.83, 5.59) |
| Bosnia and Herzegovina | Intracerebral hemorrhage | 2388  (1790, 3087) | -40.13  (-62.29, -13.36) | 464  (252, 799) | -24.1  (-65.01, 35.05) | 558  (451, 651) | -27.91  (-39.83, -14.13) | 6420  (3303, 11961) | -53.95  (-82.17, -4.48) |
| Bosnia and Herzegovina | Ischemic stroke | 46143  (41939, 50066) | -29.05  (-38.85, -17.58) | 5279  (4721, 5805) | -13.19  (-24.64, 0.58) | 1076  (895, 1404) | -63.58  (-69.21, -53.97) | 52669  (46123, 62445) | -23.81  (-41.12, 0.35) |
| Bosnia and Herzegovina | Subarachnoid hemorrhage | 2790  (2468, 3069) | -17.62  (-26.76, -6.75) | 259  (241, 263) | -2.77  (-5.89, -1.04) | 142  (129, 146) | -9.13  (-15.46, -5.62) | 4953  (4443, 5523) | 5.91  (-6.87, 20.37) |
| Botswana | All stroke | 54438  (50028, 59008) | 40.7  (29.31, 52.52) | 4287  (3865, 4683) | 3.34  (-6.83, 12.88) | 1239  (1050, 1492) | -12.45  (-25.82, 5.39) | 59071  (47688, 68850) | 41.38  (14.14, 64.79) |
| Botswana | Intracerebral hemorrhage | 6763  (6384, 7135) | -4.36  (-9.95, 0.86) | 1009  (899, 1100) | -41.64  (-48.32, -36.09) | 637  (539, 787) | -54.43  (-61.65, -44.06) | 29863  (23997, 35092) | -20.44  (-36.35, -6.48) |
| Botswana | Ischemic stroke | 37128  (34486, 40957) | -5.6  (-12.15, 4.56) | 3103  (2804, 3399) | -29.82  (-36.37, -22.61) | 560  (472, 659) | -46.69  (-55.13, -37.12) | 27064  (21954, 31144) | -2.83  (-21.14, 13.46) |
| Botswana | Subarachnoid hemorrhage | 3293  (2966, 3589) | 23.2  (10.67, 34.54) | 175  (162, 183) | -6.88  (-13.3, -2.75) | 42  (39, 45) | 2.92  (-3.65, 12.41) | 2144  (1736, 2614) | 36.37  (11.55, 67.98) |
| Brazil | All stroke | 3510474  (3397768, 3623841) | 52.39  (47.49, 57.31) | 310735  (288438, 331362) | 44.26  (33.91, 53.84) | 125207  (112532, 138751) | 34.77  (21.13, 49.34) | 3854702  (3316213, 4429090) | 44.59  (24.39, 66.13) |
| Brazil | Intracerebral hemorrhage | 384637  (362508, 407591) | 8.87  (3.19, 18.47) | 60346  (53449, 68338) | -9.49  (-24.91, 5.41) | 52866  (48020, 57892) | -11.38  (-19.81, -3.45) | 1513341  (1262996, 1723016) | -8.5  (-24.34, 7.02) |
| Brazil | Ischemic stroke | 2250203  (2171117, 2334884) | 5.3  (0.35, 9.08) | 221735  (207243, 233160) | 1.2  (-5.87, 6.42) | 56253  (49512, 63734) | -18.8  (-28.3, -8.09) | 1784290  (1522095, 2114935) | 9.5  (-10.35, 32.37) |
| Brazil | Subarachnoid hemorrhage | 351191  (340562, 361534) | 5.56  (2.47, 9.45) | 28654  (27746, 29864) | 2.16  (-0.88, 5.75) | 16089  (14999, 17125) | -3.69  (-9.37, 2.46) | 557071  (531121, 591139) | 6.14  (1.18, 12.07) |
| Brunei Darussalam | All stroke | 7639  (6827, 8418) | 27.06  (13.56, 40.02) | 537  (461, 623) | -1.31  (-15.18, 14.52) | 134  (116, 161) | -12.11  (-24.31, 5.58) | 5500  (4340, 7021) | 1.74  (-19.71, 29.87) |
| Brunei Darussalam | Intracerebral hemorrhage | 1331  (1173, 1458) | -26.27  (-36.03, -18.01) | 161  (138, 194) | -28.84  (-42.24, -12.18) | 60  (51, 74) | -55.79  (-62.82, -46.86) | 3073  (2526, 3874) | -27.18  (-39.68, -10.35) |
| Brunei Darussalam | Ischemic stroke | 4378  (3854, 4969) | -32.37  (-42.21, -20.61) | 284  (238, 334) | -53.91  (-63.79, -41.23) | 48  (42, 54) | -56.55  (-62.46, -51) | 1116  (662, 1707) | -63.83  (-81.27, -45.73) |
| Brunei Darussalam | Subarachnoid hemorrhage | 931  (866, 993) | -17.1  (-22.67, -11.96) | 92  (85, 96) | -5.93  (-11.8, -1.98) | 26  (22, 33) | -35.23  (-44.3, -25.52) | 1312  (1152, 1439) | -19.15  (-26.8, -11.65) |
| Bulgaria | All stroke | 121509  (115061, 129380) | 4.81  (-0.76, 11.59) | 9182  (8518, 9904) | -36.36  (-40.96, -31.35) | 3269  (2583, 4001) | -66.17  (-73.27, -58.59) | 152620  (128455, 173196) | -34.98  (-45.28, -26.22) |
| Bulgaria | Intracerebral hemorrhage | 10138  (9427, 11013) | -19.14  (-28.52, -10.83) | 2499  (2188, 2819) | -33.69  (-43.93, -23.01) | 1229  (1035, 1463) | -64.79  (-70.5, -57.97) | 61458  (50881, 70095) | -22.07  (-40.77, -7.96) |
| Bulgaria | Ischemic stroke | 74057  (68406, 79856) | -20.84  (-30.57, -10.14) | 6202  (5870, 6590) | -53.55  (-56.76, -48.88) | 1771  (1317, 2235) | -79.43  (-84.2, -74.39) | 84446  (71545, 95803) | -49.71  (-58.59, -39.26) |
| Bulgaria | Subarachnoid hemorrhage | 6346  (5781, 7030) | -0.62  (-11.33, 11.53) | 481  (460, 495) | -6.34  (-9.75, -3.8) | 269  (231, 303) | -27.47  (-34.87, -18.75) | 6716  (6029, 7298) | -43.65  (-49.77, -36) |
| Burkina Faso | All stroke | 318701  (299330, 340128) | 19.44  (12.18, 27.47) | 30318  (27888, 32731) | 25.56  (15.5, 35.55) | 17045  (15957, 18004) | 16.85  (9.39, 23.42) | 498024  (425902, 577677) | 14.05  (-2.46, 32.3) |
| Burkina Faso | Intracerebral hemorrhage | 70768  (66900, 74606) | 0.56  (-4.77, 5.32) | 11304  (10296, 12324) | -11.35  (-17.45, -4.34) | 10799  (10108, 11385) | -11.8  (-17.1, -6.15) | 283589  (244210, 335611) | -17.99  (-27.33, -4.98) |
| Burkina Faso | Ischemic stroke | 208652  (195636, 223042) | 10.66  (5.29, 16.88) | 17744  (16336, 19107) | 27.01  (18.24, 34.57) | 5734  (5353, 6081) | -5.94  (-12.54, -0.45) | 187353  (157482, 211145) | 11.12  (-1.73, 21.99) |
| Burkina Faso | Subarachnoid hemorrhage | 24665  (23652, 25914) | 9  (4.6, 13.69) | 1270  (1256, 1299) | 0.78  (-0.58, 2.92) | 512  (496, 538) | -0.83  (-4.56, 3.73) | 27082  (24210, 30921) | 29.25  (14.8, 44.87) |
| Burundi | All stroke | 200227  (190255, 214154) | 14.71  (9, 22.69) | 21480  (20275, 22633) | 12.99  (6.65, 19.05) | 13426  (12653, 14396) | 23.71  (16.58, 32.64) | 366245  (318609, 417830) | 8.27  (-5.81, 23.52) |
| Burundi | Intracerebral hemorrhage | 39105  (36846, 41356) | 0.76  (-4.6, 6.37) | 8516  (8078, 8958) | -6.81  (-10.96, -2.66) | 9548  (8993, 10313) | -10.14  (-15.33, -2.77) | 251335  (220143, 286535) | -18.43  (-27.37, -9.53) |
| Burundi | Ischemic stroke | 127876  (120198, 135560) | -7.77  (-12.32, -2.83) | 11663  (10934, 12342) | -8.49  (-13.4, -3.69) | 3385  (3184, 3576) | -3.39  (-9.13, 2.25) | 94983  (80248, 109451) | -2.23  (-14.09, 9.65) |
| Burundi | Subarachnoid hemorrhage | 15729  (15111, 16423) | -10.37  (-13.82, -7.06) | 1301  (1263, 1333) | -3.22  (-6.02, -0.75) | 493  (476, 507) | -5.47  (-8.71, -1.99) | 19927  (18217, 21845) | -4.29  (-11.76, 4.16) |
| Cabo Verde | All stroke | 15279  (14578, 16236) | 67.83  (60.14, 78.35) | 1242  (1139, 1361) | 91.37  (75.51, 109.67) | 390  (331, 461) | 19.01  (1.1, 40.66) | 13122  (10283, 15800) | 31.81  (3.3, 58.71) |
| Cabo Verde | Intracerebral hemorrhage | 2002  (1901, 2135) | -16.22  (-21.6, -10.49) | 365  (326, 407) | -5.11  (-18.17, 7.26) | 188  (158, 233) | -34.71  (-45.07, -20.25) | 6813  (5199, 8242) | -26.38  (-52.49, -6.68) |
| Cabo Verde | Ischemic stroke | 9539  (8919, 10233) | 13.76  (2.93, 23.87) | 840  (777, 916) | 24.91  (14.25, 38.24) | 189  (161, 214) | -49.12  (-56.44, -41.68) | 5711  (4589, 6842) | -51.99  (-67.27, -36.7) |
| Cabo Verde | Subarachnoid hemorrhage | 902  (836, 967) | 23.77  (13.07, 34.76) | 37  (36, 38) | 0.18  (-1.81, 2.71) | 13  (13, 14) | 1.16  (-3.49, 6.4) | 597  (495, 716) | 9.46  (-14.9, 37.48) |
| Cambodia | All stroke | 315096  (302943, 325937) | 66.2  (59.79, 71.92) | 37265  (35400, 39459) | 39.52  (32.54, 47.73) | 23143  (21140, 25284) | 27.29  (16.27, 39.06) | 670082  (603978, 726674) | 30.36  (17.5, 41.37) |
| Cambodia | Intracerebral hemorrhage | 59944  (57246, 62244) | 8.03  (2.28, 12.65) | 13851  (13036, 14772) | -18.25  (-23.57, -12.73) | 14755  (13256, 16364) | -24.12  (-31.16, -16.45) | 409159  (364374, 446976) | -19.37  (-28.48, -10.27) |
| Cambodia | Ischemic stroke | 200803  (193145, 212299) | 28.78  (23.4, 36.36) | 21416  (20406, 22646) | 19.2  (12.08, 27.91) | 7356  (6883, 7851) | -9.22  (-14.86, -3.76) | 223751  (205448, 238680) | 11.65  (-0.56, 21.18) |
| Cambodia | Subarachnoid hemorrhage | 22655  (21362, 24112) | 0.16  (-5.87, 7.12) | 1998  (1957, 2040) | -3  (-4.98, -0.86) | 1033  (1001, 1069) | -1.51  (-4.52, 2.82) | 37172  (34155, 41018) | 8.98  (-1.64, 19.76) |
| Cameroon | All stroke | 454606  (433691, 475007) | -4.06  (-8.47, 0.25) | 39958  (37343, 42587) | -8.72  (-14.69, -2.71) | 25900  (23215, 28428) | -3.38  (-13.4, 6.05) | 751190  (667391, 832657) | -10.13  (-20.16, -0.39) |
| Cameroon | Intracerebral hemorrhage | 97452  (93267, 103592) | -25.22  (-28.29, -20.86) | 14047  (13108, 15023) | -38.18  (-41.81, -34.46) | 14394  (12824, 16040) | -33.45  (-41.04, -26.28) | 449130  (396122, 490902) | -32.63  (-39.74, -26.99) |
| Cameroon | Ischemic stroke | 295897  (279178, 310329) | -13.24  (-17.54, -9.41) | 24026  (22403, 25640) | -7.81  (-13.14, -2.56) | 10775  (9730, 11587) | -11.5  (-19.97, -4.34) | 274082  (246918, 309515) | -17.46  (-23.63, -9.59) |
| Cameroon | Subarachnoid hemorrhage | 29475  (27316, 31490) | -11.38  (-17.3, -5.42) | 1885  (1831, 1924) | -2.14  (-5.1, -0.16) | 730  (661, 801) | -22.6  (-30.34, -16.1) | 27978  (24352, 32239) | -25.35  (-34.2, -14.88) |
| Canada | All stroke | 995221  (940404, 1039678) | 15.56  (9.19, 20.72) | 51027  (47840, 54860) | 10.35  (3.46, 18.64) | 7972  (7505, 8574) | 28.14  (20.65, 37.87) | 307160  (270393, 345217) | 28.45  (13.07, 44.36) |
| Canada | Intracerebral hemorrhage | 109450  (105353, 113183) | 8.14  (2.44, 13.1) | 9134  (8579, 9882) | -2.22  (-13.46, 10.55) | 3425  (3200, 3659) | 3.26  (-3.91, 9.13) | 107302  (92719, 122436) | 15.15  (-5.13, 38.21) |
| Canada | Ischemic stroke | 590584  (547054, 622942) | -1.22  (-8.74, 4.52) | 38249  (35749, 41253) | -10.65  (-18.88, -2.92) | 3330  (3171, 3636) | 15.76  (8.88, 26.36) | 150094  (130635, 170475) | 14.71  (-4.84, 37.34) |
| Canada | Subarachnoid hemorrhage | 63644  (61557, 65670) | -4.28  (-7.5, -1.09) | 3643  (3513, 3725) | -7.52  (-10.02, -5.01) | 1217  (1135, 1279) | -8.44  (-13.92, -3.8) | 49764  (47038, 52307) | 0.47  (-4.83, 6.73) |
| Central African Republic | All stroke | 77228  (71830, 83322) | -9.68  (-15.99, -2.55) | 7605  (7199, 8095) | -29.03  (-32.82, -24.47) | 5352  (4987, 5719) | -27.41  (-32.35, -22.43) | 180432  (159321, 203234) | -21.8  (-30.95, -11.92) |
| Central African Republic | Intracerebral hemorrhage | 19253  (18012, 20559) | -1.87  (-7.56, 3.59) | 3264  (3116, 3436) | -21.96  (-25.74, -19.08) | 3992  (3735, 4259) | -17.06  (-22.91, -10.68) | 131098  (117333, 147099) | -14.92  (-21.42, -7.78) |
| Central African Republic | Ischemic stroke | 49020  (45702, 51870) | 0  (-4.79, 4.08) | 3827  (3585, 4130) | -16.29  (-19.94, -12.41) | 1140  (1039, 1229) | -10.59  (-18.01, -3.27) | 40379  (33672, 46297) | 4.81  (-4.4, 15.22) |
| Central African Republic | Subarachnoid hemorrhage | 6070  (5827, 6358) | -1.2  (-4.78, 2.33) | 514  (499, 528) | -7.29  (-10.76, -4.6) | 221  (213, 231) | -9.38  (-12.29, -5.83) | 8955  (8316, 9839) | -12.66  (-17.43, -4.8) |
| Chad | All stroke | 258162  (247287, 270093) | -0.55  (-4.74, 4.05) | 26170  (24919, 27726) | 6.09  (1.02, 12.4) | 17279  (16367, 17879) | 8.01  (2.32, 11.77) | 453279  (410174, 502683) | -7  (-15.84, 3.14) |
| Chad | Intracerebral hemorrhage | 62390  (59776, 65070) | -10.03  (-13.64, -6.38) | 10841  (10423, 11437) | -14.28  (-17.9, -10.89) | 10500  (9905, 10891) | -6.4  (-12, -2.39) | 287080  (264988, 312633) | -18.73  (-23.71, -13.3) |
| Chad | Ischemic stroke | 166370  (158598, 173230) | -5.97  (-9.39, -2.59) | 14266  (13447, 15208) | 3.69  (-0.56, 8.85) | 6230  (5925, 6428) | -3.85  (-9.36, -1.02) | 141121  (122018, 162755) | -24.69  (-31.03, -18.03) |
| Chad | Subarachnoid hemorrhage | 18702  (18105, 19528) | 2.51  (-0.88, 6.1) | 1063  (1049, 1080) | 1.46  (0.18, 2.74) | 548  (537, 560) | -1.75  (-3.5, 0) | 25078  (23169, 27295) | 7.59  (0.57, 15.8) |
| Chile | All stroke | 382284  (363490, 401509) | 52.61  (45.11, 60.28) | 22277  (20311, 24074) | 20.42  (9.79, 30.13) | 5054  (4592, 5529) | -2.21  (-11.17, 6.99) | 127701  (101415, 152108) | -18.98  (-35.65, -3.49) |
| Chile | Intracerebral hemorrhage | 48268  (45756, 51030) | -16.56  (-22.12, -10.71) | 4343  (4074, 4663) | -39.28  (-46.13, -31.79) | 2157  (1998, 2340) | -38.15  (-44.22, -32.65) | 47988  (39733, 55183) | -48.19  (-60.66, -35.65) |
| Chile | Ischemic stroke | 200365  (191067, 211084) | -1.26  (-7.7, 6.97) | 15597  (13945, 17033) | -11.33  (-20.34, -0.39) | 1993  (1767, 2220) | -45.2  (-51.6, -39.34) | 49225  (32642, 64993) | -44.81  (-70.41, -21.46) |
| Chile | Subarachnoid hemorrhage | 35198  (33572, 36594) | 5.16  (0.2, 11.06) | 2337  (2292, 2378) | -2.51  (-3.89, -1.04) | 904  (826, 970) | -9.46  (-14.07, -5.12) | 30488  (29040, 31932) | -4.8  (-9.85, 0.62) |
| China | All stroke | 35755119  (33532706, 37387023) | 43.65  (34.72, 50.2) | 2523807  (2295407, 2784536) | -16.7  (-24.24, -8.1) | 892485  (740613, 1103185) | -39.73  (-49.99, -25.51) | 26959276  (21478558, 32354701) | -32.33  (-46.09, -18.79) |
| China | Intracerebral hemorrhage | 4284271  (4058386, 4507387) | -17.87  (-24.05, -12.8) | 589785  (517422, 667914) | -50.46  (-57.91, -42.45) | 370868  (307100, 460863) | -71.81  (-77.05, -66.01) | 11737668  (9072472, 14413075) | -66.95  (-75.3, -57.57) |
| China | Ischemic stroke | 19580752  (18530978, 20670857) | -19.81  (-25, -14.58) | 1781733  (1639183, 1947730) | -46.87  (-51.71, -41.51) | 426993  (352201, 536489) | -59.16  (-66.25, -48.29) | 12498597  (10021858, 14885023) | -51.38  (-63.03, -40.87) |
| China | Subarachnoid hemorrhage | 2055520  (1849150, 2425938) | 30.31  (18.36, 44.62) | 152288  (138802, 168892) | -1.79  (-8.11, 8.54) | 94623  (81311, 105833) | -5.31  (-15.92, 14.86) | 2723011  (2384228, 3056603) | 11.11  (-6.08, 31.04) |
| Colombia | All stroke | 791112  (760059, 819386) | 57.13  (50.96, 62.74) | 63329  (58436, 68968) | 84.96  (70.67, 101.43) | 18440  (17094, 19977) | 63.06  (51.18, 76.66) | 512950  (407523, 629375) | 51.35  (20.25, 85.71) |
| Colombia | Intracerebral hemorrhage | 81967  (75740, 88988) | 13.35  (3.01, 22.8) | 10750  (9259, 12584) | 5.44  (-19.74, 24.89) | 7041  (6429, 7770) | 1.94  (-6.61, 11.52) | 152336  (110475, 193845) | -27.74  (-55.25, -1.49) |
| Colombia | Ischemic stroke | 439661  (420226, 459851) | 17.14  (9.13, 22.76) | 43679  (40521, 47298) | 38.59  (26.26, 51.02) | 7760  (7265, 8281) | 3.86  (-2.65, 10.62) | 235483  (180766, 302081) | 13.06  (-25.9, 57.62) |
| Colombia | Subarachnoid hemorrhage | 115028  (107823, 120497) | -10.12  (-14.09, -6.53) | 8901  (8656, 9087) | -4.72  (-7.58, -2.93) | 3639  (3399, 3927) | -16.01  (-20.88, -11.15) | 125130  (116281, 133448) | -4.78  (-11.33, 2.05) |
| Comoros | All stroke | 15405  (14455, 16484) | 22.44  (14.89, 31.02) | 1498  (1351, 1639) | 24.12  (12, 35.85) | 702  (612, 804) | 14.75  (0.06, 31.39) | 21238  (15911, 27222) | 19.55  (-10.43, 53.24) |
| Comoros | Intracerebral hemorrhage | 2526  (2385, 2728) | 17.05  (10.61, 26.54) | 573  (504, 636) | 26.69  (11.4, 40.03) | 432  (364, 510) | -21.13  (-33.94, -7.36) | 12847  (9525, 16754) | -12.55  (-35.35, 12.5) |
| Comoros | Ischemic stroke | 10053  (9216, 10883) | -2.74  (-10.56, 5.06) | 854  (780, 931) | -9.17  (-16.97, -1.16) | 246  (225, 269) | -3.57  (-11.36, 5.46) | 7152  (5324, 9015) | 5.11  (-20.2, 32.02) |
| Comoros | Subarachnoid hemorrhage | 1175  (1114, 1276) | 9.58  (3.84, 18.68) | 71  (67, 73) | -5.74  (-10.43, -2.93) | 24  (23, 25) | -1.07  (-5.69, 3.56) | 1239  (1062, 1453) | 33.1  (14.25, 55.93) |
| Congo | All stroke | 107551  (101755, 113744) | 18.19  (11.82, 24.99) | 9437  (8854, 10109) | -0.47  (-6.61, 6.62) | 5026  (4430, 5508) | 0.07  (-11.79, 9.68) | 163623  (145175, 184510) | 6.95  (-5.11, 20.6) |
| Congo | Intracerebral hemorrhage | 16398  (15629, 17363) | -3.53  (-7.65, 1.99) | 2984  (2742, 3209) | -18.93  (-25.4, -13.31) | 2828  (2416, 3183) | -35.24  (-45.17, -26.84) | 95787  (84552, 108450) | -25.31  (-33.04, -15.73) |
| Congo | Ischemic stroke | 71759  (67249, 76234) | -8.49  (-13.95, -3.6) | 6013  (5684, 6448) | -19.09  (-23.01, -13.62) | 2058  (1890, 2173) | -8.65  (-16.16, -3.7) | 60991  (54949, 67985) | -1.41  (-9.58, 7.01) |
| Congo | Subarachnoid hemorrhage | 7355  (6828, 8083) | 12.9  (5.52, 23.57) | 440  (428, 451) | -7.06  (-9.83, -4.85) | 140  (124, 152) | -22.34  (-31.62, -15.12) | 6845  (5675, 8076) | 0.71  (-14.37, 17) |
| Costa Rica | All stroke | 78619  (72591, 83918) | 52.5  (40.81, 62.78) | 5077  (4406, 5763) | 55.73  (35.16, 76.78) | 1212  (1061, 1410) | 43.5  (25.55, 66.84) | 29848  (18215, 45377) | 15.81  (-29.33, 76.06) |
| Costa Rica | Intracerebral hemorrhage | 7200  (6632, 7926) | -16.03  (-26.15, -7.8) | 882  (707, 1038) | -23.37  (-47.52, -1.63) | 444  (379, 526) | -20.31  (-32.71, -7.29) | 11510  (6165, 16558) | -17.51  (-59.66, 22.03) |
| Costa Rica | Ischemic stroke | 41008  (37679, 44922) | 1.82  (-10.71, 13.76) | 3608  (3125, 4122) | 6.77  (-12.97, 27.34) | 547  (476, 650) | -6.81  (-17.89, 11.07) | 12723  (7161, 22489) | -22.35  (-60.05, 56.37) |
| Costa Rica | Subarachnoid hemorrhage | 10461  (10117, 10900) | -2.77  (-6.9, 1.54) | 587  (574, 602) | -3.73  (-5.73, -1.45) | 222  (206, 234) | -2.71  (-7.53, 1.51) | 5615  (4889, 6330) | -25.97  (-37.25, -14.39) |
| Croatia | All stroke | 59857  (55487, 63730) | 2.79  (-4.72, 9.44) | 4180  (3873, 4557) | -22.52  (-28.2, -15.52) | 1264  (1142, 1478) | -36.69  (-42.76, -25.96) | 31883  (26075, 37647) | -36.81  (-48.32, -25.39) |
| Croatia | Intracerebral hemorrhage | 4667  (4276, 5167) | 11.14  (-3.38, 25.84) | 766  (719, 830) | -14.34  (-25.1, -3.24) | 561  (517, 643) | -31.46  (-39.2, -23.24) | 12947  (10840, 14966) | -36.98  (-57.61, -15.44) |
| Croatia | Ischemic stroke | 32248  (29709, 35187) | -35.78  (-47.01, -25.28) | 3174  (2917, 3482) | -43.27  (-52.68, -32.13) | 557  (494, 673) | -61.1  (-66.49, -53.49) | 13687  (10268, 17199) | -60.51  (-75.51, -42.6) |
| Croatia | Subarachnoid hemorrhage | 4939  (4463, 5415) | 35.43  (17.52, 53.27) | 241  (237, 245) | 2.02  (0.81, 3.36) | 145  (132, 162) | -11.23  (-15.93, -4.18) | 5249  (4967, 5482) | 8.89  (0.73, 16.51) |
| Cuba | All stroke | 171525  (159701, 187034) | 31.01  (21.98, 42.86) | 12155  (10729, 13701) | 13.7  (0.37, 28.16) | 4730  (3582, 6021) | -13.39  (-34.42, 10.27) | 98537  (65208, 131385) | -30.66  (-54.11, -7.54) |
| Cuba | Intracerebral hemorrhage | 17539  (16025, 20803) | -12.12  (-20.47, 6.12) | 2708  (2258, 3177) | -28.1  (-44.27, -13.06) | 2151  (1684, 2580) | -34.97  (-48.53, -21.65) | 37653  (23733, 49807) | -63.61  (-78.53, -48.58) |
| Cuba | Ischemic stroke | 98292  (91111, 110060) | -13.83  (-22.96, -1.11) | 8384  (7431, 9447) | -26.65  (-34.97, -13.66) | 2105  (1438, 2944) | -49.26  (-62.76, -32.28) | 42951  (26336, 60893) | -48.39  (-73.37, -25.38) |
| Cuba | Subarachnoid hemorrhage | 21380  (20357, 22537) | -0.09  (-4.87, 6.58) | 1063  (1040, 1077) | -1.56  (-4.13, -0.18) | 473  (460, 497) | 0.82  (-1.91, 5.46) | 17933  (15139, 20685) | 8.99  (-10.2, 31.47) |
| Cyprus | All stroke | 15643  (13137, 17113) | 39.15  (16.86, 52.22) | 1238  (1037, 1397) | 59.76  (33.88, 80.33) | 349  (323, 377) | 20.44  (11.42, 30.13) | 8777  (6208, 11229) | 14.54  (-18.99, 46.54) |
| Cyprus | Intracerebral hemorrhage | 2460  (2240, 2827) | -3.78  (-13.61, 14.07) | 284  (252, 308) | -19.7  (-33.77, -7) | 136  (125, 147) | -29.27  (-34.8, -23.99) | 2107  (1276, 2985) | -64.02  (-82.98, -38.2) |
| Cyprus | Ischemic stroke | 8012  (6115, 9367) | -36.43  (-63.54, -12.31) | 835  (670, 968) | -4.08  (-41.2, 25.76) | 166  (152, 182) | -21.97  (-28.76, -13.58) | 4892  (3254, 6341) | -27.41  (-59.25, 6.97) |
| Cyprus | Subarachnoid hemorrhage | 2410  (2250, 2529) | 20.94  (11.78, 29.35) | 119  (115, 122) | -3.98  (-6.48, -2.05) | 47  (46, 48) | -0.42  (-2.11, 1.69) | 1779  (1677, 1903) | 25.98  (17.02, 38.4) |
| Democratic Republic of the Congo | All stroke | 1364984  (1253701, 1471338) | 3.33  (-5.1, 11.38) | 147662  (135432, 160197) | -1.23  (-9.41, 7.16) | 78282  (69429, 88688) | -2.81  (-13.8, 10.11) | 2130090  (1503802, 2718234) | -11.25  (-37.34, 13.26) |
| Democratic Republic of the Congo | Intracerebral hemorrhage | 315727  (289417, 347550) | 16.43  (7.76, 27.11) | 56553  (51792, 60624) | -3.45  (-10.35, 3.1) | 54367  (47400, 62073) | -5.06  (-16.53, 5.67) | 1367964  (967796, 1714988) | -24.88  (-42.16, -10.24) |
| Democratic Republic of the Congo | Ischemic stroke | 878587  (781435, 970037) | 2.13  (-5.67, 10.02) | 81714  (74691, 89854) | 1.01  (-6.02, 9.03) | 20730  (19144, 22314) | 1.46  (-4.76, 9.04) | 559354  (367506, 755394) | -5.36  (-24.73, 15.95) |
| Democratic Republic of the Congo | Subarachnoid hemorrhage | 143028  (134408, 154810) | 16.54  (10.3, 25.24) | 9395  (8948, 9719) | -2.59  (-7.03, 0.49) | 3186  (2885, 4301) | 3.36  (-5.03, 27.85) | 202772  (168500, 247853) | 58.3  (33.52, 87.94) |
| Denmark | All stroke | 93700  (88193, 99183) | 4.13  (-1.98, 10.23) | 6384  (5926, 6776) | 12.17  (4.12, 19.05) | 1374  (1229, 1516) | -14.59  (-23.58, -5.77) | 52678  (46937, 59450) | 18.76  (5.81, 34.02) |
| Denmark | Intracerebral hemorrhage | 9483  (9094, 9929) | 2.87  (-3.08, 8.51) | 1195  (1107, 1290) | 4.04  (-8.16, 15.2) | 565  (496, 641) | -25.75  (-34.9, -14.38) | 19117  (16941, 21648) | 11.57  (-9.89, 26.85) |
| Denmark | Ischemic stroke | 56057  (52723, 59474) | -2.99  (-8.86, 4.51) | 4639  (4285, 4921) | -1.26  (-12.23, 5.16) | 608  (540, 664) | -24.97  (-33.66, -18.21) | 24845  (21667, 28680) | 10.94  (-7.68, 34.88) |
| Denmark | Subarachnoid hemorrhage | 9581  (9183, 9992) | -0.02  (-4.8, 5.3) | 551  (534, 564) | -4.73  (-7.04, -2.94) | 201  (193, 211) | -8  (-10.91, -5.09) | 8716  (8329, 9122) | 33.09  (26.06, 39.11) |
| Djibouti | All stroke | 18068  (16802, 19625) | 12.71  (4.82, 22.43) | 1503  (1364, 1624) | -6.09  (-14.77, 1.45) | 921  (822, 1034) | 27.23  (13.57, 42.83) | 19727  (15340, 23881) | -13.22  (-32.52, 5.06) |
| Djibouti | Intracerebral hemorrhage | 2019  (1881, 2213) | -36.06  (-40.71, -29.77) | 321  (282, 356) | -49.05  (-55.41, -43.4) | 528  (457, 594) | -26.31  (-36.61, -17.16) | 10526  (8242, 12452) | -47.79  (-59.29, -37.45) |
| Djibouti | Ischemic stroke | 12695  (11750, 13590) | -12.43  (-19.13, -5.25) | 1090  (995, 1173) | -20.09  (-27.95, -13.85) | 368  (341, 411) | 7.78  (0.15, 19.44) | 8297  (6366, 10318) | -19.16  (-41.32, 3.72) |
| Djibouti | Subarachnoid hemorrhage | 1316  (1194, 1463) | -9.9  (-18.64, 1.14) | 92  (87, 95) | -12.78  (-17.78, -9.89) | 26  (23, 29) | -27.89  (-33.67, -19.05) | 905  (733, 1111) | -35.44  (-48.18, -20.23) |
| Dominican Republic | All stroke | 175919  (161222, 191173) | 24.54  (14.14, 35.34) | 10074  (8850, 11301) | -27.34  (-36.17, -18.49) | 2822  (2375, 3690) | -57.8  (-64.49, -44.82) | 129525  (101632, 153754) | -34.13  (-48.32, -21.81) |
| Dominican Republic | Intracerebral hemorrhage | 19864  (17806, 21826) | -29.43  (-37.32, -21.82) | 1719  (1369, 2138) | -73.69  (-80.48, -66.03) | 1347  (1074, 1873) | -74.74  (-80.9, -65.46) | 67664  (57107, 77055) | -52.48  (-60.5, -45.31) |
| Dominican Republic | Ischemic stroke | 101272  (94214, 108969) | -7.52  (-15.44, 0.3) | 6942  (6113, 7715) | -35.93  (-44.43, -28.31) | 1012  (867, 1322) | -71.66  (-76.04, -63.16) | 47381  (31372, 60947) | -38.12  (-60.81, -17.57) |
| Dominican Republic | Subarachnoid hemorrhage | 20489  (19578, 21354) | -1.25  (-5.49, 3.48) | 1414  (1367, 1448) | -3.6  (-6.58, -1.64) | 464  (434, 495) | -19.92  (-25.37, -15.93) | 14479  (13154, 15751) | -33.36  (-39.57, -27.36) |
| Ecuador | All stroke | 269381  (254035, 280498) | 29.59  (22.2, 34.93) | 20920  (19311, 22679) | 41.83  (30.91, 53.76) | 7431  (6813, 8039) | 49.17  (36.77, 61.36) | 228233  (184735, 271861) | 46.52  (18.59, 74.53) |
| Ecuador | Intracerebral hemorrhage | 30314  (28019, 32803) | 1.41  (-7.16, 10.1) | 4077  (3432, 4722) | -11.3  (-28.36, 9.15) | 2929  (2578, 3303) | -9.42  (-18.76, 3.45) | 86666  (68167, 105468) | -0.86  (-25.97, 23.41) |
| Ecuador | Ischemic stroke | 152903  (143725, 161828) | -2.64  (-9.18, 3.74) | 13483  (12629, 14510) | 7.93  (-2.59, 16.57) | 2689  (2523, 2869) | 7.68  (0.7, 16.41) | 96371  (74356, 118093) | 47.99  (5.18, 83.09) |
| Ecuador | Subarachnoid hemorrhage | 40992  (39285, 42860) | -19.82  (-23.22, -15.65) | 3360  (3250, 3447) | -4.79  (-7.88, -1.28) | 1813  (1713, 1867) | -2.86  (-7.81, 0) | 45196  (42212, 48299) | -18  (-24.13, -12.52) |
| Egypt | All stroke | 1489419  (1411787, 1551942) | 6.42  (0.87, 10.89) | 132731  (121909, 143337) | -11.54  (-18.75, -4.47) | 71159  (63810, 81006) | -22.5  (-30.5, -11.77) | 2610119  (2248200, 2920669) | -0.53  (-14.32, 11.31) |
| Egypt | Intracerebral hemorrhage | 138237  (128060, 150541) | -14.88  (-21.02, -6.94) | 19021  (15537, 21914) | -37.41  (-50.59, -26.14) | 21873  (19740, 25099) | -34.04  (-40.91, -24.27) | 745431  (618402, 867803) | -22.13  (-35.92, -9.31) |
| Egypt | Ischemic stroke | 1166180  (1105489, 1225386) | -17.13  (-21.5, -12.85) | 107075  (99882, 114616) | -32.49  (-37.52, -27.06) | 46568  (41518, 53005) | -53.16  (-58.11, -46.98) | 1694596  (1481586, 1852972) | -33.94  (-43.02, -26.84) |
| Egypt | Subarachnoid hemorrhage | 109085  (98786, 120052) | 16.32  (5.1, 28.08) | 6635  (6489, 6806) | -0.37  (-2.42, 2.23) | 2717  (2552, 2902) | -9.57  (-15.65, -3.04) | 170091  (148212, 199894) | 68.2  (43.99, 95.85) |
| El Salvador | All stroke | 79531  (76658, 82521) | 55.35  (49.73, 61.19) | 5566  (5204, 5956) | 56.99  (46.79, 68.01) | 1898  (1776, 2022) | 33.76  (25.2, 42.51) | 60377  (48097, 71497) | 42.1  (13.2, 68.27) |
| El Salvador | Intracerebral hemorrhage | 8263  (7835, 8759) | 8.44  (1.88, 15.44) | 1255  (1102, 1394) | 8.27  (-6.07, 22.31) | 897  (816, 980) | -11.03  (-19.34, -2.79) | 14920  (9808, 19580) | -43.49  (-63.81, -21.24) |
| El Salvador | Ischemic stroke | 44212  (41963, 46389) | 32.58  (24.72, 39.97) | 3658  (3457, 3903) | 30.34  (20.33, 40.57) | 748  (713, 783) | -1.14  (-6.44, 4.15) | 32846  (26755, 38311) | 77.69  (41.02, 113.1) |
| El Salvador | Subarachnoid hemorrhage | 12321  (11560, 13325) | 28.16  (19.78, 39.54) | 652  (645, 659) | 1.24  (0.29, 2.47) | 253  (247, 259) | 1.91  (-0.54, 5.18) | 12612  (11534, 13606) | 46.72  (34.77, 59.71) |
| Equatorial Guinea | All stroke | 24736  (21499, 27730) | 59.3  (38.46, 78.58) | 2008  (1739, 2238) | 47.13  (27.47, 64.01) | 646  (551, 757) | 3.3  (-11.94, 20.97) | 25882  (19356, 35245) | 34.68  (0.72, 83.4) |
| Equatorial Guinea | Intracerebral hemorrhage | 3600  (3261, 3959) | 19.29  (8.82, 30.84) | 522  (452, 593) | -3.55  (-16.82, 8.11) | 337  (284, 404) | -40.36  (-48.76, -27.55) | 12001  (8608, 16954) | -24.75  (-45.92, 5.14) |
| Equatorial Guinea | Ischemic stroke | 16211  (14340, 17944) | 24.13  (10.51, 35.8) | 1396  (1200, 1552) | 19.78  (5.29, 32.42) | 285  (245, 327) | -24.22  (-35.36, -13.79) | 12572  (9761, 16554) | 23.53  (-1.2, 60.42) |
| Equatorial Guinea | Subarachnoid hemorrhage | 2383  (2112, 2714) | 99.19  (78.02, 125.7) | 89  (87, 93) | 5.06  (2.3, 8.97) | 24  (22, 25) | 19.81  (11.32, 27.36) | 1310  (986, 1736) | 55.29  (19.61, 103.94) |
| Eritrea | All stroke | 66086  (61631, 70833) | -24.42  (-29.52, -18.99) | 6103  (5518, 6735) | -36.88  (-42.93, -30.35) | 5184  (4844, 5559) | -7.24  (-13.32, -0.54) | 135384  (119772, 153847) | -23.93  (-32.7, -13.56) |
| Eritrea | Intracerebral hemorrhage | 13192  (11899, 14474) | -30.95  (-37.11, -24.5) | 2265  (2063, 2465) | -41.91  (-46.21, -36.95) | 3657  (3377, 3967) | -22.27  (-28.88, -15.84) | 92506  (84242, 102860) | -37.75  (-43.15, -31.54) |
| Eritrea | Ischemic stroke | 44322  (39741, 48835) | -26.73  (-33.19, -20.14) | 3415  (3076, 3790) | -36.76  (-41.86, -30.87) | 1341  (1298, 1393) | -0.15  (-3.11, 3.94) | 36788  (30131, 44232) | -1.98  (-16.93, 12) |
| Eritrea | Subarachnoid hemorrhage | 4880  (4430, 5318) | -37.18  (-42.52, -32.14) | 423  (379, 480) | -31.78  (-38.44, -23) | 186  (170, 200) | -19.29  (-25.45, -14.42) | 6090  (5399, 6755) | -37.36  (-43.88, -30.85) |
| Estonia | All stroke | 17402  (15167, 20288) | 3.72  (-9.6, 20.93) | 1471  (1286, 1648) | 12.73  (-1.41, 26.34) | 301  (278, 334) | -26.57  (-32.18, -18.49) | 10566  (8411, 12805) | -11.92  (-29.88, 6.75) |
| Estonia | Intracerebral hemorrhage | 1941  (1525, 2520) | 58.4  (15.89, 114.39) | 218  (181, 244) | -15.03  (-42.87, 8.66) | 113  (105, 122) | -30.3  (-35.11, -22.76) | 1608  (846, 2210) | -62.15  (-83.53, -34.09) |
| Estonia | Ischemic stroke | 12148  (10526, 13871) | -30.58  (-49.34, -9.82) | 1162  (1016, 1312) | -27.15  (-45.55, -7.92) | 149  (135, 170) | -61.05  (-64.67, -55.48) | 7543  (6256, 9017) | -32.46  (-54.26, -7.43) |
| Estonia | Subarachnoid hemorrhage | 2142  (1959, 2398) | 73.66  (52.28, 94.14) | 91  (89, 92) | -2  (-3.74, -0.75) | 39  (37, 42) | -6.69  (-11.52, -2.6) | 1415  (1310, 1579) | 6.83  (-4.13, 21.43) |
| Eswatini | All stroke | 15918  (14849, 17008) | 26.9  (18.37, 35.59) | 1792  (1644, 1926) | 22.78  (12.6, 31.92) | 704  (624, 787) | -28.6  (-36.65, -20.1) | 24550  (19438, 29101) | -14.31  (-32.15, 1.57) |
| Eswatini | Intracerebral hemorrhage | 2373  (2183, 2526) | -2.88  (-10.24, 3.17) | 521  (467, 557) | -13.95  (-21.94, -8.21) | 407  (354, 458) | -52.77  (-58.79, -46.85) | 14082  (11731, 16501) | -41.77  (-50.46, -32.85) |
| Eswatini | Ischemic stroke | 11627  (10850, 12383) | 10.95  (4.56, 17.63) | 1210  (1117, 1305) | 5.13  (-2.36, 12.73) | 274  (249, 306) | -36.54  (-42.4, -29.27) | 9244  (6645, 11157) | -14.82  (-34.32, 0.89) |
| Eswatini | Subarachnoid hemorrhage | 1479  (1342, 1702) | 66.44  (51.97, 92.21) | 62  (60, 64) | -1.69  (-4.12, 1.31) | 22  (21, 23) | -7.98  (-12.68, -3.76) | 1224  (1063, 1443) | 34.86  (18.6, 57.28) |
| Ethiopia | All stroke | 1444602  (1313381, 1591327) | 44.69  (31.55, 59.39) | 165725  (147288, 181473) | 69.45  (50.61, 85.56) | 64001  (57167, 73863) | 30.17  (16.29, 50.25) | 2520287  (1999123, 3024042) | 70.76  (35.45, 104.89) |
| Ethiopia | Intracerebral hemorrhage | 295915  (272702, 320269) | 31.05  (20.97, 41.53) | 58742  (53151, 63878) | 20.24  (9.03, 29.46) | 42974  (38157, 49238) | -15.71  (-25.68, -3.76) | 1541928  (1242886, 1798585) | 11.67  (-9.34, 28.16) |
| Ethiopia | Ischemic stroke | 896939  (805918, 1004702) | 26.15  (14.25, 38.87) | 98975  (86347, 109386) | 51.21  (34.29, 65.77) | 18345  (16593, 21445) | 19.21  (7.87, 39.74) | 816144  (618524, 1040377) | 100.94  (60.18, 144.52) |
| Ethiopia | Subarachnoid hemorrhage | 132873  (122133, 143532) | 17.58  (8.33, 26.16) | 8009  (7790, 8209) | -1.22  (-4.01, 1.22) | 2682  (2417, 3180) | 16.76  (4.62, 35.84) | 162215  (137713, 185080) | 75.2  (50.06, 98.51) |
| Fiji | All stroke | 17125  (16053, 18459) | 18.45  (11.04, 27.68) | 1748  (1604, 1916) | 31.5  (20.68, 44.13) | 676  (567, 773) | -14.41  (-28.23, -2.13) | 24680  (20759, 28678) | -0.73  (-16.5, 15.35) |
| Fiji | Intracerebral hemorrhage | 2998  (2797, 3197) | 5.18  (-2.37, 12.07) | 708  (642, 776) | 40.64  (25.21, 53.9) | 432  (346, 501) | -41.33  (-51.87, -30.62) | 15671  (13564, 18034) | -25.06  (-35.86, -12.6) |
| Fiji | Ischemic stroke | 10220  (9528, 11329) | -5.57  (-12.43, 5.77) | 890  (819, 983) | -5.99  (-13.87, 5.64) | 183  (164, 208) | -17.85  (-26.73, -7.25) | 6175  (4607, 7458) | -17.82  (-42.4, 1.73) |
| Fiji | Subarachnoid hemorrhage | 2029  (1857, 2247) | -3.94  (-12.02, 6.96) | 150  (143, 156) | -6.24  (-11.07, -2.08) | 61  (58, 64) | -9.75  (-14.01, -4.81) | 2835  (2588, 3186) | 6.76  (-2.03, 21.37) |
| Finland | All stroke | 138111  (131941, 144180) | 2.14  (-2.43, 6.62) | 7958  (7442, 8588) | 5.83  (-1.03, 14.2) | 1344  (1208, 1496) | -9.5  (-18.66, 0.7) | 53495  (47087, 60051) | 16.86  (2.87, 31.19) |
| Finland | Intracerebral hemorrhage | 15015  (14052, 15915) | 19.45  (10.13, 33.03) | 1193  (1086, 1291) | -21.01  (-31.54, -9.87) | 486  (436, 531) | -20.58  (-30.57, -13.82) | 15543  (13698, 17608) | 9.62  (-8.46, 26.96) |
| Finland | Ischemic stroke | 70080  (65698, 74096) | -5.72  (-11.41, 0.39) | 6062  (5670, 6573) | 2.3  (-6.8, 10.98) | 642  (569, 713) | -19.31  (-29.43, -10.79) | 28065  (23994, 32160) | 17.67  (-3.91, 37.37) |
| Finland | Subarachnoid hemorrhage | 15169  (14500, 15750) | 8.76  (3.95, 13.17) | 704  (686, 724) | -6.9  (-10.24, -4.46) | 216  (203, 252) | -17.56  (-21.63, -10.94) | 9887  (9395, 10282) | 12.96  (7.4, 18.6) |
| France | All stroke | 1446416  (1357028, 1563708) | 50.54  (41.24, 62.75) | 66810  (59535, 71989) | 20.96  (7.78, 30.33) | 14496  (13190, 16291) | 37.31  (24.93, 54.32) | 377172  (316332, 438501) | 11.2  (-6.73, 29.28) |
| France | Intracerebral hemorrhage | 105742  (96164, 117356) | 19.59  (3.64, 36.74) | 12166  (10763, 13409) | 9.19  (-9.95, 26.54) | 5780  (5346, 6158) | 4.07  (-3.46, 10.59) | 114285  (88071, 133707) | -23.95  (-44.95, 0.69) |
| France | Ischemic stroke | 695461  (645822, 753943) | 11.96  (3.06, 21.1) | 49577  (43764, 53437) | -5.45  (-16.34, 4.25) | 6779  (6006, 8108) | 19.81  (7.31, 40.8) | 192704  (162774, 229153) | 1.22  (-22.79, 27.08) |
| France | Subarachnoid hemorrhage | 110460  (101512, 120969) | 24.25  (13.67, 36.78) | 5068  (5009, 5143) | -2.12  (-3.37, -0.87) | 1937  (1838, 2024) | 1.8  (-0.9, 5.41) | 70183  (65488, 75642) | 3.26  (-5.1, 13.26) |
| Gabon | All stroke | 50322  (41096, 63406) | 72.87  (41.17, 117.82) | 3107  (2750, 3454) | 11.04  (-1.75, 23.41) | 1211  (996, 1392) | -7.91  (-24.25, 5.84) | 43271  (32438, 53657) | 12.41  (-15.73, 39.39) |
| Gabon | Intracerebral hemorrhage | 6488  (5669, 7713) | 20.77  (5.63, 43.61) | 791  (701, 903) | -27.8  (-35.56, -17.58) | 674  (556, 779) | -44.28  (-54.27, -35.85) | 26238  (20085, 32102) | -20.29  (-38.61, -2.94) |
| Gabon | Ischemic stroke | 28013  (24744, 32564) | 8.21  (-4.04, 25.48) | 2174  (1910, 2403) | -10.29  (-21.1, -0.82) | 495  (401, 569) | -28.16  (-42.05, -17.71) | 15050  (10807, 19014) | -18.32  (-40.61, 2.49) |
| Gabon | Subarachnoid hemorrhage | 3563  (3023, 4227) | 68.14  (42.89, 99.13) | 142  (138, 147) | -2.76  (-4.97, 0.92) | 41  (39, 44) | -6.06  (-11.52, 1.21) | 1983  (1546, 2540) | 20.54  (-6.14, 53.42) |
| Gambia | All stroke | 44243  (40010, 48563) | 13.59  (2.73, 24.68) | 3704  (3318, 4136) | 7.36  (-3.83, 19.89) | 2570  (2315, 2941) | 8  (-2.69, 23.58) | 63359  (49277, 78010) | -11.38  (-31.07, 9.12) |
| Gambia | Intracerebral hemorrhage | 9239  (8356, 10738) | 3.03  (-6.54, 17.99) | 1253  (1105, 1388) | -22.6  (-30.57, -15.56) | 1292  (1134, 1570) | -29.56  (-38.39, -14.43) | 34574  (26754, 42761) | -34.86  (-48.89, -21.6) |
| Gambia | Ischemic stroke | 28849  (24901, 33499) | -5.34  (-16.71, 6.87) | 2298  (2063, 2588) | -5.62  (-13.56, 5.1) | 1205  (1114, 1293) | -13.68  (-20.29, -7.1) | 25731  (19918, 31687) | -25.5  (-38.2, -14) |
| Gambia | Subarachnoid hemorrhage | 3162  (2861, 3590) | 20.07  (10.08, 36.13) | 153  (149, 160) | 1.39  (-0.8, 5.78) | 72  (68, 77) | -10.96  (-16.44, -5.14) | 3054  (2605, 3562) | -2.35  (-15.45, 11.13) |
| Georgia | All stroke | 199028  (185481, 213048) | -20.24  (-25.67, -14.62) | 12307  (11159, 13435) | -24.31  (-31.38, -17.38) | 2969  (2076, 4212) | -60.15  (-72.14, -43.47) | 157466  (136905, 180115) | -24.51  (-34.37, -13.65) |
| Georgia | Intracerebral hemorrhage | 23116  (20122, 26356) | -26.14  (-37.57, -15.31) | 3321  (2913, 3734) | -22.53  (-32.6, -13.2) | 1150  (823, 1731) | -68.67  (-77.88, -56.3) | 76198  (67347, 85347) | -16.56  (-30.02, -4.78) |
| Georgia | Ischemic stroke | 123533  (113613, 132996) | -30.7  (-37.26, -24.03) | 7862  (7262, 8458) | -35.52  (-41.7, -29.1) | 1366  (889, 1930) | -68.03  (-78.2, -55.21) | 57815  (48483, 68531) | -46.62  (-59.18, -32.48) |
| Georgia | Subarachnoid hemorrhage | 13222  (12062, 14441) | -30.43  (-38.2, -24.63) | 1124  (984, 1243) | -27.19  (-41.59, -15.64) | 453  (364, 551) | -48.79  (-61.27, -38.81) | 23453  (21076, 26237) | -8.99  (-20.97, -0.16) |
| Germany | All stroke | 2212340  (2134743, 2287921) | 17.18  (13.07, 21.18) | 128294  (120865, 134494) | 21.21  (14.19, 27.07) | 22104  (20476, 23609) | 14.46  (6.04, 22.24) | 854094  (780560, 922172) | 43.26  (30.92, 54.68) |
| Germany | Intracerebral hemorrhage | 162065  (156731, 170176) | 13.4  (7.79, 20.16) | 19967  (18545, 21279) | 26.24  (11.17, 38.9) | 8094  (7512, 8706) | -3.32  (-12.11, 3.98) | 247835  (227529, 271072) | 27.4  (9.92, 44.75) |
| Germany | Ischemic stroke | 1249953  (1190758, 1304798) | 0.13  (-5.35, 6.2) | 102088  (96183, 106893) | 1.2  (-5.72, 7.79) | 11547  (10575, 12337) | -11.75  (-19.09, -5.39) | 507317  (459709, 545052) | 22.63  (6.55, 39.3) |
| Germany | Subarachnoid hemorrhage | 139843  (132407, 153527) | 10.98  (6.61, 17.48) | 6238  (6138, 6322) | -3.4  (-5.05, -1.88) | 2462  (2389, 2566) | -6.23  (-8.95, -3.11) | 98942  (93322, 106048) | 11.93  (5.11, 18.73) |
| Ghana | All stroke | 809344  (756147, 849819) | 11.01  (3.71, 16.56) | 67268  (62798, 72205) | 5.36  (-1.64, 13.09) | 33367  (29115, 38606) | -8.49  (-20.15, 5.88) | 1000260  (847001, 1151566) | -12.65  (-26.03, 0.57) |
| Ghana | Intracerebral hemorrhage | 139118  (127102, 170836) | -10.18  (-17.92, 10.05) | 16783  (15391, 17926) | -35.86  (-41.05, -31.51) | 17651  (15522, 20470) | -36.68  (-44.66, -26.78) | 496473  (408782, 583987) | -39.3  (-49.81, -29.23) |
| Ghana | Ischemic stroke | 561652  (526163, 597695) | -10.79  (-16.4, -5.6) | 48102  (45072, 51847) | -3.34  (-9.02, 4.18) | 14749  (12727, 17073) | -36.09  (-44.43, -25.62) | 460441  (403444, 510825) | -25.92  (-35.21, -18.24) |
| Ghana | Subarachnoid hemorrhage | 50111  (44969, 55746) | 15.57  (4.06, 28.55) | 2383  (2335, 2432) | -1.82  (-3.8, 0.15) | 966  (866, 1063) | -22.51  (-30.41, -14.91) | 43347  (34775, 56754) | -13.17  (-30.27, 13.42) |
| Greece | All stroke | 199668  (190842, 210227) | 22.92  (17.49, 29.42) | 13193  (12359, 14144) | 22.23  (14.5, 31.03) | 3353  (3066, 3695) | -2.99  (-11.29, 6.91) | 95132  (82020, 106604) | 3.68  (-10.61, 16.19) |
| Greece | Intracerebral hemorrhage | 20261  (19043, 21421) | -0.17  (-9.26, 8.15) | 2693  (2496, 2922) | -10.97  (-20.14, 0.81) | 1576  (1444, 1733) | -34.68  (-40.88, -26.87) | 48050  (42721, 52925) | -13.12  (-26.74, -1.04) |
| Greece | Ischemic stroke | 102541  (96631, 108337) | -0.94  (-8.48, 7.95) | 9699  (9074, 10410) | 2.2  (-8.48, 13.93) | 1413  (1265, 1593) | -11.87  (-20.58, -2.15) | 37394  (30316, 43228) | -20.42  (-45.4, 5.74) |
| Greece | Subarachnoid hemorrhage | 14930  (14035, 15727) | -1.7  (-7.91, 5.47) | 802  (790, 811) | -4.15  (-6.07, -2.77) | 364  (357, 369) | -3.97  (-5.96, -1.66) | 9689  (8983, 10451) | -21.12  (-29.2, -14.22) |
| Grenada | All stroke | 1700  (1390, 1966) | 33.02  (8.81, 53.85) | 102  (74, 128) | -18.53  (-41.12, 2.66) | 56  (44, 70) | -23.51  (-39.65, -3.93) | 1213  (679, 1797) | -36.31  (-64.33, -5.62) |
| Grenada | Intracerebral hemorrhage | 226  (197, 258) | 1.29  (-12.71, 16.42) | 30  (22, 39) | -37.1  (-59.93, -10.36) | 28  (24, 33) | -38.51  (-46.35, -28.23) | 625  (344, 900) | -49.3  (-73.59, -19.45) |
| Grenada | Ischemic stroke | 930  (702, 1146) | -21.38  (-50.3, 2.91) | 59  (39, 76) | -48.78  (-70.91, -27.62) | 22  (15, 31) | -47.05  (-63.79, -26.52) | 429  (197, 712) | -56.47  (-82.1, -16.53) |
| Grenada | Subarachnoid hemorrhage | 196  (185, 205) | 0.43  (-5.64, 5.86) | 13  (12, 13) | -1.89  (-4.82, 0.66) | 6  (5, 6) | -16.98  (-26.09, -10.35) | 159  (139, 185) | -27.05  (-38.91, -14.52) |
| Guatemala | All stroke | 212099  (196746, 226641) | 52.68  (41.63, 63.15) | 16120  (14482, 17840) | 45.37  (30.61, 60.89) | 5748  (5300, 6277) | 36.81  (26.1, 49.4) | 132263  (94688, 172459) | -0.02  (-28.42, 30.36) |
| Guatemala | Intracerebral hemorrhage | 33659  (31191, 35844) | 2.95  (-4.43, 10.17) | 4506  (3751, 5171) | -6.67  (-23.57, 7.87) | 3279  (2949, 3631) | -15.36  (-24.42, -6.45) | 55181  (35729, 72687) | -48.61  (-67.23, -32.55) |
| Guatemala | Ischemic stroke | 110173  (101082, 117865) | 15.71  (6.53, 25.87) | 9756  (8891, 10776) | 25.67  (13.89, 39.9) | 1891  (1800, 2015) | 4.35  (-0.71, 11.87) | 49960  (34627, 69869) | 4.06  (-31.6, 50.1) |
| Guatemala | Subarachnoid hemorrhage | 29567  (28306, 30842) | 5.84  (1.39, 10.67) | 1858  (1839, 1894) | 0.94  (-0.1, 2.73) | 578  (550, 630) | 11.28  (6.23, 21.01) | 27122  (24332, 29903) | 34.54  (20.77, 48.52) |
| Guinea | All stroke | 227259  (214012, 240308) | 8.08  (1.78, 14.28) | 22099  (20630, 23343) | 9.68  (2.38, 15.85) | 13601  (12798, 14422) | 8.26  (1.87, 14.8) | 399095  (356246, 448513) | 6.25  (-5.16, 19.4) |
| Guinea | Intracerebral hemorrhage | 49782  (47221, 52076) | -8  (-12.6, -3.98) | 8385  (7822, 8920) | -15.8  (-20.41, -11) | 7889  (7398, 8517) | -5.96  (-12.79, 0.74) | 229491  (204275, 260422) | -10.02  (-19.29, 0.6) |
| Guinea | Ischemic stroke | 143976  (134996, 152776) | 3.47  (-2.27, 8.85) | 12870  (11975, 13566) | 14.31  (7.55, 19.5) | 5341  (5051, 5522) | -4.05  (-9.99, -0.98) | 153392  (137775, 168784) | 0.06  (-8.36, 8.08) |
| Guinea | Subarachnoid hemorrhage | 13872  (13110, 14636) | -2.86  (-7.83, 2.29) | 844  (832, 857) | 0.36  (-1.07, 1.78) | 371  (350, 384) | -3.91  (-8.9, -0.36) | 16212  (14195, 19307) | 4.09  (-8.19, 20.78) |
| Guyana | All stroke | 7124  (5388, 9301) | -20.03  (-39.52, 4.4) | 654  (517, 802) | -38.53  (-51.46, -24.63) | 162  (131, 195) | -77.31  (-81.66, -72.69) | 9312  (6897, 12421) | -54.44  (-66.26, -39.23) |
| Guyana | Intracerebral hemorrhage | 1676  (1534, 1853) | -15.52  (-23, -6.93) | 207  (174, 250) | -58.79  (-65.94, -49.55) | 86  (67, 106) | -83.5  (-87.5, -79.62) | 4883  (3742, 6385) | -65.92  (-74.5, -54.43) |
| Guyana | Ischemic stroke | 4813  (3716, 6268) | -27.69  (-46.77, -3.63) | 362  (261, 465) | -50.33  (-64.71, -32.88) | 48  (40, 57) | -87.11  (-89.25, -84.22) | 3305  (2182, 4767) | -57.29  (-73.1, -36.28) |
| Guyana | Subarachnoid hemorrhage | 1297  (1222, 1362) | 12.6  (5.72, 18.92) | 85  (81, 88) | -9.07  (-12.56, -5.74) | 28  (24, 32) | -40.92  (-50.31, -32.77) | 1124  (973, 1270) | -35.25  (-44.96, -26.6) |
| Haiti | All stroke | 135089  (129093, 141076) | 8.58  (3.76, 13.39) | 15387  (14287, 16695) | -1.49  (-8.53, 6.88) | 14048  (12073, 15479) | 7.87  (-7.29, 18.86) | 283822  (242769, 321253) | -27.72  (-38.17, -18.18) |
| Haiti | Intracerebral hemorrhage | 29492  (27858, 30871) | -7.87  (-12.76, -3.47) | 5658  (5170, 6307) | -28.64  (-35.1, -20.55) | 7865  (6813, 8738) | -26.49  (-36.16, -18.14) | 158066  (134357, 178264) | -47.53  (-55.44, -40.75) |
| Haiti | Ischemic stroke | 78454  (72967, 82964) | -10.86  (-17.27, -5.57) | 7800  (7247, 8387) | -16.25  (-22.3, -9.59) | 4783  (3903, 5223) | -16.77  (-31.49, -9.83) | 80967  (67262, 94737) | -40.96  (-51.28, -30.97) |
| Haiti | Subarachnoid hemorrhage | 19758  (19153, 20372) | -8.02  (-10.8, -5.06) | 1929  (1871, 2002) | -4.7  (-7.68, -1.2) | 1399  (1357, 1518) | -13.5  (-16.07, -6.24) | 44789  (41150, 48252) | -22.03  (-28.41, -15.99) |
| Honduras | All stroke | 147855  (140842, 154406) | 75.85  (67.51, 83.64) | 11992  (11052, 12962) | 58.31  (45.9, 71.11) | 10427  (8863, 11747) | 42.61  (21.21, 60.66) | 219827  (188327, 258831) | 9.72  (-6.01, 29.18) |
| Honduras | Intracerebral hemorrhage | 17768  (16377, 19420) | 14.37  (4.68, 25.23) | 2815  (2465, 3191) | -11.66  (-24.33, 1.17) | 4957  (4239, 5759) | -26.61  (-35.31, -14.43) | 106323  (91478, 129897) | -34.74  (-44.47, -19.27) |
| Honduras | Ischemic stroke | 88237  (83118, 94419) | 23.48  (15.35, 32.98) | 7570  (7002, 8146) | 9.02  (-0.56, 17.89) | 3895  (3097, 4368) | -19  (-33.89, -7.98) | 75843  (63129, 87866) | -26.73  (-40.63, -13.81) |
| Honduras | Subarachnoid hemorrhage | 20400  (19543, 21376) | 4.61  (0.14, 9.97) | 1607  (1585, 1625) | 2.11  (0.86, 3.36) | 1576  (1527, 1620) | -4.23  (-7.43, -1.2) | 37661  (33720, 41069) | -19.11  (-27.44, -11.73) |
| Hungary | All stroke | 154730  (147965, 161973) | 11.39  (6.52, 16.6) | 9400  (8824, 9916) | -22.32  (-27.09, -18.06) | 2528  (2303, 2799) | -48.36  (-52.96, -42.82) | 73611  (63274, 83262) | -43.88  (-51.76, -36.52) |
| Hungary | Intracerebral hemorrhage | 9268  (8379, 10246) | -24.72  (-34.84, -12.53) | 1585  (1454, 1706) | -28.87  (-37.36, -20.77) | 1114  (996, 1196) | -32.31  (-39.51, -25.34) | 20616  (16836, 23582) | -52.46  (-65.95, -40.42) |
| Hungary | Ischemic stroke | 92750  (87808, 97134) | -18.57  (-23.55, -12) | 7252  (6814, 7642) | -39.59  (-44.19, -34.57) | 1147  (1044, 1330) | -73.1  (-76.33, -67.61) | 44201  (38263, 50214) | -55.95  (-64.2, -48.18) |
| Hungary | Subarachnoid hemorrhage | 10762  (10281, 11320) | 23.75  (16.81, 29.53) | 562  (556, 568) | 0.55  (-0.96, 1.65) | 268  (263, 273) | -3.18  (-4.95, -1.06) | 8794  (8175, 9466) | -7.54  (-17.61, 0.81) |
| Iceland | All stroke | 5852  (5200, 6624) | 15.88  (2.97, 31.17) | 348  (299, 400) | 17.79  (1.41, 35.59) | 76  (69, 82) | 68.17  (53.32, 82.7) | 3518  (2595, 4252) | 131.79  (70.94, 180.09) |
| Iceland | Intracerebral hemorrhage | 555  (515, 601) | -3.21  (-13.04, 7.09) | 85  (75, 94) | 34.35  (6.2, 60.47) | 30  (26, 33) | 8.7  (-3.84, 21.74) | 1405  (1033, 1707) | 133.73  (64.2, 217.37) |
| Iceland | Ischemic stroke | 3674  (3110, 4270) | 5.82  (-15.88, 27.38) | 230  (192, 271) | -20.75  (-39.84, -0.55) | 36  (33, 38) | 32.47  (23.28, 43.39) | 1672  (1154, 2075) | 93.17  (17.06, 174.17) |
| Iceland | Subarachnoid hemorrhage | 607  (585, 631) | 3.86  (-0.67, 8.86) | 33  (32, 35) | -2.9  (-5.44, 0) | 10  (10, 11) | 14.62  (4.09, 23.98) | 442  (408, 470) | 41.18  (29.59, 52.66) |
| India | All stroke | 19280499  (18393127, 20237245) | 61.94  (54.48, 69.97) | 2120629  (1925806, 2279245) | 57.81  (43.31, 69.62) | 861061  (751042, 995882) | 4.68  (-8.69, 21.07) | 23101941  (19069349, 26865470) | -0.51  (-17.88, 15.69) |
| India | Intracerebral hemorrhage | 3083651  (2951021, 3264767) | -19.74  (-23.58, -15.14) | 503979  (452366, 547373) | -37.15  (-44.4, -31.02) | 378186  (312996, 471231) | -47.26  (-55.9, -36.61) | 9905414  (7701466, 11853046) | -52.28  (-64.43, -39.11) |
| India | Ischemic stroke | 12786611  (11940564, 13507697) | 38.8  (28.88, 47.58) | 1491577  (1353312, 1602679) | 60.42  (45.76, 74.39) | 415345  (378020, 451931) | -12.62  (-20.25, -4.91) | 11680189  (10181105, 13213849) | 0.34  (-15.96, 17.36) |
| India | Subarachnoid hemorrhage | 1605437  (1499040, 1725005) | 18.1  (9.71, 26.99) | 125073  (120128, 129193) | -1.76  (-4.92, 1.64) | 67530  (60026, 72721) | -14.66  (-23.63, -8.1) | 1516339  (1186778, 1798575) | -40.98  (-56.7, -29.94) |
| Indonesia | All stroke | 5852944  (5650320, 6085679) | 21.81  (17.6, 26.66) | 599330  (560992, 641050) | 3.28  (-3.33, 10.47) | 308960  (269218, 353307) | -21.77  (-31.83, -10.54) | 10186290  (9130334, 11251685) | -14.05  (-22.96, -5.06) |
| Indonesia | Intracerebral hemorrhage | 1116999  (1085634, 1163031) | -11.18  (-13.93, -7.27) | 194006  (180396, 210033) | -32.77  (-37.6, -26.85) | 171021  (148642, 194109) | -53  (-58.59, -46.89) | 5764232  (5234050, 6283978) | -42.03  (-47.98, -36.41) |
| Indonesia | Ischemic stroke | 3684605  (3530824, 3850961) | -9.23  (-13.46, -4.33) | 368726  (345282, 392968) | -16.75  (-22.03, -10.23) | 118619  (102354, 138804) | -35.9  (-43.92, -25.37) | 3738250  (3284590, 4208901) | -25.37  (-35.05, -14.54) |
| Indonesia | Subarachnoid hemorrhage | 431741  (409388, 466175) | 0.41  (-4.4, 7.91) | 36598  (35314, 38049) | -0.38  (-3.86, 3.71) | 19320  (18222, 20394) | -6.55  (-11.31, -0.6) | 683808  (611694, 758806) | -7.55  (-17.74, 2.71) |
| Iran  (Islamic Republic of) | All stroke | 1475070  (1430017, 1518346) | 55.07  (50.33, 59.62) | 139776  (132823, 147567) | 98.16  (88.29, 109.19) | 49409  (44068, 55154) | 77.64  (58.47, 98.32) | 1688140  (1491467, 1871237) | 100.77  (77.38, 122.55) |
| Iran  (Islamic Republic of) | Intracerebral hemorrhage | 117780  (108790, 127694) | 5.56  (-3.89, 13.99) | 20694  (18133, 23122) | 37.47  (15.5, 58.16) | 14050  (13192, 14826) | 17.82  (11.67, 26.1) | 361629  (297200, 429590) | 7.45  (-15.45, 29.65) |
| Iran  (Islamic Republic of) | Ischemic stroke | 1117025  (1083974, 1153971) | 0.4  (-2.74, 3.49) | 114204  (109869, 119487) | 12.12  (6.84, 18.07) | 33555  (29269, 38387) | -22.71  (-31.53, -13.61) | 1235784  (1113167, 1339707) | 4.46  (-5.18, 14.23) |
| Iran  (Islamic Republic of) | Subarachnoid hemorrhage | 82397  (78948, 86484) | 5.13  (0.91, 11.08) | 4879  (4821, 4958) | 1.04  (0, 2.71) | 1804  (1607, 1941) | 16.36  (6.36, 27.27) | 90727  (81100, 101940) | 62.17  (40.8, 85.57) |
| Iraq | All stroke | 741405  (700191, 777290) | 2.7  (-3.01, 7.67) | 61468  (55618, 67543) | -16.16  (-24.14, -7.88) | 34960  (30039, 40576) | -20.85  (-31.99, -8.13) | 1076183  (924981, 1222424) | -17.25  (-28.88, -6.01) |
| Iraq | Intracerebral hemorrhage | 113167  (105686, 118911) | -23.09  (-28.22, -19.29) | 12239  (10410, 14273) | -49.57  (-57.09, -41.52) | 11705  (9922, 13536) | -53.41  (-60.31, -45.23) | 460912  (393881, 519919) | -37.04  (-45.72, -29.27) |
| Iraq | Ischemic stroke | 520119  (495662, 543958) | -24.5  (-27.78, -21.39) | 46394  (42417, 50389) | -37.42  (-42.78, -32.04) | 22162  (19093, 25875) | -50.46  (-57.96, -41.73) | 571327  (492310, 652618) | -49.34  (-56.4, -42.69) |
| Iraq | Subarachnoid hemorrhage | 46251  (44129, 48219) | 2.48  (-2.12, 6.73) | 2836  (2791, 2880) | -3.72  (-5.5, -1.95) | 1093  (1024, 1165) | -16.14  (-21.26, -9.84) | 43945  (38790, 49887) | -7.68  (-18.6, 4.47) |
| Ireland | All stroke | 90728  (84143, 97468) | 79.92  (66.86, 93.29) | 5345  (4755, 5806) | 61.64  (43.81, 75.58) | 1034  (962, 1105) | 38.52  (28.84, 48.07) | 32017  (23133, 42260) | 44.02  (4.05, 90.09) |
| Ireland | Intracerebral hemorrhage | 8523  (8022, 9033) | 3.36  (-3.69, 11.26) | 1140  (1036, 1216) | 27.77  (13.55, 43.32) | 383  (355, 416) | -4.1  (-11.81, 3.37) | 7739  (4235, 11506) | 10.14  (-46.38, 52.33) |
| Ireland | Ischemic stroke | 45597  (41173, 50634) | 24.61  (10.11, 40.58) | 3677  (3207, 4048) | 10.82  (-9.05, 28) | 474  (439, 504) | 2.11  (-4.93, 8.69) | 17053  (12171, 22990) | 41.13  (-15.69, 101) |
| Ireland | Subarachnoid hemorrhage | 8989  (8550, 9525) | 4.61  (-0.12, 10.8) | 528  (512, 541) | -2.76  (-6.05, -0.42) | 177  (168, 185) | -11.32  (-14.72, -7.92) | 7226  (6726, 7764) | 22.44  (13.5, 31.92) |
| Israel | All stroke | 158273  (146270, 170595) | 8.29  (0.07, 16.72) | 9149  (8177, 10048) | 13.62  (1.54, 24.78) | 1624  (1486, 1788) | 5.78  (-3.27, 16.46) | 56634  (41650, 69999) | 11.67  (-17.88, 38.02) |
| Israel | Intracerebral hemorrhage | 14940  (13973, 16206) | 21.64  (12.85, 32.6) | 1743  (1586, 1957) | 8.63  (-4.95, 26.53) | 762  (681, 856) | -27.17  (-35.55, -18.5) | 24435  (19281, 29560) | -2.08  (-27.19, 24.84) |
| Israel | Ischemic stroke | 92629  (83640, 99705) | -5.82  (-16.56, 3.18) | 6679  (5877, 7352) | -5.26  (-20.41, 6.73) | 692  (644, 749) | 12.5  (4.89, 21.2) | 26978  (17954, 34467) | 5.54  (-37.54, 52.37) |
| Israel | Subarachnoid hemorrhage | 12423  (11889, 13012) | -1.07  (-5.44, 4.2) | 728  (714, 740) | -4.72  (-6.78, -3.39) | 170  (161, 183) | 0  (-4.92, 6.56) | 5221  (4416, 5972) | -23.83  (-40.24, -10.27) |
| Italy | All stroke | 1133197  (1051883, 1210185) | 77.13  (64.42, 89.16) | 63902  (59520, 69279) | 58.06  (47.22, 71.37) | 14581  (13415, 15903) | 24.34  (14.4, 35.62) | 433652  (377689, 488573) | 39.67  (21.65, 57.36) |
| Italy | Intracerebral hemorrhage | 79096  (73947, 84669) | -5.41  (-14.45, 2.68) | 11903  (11187, 12630) | -0.86  (-10.2, 10.75) | 6249  (5651, 6777) | -21.91  (-30.15, -13.55) | 159074  (139524, 180571) | -13.95  (-33.17, 4.98) |
| Italy | Ischemic stroke | 554147  (501186, 597633) | 7.71  (-5.78, 23.67) | 48448  (44818, 53071) | 18.98  (5.26, 32.59) | 6700  (6223, 7411) | -1.12  (-8.18, 9.11) | 217408  (184531, 247380) | 21.08  (-10.19, 46.33) |
| Italy | Subarachnoid hemorrhage | 73884  (70577, 77342) | 28.51  (21.27, 36.09) | 3551  (3515, 3579) | 2.44  (1.46, 3.57) | 1632  (1540, 1715) | 16.85  (12.92, 21.35) | 57170  (53634, 60623) | 36.04  (22.86, 46.89) |
| Jamaica | All stroke | 44716  (41706, 50085) | 84.5  (72.08, 106.65) | 3698  (3376, 4077) | 55.54  (42, 71.49) | 1747  (1330, 2158) | 24.05  (-5.56, 53.2) | 43850  (33216, 55246) | 19.11  (-9.78, 50.06) |
| Jamaica | Intracerebral hemorrhage | 6732  (5500, 9117) | 24.42  (3.79, 69.6) | 975  (861, 1095) | -22.02  (-33.5, -11.43) | 953  (692, 1248) | -28.45  (-47.57, -9.94) | 20811  (16121, 26394) | -40.15  (-58.98, -17.28) |
| Jamaica | Ischemic stroke | 24220  (22180, 27498) | 15.17  (1.57, 34.26) | 2374  (2170, 2631) | 10.3  (-2.93, 23.76) | 627  (476, 738) | -40.38  (-53.87, -30.19) | 15413  (10566, 19309) | -40.57  (-68.53, -12.29) |
| Jamaica | Subarachnoid hemorrhage | 5830  (5401, 6600) | 12.44  (3.73, 29.06) | 349  (346, 352) | -0.96  (-1.85, 0.15) | 168  (162, 171) | 1.31  (-1.13, 3.38) | 7625  (6529, 9542) | 51.45  (32.7, 92.05) |
| Japan | All stroke | 3091851  (2977710, 3186189) | 4.54  (0.68, 7.73) | 159502  (148518, 168892) | 4.69  (-2.53, 10.85) | 35062  (31077, 39077) | 5.96  (-6.1, 18.09) | 1216581  (1121984, 1301300) | 10.56  (1.96, 18.26) |
| Japan | Intracerebral hemorrhage | 382989  (364725, 397838) | -6.02  (-11.9, -0.97) | 31216  (28719, 33064) | -23.73  (-32.16, -12.87) | 14156  (12509, 15624) | -30.11  (-38.37, -21.65) | 437344  (397219, 476003) | -13.17  (-28.62, -0.7) |
| Japan | Ischemic stroke | 1444903  (1379938, 1513108) | -11.01  (-18.82, -5.04) | 104339  (97332, 110957) | -19.02  (-26.96, -12.8) | 14605  (12712, 16885) | -3.43  (-18.43, 9.79) | 524871  (481462, 559838) | -4.16  (-21.78, 9.5) |
| Japan | Subarachnoid hemorrhage | 405466  (386442, 423926) | -6.14  (-13.33, 3.61) | 23948  (22467, 24871) | -11.3  (-19.41, -5.8) | 6301  (5855, 6568) | -15.87  (-21.03, -10.71) | 254365  (243303, 265460) | -15.69  (-22.11, -9.25) |
| Jordan | All stroke | 207476  (198486, 215799) | 19.81  (14.62, 24.62) | 19118  (17889, 20272) | 30.58  (22.19, 38.47) | 5611  (5023, 6183) | 61.03  (44.15, 77.47) | 197016  (168797, 229714) | 71.33  (46.79, 99.77) |
| Jordan | Intracerebral hemorrhage | 17546  (16336, 18918) | -6.29  (-13.36, 0.97) | 2854  (2520, 3159) | 3.95  (-9.34, 16.6) | 1775  (1630, 1914) | -7.33  (-14.52, -0.29) | 57433  (47229, 69352) | 7.04  (-14.12, 32.3) |
| Jordan | Ischemic stroke | 159467  (150973, 167879) | -21.84  (-26.4, -18.12) | 15584  (14699, 16421) | -22.63  (-26.96, -17.43) | 3699  (3277, 4116) | -26.1  (-34.59, -18.32) | 132349  (115619, 151431) | -8.19  (-22.49, 6.98) |
| Jordan | Subarachnoid hemorrhage | 11297  (10696, 11916) | -5.24  (-10.86, 0.28) | 680  (670, 692) | 0.36  (-1.09, 1.81) | 137  (116, 154) | 86.27  (60.78, 111.76) | 7234  (5949, 8932) | 83.86  (48.67, 131.09) |
| Kazakhstan | All stroke | 340014  (318057, 363931) | -14.51  (-20.03, -8.49) | 28849  (26056, 31632) | -30.65  (-37.36, -23.96) | 8174  (6710, 9929) | -59.79  (-66.99, -51.15) | 473925  (406686, 532431) | -19.56  (-30.97, -9.62) |
| Kazakhstan | Intracerebral hemorrhage | 43098  (40277, 45967) | -17.33  (-22.66, -11.19) | 7264  (6367, 8216) | -38.88  (-47.31, -30.45) | 4432  (3784, 5205) | -58.49  (-63.79, -51.2) | 206446  (184332, 227924) | -30.11  (-37.98, -22.86) |
| Kazakhstan | Ischemic stroke | 258249  (242211, 273520) | -21.71  (-26.67, -16.72) | 19363  (17561, 21108) | -40.72  (-46.35, -34.12) | 2871  (2159, 3722) | -76.35  (-82.51, -69.65) | 233146  (190929, 267784) | -31.99  (-43.23, -19.67) |
| Kazakhstan | Subarachnoid hemorrhage | 24452  (23251, 25987) | -2.95  (-7.84, 3.8) | 2222  (2128, 2307) | -8.27  (-11.7, -4.51) | 871  (767, 1002) | -35.16  (-42.58, -26.94) | 34334  (31425, 36723) | -22.23  (-28.74, -16.36) |
| Kenya | All stroke | 878290  (822918, 939269) | 47.05  (37.78, 57.26) | 82994  (75431, 90270) | 43.31  (30.25, 55.88) | 27638  (23684, 32443) | 5.27  (-9.79, 23.57) | 815306  (582108, 1054664) | 3.17  (-26.34, 33.46) |
| Kenya | Intracerebral hemorrhage | 145226  (134407, 173445) | 15.76  (6.61, 36.28) | 24318  (21774, 26589) | -13.98  (-21.87, -6.42) | 16438  (13897, 20073) | -43.58  (-53.04, -31.51) | 498223  (370083, 623399) | -36.73  (-50.77, -21.36) |
| Kenya | Ischemic stroke | 572345  (526087, 613385) | 7.28  (-0.57, 15.35) | 54948  (50053, 59859) | 14.58  (5.44, 23) | 10333  (9109, 11376) | -7.83  (-17.83, 1.22) | 272832  (181650, 360213) | -6.17  (-33.14, 16.77) |
| Kenya | Subarachnoid hemorrhage | 75605  (68123, 83963) | 31.06  (18.72, 45.04) | 3727  (3605, 3822) | -5.22  (-8.7, -2.61) | 866  (678, 995) | -25.58  (-41.4, -12.09) | 44251  (30375, 71052) | -1.45  (-29.09, 53.87) |
| Kiribati | All stroke | 2851  (2618, 3015) | 2.39  (-5.98, 8.27) | 445  (407, 483) | 33.71  (22.2, 45.15) | 372  (317, 509) | 127.08  (93.85, 210.67) | 10602  (9167, 11814) | 82.32  (57.66, 103.17) |
| Kiribati | Intracerebral hemorrhage | 887  (838, 933) | -16.94  (-21.28, -12.46) | 226  (207, 246) | 4.35  (-4.57, 13.08) | 277  (249, 313) | 61.19  (44.73, 80.28) | 8469  (7443, 9356) | 54.98  (36.54, 71.33) |
| Kiribati | Ischemic stroke | 1554  (1423, 1702) | -11.83  (-19.18, -3.69) | 185  (170, 199) | 12.75  (3.32, 21.5) | 79  (56, 174) | 74.85  (24.34, 313.86) | 1449  (1090, 1706) | 0.84  (-24.13, 18.09) |
| Kiribati | Subarachnoid hemorrhage | 317  (300, 334) | -20.8  (-24.92, -16.48) | 34  (30, 38) | 1.49  (-7.9, 15.05) | 15  (12, 22) | 21.41  (-1.95, 72) | 683  (634, 752) | 22.88  (13.94, 35.28) |
| Kuwait | All stroke | 115244  (108142, 123138) | 124.08  (110.27, 139.43) | 8588  (7799, 9310) | 164.96  (140.62, 187.22) | 1890  (1643, 2136) | 247.15  (201.77, 292.29) | 84572  (72351, 97752) | 263.63  (211.08, 320.3) |
| Kuwait | Intracerebral hemorrhage | 9752  (9269, 10208) | -12.39  (-17.54, -8.3) | 1286  (1169, 1382) | -3.32  (-15.16, 11.34) | 856  (766, 952) | 58.19  (42.77, 70.52) | 24703  (20830, 28674) | 55.21  (26.77, 87.32) |
| Kuwait | Ischemic stroke | 78256  (73615, 84077) | 2.65  (-4.89, 11.94) | 6984  (6321, 7602) | 17.13  (3.58, 29.88) | 941  (795, 1082) | -16.92  (-29.61, -5.24) | 56236  (48251, 65087) | 40.55  (11.01, 74.09) |
| Kuwait | Subarachnoid hemorrhage | 6066  (5731, 6390) | -10.15  (-14.67, -4.84) | 318  (309, 326) | -2.26  (-6.02, 0.3) | 93  (82, 102) | 141.03  (115.38, 161.54) | 3632  (3270, 3991) | 60.17  (33.89, 83.37) |
| Kyrgyzstan | All stroke | 138322  (133457, 147153) | 50.91  (45.6, 60.54) | 19376  (18399, 20438) | 77.05  (68.11, 86.75) | 7822  (6624, 9248) | 53.94  (30.36, 82.01) | 308477  (279511, 337922) | 90.5  (72.61, 108.68) |
| Kyrgyzstan | Intracerebral hemorrhage | 18534  (17290, 20464) | 23.09  (14.67, 35.82) | 5315  (5014, 5671) | 39.21  (31.74, 49.26) | 3398  (2766, 4175) | 33.66  (9.36, 62.3) | 130784  (113867, 149817) | 69.05  (46.43, 92.21) |
| Kyrgyzstan | Ischemic stroke | 101263  (96578, 108433) | 25.92  (19.98, 35.31) | 13190  (12534, 13880) | 45.04  (37.13, 52.84) | 3901  (3354, 4485) | -5.61  (-17.85, 9.26) | 158867  (148168, 167561) | 34.62  (25.53, 43.66) |
| Kyrgyzstan | Subarachnoid hemorrhage | 9005  (8616, 9598) | -3.69  (-7.83, 2.59) | 872  (851, 887) | -3.06  (-5.45, -1.24) | 522  (504, 588) | -0.29  (-3.89, 11.38) | 18827  (17476, 20545) | 1.21  (-6.29, 10.4) |
| Lao People's Democratic Republic | All stroke | 148757  (142273, 157213) | 57.18  (50.33, 66.12) | 17033  (15914, 18074) | 42.77  (33.39, 51.5) | 7605  (6875, 8407) | -8.24  (-17.06, 1.43) | 257894  (229695, 288516) | 0.82  (-10.2, 12.79) |
| Lao People's Democratic Republic | Intracerebral hemorrhage | 29221  (27778, 30889) | 5.63  (0.01, 11.69) | 5743  (5380, 6170) | -18.05  (-24.04, -12.71) | 4206  (3794, 4685) | -49.25  (-54.45, -43.9) | 141675  (126048, 158754) | -38.84  (-46.4, -31.31) |
| Lao People's Democratic Republic | Ischemic stroke | 99194  (93678, 107634) | 25.16  (18.33, 36.17) | 10452  (9711, 11055) | 22.36  (12.95, 29.61) | 2921  (2615, 3233) | -27.52  (-35.11, -19.96) | 95964  (84917, 107542) | -8.9  (-20.81, 1.98) |
| Lao People's Democratic Republic | Subarachnoid hemorrhage | 10851  (10370, 11501) | 12.75  (7.42, 19.6) | 838  (823, 849) | -0.66  (-2.46, 0.66) | 478  (465, 489) | 1.96  (-0.71, 4.11) | 20255  (18730, 22220) | 19.57  (10.65, 30.79) |
| Latvia | All stroke | 19512  (16999, 21946) | -39.61  (-47.39, -32.07) | 2139  (1876, 2402) | -33.03  (-41.25, -24.78) | 484  (410, 598) | -68.08  (-72.95, -60.55) | 22295  (17422, 26774) | -40.7  (-53.66, -28.79) |
| Latvia | Intracerebral hemorrhage | 1488  (1243, 1694) | -33.18  (-49.68, -19.75) | 264  (231, 290) | -50.91  (-62.36, -39.9) | 167  (150, 184) | -59.66  (-64.04, -53.82) | 2953  (1922, 4097) | -72.71  (-83.14, -56.94) |
| Latvia | Ischemic stroke | 14904  (12737, 16964) | -50.7  (-63.34, -38.99) | 1755  (1529, 1989) | -43.92  (-54.78, -32.32) | 260  (206, 355) | -80.96  (-85.13, -74.27) | 17603  (13915, 20815) | -35.94  (-60.87, -15.77) |
| Latvia | Subarachnoid hemorrhage | 1985  (1811, 2140) | 13.52  (2.34, 26.96) | 121  (117, 123) | -8.37  (-11.98, -6.3) | 58  (54, 60) | -9.19  (-16.16, -5.57) | 1739  (1585, 1863) | -17.68  (-26.71, -10.28) |
| Lebanon | All stroke | 83795  (78519, 90011) | 38.53  (29.81, 48.81) | 7577  (7058, 8178) | 58.12  (47.27, 70.65) | 1893  (1695, 2181) | 67.74  (50.16, 93.22) | 65428  (51131, 81004) | 85.55  (45.01, 129.73) |
| Lebanon | Intracerebral hemorrhage | 5874  (5427, 6391) | -21.98  (-28.25, -14.53) | 1014  (857, 1184) | -4.01  (-23.93, 15.63) | 793  (709, 898) | 16.5  (3.34, 29.76) | 19676  (14966, 25116) | -12.58  (-36.01, 22.02) |
| Lebanon | Ischemic stroke | 63651  (58973, 68735) | 9.48  (-0.8, 19.57) | 6293  (5935, 6719) | 12.7  (4.8, 22.73) | 1007  (897, 1185) | 8.8  (-2.24, 33.33) | 41151  (32196, 50641) | 42.41  (0.4, 81.57) |
| Lebanon | Subarachnoid hemorrhage | 4730  (4398, 5123) | 0.77  (-5.75, 11.24) | 271  (266, 275) | 1.16  (-1.16, 2.97) | 93  (89, 98) | -4.62  (-10.4, 0.58) | 4601  (3969, 5247) | 29.41  (6.73, 54.26) |
| Lesotho | All stroke | 26854  (24910, 28633) | 34.55  (24.81, 43.47) | 3018  (2828, 3248) | 8.27  (1.46, 16.52) | 2167  (2001, 2354) | -7.87  (-14.89, 0.1) | 55925  (48354, 62958) | -13.99  (-25.63, -3.17) |
| Lesotho | Intracerebral hemorrhage | 4835  (4541, 5107) | 18.83  (12.21, 25.09) | 955  (905, 1022) | -25.71  (-29.45, -21.14) | 1336  (1205, 1479) | -33.17  (-39.85, -26.38) | 36166  (31633, 40504) | -33.85  (-41.49, -27.36) |
| Lesotho | Ischemic stroke | 17185  (15708, 18254) | 12.42  (3.15, 17.74) | 1953  (1818, 2114) | 3.43  (-3.14, 11.04) | 781  (749, 822) | -10.95  (-14.32, -5.96) | 17772  (14930, 20186) | -14.97  (-25.63, -6.17) |
| Lesotho | Subarachnoid hemorrhage | 1773  (1673, 1879) | 24.58  (18.38, 31.52) | 109  (105, 112) | -2.81  (-6.5, -0.53) | 50  (48, 53) | -14.42  (-18.91, -9.29) | 1988  (1792, 2268) | -1.58  (-11.56, 10.3) |
| Liberia | All stroke | 73277  (68593, 78851) | 3.21  (-3.38, 11.06) | 7048  (6392, 7601) | 13.59  (3.02, 22.5) | 4568  (4312, 4826) | 15.89  (9.39, 22.45) | 129133  (110087, 148740) | 2.25  (-12.83, 17.78) |
| Liberia | Intracerebral hemorrhage | 14988  (14011, 16175) | -11.73  (-17.41, -5.12) | 2981  (2792, 3140) | 18.29  (12.29, 24.48) | 2770  (2573, 2956) | 1.05  (-5.71, 7.88) | 86417  (75541, 98908) | 6.22  (-5.89, 17.56) |
| Liberia | Ischemic stroke | 44663  (40037, 47780) | -3.74  (-11.64, 1.59) | 3732  (3272, 4120) | 8.28  (-1.98, 18.2) | 1653  (1598, 1720) | 0.3  (-2.29, 4.59) | 36647  (29172, 43001) | -17.87  (-29.83, -6.6) |
| Liberia | Subarachnoid hemorrhage | 5276  (4986, 5580) | 2.39  (-2.29, 7.99) | 335  (328, 341) | 1.77  (0, 4.08) | 145  (141, 150) | 1.13  (-1.89, 4.53) | 6069  (5373, 6831) | 2.72  (-8.18, 14.57) |
| Libya | All stroke | 115366  (105644, 125025) | 26.64  (15.96, 37.24) | 8013  (7076, 9069) | 12.88  (-0.32, 27.76) | 2792  (2235, 3563) | -14.24  (-31.36, 9.44) | 114059  (88891, 138659) | 5.56  (-17.74, 28.32) |
| Libya | Intracerebral hemorrhage | 10560  (9790, 11521) | -22.01  (-27.57, -13.39) | 1084  (867, 1293) | -39.23  (-54.46, -24.97) | 990  (882, 1162) | -42.49  (-50.3, -31.53) | 39586  (30980, 48334) | -25.24  (-42.36, -7.07) |
| Libya | Ischemic stroke | 92991  (83108, 100444) | -1.8  (-13.03, 8.69) | 6490  (5781, 7330) | -24.89  (-34.75, -14.15) | 1664  (1223, 2251) | -57.03  (-67.73, -43.47) | 68831  (53058, 84015) | -35.69  (-51.55, -19.37) |
| Libya | Subarachnoid hemorrhage | 6920  (6545, 7357) | -5.99  (-11.36, 0.66) | 438  (428, 446) | -2.75  (-5.01, -0.97) | 138  (130, 150) | -23.26  (-29.3, -16.28) | 5641  (4853, 6310) | -16.88  (-29.46, -5.38) |
| Lithuania | All stroke | 65042  (57599, 71272) | 17.42  (3.98, 28.67) | 5639  (4877, 6317) | 6.02  (-8.31, 18.76) | 650  (581, 764) | -57.99  (-62.41, -50.58) | 28908  (24008, 35073) | -32.8  (-44.19, -18.47) |
| Lithuania | Intracerebral hemorrhage | 2791  (2583, 3007) | -19.51  (-26.33, -10.51) | 423  (373, 471) | -46.29  (-56.25, -33.59) | 242  (214, 272) | -53.97  (-60.56, -46.78) | 4771  (3448, 6597) | -57.67  (-75.86, -36.21) |
| Lithuania | Ischemic stroke | 40360  (35193, 44937) | -15.47  (-29.27, -0.02) | 5040  (4330, 5665) | 7.66  (-12.83, 29.17) | 323  (286, 405) | -77.31  (-80.28, -71.28) | 21357  (17949, 25519) | -40.09  (-56.72, -23.07) |
| Lithuania | Subarachnoid hemorrhage | 3198  (3026, 3358) | 28.95  (19.85, 37.71) | 177  (174, 180) | -5.72  (-7.07, -4.26) | 85  (81, 87) | -15.34  (-19.18, -11.23) | 2779  (2611, 2957) | -14.6  (-21.97, -7.66) |
| Luxembourg | All stroke | 11022  (10193, 12290) | 70.41  (57.58, 90) | 668  (576, 757) | 51.68  (30.93, 72.05) | 165  (149, 184) | 44.74  (31.17, 61.87) | 4616  (3072, 6104) | 41.94  (-5.55, 87.7) |
| Luxembourg | Intracerebral hemorrhage | 940  (872, 1027) | 2.19  (-7.4, 13.44) | 106  (84, 125) | -29.46  (-61.03, -2.22) | 69  (61, 79) | -17.79  (-28.21, -5.45) | 1173  (654, 1571) | -44.08  (-70.76, 6.14) |
| Luxembourg | Ischemic stroke | 6556  (5872, 7203) | 36.56  (17.98, 54.26) | 511  (442, 581) | 5.79  (-18.6, 29) | 76  (70, 84) | 2.87  (-5.53, 14.34) | 2751  (1791, 3779) | 25.91  (-34.4, 119.22) |
| Luxembourg | Subarachnoid hemorrhage | 842  (801, 880) | 7.59  (0.97, 14.12) | 51  (50, 51) | 0  (-1.69, 1.69) | 19  (18, 20) | 26.03  (16.44, 36.3) | 692  (627, 755) | 28.9  (11.16, 45.38) |
| Madagascar | All stroke | 504281  (473264, 529229) | 1.6  (-4.65, 6.63) | 52001  (48677, 56078) | -4.51  (-10.62, 2.98) | 37281  (34741, 39597) | 13.26  (5.54, 20.29) | 989730  (892642, 1087601) | -9.08  (-18, -0.09) |
| Madagascar | Intracerebral hemorrhage | 100006  (94575, 105763) | -16.35  (-20.7, -11.85) | 19256  (18048, 21031) | -20.91  (-25.48, -14.09) | 26950  (24749, 29030) | -14.11  (-21.97, -7.6) | 720085  (656389, 784926) | -25.26  (-31.23, -19) |
| Madagascar | Ischemic stroke | 336364  (316678, 353034) | -10.66  (-15.78, -6.51) | 29655  (27660, 31855) | -11.95  (-17.52, -5.68) | 8888  (8610, 9083) | -1.55  (-4.43, 0.61) | 223536  (194548, 252137) | -15.26  (-24.62, -5.96) |
| Madagascar | Subarachnoid hemorrhage | 38046  (35828, 40154) | -13.75  (-18.16, -9.08) | 3090  (2968, 3192) | -9.22  (-12.56, -6.28) | 1443  (1382, 1484) | -7.37  (-10.74, -4.84) | 46109  (41705, 50538) | -28.31  (-34.85, -21.67) |
| Malawi | All stroke | 280409  (264614, 299709) | 10.69  (4.45, 18.3) | 29850  (27874, 31859) | 14.73  (7.14, 22.46) | 20776  (19285, 21963) | 17.58  (9.14, 24.29) | 487864  (424483, 554777) | -8.42  (-20.32, 4.14) |
| Malawi | Intracerebral hemorrhage | 54580  (49697, 58611) | 18.28  (8.86, 26.12) | 11289  (10579, 11913) | 7.62  (1.08, 12.4) | 13547  (12385, 14396) | -13.33  (-20.89, -7.47) | 302497  (264859, 340545) | -33.16  (-40.27, -25.92) |
| Malawi | Ischemic stroke | 190565  (178300, 204551) | -12.04  (-16.38, -6.81) | 16945  (15705, 18302) | -16.56  (-21.67, -11.52) | 6532  (6233, 6854) | -7.25  (-11.1, -2.01) | 159936  (137362, 185741) | -18.55  (-26.1, -8.66) |
| Malawi | Subarachnoid hemorrhage | 22619  (21618, 23675) | 2.41  (-1.62, 6.89) | 1617  (1590, 1644) | -2.01  (-4.16, -0.27) | 697  (668, 713) | -4.37  (-8.47, -2.46) | 25431  (22263, 28491) | -14.24  (-23.21, -5.1) |
| Malaysia | All stroke | 724147  (674899, 780004) | 40.02  (30.5, 50.82) | 40974  (36905, 45794) | -14.28  (-22.79, -4.2) | 14171  (12011, 16933) | -28.63  (-39.5, -14.71) | 439999  (339435, 534573) | -30.14  (-46.11, -15.13) |
| Malaysia | Intracerebral hemorrhage | 115241  (108986, 122575) | -12.35  (-17.24, -5.76) | 10992  (9552, 12770) | -49.97  (-57.49, -42.16) | 8561  (7189, 10275) | -55.85  (-63.92, -47.44) | 264421  (213357, 308085) | -47.68  (-59.33, -39.47) |
| Malaysia | Ischemic stroke | 396580  (369034, 425616) | -12.5  (-19.31, -5.8) | 26305  (23751, 29284) | -37.68  (-44.74, -28.44) | 4329  (3609, 5320) | -61.45  (-67.95, -52.29) | 129046  (85177, 175065) | -52.59  (-70.62, -33.18) |
| Malaysia | Subarachnoid hemorrhage | 67581  (63457, 72751) | 23.61  (15.86, 33.64) | 3677  (3602, 3741) | -1.17  (-2.87, 0.64) | 1281  (1213, 1338) | -2.5  (-6.79, 1.07) | 46532  (40901, 51422) | -1.95  (-15.19, 11.43) |
| Maldives | All stroke | 8945  (8255, 9782) | 83.23  (69.1, 100.37) | 691  (606, 800) | 71.85  (50.82, 98.94) | 287  (244, 340) | 103.12  (72.54, 140.01) | 10808  (9013, 13138) | 118.12  (81.89, 165.14) |
| Maldives | Intracerebral hemorrhage | 1472  (1382, 1542) | -12.04  (-18.2, -6.31) | 162  (136, 196) | -47.75  (-64.31, -30.4) | 139  (113, 171) | -37.94  (-49.4, -25.9) | 5158  (4373, 6190) | -4.33  (-20.16, 15.18) |
| Maldives | Ischemic stroke | 5731  (5228, 6340) | -3.84  (-18.04, 9.32) | 483  (427, 556) | -26.84  (-45.7, -9.52) | 135  (119, 155) | -16.05  (-25.28, -4.69) | 5104  (4150, 6332) | 29.02  (-13.21, 78.13) |
| Maldives | Subarachnoid hemorrhage | 828  (787, 868) | 10.43  (4.01, 17.43) | 46  (43, 47) | -7.02  (-10.71, -3.45) | 13  (12, 14) | -11.41  (-17.39, -4.35) | 546  (490, 616) | 2.69  (-9.82, 18.99) |
| Mali | All stroke | 318449  (301822, 334604) | 0.31  (-4.92, 5.4) | 28851  (26203, 31102) | 6.51  (-3.26, 14.82) | 17172  (15960, 18261) | -3.81  (-10.6, 2.29) | 463438  (398731, 532824) | -15.74  (-27.51, -3.13) |
| Mali | Intracerebral hemorrhage | 70040  (65692, 74288) | -13.84  (-18.73, -9.24) | 11190  (10200, 11810) | -11.64  (-17.8, -6.84) | 10270  (9353, 11151) | -19.05  (-25.7, -12.83) | 272620  (232748, 312490) | -28.54  (-36.39, -19.92) |
| Mali | Ischemic stroke | 205389  (192059, 220011) | -1.63  (-6.71, 3.64) | 16239  (14600, 17844) | 8.15  (-0.49, 15.09) | 6294  (6020, 6482) | -4.29  (-8.42, -1.56) | 165606  (144475, 191280) | -12.28  (-20.18, -2.5) |
| Mali | Subarachnoid hemorrhage | 25039  (24016, 26234) | -2.27  (-5.82, 1.78) | 1421  (1403, 1447) | 0.18  (-1.09, 1.82) | 608  (587, 628) | -3.24  (-6.83, -0.36) | 25211  (21509, 29053) | -3.93  (-16.63, 7.23) |
| Malta | All stroke | 9400  (8273, 10610) | 91.39  (68.44, 116.02) | 461  (359, 557) | 34.81  (4.78, 62.68) | 107  (96, 118) | 31.19  (18.67, 45.15) | 1701  (890, 2678) | -26.71  (-61.66, 15.36) |
| Malta | Intracerebral hemorrhage | 918  (852, 1004) | 8.97  (-1.63, 19.12) | 102  (87, 118) | -27.88  (-53.56, -4.24) | 47  (41, 52) | -17.32  (-27.93, -7.45) | 454  (242, 753) | -56.75  (-79.64, -17.48) |
| Malta | Ischemic stroke | 4486  (3856, 5359) | 17.1  (-12.26, 45.12) | 323  (237, 402) | -8.74  (-43.81, 19.28) | 50  (47, 55) | -12.47  (-19.33, -3.95) | 954  (415, 1585) | -23.72  (-64.91, 56.25) |
| Malta | Subarachnoid hemorrhage | 750  (716, 790) | 14.5  (8.75, 21.62) | 36  (35, 37) | -7.83  (-10.49, -5.47) | 10  (9, 11) | 9.26  (-1.85, 20.37) | 293  (233, 340) | -25.34  (-49.15, -7.31) |
| Mauritania | All stroke | 82654  (77594, 90610) | 6.28  (-0.23, 16.51) | 6792  (6191, 7352) | 8.23  (-1.36, 17.15) | 3637  (3314, 3937) | -4.08  (-12.6, 3.87) | 109329  (93781, 128085) | -0.63  (-14.76, 16.42) |
| Mauritania | Intracerebral hemorrhage | 14720  (13951, 15731) | -9.78  (-13.99, -3.78) | 2099  (1914, 2283) | -13.21  (-19.89, -5.47) | 1846  (1642, 2051) | -26.25  (-34.33, -18.1) | 61754  (53067, 72323) | -15.69  (-27.39, -4.11) |
| Mauritania | Ischemic stroke | 54836  (51891, 58706) | -2.48  (-6.73, 3.61) | 4404  (3990, 4772) | 5.99  (-2.58, 12.78) | 1680  (1568, 1759) | -9.41  (-15.15, -5.27) | 43528  (37459, 50432) | -12.2  (-20.57, -1.1) |
| Mauritania | Subarachnoid hemorrhage | 5746  (5409, 6191) | 9.43  (3.63, 17.03) | 290  (287, 297) | 0.39  (-0.78, 2.53) | 110  (103, 127) | -1.84  (-8.76, 10.6) | 4047  (3255, 5330) | -7.73  (-23.54, 17.7) |
| Mauritius | All stroke | 23493  (20940, 26671) | 32.87  (18.43, 50.84) | 1523  (1279, 1780) | -3.72  (-19.17, 12.49) | 533  (427, 661) | -25.53  (-40.26, -7.53) | 18674  (15125, 23821) | -18.39  (-33.9, 4.11) |
| Mauritius | Intracerebral hemorrhage | 2881  (2711, 3047) | -18.11  (-23.63, -11.98) | 337  (271, 391) | -48.19  (-59.96, -37.28) | 272  (224, 327) | -45.65  (-54.28, -34.84) | 7154  (5681, 9169) | -49.1  (-61.93, -31.64) |
| Mauritius | Ischemic stroke | 16094  (14068, 18416) | 14.51  (-4.53, 34.94) | 1070  (906, 1258) | -26.92  (-43.37, -6.75) | 208  (164, 265) | -50.25  (-60.9, -37.71) | 9316  (7414, 12261) | -17.8  (-42.63, 11.95) |
| Mauritius | Subarachnoid hemorrhage | 1875  (1761, 1980) | -21.44  (-26.46, -16.66) | 117  (102, 131) | -31.3  (-39.45, -19.05) | 53  (39, 70) | -39.44  (-55.12, -27.8) | 2204  (2029, 2391) | -27.92  (-35.2, -20.84) |
| Mexico | All stroke | 1802934  (1735629, 1873016) | 35.73  (30.67, 41.01) | 118799  (105927, 131468) | 29.37  (15.36, 43.18) | 49590  (45107, 56598) | 72.24  (56.68, 96.59) | 1266622  (964462, 1612383) | 40.15  (6.72, 78.41) |
| Mexico | Intracerebral hemorrhage | 213379  (200296, 225942) | 1.15  (-5.63, 8.59) | 24126  (19932, 27855) | -15.79  (-34.33, 0.98) | 21198  (19076, 23599) | 14.82  (0.46, 33.85) | 455063  (350608, 584529) | -11.33  (-33.29, 16.37) |
| Mexico | Ischemic stroke | 1115362  (1056439, 1177611) | 6.82  (0.74, 13.35) | 78670  (70491, 86734) | -11.93  (-24.89, -1.64) | 21411  (19420, 25313) | 14.92  (3.39, 48.15) | 577734  (400199, 769314) | 7.39  (-32.71, 54.5) |
| Mexico | Subarachnoid hemorrhage | 233064  (227358, 240760) | -2.75  (-5.34, 0.6) | 16004  (15504, 16879) | 1.38  (-1.73, 8.91) | 6981  (6612, 7687) | 3.67  (-2.52, 21.56) | 233826  (213654, 258540) | 5  (-5.18, 17.63) |
| Mongolia | All stroke | 75573  (71696, 80797) | 39.65  (32.48, 49.3) | 7097  (6359, 7872) | 0.22  (-10.21, 11.15) | 2878  (2511, 3282) | -7.22  (-19.04, 5.81) | 119335  (104261, 135954) | 16.64  (1.91, 32.89) |
| Mongolia | Intracerebral hemorrhage | 14743  (13905, 15456) | -23.75  (-28.49, -19.79) | 3125  (2748, 3500) | -45.23  (-52.9, -38.85) | 1753  (1508, 2026) | -53.6  (-59.45, -46.88) | 77658  (68472, 87013) | -28.14  (-35.53, -18.45) |
| Mongolia | Ischemic stroke | 44465  (41887, 47504) | 5.27  (-1.9, 12.7) | 3458  (3124, 3835) | -5.53  (-16.51, 8.02) | 776  (706, 860) | 10.68  (0.9, 22.33) | 31568  (26482, 37622) | 26.14  (2.31, 57.21) |
| Mongolia | Subarachnoid hemorrhage | 4751  (4484, 5015) | -1.76  (-6.6, 4.38) | 515  (487, 536) | -6.44  (-10.52, -3.51) | 349  (297, 397) | -29.07  (-38.68, -21.05) | 10108  (9306, 11319) | -33.16  (-40.09, -25.55) |
| Montenegro | All stroke | 7302  (6177, 8773) | -3.93  (-18.73, 15.43) | 878  (684, 1060) | -18.58  (-36.57, -1.76) | 276  (210, 393) | -68.52  (-76.05, -55.09) | 18397  (14941, 22275) | -11.19  (-27.88, 7.53) |
| Montenegro | Intracerebral hemorrhage | 1262  (1112, 1417) | -21.82  (-33.59, -10.5) | 470  (377, 537) | -22.15  (-39.46, -2.65) | 165  (122, 252) | -77.27  (-83.11, -67.42) | 14292  (12395, 16276) | -6.85  (-19.23, 10.27) |
| Montenegro | Ischemic stroke | 4304  (3498, 5457) | -24.17  (-46.48, 1.1) | 376  (276, 490) | -39.12  (-62.62, -9.02) | 94  (72, 124) | -59.48  (-67.95, -47.96) | 3618  (2175, 5414) | -18.28  (-63.1, 36.85) |
| Montenegro | Subarachnoid hemorrhage | 412  (345, 482) | -18.43  (-36.78, -4.23) | 32  (32, 33) | 1.06  (-0.53, 2.64) | 16  (16, 17) | -0.44  (-4.89, 4.44) | 487  (371, 585) | -11.19  (-36.17, 11.88) |
| Morocco | All stroke | 643443  (621770, 662481) | 20.17  (16.12, 23.72) | 64576  (59824, 69664) | 16.11  (7.57, 25.26) | 30755  (27785, 34766) | -3.34  (-12.68, 9.27) | 970495  (832914, 1118959) | 9.69  (-5.86, 26.47) |
| Morocco | Intracerebral hemorrhage | 62781  (59679, 66756) | -7.74  (-12.3, -1.38) | 11821  (10391, 13470) | -9.95  (-21.89, 4.32) | 9248  (8303, 10869) | -34.76  (-42.46, -22.03) | 327370  (261994, 415174) | -18.6  (-33.9, -1.67) |
| Morocco | Ischemic stroke | 476801  (458562, 495180) | -8.94  (-12.7, -5.39) | 49835  (46603, 53202) | -15.9  (-21.4, -9.35) | 20147  (18282, 22441) | -37.28  (-43.02, -30.57) | 593629  (526766, 647921) | -28.11  (-36.25, -19.96) |
| Morocco | Subarachnoid hemorrhage | 41804  (40310, 43763) | 3.13  (-1.08, 8.03) | 2920  (2829, 2992) | -6.89  (-9.54, -4.77) | 1361  (1201, 1455) | -17.31  (-25.97, -11.04) | 49497  (44155, 55863) | -4.26  (-14.92, 11.2) |
| Mozambique | All stroke | 519435  (489404, 549431) | 6.85  (0.68, 13.02) | 57903  (53512, 62655) | -3.46  (-10.78, 4.47) | 43253  (38496, 47614) | -2.79  (-13.48, 7.02) | 1020304  (883508, 1170660) | -23.89  (-34.09, -12.67) |
| Mozambique | Intracerebral hemorrhage | 94574  (89344, 100381) | -13.55  (-17.53, -9.17) | 21019  (19588, 22812) | -24.66  (-28.76, -19.69) | 28049  (24642, 31446) | -25.09  (-35.71, -13.51) | 648909  (563433, 744523) | -43.81  (-50.56, -37.48) |
| Mozambique | Ischemic stroke | 351752  (331321, 376349) | -9.04  (-13.57, -4.17) | 33933  (31061, 36831) | -14.08  (-19.8, -8.37) | 13652  (12388, 14562) | -10.24  (-19.05, -3.46) | 322423  (277556, 369597) | -26.87  (-34.04, -19.27) |
| Mozambique | Subarachnoid hemorrhage | 38010  (36138, 40133) | -0.22  (-4.61, 4.61) | 2951  (2863, 3012) | -4.83  (-7.19, -2.59) | 1552  (1466, 1606) | -9.47  (-15.66, -6.19) | 48972  (42519, 56540) | -29.51  (-37.67, -20.17) |
| Myanmar | All stroke | 961136  (936627, 981700) | 37.84  (34.33, 40.79) | 117908  (111871, 125635) | 35.71  (28.76, 44.61) | 88210  (83526, 93216) | 28.48  (21.66, 35.77) | 2713590  (2568694, 2838799) | 33.06  (25.96, 39.2) |
| Myanmar | Intracerebral hemorrhage | 207103  (201008, 214286) | 11.13  (7.62, 14.91) | 49099  (45913, 53209) | -0.06  (-6.98, 7.85) | 58111  (55140, 61271) | -13.18  (-17.75, -8.32) | 1779109  (1680727, 1855761) | -2.42  (-7.97, 2.51) |
| Myanmar | Ischemic stroke | 619127  (597181, 640948) | 10.5  (6.3, 14.72) | 63099  (60302, 66634) | 4.28  (-1.1, 12.23) | 26555  (24916, 28299) | -10.49  (-16.04, -4.5) | 806495  (767948, 845735) | 6.89  (0.17, 12.25) |
| Myanmar | Subarachnoid hemorrhage | 65221  (62866, 68064) | -6.42  (-10.14, -2.35) | 5710  (5656, 5791) | -1.63  (-2.63, -0.27) | 3545  (3470, 3646) | -2.66  (-4.53, 0.31) | 127986  (120019, 137302) | -0.1  (-7.45, 7.51) |
| Namibia | All stroke | 45153  (38956, 55648) | 42.31  (22.78, 75.39) | 4064  (3664, 4424) | 13.96  (2.74, 24.04) | 1552  (1293, 1796) | -26.63  (-38.9, -15.1) | 50984  (39212, 62338) | -12.97  (-33.07, 6.41) |
| Namibia | Intracerebral hemorrhage | 6152  (5746, 6561) | 16.36  (8.96, 23.44) | 1034  (904, 1155) | -20.06  (-28.92, -11.26) | 936  (775, 1099) | -45.32  (-54.79, -35.82) | 27703  (21301, 33818) | -40.62  (-54.12, -28.88) |
| Namibia | Ischemic stroke | 28916  (26379, 33244) | 10.06  (1.1, 23.74) | 2874  (2609, 3109) | -3.77  (-11.74, 3.32) | 570  (473, 649) | -46.54  (-54.39, -39.17) | 20915  (15978, 25540) | -20.23  (-35.82, -4.09) |
| Namibia | Subarachnoid hemorrhage | 3755  (3440, 4070) | 61.39  (48.31, 74.13) | 155  (151, 159) | -1.77  (-4.51, 0.48) | 46  (45, 49) | -2.54  (-6.09, 2.03) | 2366  (1933, 2980) | 31.81  (8.17, 62.88) |
| Nepal | All stroke | 354545  (333424, 373986) | 39.9  (31.57, 47.57) | 36224  (33400, 39559) | 17.27  (8.12, 28.05) | 19404  (17258, 21526) | 1.59  (-9.66, 12.69) | 571966  (496873, 650361) | 9.29  (-5.06, 24.27) |
| Nepal | Intracerebral hemorrhage | 70376  (67005, 74361) | 1.62  (-3.35, 7.55) | 11730  (10900, 12734) | -26.89  (-32.61, -20.59) | 8829  (7359, 10410) | -37.96  (-46.89, -26.49) | 255530  (211678, 300640) | -30.91  (-43.16, -19.04) |
| Nepal | Ischemic stroke | 226056  (209255, 244851) | 29.41  (18.37, 39.26) | 21489  (19596, 23734) | 14.79  (2.68, 26.61) | 9350  (8901, 9737) | -2.36  (-6.62, 1.75) | 283752  (256420, 312916) | 24.83  (11.1, 37.91) |
| Nepal | Subarachnoid hemorrhage | 38126  (36183, 40266) | 14.35  (8.5, 21.01) | 3005  (2904, 3091) | -6.35  (-9.58, -3.52) | 1225  (998, 1380) | -27.69  (-39.81, -18.27) | 32683  (28774, 36805) | -41.06  (-49.28, -33.9) |
| Netherlands | All stroke | 342301  (330538, 355403) | 29.19  (24.75, 34.13) | 19683  (18727, 20680) | 22.19  (16.25, 28.38) | 3936  (3609, 4172) | 8.07  (-0.89, 14.54) | 135072  (118305, 150002) | 27.8  (11.94, 41.93) |
| Netherlands | Intracerebral hemorrhage | 26046  (24943, 27108) | -0.83  (-6.54, 4.11) | 3237  (3024, 3472) | 0.78  (-10.61, 13.03) | 1568  (1408, 1659) | -4.6  (-13.81, 2.76) | 40505  (33609, 45871) | 6.5  (-19.21, 24.42) |
| Netherlands | Ischemic stroke | 198806  (191203, 205516) | 10.98  (7.09, 16.07) | 15052  (14336, 15785) | 2.91  (-4.27, 10.08) | 1840  (1683, 1976) | -17.41  (-25.12, -11.56) | 74236  (65405, 82932) | 9.91  (-7.41, 30) |
| Netherlands | Subarachnoid hemorrhage | 23592  (22874, 24412) | -2.93  (-6.4, 0.7) | 1394  (1367, 1423) | -3.8  (-6.42, -1.9) | 527  (519, 537) | 0  (-1.79, 1.79) | 20331  (19291, 21198) | 25.97  (19.15, 32.76) |
| New Zealand | All stroke | 98904  (94653, 104605) | 55.55  (48.86, 64.52) | 5655  (5270, 6031) | 42.94  (33.2, 52.44) | 1184  (1094, 1251) | 27.21  (17.52, 34.45) | 38838  (32975, 44947) | 36.92  (16.25, 58.46) |
| New Zealand | Intracerebral hemorrhage | 8497  (7443, 9757) | 22.82  (3.72, 42.47) | 999  (856, 1129) | 34.78  (5.61, 68.88) | 474  (420, 504) | -0.19  (-11.8, 6.96) | 11027  (8316, 14003) | 3.9  (-27.24, 32.75) |
| New Zealand | Ischemic stroke | 52712  (50251, 55262) | 8.15  (1.6, 16.09) | 4249  (4016, 4484) | 9.16  (0.64, 19.49) | 499  (471, 529) | -0.41  (-6.12, 5.1) | 19732  (17000, 22453) | 20.5  (-2.77, 44.23) |
| New Zealand | Subarachnoid hemorrhage | 8642  (8179, 9112) | 38.96  (29.94, 47.23) | 407  (398, 418) | 1.01  (-0.72, 3.03) | 210  (202, 218) | -7.38  (-10.15, -4.31) | 8079  (7659, 8491) | 7.07  (0.34, 14.25) |
| Nicaragua | All stroke | 93626  (89751, 99615) | 49.52  (43.33, 59.09) | 7346  (6713, 8034) | 43.66  (31.28, 57.11) | 2674  (2449, 2948) | 84.01  (68.56, 102.89) | 56769  (35529, 76463) | 25.66  (-21.36, 69.25) |
| Nicaragua | Intracerebral hemorrhage | 11081  (10145, 11882) | -2.22  (-11.02, 5.76) | 1817  (1576, 2095) | -4.99  (-20.75, 8.1) | 1346  (1224, 1488) | 5.98  (-3.67, 18.16) | 21272  (10165, 30958) | -38.07  (-74.23, -5.1) |
| Nicaragua | Ischemic stroke | 56373  (51893, 60367) | 12.59  (3.25, 22.59) | 4647  (4268, 5043) | -5.25  (-14.49, 4.25) | 1040  (952, 1149) | 9.49  (-0.2, 19.7) | 24185  (15759, 32787) | -5.66  (-47.74, 34.86) |
| Nicaragua | Subarachnoid hemorrhage | 13841  (13297, 14428) | 7.28  (2.8, 12) | 882  (869, 896) | -0.9  (-2.24, 1.08) | 288  (273, 311) | 11.26  (5.3, 19.54) | 11312  (9605, 12718) | 24.31  (3.87, 40.59) |
| Niger | All stroke | 376480  (354409, 404592) | -2.21  (-7.95, 5.09) | 35748  (32716, 38177) | -2.99  (-11.22, 3.61) | 19558  (18206, 20848) | -12.53  (-18.57, -6.76) | 470889  (376394, 564252) | -28.99  (-43.24, -14.91) |
| Niger | Intracerebral hemorrhage | 87351  (82105, 94487) | -9.74  (-14.52, -3.34) | 15509  (14559, 16411) | -4.74  (-10.25, -0.68) | 12919  (11780, 13936) | -14.78  (-22.61, -7.58) | 316092  (258064, 375836) | -30.82  (-39.8, -21.11) |
| Niger | Ischemic stroke | 241868  (223557, 256898) | 1.3  (-4.96, 5.63) | 18480  (16420, 19974) | 3.22  (-3.22, 9.99) | 5931  (5742, 6183) | -3.18  (-6.43, 0.73) | 135317  (103707, 164055) | -23.7  (-36.5, -12.62) |
| Niger | Subarachnoid hemorrhage | 31758  (30062, 33577) | 6.36  (1.23, 11.4) | 1759  (1737, 1792) | 0.58  (-0.58, 2.7) | 708  (683, 729) | 0  (-2.73, 2.34) | 19480  (14623, 24361) | -28.73  (-44.62, -16.14) |
| Nigeria | All stroke | 3046345  (2856746, 3235122) | 5.49  (-1.07, 12.03) | 255799  (230638, 276715) | 6.17  (-4.28, 14.85) | 125101  (116902, 137121) | 13.22  (5.81, 24.12) | 3479135  (2747049, 4114734) | 4.74  (-17.3, 23.87) |
| Nigeria | Intracerebral hemorrhage | 540714  (515816, 570250) | -9.69  (-13.36, -5.25) | 80519  (73530, 87537) | -16.17  (-22.38, -9.72) | 66288  (60680, 75214) | -2.46  (-11.56, 9.2) | 1814560  (1376800, 2151768) | -9.99  (-27.25, 6.2) |
| Nigeria | Ischemic stroke | 2045125  (1909743, 2160842) | 1.18  (-4.18, 6.29) | 164329  (146408, 177859) | 7.05  (-1.39, 15.34) | 55443  (53075, 58120) | -0.35  (-4.87, 3.04) | 1527765  (1281903, 1785987) | 1.69  (-11.68, 14.22) |
| Nigeria | Subarachnoid hemorrhage | 238397  (220948, 261732) | 22.95  (14.78, 33.55) | 10951  (10700, 11319) | 4.69  (2.01, 8.48) | 3371  (3148, 3787) | 10.88  (4.08, 23.13) | 136810  (88346, 176979) | 8.88  (-26.32, 39.35) |
| Norway | All stroke | 114054  (108740, 119167) | 6.02  (1.08, 10.77) | 6983  (6603, 7344) | 2.95  (-2.66, 8.28) | 1219  (1137, 1306) | 30.07  (21.38, 39.4) | 42134  (34502, 49709) | 38.47  (13.39, 63.36) |
| Norway | Intracerebral hemorrhage | 8161  (7654, 8643) | -6.01  (-12.68, 1.1) | 1100  (1023, 1174) | -3.01  (-15.56, 9.03) | 506  (457, 552) | 6.09  (-4.35, 16.96) | 11883  (8712, 14807) | 6.05  (-36.29, 43.83) |
| Norway | Ischemic stroke | 65288  (61903, 68196) | -4.38  (-9.48, 1.06) | 5378  (5085, 5652) | -12.48  (-18.55, -6.86) | 538  (513, 575) | 4.55  (-0.68, 11.59) | 22679  (18659, 26848) | 11.68  (-20.7, 47.31) |
| Norway | Subarachnoid hemorrhage | 8860  (8519, 9196) | -6.53  (-10.37, -2.17) | 506  (494, 518) | -3.44  (-5.34, -2.14) | 175  (167, 179) | 0.96  (-1.91, 5.74) | 7571  (7132, 8054) | 39.52  (29.53, 47.64) |
| Oman | All stroke | 76036  (73224, 78433) | 37.76  (32.67, 42.1) | 6781  (6336, 7246) | 74.78  (63.3, 86.75) | 2274  (1923, 2658) | 107.73  (75.68, 142.74) | 83627  (77073, 89695) | 119.78  (102.56, 135.73) |
| Oman | Intracerebral hemorrhage | 7771  (7340, 8157) | -20.6  (-24.87, -16.07) | 1009  (898, 1113) | -22.6  (-32.59, -12.87) | 789  (701, 876) | -12.04  (-21.32, -2.99) | 25282  (23250, 27213) | 4.23  (-5.14, 14.09) |
| Oman | Ischemic stroke | 55403  (53757, 56920) | -10.67  (-13.7, -8.13) | 5235  (4981, 5553) | -2.95  (-8.6, 4.29) | 1409  (1149, 1701) | -34.11  (-45.25, -22.05) | 54275  (50098, 58084) | 0.45  (-8.12, 8.47) |
| Oman | Subarachnoid hemorrhage | 5463  (5298, 5653) | -18.39  (-21.17, -15.31) | 537  (456, 580) | -5.85  (-17.05, -0.2) | 77  (74, 80) | 0.81  (-3.25, 4.88) | 4070  (3725, 4399) | 35.94  (23.44, 47.73) |
| Pakistan | All stroke | 3150184  (2982643, 3323366) | 10.23  (4.37, 16.29) | 299468  (275707, 327264) | -3.32  (-10.99, 5.66) | 162853  (151105, 176711) | 10.58  (2.59, 19.98) | 4701558  (3946798, 5355293) | 3.65  (-12.99, 18.07) |
| Pakistan | Intracerebral hemorrhage | 616879  (589106, 647281) | -16.26  (-19.95, -12.34) | 89839  (82298, 96779) | -31.16  (-36.45, -26.1) | 88981  (81399, 97780) | -10.24  (-18.2, -1.43) | 2634930  (2157624, 2985900) | -10.14  (-24.02, 2.48) |
| Pakistan | Ischemic stroke | 2049692  (1911592, 2167623) | 6.28  (-0.25, 12.09) | 184311  (169074, 203878) | 2.54  (-4.85, 12.76) | 64032  (60921, 67806) | -0.07  (-4.98, 5.52) | 1742828  (1509853, 1995943) | 1.52  (-11.08, 15.3) |
| Pakistan | Subarachnoid hemorrhage | 272310  (259783, 287140) | -9.6  (-13.51, -4.65) | 25318  (24335, 26607) | -13.86  (-17.03, -9.71) | 9840  (8785, 11125) | -26.5  (-34.59, -15.98) | 323801  (279320, 373450) | -34.72  (-43.75, -25.63) |
| Panama | All stroke | 62907  (57497, 66693) | 20.82  (10.43, 28.09) | 4691  (4147, 5184) | 24.28  (9.86, 37.32) | 1210  (1084, 1364) | -21.32  (-29.46, -11.24) | 38419  (31263, 46620) | -11.74  (-28.18, 7.1) |
| Panama | Intracerebral hemorrhage | 6859  (6475, 7255) | -27.61  (-32.25, -22.11) | 810  (717, 902) | -43.7  (-52.79, -34.19) | 578  (527, 642) | -47.64  (-53.33, -40.62) | 13999  (11643, 16904) | -52.38  (-62.79, -40.58) |
| Panama | Ischemic stroke | 36742  (33602, 40000) | 3.74  (-6.46, 13.69) | 3220  (2798, 3603) | 1.59  (-14.49, 16.61) | 416  (364, 479) | -51.44  (-57.13, -44.27) | 17841  (13462, 22726) | -8.64  (-32.51, 22.48) |
| Panama | Subarachnoid hemorrhage | 9441  (9150, 9685) | -10.4  (-13.32, -8.05) | 662  (632, 679) | -6.15  (-9.26, -3.64) | 216  (193, 243) | -29.44  (-34.81, -21.48) | 6579  (6157, 6989) | -34.39  (-39.06, -30.04) |
| Papua New Guinea | All stroke | 143642  (137601, 150349) | 57.85  (51.21, 65.22) | 15443  (14299, 16489) | 55.16  (43.67, 65.68) | 12745  (11884, 13627) | 28.86  (20.15, 37.78) | 367928  (335519, 398894) | 17.8  (7.42, 27.71) |
| Papua New Guinea | Intracerebral hemorrhage | 30735  (29286, 32369) | 19.34  (13.89, 25.43) | 7149  (6637, 7548) | 7.74  (0.33, 13.45) | 9616  (8964, 10193) | -19  (-24.48, -14.47) | 265272  (246209, 284397) | -19.18  (-24.56, -13.66) |
| Papua New Guinea | Ischemic stroke | 86237  (80598, 90953) | 33.17  (25.07, 40.68) | 7174  (6560, 7805) | 23.63  (13.36, 33.82) | 2324  (2163, 2588) | -2.53  (-9.77, 8.21) | 77914  (66608, 87842) | 20.81  (5.53, 35.1) |
| Papua New Guinea | Subarachnoid hemorrhage | 14819  (14207, 15494) | -9.35  (-12.98, -5.24) | 1120  (1102, 1136) | -2.39  (-3.91, -1.05) | 804  (757, 845) | -10.22  (-15.33, -5.84) | 24742  (22702, 26656) | -24.15  (-30.47, -18.23) |
| Paraguay | All stroke | 87395  (83352, 91419) | 21.43  (15.82, 27.03) | 6809  (6101, 7466) | 0.39  (-10.05, 10.08) | 2889  (2497, 3379) | -14.19  (-25.84, 0.34) | 82831  (64410, 100960) | -12.56  (-32.01, 6.58) |
| Paraguay | Intracerebral hemorrhage | 10482  (9571, 11466) | -12.57  (-21, -3.33) | 1565  (1317, 1791) | -30.94  (-43.29, -19.35) | 1324  (1150, 1541) | -45.11  (-52.66, -36.11) | 36634  (29014, 43804) | -43.85  (-57.93, -32.31) |
| Paraguay | Ischemic stroke | 52113  (48979, 55258) | -9.35  (-15.18, -3.15) | 4278  (3846, 4692) | -29.46  (-37.56, -20.77) | 1107  (911, 1356) | -46.88  (-56.22, -33.94) | 31861  (21897, 41829) | -35.67  (-59.8, -13.47) |
| Paraguay | Subarachnoid hemorrhage | 12263  (11954, 12695) | 2.25  (-1.11, 5.59) | 965  (938, 983) | -3.42  (-5.9, -1.6) | 459  (435, 482) | -9.88  (-13.83, -5.78) | 14336  (13499, 15328) | -14.42  (-19.5, -8.79) |
| Peru | All stroke | 404493  (372276, 442165) | 12.4  (3.45, 22.87) | 30157  (26659, 34307) | 30.24  (15.14, 48.17) | 10570  (9281, 11701) | 15.56  (1.49, 27.95) | 292537  (202786, 389013) | -1.5  (-31.72, 30.98) |
| Peru | Intracerebral hemorrhage | 50271  (46151, 54408) | -14.51  (-21.77, -6.95) | 7211  (6114, 8297) | 0.78  (-19.57, 21.52) | 4510  (4051, 5047) | -27.81  (-35.47, -19.05) | 113660  (81566, 157147) | -37.17  (-58.07, -14.51) |
| Peru | Ischemic stroke | 249982  (229010, 276261) | 2.2  (-7.54, 14.51) | 18413  (16114, 21397) | -0.86  (-17.08, 14.04) | 4031  (3290, 4569) | -9.22  (-25.36, 2.67) | 116474  (62738, 164449) | 5.5  (-38.49, 55.44) |
| Peru | Subarachnoid hemorrhage | 67492  (66021, 69054) | -16.22  (-18.24, -14.21) | 4532  (4430, 4613) | -3.09  (-4.94, -1.54) | 2030  (1941, 2086) | -3.36  (-7.31, -0.79) | 62402  (58482, 67418) | -16.98  (-22.35, -10.61) |
| Philippines | All stroke | 1973078  (1907887, 2054045) | 25.07  (20.94, 30.2) | 189688  (171561, 205175) | 9.44  (-1.02, 18.37) | 102775  (91980, 115485) | 1.08  (-9.54, 13.57) | 3539287  (3134829, 4004676) | 8.62  (-3.79, 22.9) |
| Philippines | Intracerebral hemorrhage | 386840  (366947, 413533) | -7.63  (-12.56, -1.21) | 70327  (61828, 75801) | -22.71  (-31.64, -15.78) | 60223  (52600, 68200) | -35.28  (-43.31, -26.81) | 2123021  (1893613, 2363313) | -22.7  (-31.03, -14.3) |
| Philippines | Ischemic stroke | 1222470  (1157266, 1284796) | 1.34  (-4.28, 6.81) | 105424  (96009, 114964) | -9.03  (-18.22, 0.19) | 35999  (33157, 39845) | -10.22  (-17.32, -0.81) | 1075565  (926907, 1268244) | -10.84  (-25.34, 7.16) |
| Philippines | Subarachnoid hemorrhage | 190017  (179281, 203351) | 12.13  (5.5, 20.37) | 13937  (13724, 14410) | 0.91  (-0.64, 4.55) | 6553  (6222, 7440) | 9.45  (3.74, 25.71) | 340700  (314309, 373119) | 45.33  (33.5, 59.36) |
| Poland | All stroke | 519057  (484691, 557707) | 35.15  (26.2, 45.21) | 30947  (28024, 33936) | -15.5  (-23.49, -7.34) | 8864  (8180, 9786) | -40.56  (-45.16, -34.38) | 241271  (196650, 278644) | -41.26  (-52.12, -32.16) |
| Poland | Intracerebral hemorrhage | 34631  (31722, 37594) | -15.53  (-25.44, -4.12) | 5120  (4562, 5642) | -38.36  (-49.78, -29.05) | 3639  (3371, 4056) | -41.3  (-46.63, -33.99) | 85537  (69756, 99619) | -44.83  (-58.75, -29.74) |
| Poland | Ischemic stroke | 279125  (255117, 299120) | -14.27  (-22.45, -5.19) | 23569  (21233, 26004) | -36.65  (-43.86, -27.66) | 4041  (3648, 4524) | -69.68  (-73.27, -66.17) | 118518  (91844, 140280) | -65  (-78.94, -54.28) |
| Poland | Subarachnoid hemorrhage | 41586  (40450, 42663) | 19.3  (15.05, 22.47) | 2258  (2229, 2290) | -0.5  (-2.12, 0.5) | 1184  (1161, 1206) | -5.39  (-8.38, -2.4) | 37216  (35050, 38745) | -14.34  (-21.24, -7.98) |
| Portugal | All stroke | 159344  (148160, 169610) | 42.26  (32.27, 51.42) | 10338  (9040, 11270) | 28.91  (12.73, 40.54) | 3146  (2899, 3414) | -9.55  (-16.67, -1.86) | 83029  (63437, 99323) | -6.44  (-28.52, 11.92) |
| Portugal | Intracerebral hemorrhage | 16871  (15211, 18348) | 8.87  (-3.76, 19.9) | 2341  (2131, 2595) | -6.21  (-20.27, 10.98) | 1475  (1372, 1593) | -29.75  (-35.44, -23.34) | 41798  (34719, 48605) | -18.6  (-42.61, 4) |
| Portugal | Ischemic stroke | 83783  (75651, 90675) | -2.23  (-16.68, 9.29) | 7298  (6225, 7969) | -0.68  (-19.89, 14.87) | 1273  (1143, 1417) | -34.72  (-41.35, -28.19) | 28971  (17116, 37847) | -41.21  (-70.69, -10.36) |
| Portugal | Subarachnoid hemorrhage | 12523  (11758, 13146) | 12.66  (5.47, 17.46) | 700  (684, 706) | -0.98  (-2.1, 0.14) | 398  (384, 404) | -7.4  (-9.76, -5.62) | 12259  (11603, 12871) | -4.12  (-10.64, 2.4) |
| Qatar | All stroke | 42529  (39366, 46181) | 63.09  (50.96, 77.1) | 2844  (2480, 3159) | 85.15  (61.45, 105.66) | 409  (365, 453) | 81.37  (61.72, 100.76) | 16095  (10732, 21375) | 45.63  (-2.9, 93.4) |
| Qatar | Intracerebral hemorrhage | 5954  (5547, 6451) | -14.9  (-21.55, -8.94) | 552  (485, 631) | -20.38  (-33.44, -6.85) | 194  (169, 219) | -42.84  (-49.19, -34.88) | 6829  (4771, 8925) | -36.9  (-59, -10.21) |
| Qatar | Ischemic stroke | 27850  (26009, 30004) | -15.4  (-22.08, -6.64) | 2070  (1781, 2298) | -3.18  (-18.54, 8.34) | 169  (152, 185) | -61.12  (-64.17, -57.54) | 6725  (3797, 9609) | -51.87  (-76.3, -23.82) |
| Qatar | Subarachnoid hemorrhage | 3371  (3206, 3513) | -19.06  (-22.93, -14.76) | 222  (214, 230) | -11.38  (-15.29, -8.04) | 46  (44, 49) | -31.5  (-35.5, -27) | 2541  (2164, 2841) | -0.11  (-17.32, 12.13) |
| Republic of Korea | All stroke | 1497705  (1422679, 1569585) | 51.73  (44.13, 59.01) | 100379  (91731, 107529) | 55.76  (42.35, 66.86) | 16369  (14119, 18374) | 26.78  (9.35, 42.32) | 1036495  (920814, 1212248) | 133.78  (107.69, 173.42) |
| Republic of Korea | Intracerebral hemorrhage | 227894  (215193, 239612) | 1.36  (-5.78, 8.62) | 18993  (17038, 21296) | -1.93  (-13.67, 13.24) | 6724  (5701, 7670) | -27.35  (-41.18, -15.93) | 354458  (317652, 396508) | 39.45  (17.19, 67.34) |
| Republic of Korea | Ischemic stroke | 934339  (880800, 990704) | 1.46  (-5.1, 8.9) | 73744  (67341, 78369) | 6.57  (-2.35, 17.29) | 7225  (6177, 8212) | -32.66  (-43.02, -22.73) | 569583  (497015, 694352) | 59.82  (28.5, 93.44) |
| Republic of Korea | Subarachnoid hemorrhage | 123109  (113472, 131615) | 1.72  (-4.22, 10.35) | 7642  (7352, 7864) | -4.93  (-8.24, -2.01) | 2420  (2241, 2493) | -4.88  (-9.21, -1.63) | 112455  (106147, 121388) | 20.33  (10.95, 30.39) |
| Republic of Moldova | All stroke | 43079  (40147, 46231) | 4.63  (-2.5, 12.28) | 6578  (6253, 6978) | 29.66  (23.24, 37.54) | 2380  (2088, 2804) | -11.96  (-22.76, 3.73) | 95796  (85233, 106759) | 32.8  (18.16, 48) |
| Republic of Moldova | Intracerebral hemorrhage | 4962  (4661, 5346) | 3.12  (-4.28, 11.41) | 1736  (1575, 1914) | 38.83  (20.57, 57.57) | 1037  (926, 1223) | -16.86  (-24.46, -6.17) | 37747  (32601, 43313) | 12.23  (-8.77, 32.6) |
| Republic of Moldova | Ischemic stroke | 32262  (30122, 34807) | -8  (-16.21, 0.58) | 4655  (4494, 4874) | 13.67  (6.48, 21.19) | 1255  (1076, 1490) | -25.52  (-36.33, -13.68) | 53532  (48602, 58378) | 25.83  (9.96, 46.92) |
| Republic of Moldova | Subarachnoid hemorrhage | 2146  (2038, 2252) | -5.72  (-11.04, 0.18) | 187  (183, 190) | -2.05  (-4.19, 0.09) | 88  (86, 92) | 6.49  (3.12, 11.69) | 4517  (4030, 5069) | 72.37  (49.94, 96.91) |
| Romania | All stroke | 386504  (370083, 406282) | 20.1  (15, 26.25) | 27436  (25164, 29586) | -21.41  (-27.92, -15.26) | 6749  (5921, 8092) | -65.22  (-69.48, -58.3) | 311386  (282981, 346725) | -35.65  (-41.52, -28.34) |
| Romania | Intracerebral hemorrhage | 24561  (23142, 26282) | -13.08  (-18.65, -5.93) | 4798  (4336, 5207) | -39.18  (-47.1, -32.24) | 2972  (2703, 3323) | -65.66  (-69.02, -61.97) | 136197  (125381, 150087) | -36.21  (-45.37, -29) |
| Romania | Ischemic stroke | 237940  (223951, 252295) | -6.5  (-13.66, 3.96) | 20883  (19187, 22556) | -34.07  (-40.2, -27.88) | 2956  (2499, 3885) | -78.73  (-82.25, -72.98) | 148031  (132164, 167646) | -55.21  (-62.56, -46.64) |
| Romania | Subarachnoid hemorrhage | 21613  (20716, 22459) | 20.95  (16.27, 26.51) | 1756  (1641, 1822) | -6.82  (-10.85, -4.23) | 820  (720, 883) | -25.83  (-31.94, -20.94) | 27158  (25436, 28992) | -21.7  (-27.25, -14.62) |
| Russian Federation | All stroke | 2553318  (2368521, 2688176) | 14.82  (6.51, 20.89) | 261178  (240171, 282179) | -0.34  (-8.35, 7.68) | 114432  (95378, 139788) | -18.73  (-32.26, -0.72) | 4329782  (3879109, 4877870) | 12.59  (0.87, 26.84) |
| Russian Federation | Intracerebral hemorrhage | 224008  (211856, 235291) | -5.52  (-10.93, -0.48) | 49142  (43570, 54524) | -15.01  (-26.68, -4.47) | 41972  (38088, 45370) | -15.95  (-23.35, -9.69) | 1247034  (1144228, 1362912) | -8.43  (-16.45, -0.11) |
| Russian Federation | Ischemic stroke | 1802588  (1713575, 1883018) | -11.09  (-15.53, -6.66) | 196242  (181446, 211133) | -23.21  (-31.44, -16.84) | 64927  (50405, 86171) | -46.68  (-57.79, -32.31) | 2771314  (2441084, 3183385) | -13.06  (-25.53, -1.6) |
| Russian Federation | Subarachnoid hemorrhage | 158988  (152934, 167115) | -8.57  (-12.68, -4.08) | 15794  (15155, 16521) | -0.79  (-4.5, 5.68) | 7532  (6884, 8247) | -22.32  (-29.13, -16.81) | 311434  (293798, 331573) | -9.38  (-15.92, -1.15) |
| Rwanda | All stroke | 215481  (202268, 226336) | 29.14  (21.23, 35.65) | 22279  (20668, 23807) | 24.83  (15.81, 33.4) | 10596  (9470, 12075) | 7.21  (-4.17, 22.18) | 299204  (239244, 358538) | 2.36  (-18.15, 22.66) |
| Rwanda | Intracerebral hemorrhage | 38621  (36314, 41113) | 7.75  (1.44, 14.15) | 7043  (6385, 7614) | -19.48  (-26.3, -13.15) | 6775  (5949, 7955) | -35.48  (-43.53, -24.44) | 161503  (129491, 195737) | -43.74  (-54.38, -32.66) |
| Rwanda | Ischemic stroke | 143148  (133376, 152316) | 5.75  (-0.58, 12.07) | 14074  (13159, 14997) | 10.89  (4.3, 17.55) | 3431  (3159, 3716) | 1.95  (-5.84, 10.09) | 121599  (95916, 144276) | 33.76  (9.49, 56.61) |
| Rwanda | Subarachnoid hemorrhage | 17582  (16574, 18785) | 3.29  (-2.6, 9.66) | 1162  (1125, 1197) | -5.84  (-8.94, -2.98) | 390  (362, 405) | -4.9  (-12.24, -1.05) | 16101  (13837, 18526) | 0.94  (-12.82, 15.38) |
| Samoa | All stroke | 5047  (4520, 6041) | 30.46  (16.85, 56.16) | 499  (437, 573) | 30.56  (14.56, 49.92) | 257  (216, 340) | 11.72  (-6.12, 48.08) | 9125  (6919, 11481) | 25.26  (-5.02, 57.6) |
| Samoa | Intracerebral hemorrhage | 1225  (1070, 1606) | 23.56  (7.98, 61.82) | 232  (199, 272) | 27.5  (9.9, 49.69) | 182  (151, 250) | -9.51  (-25.09, 23.93) | 6805  (5400, 8296) | 16.32  (-7.52, 41.77) |
| Samoa | Ischemic stroke | 3217  (2816, 4016) | 34.6  (18.1, 67.66) | 230  (203, 263) | 13.05  (-0.41, 28.78) | 58  (49, 72) | 14.11  (-2, 42.49) | 1367  (748, 1926) | -12.26  (-52.66, 22.78) |
| Samoa | Subarachnoid hemorrhage | 484  (449, 539) | -3.28  (-10.42, 7.8) | 36  (35, 37) | -2.59  (-4.7, -0.54) | 17  (16, 19) | -1.18  (-7.5, 9.74) | 953  (772, 1259) | 38.14  (11.77, 83.31) |
| Saudi Arabia | All stroke | 767665  (722932, 819321) | 98.72  (87.14, 112.09) | 50622  (46758, 54716) | 41.2  (30.42, 52.62) | 12392  (10804, 14409) | -22.59  (-32.52, -10) | 794939  (704018, 888600) | 34.57  (19.18, 50.43) |
| Saudi Arabia | Intracerebral hemorrhage | 74552  (70857, 79271) | -23.45  (-28.25, -18.63) | 10112  (8913, 11455) | -44.37  (-53.74, -36.07) | 6833  (5870, 7996) | -59.94  (-66.41, -53.46) | 330937  (301713, 360566) | -31.34  (-36.79, -24.94) |
| Saudi Arabia | Ischemic stroke | 476066  (447195, 503468) | -11.99  (-17.23, -6.1) | 38012  (35387, 40721) | -33.22  (-39.53, -27.02) | 4695  (4147, 5498) | -82.49  (-85.27, -78.88) | 431923  (374200, 492854) | -38.12  (-45.29, -30.49) |
| Saudi Arabia | Subarachnoid hemorrhage | 43625  (41105, 46039) | 9.91  (4.25, 16.63) | 2498  (2457, 2540) | 2.5  (0.71, 3.92) | 865  (787, 916) | 5.26  (-2.63, 10.53) | 32078  (28104, 35179) | -9.48  (-21.42, 2.83) |
| Senegal | All stroke | 333988  (320721, 348365) | 13.65  (9.13, 18.54) | 27717  (26208, 29635) | 15.07  (8.81, 23.04) | 19273  (17589, 21068) | 19.44  (9, 30.56) | 492541  (434220, 555179) | 3.27  (-8.96, 16.4) |
| Senegal | Intracerebral hemorrhage | 52568  (50201, 55104) | -23.32  (-26.47, -19.66) | 7850  (7334, 8539) | -26.62  (-31.31, -20.69) | 10717  (9779, 11782) | -14.49  (-22.65, -6.14) | 255585  (228673, 281221) | -28.75  (-35.4, -21.26) |
| Senegal | Ischemic stroke | 225293  (213405, 235298) | -3.84  (-8.43, -0.05) | 18770  (17796, 19976) | 8.88  (3.93, 15.53) | 8096  (7389, 8792) | -13.03  (-21.03, -5.11) | 221875  (193938, 255584) | -9.01  (-19.78, 3.32) |
| Senegal | Subarachnoid hemorrhage | 22128  (20550, 23421) | 10.24  (3.01, 16.29) | 1096  (1079, 1119) | 0.57  (-1.14, 2.65) | 460  (421, 494) | -18.88  (-25.87, -11.89) | 15080  (11609, 18374) | -30.2  (-45.72, -15.56) |
| Serbia | All stroke | 128734  (122692, 136964) | 10.03  (4.87, 17.07) | 11464  (10473, 12348) | -11.38  (-19.05, -4.55) | 4966  (4118, 6065) | -47.92  (-56.81, -36.38) | 187166  (159515, 213069) | -16.43  (-28.78, -4.87) |
| Serbia | Intracerebral hemorrhage | 8788  (8129, 9533) | -6.92  (-15.41, 3.7) | 1928  (1632, 2178) | -20.19  (-33.42, -7.63) | 1564  (1390, 1789) | -45.5  (-52.67, -39.38) | 56019  (46477, 62366) | -15.7  (-29.77, 0.68) |
| Serbia | Ischemic stroke | 80353  (75337, 86220) | -16.28  (-24.57, -7) | 8862  (8218, 9468) | -29.41  (-35.73, -23.16) | 3074  (2451, 3883) | -63.64  (-70.04, -55.37) | 118579  (101580, 137143) | -34.04  (-47.57, -20.56) |
| Serbia | Subarachnoid hemorrhage | 6442  (6177, 6761) | -16.84  (-21.05, -12.71) | 673  (623, 702) | -8.45  (-13.18, -5.35) | 328  (278, 393) | -30.25  (-38.28, -21.8) | 12568  (11457, 13560) | -13.28  (-22.46, -5.01) |
| Seychelles | All stroke | 2596  (2292, 2983) | 42.75  (26, 64) | 194  (162, 229) | 20.31  (0.18, 41.94) | 80  (66, 100) | 10.16  (-9.12, 36.81) | 3901  (3161, 4729) | 61.4  (30.79, 95.65) |
| Seychelles | Intracerebral hemorrhage | 370  (341, 407) | -12.22  (-19.46, -3.65) | 54  (47, 61) | -24.82  (-36.95, -11.48) | 40  (34, 49) | -37.25  (-45.23, -27.29) | 1850  (1537, 2214) | 2.48  (-15.66, 23.91) |
| Seychelles | Ischemic stroke | 1691  (1466, 1950) | -3.99  (-23.96, 13.49) | 131  (106, 157) | -20.34  (-45.38, 3.38) | 37  (29, 47) | -23.24  (-38.11, -5.08) | 1912  (1506, 2349) | 40.22  (4.8, 84.76) |
| Seychelles | Subarachnoid hemorrhage | 169  (158, 182) | -6.95  (-14.34, 0.15) | 9  (9, 10) | -12.25  (-18.32, -8.2) | 3  (3, 4) | -8.63  (-21.18, 4.71) | 139  (118, 166) | -11.02  (-27.56, 7.22) |
| Sierra Leone | All stroke | 157893  (153682, 164732) | 16.08  (12.98, 21.11) | 13358  (12701, 13976) | 14.81  (9.17, 20.13) | 9181  (8807, 9658) | 22.32  (17.34, 28.68) | 256162  (231627, 278681) | 11.02  (0.39, 20.78) |
| Sierra Leone | Intracerebral hemorrhage | 27631  (26503, 28694) | -16.08  (-19.44, -13.35) | 4650  (4400, 4898) | -7.72  (-12.15, -2.75) | 5191  (4963, 5477) | -3.35  (-7.31, 2.64) | 150746  (137266, 160936) | -4.96  (-12.25, 1.14) |
| Sierra Leone | Ischemic stroke | 103164  (99499, 108083) | 3.07  (-0.42, 6.83) | 8187  (7788, 8551) | 9.22  (4.28, 13.86) | 3749  (3614, 3934) | -6.02  (-9.85, -1.44) | 97031  (87138, 108196) | -7.54  (-16.52, 1.56) |
| Sierra Leone | Subarachnoid hemorrhage | 9237  (8881, 9678) | 2.81  (-0.8, 7.19) | 520  (513, 527) | 0.72  (-0.72, 1.97) | 240  (231, 248) | -2.47  (-6.36, 0.35) | 8385  (7223, 9548) | -14.33  (-25.14, -3.25) |
| Singapore | All stroke | 147025  (141503, 155913) | 68.56  (62.23, 78.75) | 9042  (8261, 9741) | 65.72  (51.41, 78.53) | 1366  (1238, 1504) | 101.83  (82.94, 122.3) | 66729  (53712, 81652) | 122.68  (79.24, 172.48) |
| Singapore | Intracerebral hemorrhage | 22055  (20876, 22908) | 4.5  (-0.69, 9.7) | 1680  (1504, 1872) | -6.78  (-23.51, 9.09) | 558  (495, 618) | -19.51  (-29.97, -8.36) | 25054  (19750, 31053) | 35.82  (1.88, 71.64) |
| Singapore | Ischemic stroke | 82457  (76315, 87120) | 8.51  (0.1, 15.22) | 6543  (5957, 7027) | 11.38  (0.28, 20.97) | 618  (567, 677) | 63.4  (49.06, 78.87) | 33755  (26787, 41907) | 81.91  (33.08, 121.85) |
| Singapore | Subarachnoid hemorrhage | 13425  (12666, 14105) | -4.81  (-8.43, 0.6) | 819  (801, 842) | 5.26  (2.91, 6.88) | 189  (176, 209) | 24.22  (16.77, 35.4) | 7920  (7174, 8692) | 16.83  (5.37, 28.33) |
| Slovakia | All stroke | 129524  (124195, 135681) | 30.28  (24.92, 36.47) | 8347  (7830, 8915) | 1.07  (-5.18, 7.96) | 1659  (1513, 1890) | -41.79  (-46.9, -33.65) | 60673  (53644, 67956) | -23.22  (-32.12, -14.01) |
| Slovakia | Intracerebral hemorrhage | 6627  (6150, 7042) | -2.01  (-10.64, 5.51) | 948  (876, 1005) | -33.99  (-42.31, -27.65) | 721  (677, 784) | -42.55  (-47.58, -37.08) | 19374  (17462, 22145) | -27.55  (-39.8, -14.96) |
| Slovakia | Ischemic stroke | 71670  (66942, 75549) | -20.69  (-27.32, -13.47) | 7089  (6648, 7597) | -21.76  (-28.41, -12.12) | 781  (684, 946) | -70.08  (-74.46, -63.57) | 36731  (31910, 40890) | -45.81  (-55.36, -36.7) |
| Slovakia | Subarachnoid hemorrhage | 6954  (6649, 7292) | 31.85  (26.58, 40.24) | 310  (307, 313) | -0.61  (-1.82, 0.45) | 156  (152, 160) | -2.3  (-5.36, 0.38) | 4568  (4272, 4921) | -16.08  (-23.02, -9.49) |
| Slovenia | All stroke | 40661  (37037, 44467) | 74.71  (59.14, 91.07) | 2807  (2530, 3122) | 54.67  (39.4, 71.98) | 550  (494, 612) | 8.41  (-2.63, 20.66) | 23334  (19717, 27254) | 71.35  (44.78, 100.13) |
| Slovenia | Intracerebral hemorrhage | 2381  (2024, 2706) | 3.59  (-18.9, 22.92) | 436  (400, 475) | 12.3  (-8.35, 29.79) | 212  (187, 230) | -2.56  (-13.37, 8.97) | 7954  (6839, 9189) | 92.4  (44.18, 156.57) |
| Slovenia | Ischemic stroke | 21726  (20043, 23561) | 23.64  (9.13, 39.6) | 2247  (2008, 2521) | 24.27  (3.76, 53.32) | 284  (256, 324) | -34.91  (-41.99, -24.58) | 13559  (11193, 16070) | 33.7  (-10.47, 78.01) |
| Slovenia | Subarachnoid hemorrhage | 2944  (2787, 3152) | 35.31  (25.9, 47.42) | 124  (123, 126) | -0.16  (-1.1, 0.94) | 54  (51, 58) | 32.14  (21.43, 45.71) | 1821  (1684, 1995) | 33.04  (19.08, 48.88) |
| Solomon Islands | All stroke | 14844  (13648, 16611) | -1.6  (-9.53, 10.11) | 1842  (1661, 2096) | -14.98  (-23.35, -3.26) | 1290  (1200, 1385) | 24.17  (15.53, 33.35) | 32342  (28228, 36686) | -4.67  (-16.79, 8.14) |
| Solomon Islands | Intracerebral hemorrhage | 3780  (3542, 4044) | -31.14  (-35.55, -26.88) | 902  (823, 1014) | -37.45  (-42.71, -30.1) | 996  (931, 1057) | -6.34  (-12.48, -1.26) | 24310  (22045, 26704) | -21.5  (-28.55, -14.34) |
| Solomon Islands | Ischemic stroke | 7454  (6634, 8223) | -15.63  (-24.43, -7.95) | 777  (686, 902) | -14.79  (-23.72, -2.47) | 221  (200, 251) | 7.93  (-2.04, 22.27) | 5751  (4092, 7481) | -4.35  (-29.55, 20.09) |
| Solomon Islands | Subarachnoid hemorrhage | 1391  (1319, 1478) | -33.24  (-36.41, -29) | 164  (152, 181) | -18.34  (-24.38, -10.19) | 73  (69, 77) | -12.14  (-16.86, -7.71) | 2280  (2091, 2501) | -27.82  (-33.43, -21.11) |
| South Africa | All stroke | 1110967  (1055674, 1150637) | 32.64  (26.03, 37.37) | 97621  (91266, 103559) | 34.63  (25.87, 42.82) | 41869  (35053, 47601) | 16.99  (-2.06, 33.01) | 1339407  (1196519, 1471783) | 26.24  (12.78, 38.72) |
| South Africa | Intracerebral hemorrhage | 132451  (127338, 137065) | 16.49  (11.74, 20.75) | 22228  (20619, 23845) | -0.26  (-7.35, 7.92) | 24184  (19949, 27311) | -21.75  (-34.33, -13.42) | 746519  (691342, 808580) | -11.87  (-19.07, -4.29) |
| South Africa | Ischemic stroke | 730168  (699028, 760456) | -6.35  (-10.37, -2.55) | 72165  (67503, 76385) | -5.26  (-11.63, 0.73) | 16614  (14076, 19162) | -27.79  (-38.52, -17.99) | 547418  (467860, 607837) | -10.08  (-24.49, 1.53) |
| South Africa | Subarachnoid hemorrhage | 70398  (66342, 75387) | 41.83  (33.34, 52.65) | 3228  (3145, 3330) | -0.38  (-2.87, 2.87) | 1071  (1028, 1128) | 5.81  (0.65, 10.97) | 45470  (37317, 55366) | 19.52  (-4.04, 49.69) |
| Spain | All stroke | 998541  (954554, 1055432) | 49.06  (42.49, 57.55) | 57115  (54547, 60929) | 38.18  (31.97, 47.41) | 11728  (10939, 12592) | 54.2  (43.8, 65.57) | 383634  (319957, 459570) | 61.96  (35.08, 94.02) |
| Spain | Intracerebral hemorrhage | 69552  (65768, 73463) | 9.04  (1.92, 16.45) | 8794  (8250, 9431) | -3.77  (-14.12, 7.46) | 5255  (4971, 5545) | -4  (-9.6, 1.44) | 146056  (130148, 168002) | 12.33  (-9.43, 40.61) |
| Spain | Ischemic stroke | 559904  (528461, 596135) | 5.3  (-1.72, 13.6) | 44897  (42909, 48035) | -7.64  (-14.77, -0.44) | 5053  (4599, 5564) | 9.77  (1.14, 21.36) | 191564  (147558, 239565) | 12.04  (-17.38, 55.95) |
| Spain | Subarachnoid hemorrhage | 63321  (61466, 65474) | 7.12  (3.48, 11.27) | 3423  (3387, 3462) | -0.78  (-2.2, 0.39) | 1420  (1369, 1483) | 6.86  (3.43, 9.31) | 46015  (42250, 52003) | 5.56  (-3.51, 15.74) |
| Sri Lanka | All stroke | 344359  (326700, 360663) | 21.16  (14.95, 26.9) | 28919  (26941, 31450) | 17.57  (9.52, 27.85) | 13360  (11157, 15682) | -2.83  (-18.85, 14.05) | 413759  (357728, 471839) | 10.25  (-4.68, 25.73) |
| Sri Lanka | Intracerebral hemorrhage | 42613  (40234, 45075) | -4.34  (-9.68, 1.73) | 5766  (4991, 6745) | -21.77  (-32.92, -4.59) | 6048  (5003, 7191) | -32.58  (-42.36, -21.48) | 213804  (185372, 238925) | -12.72  (-26.94, 0.93) |
| Sri Lanka | Ischemic stroke | 221647  (210618, 235089) | -8.37  (-13.89, -1.69) | 20879  (19795, 22344) | -12.23  (-20.35, -5.06) | 6819  (5682, 7980) | -27.5  (-37.89, -15.75) | 178548  (153474, 208913) | -25.24  (-43.4, -12.41) |
| Sri Lanka | Subarachnoid hemorrhage | 27700  (26579, 28962) | -15.64  (-20.04, -11.65) | 2274  (2156, 2361) | -11.99  (-15.73, -8.95) | 493  (472, 511) | 1.09  (-2.73, 5.46) | 21406  (18882, 24001) | 10.44  (-5.82, 26.39) |
| Sudan | All stroke | 770297  (726888, 826108) | 31.23  (23.84, 40.74) | 74336  (68782, 79428) | 29.88  (20.18, 38.78) | 35659  (32812, 38777) | 18.74  (9.25, 29.12) | 1197537  (1036286, 1357758) | 26.12  (9.14, 42.99) |
| Sudan | Intracerebral hemorrhage | 116438  (109044, 122855) | 33.64  (25.67, 40.58) | 21881  (19963, 23193) | 68.99  (55.3, 79.21) | 15325  (13591, 17667) | 9.18  (-3.19, 27.8) | 619900  (531873, 705716) | 41.25  (22.82, 58.26) |
| Sudan | Ischemic stroke | 528188  (499363, 560625) | 9.99  (4.68, 16.33) | 49337  (45773, 53065) | 2.88  (-3.75, 10.32) | 19187  (18177, 19853) | -9.94  (-14.32, -6.5) | 537318  (471385, 604073) | -6.3  (-16.28, 4.04) |
| Sudan | Subarachnoid hemorrhage | 46840  (44486, 49098) | 16.44  (10.69, 22.2) | 3118  (3045, 3171) | -2.36  (-4.73, -0.68) | 1148  (1043, 1258) | -21.6  (-29.62, -13.59) | 40318  (33029, 47969) | -24.22  (-37.99, -11.03) |
| Suriname | All stroke | 9547  (9110, 9952) | 22.96  (17.32, 28.18) | 874  (787, 956) | 6.23  (-4.37, 16.23) | 557  (475, 621) | 5.2  (-10.19, 17.43) | 13665  (11549, 15530) | -9.84  (-23.8, 2.47) |
| Suriname | Intracerebral hemorrhage | 1546  (1477, 1621) | -6.87  (-11.19, -2) | 286  (249, 326) | -33.52  (-43.47, -21.25) | 323  (270, 357) | -29.14  (-39.45, -21.43) | 8433  (7352, 9426) | -35.52  (-45.4, -24.76) |
| Suriname | Ischemic stroke | 5370  (4984, 5757) | -8.82  (-16.82, -0.09) | 511  (462, 552) | -16.28  (-25.49, -6.75) | 197  (170, 227) | -20.91  (-31.01, -10.13) | 3902  (2937, 4698) | -38.62  (-60.1, -21.72) |
| Suriname | Subarachnoid hemorrhage | 1158  (1129, 1191) | 5.89  (2.98, 9.49) | 77  (76, 78) | -2.85  (-4.68, -1.35) | 37  (35, 37) | -10.2  (-13.65, -8.22) | 1330  (1260, 1406) | -4.21  (-10.16, 2.46) |
| Sweden | All stroke | 212843  (205248, 219958) | 4.59  (0.86, 8.09) | 11283  (10672, 11900) | -2.01  (-7.31, 3.35) | 2279  (2145, 2424) | 5.9  (-0.33, 12.63) | 78186  (67279, 89117) | 22.74  (5.61, 39.9) |
| Sweden | Intracerebral hemorrhage | 20595  (19691, 21437) | -9.43  (-14.65, -4.15) | 2005  (1864, 2158) | -16.22  (-25.78, -7.01) | 939  (864, 1012) | -10.69  (-17.39, -4.53) | 24197  (20340, 28349) | -8.8  (-33.67, 13.14) |
| Sweden | Ischemic stroke | 109696  (104486, 114179) | -7.51  (-11.51, -3.04) | 8390  (7936, 8820) | -15.48  (-22.49, -9.54) | 1035  (989, 1096) | -8.66  (-12.81, -2.64) | 42420  (35855, 48640) | 20.17  (-7.06, 44.72) |
| Sweden | Subarachnoid hemorrhage | 14642  (14064, 15086) | -0.8  (-4.82, 2.24) | 888  (872, 922) | -4.04  (-6.06, -2.02) | 305  (292, 316) | 6.01  (0.55, 15.85) | 11569  (11084, 12129) | 31.07  (23.38, 37.76) |
| Switzerland | All stroke | 169161  (158746, 180243) | 45.35  (36.41, 54.88) | 8711  (7967, 9690) | 25.92  (15.18, 40.09) | 1849  (1710, 1986) | 72.12  (59.2, 84.89) | 57307  (44093, 80780) | 68.38  (29.55, 137.35) |
| Switzerland | Intracerebral hemorrhage | 15023  (14429, 15764) | 11.81  (6.75, 18.92) | 1870  (1731, 1992) | 25.75  (9.93, 39.61) | 729  (673, 799) | 34.85  (23.45, 44.95) | 22449  (18361, 30238) | 61.87  (25.48, 119.37) |
| Switzerland | Ischemic stroke | 87670  (81501, 99173) | 8.99  (0.21, 21.85) | 6141  (5550, 6985) | -10.12  (-20.86, 2.8) | 871  (802, 926) | 25  (15.12, 32.27) | 25957  (17314, 41020) | 10.7  (-28.51, 73.78) |
| Switzerland | Subarachnoid hemorrhage | 12371  (11870, 12809) | 6.35  (2.67, 10.58) | 699  (685, 713) | -2.58  (-4.21, -0.81) | 248  (235, 260) | 18.79  (11.41, 30.2) | 8902  (8418, 9523) | 34.75  (26.7, 42.5) |
| Syrian Arab Republic | All stroke | 392044  (377248, 407825) | 25.85  (21.1, 30.92) | 33285  (30729, 35738) | 22.81  (13.38, 31.86) | 19615  (17880, 20756) | 24.85  (13.81, 32.11) | 594218  (530221, 660387) | 26.19  (12.6, 40.24) |
| Syrian Arab Republic | Intracerebral hemorrhage | 67870  (64696, 71423) | 9.64  (4.42, 15.1) | 9986  (8937, 11055) | 4.01  (-7.19, 15.11) | 8063  (7415, 8692) | -14.67  (-21.46, -7.5) | 249026  (216393, 279060) | -8.16  (-20.14, 2.56) |
| Syrian Arab Republic | Ischemic stroke | 263276  (255089, 274753) | 2.27  (-0.7, 6.74) | 21781  (20314, 23112) | -4.37  (-10.87, 1.37) | 10946  (9889, 11422) | -7.34  (-16.56, -3.07) | 321881  (293480, 354638) | 3.36  (-5.95, 13.77) |
| Syrian Arab Republic | Subarachnoid hemorrhage | 29907  (28825, 31210) | -2.7  (-6.23, 1.45) | 1518  (1478, 1571) | 1.61  (-1, 5.22) | 606  (575, 642) | -1.96  (-6.86, 3.92) | 23311  (20347, 26690) | 1.07  (-11.49, 15.83) |
| Tajikistan | All stroke | 175927  (166119, 189222) | 46.93  (38.74, 58.03) | 21455  (19824, 23287) | 31.31  (21.33, 42.53) | 7896  (6825, 9282) | 13.49  (-1.91, 33.42) | 273411  (230653, 318618) | 28.75  (8.61, 50.04) |
| Tajikistan | Intracerebral hemorrhage | 21584  (19420, 24665) | -4.58  (-14.1, 9.08) | 6179  (5608, 6774) | -21.6  (-28.64, -13.44) | 3312  (2734, 3926) | -39.38  (-50.16, -27.98) | 93312  (71636, 115509) | -37.43  (-52.22, -23.84) |
| Tajikistan | Ischemic stroke | 122529  (112956, 130089) | 13.28  (4.44, 20.06) | 13941  (12902, 15161) | 2.29  (-5.32, 11.03) | 4193  (3712, 4950) | -15.78  (-25.2, -0.96) | 162031  (143782, 181440) | 19.29  (6.28, 33.94) |
| Tajikistan | Subarachnoid hemorrhage | 13490  (12916, 14372) | 2.69  (-1.71, 9.54) | 1335  (1314, 1352) | -0.82  (-2.29, 0.57) | 391  (379, 406) | -0.28  (-3.12, 3.69) | 18069  (15235, 21668) | 21.54  (2.52, 46.06) |
| Thailand | All stroke | 1577623  (1500598, 1688463) | 49.64  (42.34, 60.16) | 119040  (105708, 132694) | 41.04  (25.25, 57.22) | 47574  (42027, 53862) | 14.39  (1.05, 29.51) | 1491281  (1158263, 1904063) | 11.68  (-13.26, 42.59) |
| Thailand | Intracerebral hemorrhage | 217332  (205074, 232754) | -7.03  (-13.31, -1.22) | 22597  (18292, 26997) | -38.99  (-54.69, -24.42) | 21744  (19415, 24458) | -35.41  (-45.02, -26.72) | 496808  (389194, 653947) | -46.05  (-58.31, -32.69) |
| Thailand | Ischemic stroke | 897119  (839369, 963977) | -1.16  (-10.14, 9.63) | 85831  (77250, 94738) | 7.88  (-5.34, 21.51) | 20113  (17572, 23132) | -3.54  (-16.71, 9.43) | 749369  (540287, 986784) | -5.11  (-41.35, 28.26) |
| Thailand | Subarachnoid hemorrhage | 137532  (132123, 144380) | -5.87  (-9.05, -1.74) | 10611  (10167, 10958) | -5.02  (-8.09, -2.61) | 5717  (5040, 6272) | -25.77  (-33.22, -18.44) | 245104  (228781, 263332) | -6.12  (-12.29, -0.08) |
| Timor-Leste | All stroke | 20313  (16785, 26361) | 31.22  (8.42, 70.28) | 1923  (1559, 2379) | -7.04  (-24.64, 14.98) | 2675  (1628, 3842) | 62.75  (-0.98, 133.74) | 20551  (10165, 32443) | -55.15  (-77.82, -29.2) |
| Timor-Leste | Intracerebral hemorrhage | 4543  (3683, 6181) | 25.56  (1.17, 68.59) | 800  (616, 1009) | -17.25  (-36.15, 2.71) | 1830  (1057, 2592) | 37.17  (-20.01, 100.54) | 11625  (5632, 18495) | -68.99  (-84.42, -47.13) |
| Timor-Leste | Ischemic stroke | 11723  (8956, 15658) | 5.07  (-19.36, 36.66) | 992  (815, 1234) | -21.37  (-34.71, -3.82) | 761  (492, 1139) | 30.79  (-15.17, 96.63) | 4708  (1635, 7675) | -69.64  (-88.2, -46.73) |
| Timor-Leste | Subarachnoid hemorrhage | 1970  (1735, 2463) | 25.85  (12, 56.32) | 131  (128, 136) | -1.6  (-4.31, 1.6) | 84  (78, 111) | 2.54  (-5.08, 32.54) | 4217  (2897, 6273) | 44.48  (1.65, 112.98) |
| Togo | All stroke | 149398  (142256, 156513) | 10.73  (5.43, 16) | 12074  (11075, 13052) | -4.47  (-12.37, 3.27) | 7570  (6707, 8440) | -8.48  (-18.91, 2.04) | 182135  (145198, 217148) | -29.1  (-43.48, -15.47) |
| Togo | Intracerebral hemorrhage | 24890  (23457, 26267) | -21.47  (-25.69, -17.22) | 3911  (3482, 4313) | -27.73  (-34.28, -21.26) | 4065  (3461, 4643) | -34.05  (-43.06, -24.81) | 98103  (77101, 119270) | -47.5  (-57.92, -37.71) |
| Togo | Ischemic stroke | 96939  (91072, 103137) | -5.07  (-10.13, 0.57) | 7620  (7058, 8186) | -3.69  (-10.42, 3.38) | 3239  (2996, 3521) | -16.54  (-22.6, -8.8) | 76038  (61434, 88140) | -29.55  (-39.93, -19.76) |
| Togo | Subarachnoid hemorrhage | 10563  (9975, 11245) | 14.65  (8.64, 21.55) | 543  (535, 552) | -0.54  (-1.81, 1.45) | 265  (250, 276) | -8.04  (-13.18, -4.5) | 7994  (6662, 9738) | -28.03  (-39.04, -13.34) |
| Tonga | All stroke | 1627  (1476, 1831) | 30.86  (18.68, 47.3) | 144  (122, 171) | 37.95  (17.08, 64.19) | 127  (100, 174) | 151.58  (98.27, 245.31) | 2871  (2222, 3733) | 85.87  (43.85, 141.66) |
| Tonga | Intracerebral hemorrhage | 318  (284, 378) | 29.85  (15.82, 53.93) | 60  (48, 76) | 41.92  (13.53, 83.18) | 95  (74, 131) | 136.72  (87.15, 224.21) | 2047  (1687, 2609) | 82.34  (49.95, 134.69) |
| Tonga | Ischemic stroke | 1023  (876, 1175) | 29.92  (11.71, 51.71) | 74  (65, 85) | 10.04  (-4.25, 26.18) | 27  (22, 35) | 69.2  (37.09, 119.14) | 520  (291, 720) | 3.72  (-41.46, 51.43) |
| Tonga | Subarachnoid hemorrhage | 181  (166, 200) | 17.96  (7.85, 30.35) | 10  (10, 10) | 2.76  (-0.72, 7.87) | 5  (4, 8) | 56.69  (19.48, 133.14) | 304  (244, 404) | 111.05  (67.97, 181.81) |
| Trinidad and Tobago | All stroke | 29052  (26830, 31643) | 64.93  (52.32, 79.64) | 2157  (1960, 2414) | 47.41  (33.96, 64.93) | 972  (833, 1124) | 21.27  (3.91, 40.14) | 31167  (27210, 36435) | 43.43  (25.22, 67.67) |
| Trinidad and Tobago | Intracerebral hemorrhage | 2589  (2452, 2766) | 0.29  (-6.79, 8.47) | 420  (375, 474) | -13.93  (-27.82, -0.48) | 388  (337, 460) | -28.34  (-37.05, -15.86) | 11222  (9886, 13005) | -14.79  (-28.4, 2.93) |
| Trinidad and Tobago | Ischemic stroke | 19171  (17650, 20921) | 11.08  (-0.74, 23.9) | 1576  (1435, 1772) | 7.39  (-5.77, 21.32) | 494  (417, 565) | -23.98  (-36.5, -13.95) | 16976  (14523, 20178) | 24.27  (-4.23, 53.71) |
| Trinidad and Tobago | Subarachnoid hemorrhage | 2784  (2721, 2863) | 7.83  (4.09, 11.96) | 162  (151, 167) | -6.49  (-10.36, -3.87) | 91  (79, 99) | -13.28  (-20.5, -5.71) | 2970  (2800, 3252) | -15.68  (-21.32, -8.37) |
| Tunisia | All stroke | 205096  (196577, 215649) | 33.67  (28.12, 40.55) | 18515  (17174, 19747) | 30.31  (20.88, 38.98) | 8202  (7316, 9265) | 28.06  (14.22, 44.65) | 228155  (195501, 266491) | 28.76  (10.33, 50.39) |
| Tunisia | Intracerebral hemorrhage | 18650  (17406, 19919) | -7.32  (-14.3, -0.71) | 3323  (2875, 3681) | -6.37  (-22.15, 4.34) | 2640  (2403, 2933) | -14.43  (-21.35, -5.2) | 62252  (49454, 75882) | -26.17  (-45.49, -7.4) |
| Tunisia | Ischemic stroke | 140759  (132970, 147459) | -8.51  (-13.59, -3.06) | 14292  (13424, 15147) | -13.88  (-20.64, -7.43) | 5213  (4579, 5973) | -24.51  (-32.72, -14.79) | 151603  (133349, 174138) | -20.57  (-34.96, -5.88) |
| Tunisia | Subarachnoid hemorrhage | 13659  (12970, 14188) | -3.82  (-8.27, 0.73) | 899  (875, 919) | -4.19  (-6.43, -2.54) | 348  (334, 359) | 0.52  (-3.61, 3.61) | 14300  (12698, 16471) | 24.62  (4.74, 44.74) |
| Turkmenistan | All stroke | 122691  (110618, 134776) | 21.57  (9.61, 33.54) | 8281  (7278, 9356) | -30.25  (-38.7, -21.2) | 5230  (4188, 6214) | -22.34  (-37.81, -7.73) | 151933  (125471, 180311) | -28.05  (-40.58, -14.61) |
| Turkmenistan | Intracerebral hemorrhage | 12616  (11546, 13436) | -27.84  (-33.59, -22.7) | 2366  (2056, 2714) | -48.23  (-55.01, -38.79) | 2167  (1772, 2594) | -50.83  (-59.57, -42.43) | 61524  (51571, 71345) | -51.78  (-59.59, -43.92) |
| Turkmenistan | Ischemic stroke | 91800  (80082, 103429) | 0.6  (-12.96, 12.84) | 5174  (4540, 5859) | -47.18  (-54.48, -39.98) | 2694  (2094, 3203) | -41.24  (-54.14, -31.07) | 76754  (61601, 93984) | -41.03  (-53.35, -27.22) |
| Turkmenistan | Subarachnoid hemorrhage | 8030  (7697, 8499) | 1.14  (-3.11, 6.86) | 741  (682, 782) | -8.27  (-14.83, -3.65) | 370  (322, 416) | -34.34  (-42.72, -26.46) | 13656  (12298, 14982) | -29.83  (-36.72, -23.12) |
| Uganda | All stroke | 771318  (729377, 841546) | 39.98  (32.37, 52.73) | 75770  (69605, 81814) | 40.08  (28.68, 51.25) | 26015  (24006, 28749) | 11.67  (3.06, 23.4) | 839148  (677094, 1021994) | 15.31  (-6.95, 40.44) |
| Uganda | Intracerebral hemorrhage | 128470  (120988, 135812) | 12.72  (6.97, 18.68) | 20316  (18349, 22395) | -21.28  (-27.66, -15.72) | 16650  (15273, 18553) | -35.69  (-40.42, -28.28) | 499337  (422123, 599566) | -34.1  (-43.06, -22.86) |
| Uganda | Ischemic stroke | 540349  (502710, 586821) | 12.95  (7.36, 19.55) | 52074  (47976, 55954) | 16.91  (9.29, 23.66) | 8407  (7854, 9170) | 2.68  (-2.6, 13.96) | 302216  (224767, 377077) | 27.98  (6.41, 46.98) |
| Uganda | Subarachnoid hemorrhage | 52341  (48944, 56410) | 1.57  (-3.86, 8.4) | 3379  (3280, 3465) | -4.91  (-8.73, -2.46) | 958  (879, 1026) | -8.17  (-18.29, -1.17) | 37594  (30205, 45350) | -13.94  (-26.65, 1.34) |
| Ukraine | All stroke | 947113  (914159, 982103) | 30.74  (26.19, 35.57) | 118462  (114099, 124526) | 45.41  (40.05, 52.85) | 46803  (41098, 55512) | 30.99  (15.02, 55.36) | 1857117  (1670587, 2052234) | 87.62  (68.77, 107.33) |
| Ukraine | Intracerebral hemorrhage | 70294  (65077, 87069) | -6.92  (-14.77, 9) | 20902  (19223, 23090) | 0.16  (-11.37, 9.26) | 14870  (13758, 16195) | 8.06  (-0.78, 17.03) | 441763  (385724, 509265) | 10.41  (-9.32, 23.52) |
| Ukraine | Ischemic stroke | 727611  (702567, 757789) | 0.25  (-4.89, 5.53) | 93531  (90900, 97352) | 4.41  (-0.72, 8.9) | 29267  (24771, 36516) | -17.55  (-28.21, -0.62) | 1321550  (1199376, 1436319) | 31  (20.26, 43.78) |
| Ukraine | Subarachnoid hemorrhage | 43179  (41545, 45704) | -6.4  (-10.87, 1.28) | 4029  (3976, 4084) | -1.55  (-3.02, -0.17) | 2666  (2570, 2801) | 4.43  (0.74, 9.78) | 93804  (85487, 106649) | 6.53  (-2.86, 18.85) |
| United Arab Emirates | All stroke | 176492  (162788, 193247) | 34.18  (23.76, 46.92) | 10637  (9103, 12520) | -8.29  (-21.52, 7.94) | 1703  (1529, 1916) | 29.17  (15.94, 45.26) | 95282  (68398, 122799) | 61.71  (16.09, 108.42) |
| United Arab Emirates | Intracerebral hemorrhage | 19457  (17665, 21641) | -22.05  (-29.01, -13.55) | 1750  (1529, 2004) | -44.05  (-53.55, -36.28) | 824  (747, 921) | -51.01  (-55.56, -45.86) | 29922  (21899, 37821) | -30.63  (-50.48, -13.97) |
| United Arab Emirates | Ischemic stroke | 128379  (113231, 144625) | -24.98  (-33.59, -16.59) | 8216  (6934, 9810) | -46.14  (-55.71, -36.92) | 661  (602, 739) | -78.45  (-80.53, -75.77) | 56541  (38854, 74961) | -31.94  (-54.01, -11.28) |
| United Arab Emirates | Subarachnoid hemorrhage | 10507  (9968, 10985) | -5.25  (-10, -0.37) | 672  (641, 706) | -4.63  (-8.56, -0.7) | 219  (181, 256) | -25.76  (-37.88, -12.12) | 8820  (7645, 10018) | -7.96  (-20.73, 4.74) |
| United Kingdom | All stroke | 1331731  (1249646, 1402995) | 37.83  (29.34, 45.21) | 70442  (65140, 74303) | 17.98  (9.11, 24.45) | 15672  (14656, 16833) | 22.79  (14.85, 31.91) | 564619  (503391, 648624) | 46.34  (30.47, 68.11) |
| United Kingdom | Intracerebral hemorrhage | 113043  (107472, 119168) | 21.07  (12.72, 28.95) | 11209  (10306, 12108) | -4.92  (-16.11, 7.43) | 6186  (5731, 6630) | -11.26  (-18.38, -4.97) | 168943  (147181, 192629) | 1.71  (-12.95, 18.14) |
| United Kingdom | Ischemic stroke | 770730  (722668, 813130) | 17.51  (9.02, 24.82) | 53567  (49281, 56383) | -4.91  (-15.55, 2.82) | 6957  (6484, 7599) | 13.4  (6.17, 24.04) | 295803  (261951, 350420) | 34.45  (10.44, 59.6) |
| United Kingdom | Subarachnoid hemorrhage | 89622  (85684, 92433) | -3.58  (-6.82, 0.05) | 5666  (5554, 5812) | -3.76  (-6.04, -2.16) | 2528  (2441, 2605) | -2.69  (-5.39, 0) | 99872  (94260, 105574) | 19.93  (13.17, 27.93) |
| United Republic of Tanzania | All stroke | 1174435  (1116467, 1244369) | 20.53  (14.58, 27.71) | 106856  (98017, 115505) | 11.07  (1.89, 20.06) | 46748  (43811, 50987) | 17.02  (9.67, 27.63) | 1307152  (1066753, 1541222) | 10.41  (-9.89, 30.19) |
| United Republic of Tanzania | Intracerebral hemorrhage | 189332  (181767, 201100) | 12.8  (7.96, 18.47) | 31657  (28449, 34317) | -6.49  (-15.41, 0.02) | 28281  (26269, 30783) | -9.4  (-15.95, -1.51) | 752076  (617796, 873530) | -14.4  (-28.17, -2.91) |
| United Republic of Tanzania | Ischemic stroke | 793913  (745034, 845716) | 4.33  (-1.87, 9.75) | 70035  (64634, 75852) | -1.3  (-8.45, 5.59) | 17073  (16242, 18691) | 3.96  (-0.6, 12.92) | 484580  (390915, 583012) | 12.93  (-3.77, 28.26) |
| United Republic of Tanzania | Subarachnoid hemorrhage | 81898  (77360, 88381) | 8.48  (3.05, 16.74) | 5163  (4934, 5336) | -5.11  (-9.23, -2.41) | 1394  (1300, 1513) | -4.81  (-11.06, 2.88) | 70495  (58042, 84681) | 17.27  (-2.05, 39.15) |
| United States of America | All stroke | 9902032  (9085483, 10927774) | 43.96  (32.09, 58.88) | 443938  (368718, 538889) | 42.82  (18.61, 73.36) | 86522  (76464, 104688) | 0.32  (-11.34, 21.37) | 3623037  (2691433, 5529528) | 27.82  (-5.05, 95.08) |
| United States of America | Intracerebral hemorrhage | 835290  (794168, 890499) | 12.44  (5.97, 19.57) | 61029  (54130, 72313) | -3.77  (-19.4, 17.32) | 39073  (33871, 44888) | -20.7  (-31.57, -7.39) | 1257008  (1009428, 1671836) | -1.52  (-24.38, 28.99) |
| United States of America | Ischemic stroke | 6348237  (5598865, 7211709) | 22.71  (11.54, 33.55) | 356662  (289596, 439421) | 20.28  (0.59, 48.45) | 32101  (28345, 43262) | -20.15  (-29.16, 5.04) | 1781481  (1169437, 3169154) | 17.66  (-30.99, 89.59) |
| United States of America | Subarachnoid hemorrhage | 481298  (462100, 501253) | 0.2  (-3.54, 3.58) | 26247  (24991, 27154) | -4.38  (-7.3, -1.59) | 15348  (14248, 16538) | 0  (-6.55, 5.41) | 584547  (512568, 688537) | 7.11  (-4.73, 22.01) |
| Uruguay | All stroke | 77766  (66569, 104290) | 48.55  (27.16, 99.21) | 4227  (3734, 5022) | 12.35  (-0.74, 33.5) | 971  (839, 1123) | -29.44  (-39.03, -18.37) | 29995  (20674, 41109) | -23.22  (-47.08, 5.23) |
| Uruguay | Intracerebral hemorrhage | 7699  (7223, 8220) | 2.78  (-4.54, 12.01) | 838  (755, 965) | -14.74  (-26.32, 1.08) | 406  (355, 456) | -38.33  (-47.06, -29.07) | 9652  (6553, 12873) | -35.71  (-58.42, -15.48) |
| Uruguay | Ischemic stroke | 49888  (37586, 64816) | 37.1  (3.24, 117.95) | 2890  (2542, 3528) | -6.25  (-19.54, 19.26) | 339  (297, 419) | -59.02  (-65.23, -49.61) | 13192  (7639, 20575) | -32.5  (-71.92, 12.97) |
| Uruguay | Subarachnoid hemorrhage | 7120  (6913, 7483) | 3.05  (-0.76, 8.39) | 498  (437, 530) | -8.22  (-18.06, -1.38) | 226  (187, 248) | -18.99  (-28.49, -9.64) | 7151  (6482, 7661) | -18.61  (-26.88, -11.72) |
| Uzbekistan | All stroke | 624549  (587623, 657426) | 11.01  (4.45, 16.85) | 78819  (71745, 86678) | 13.44  (3.25, 24.75) | 27433  (23336, 31165) | 15.17  (-2.02, 30.85) | 788935  (651140, 921398) | 5.96  (-12.55, 23.75) |
| Uzbekistan | Intracerebral hemorrhage | 75926  (70622, 80760) | -21.45  (-27.56, -16.42) | 20794  (17789, 23860) | -31.03  (-41.48, -20.9) | 11783  (10385, 13239) | -26.58  (-36.02, -17.68) | 293826  (249743, 339770) | -35  (-45.75, -24.43) |
| Uzbekistan | Ischemic stroke | 438551  (413660, 473456) | -19.87  (-24.32, -13.2) | 55103  (51108, 59815) | -9.46  (-16.09, -0.8) | 13836  (11300, 15944) | -25.93  (-37.96, -15.25) | 443327  (358589, 520279) | -20.16  (-36.35, -4.56) |
| Uzbekistan | Subarachnoid hemorrhage | 38208  (36278, 40455) | 10.99  (5.67, 17.61) | 2922  (2849, 3004) | 0.52  (-1.82, 3.52) | 1814  (1652, 1982) | -4.11  (-13.85, 4.55) | 51781  (42809, 61349) | -10.02  (-26.58, 7.9) |
| Vanuatu | All stroke | 8085  (7593, 8843) | 7.87  (1.3, 17.99) | 1121  (956, 1307) | 38.14  (17.77, 61.04) | 609  (519, 715) | 31.73  (12.24, 54.66) | 17527  (14241, 21503) | 10.91  (-9.89, 36.06) |
| Vanuatu | Intracerebral hemorrhage | 1934  (1782, 2101) | -11.07  (-17.86, -3.74) | 520  (430, 633) | 20.55  (0.89, 44.13) | 427  (368, 498) | 0.1  (-13.21, 17.61) | 12786  (10778, 15609) | -3.39  (-17.96, 16.03) |
| Vanuatu | Ischemic stroke | 5678  (5011, 6438) | 10.48  (-1.46, 24.75) | 529  (459, 600) | 17.93  (3.55, 33.15) | 146  (118, 179) | 30.96  (6.26, 59.7) | 3473  (2312, 4508) | -3.06  (-33.5, 24.75) |
| Vanuatu | Subarachnoid hemorrhage | 706  (664, 753) | -24.62  (-28.8, -19.57) | 72  (67, 75) | -4.79  (-11, -0.58) | 36  (33, 38) | -6.16  (-13.32, 0.3) | 1268  (1151, 1386) | -21.46  (-28.44, -14.1) |
| Viet Nam | All stroke | 2031563  (1931963, 2137348) | 41.47  (34.53, 48.83) | 214862  (195862, 240108) | 19.69  (9.1, 33.75) | 111369  (93175, 135687) | -14.4  (-28.39, 4.29) | 3714023  (3148748, 4350046) | 1.35  (-14.08, 18.7) |
| Viet Nam | Intracerebral hemorrhage | 323712  (306880, 345092) | -9.56  (-14.78, -2.69) | 67847  (59346, 75480) | -31.77  (-40.87, -23.91) | 54557  (44591, 68000) | -55.88  (-62.2, -47.04) | 1635428  (1400746, 1941691) | -47.8  (-55.21, -38.12) |
| Viet Nam | Ischemic stroke | 1228103  (1144260, 1307504) | -6.62  (-14.95, 0.48) | 134431  (124439, 151223) | -8.67  (-18.92, 2.05) | 50568  (42764, 60965) | -34.12  (-43.25, -19.86) | 1821879  (1517533, 2123330) | -8.09  (-24.04, 6.55) |
| Viet Nam | Subarachnoid hemorrhage | 161976  (153697, 172826) | 6.56  (0.83, 11.81) | 12584  (12077, 13405) | 2.2  (-2.2, 10.36) | 6245  (5820, 6722) | 0.97  (-5.23, 10.27) | 256716  (230469, 285026) | 22.68  (8.3, 39.4) |
| Yemen | All stroke | 445793  (427342, 468512) | 26.62  (21.38, 33.07) | 51226  (47886, 54709) | 29.99  (21.51, 38.82) | 40038  (36525, 41992) | 44.88  (32.16, 51.95) | 1032480  (932518, 1128126) | 26.34  (14.11, 38.04) |
| Yemen | Intracerebral hemorrhage | 72370  (68924, 76986) | 15.98  (10.39, 23.42) | 14910  (13460, 16324) | 23.29  (11.83, 35.23) | 15404  (14048, 16164) | -9.78  (-17.23, -4.99) | 457643  (404562, 510521) | -4.77  (-15.92, 5.49) |
| Yemen | Ischemic stroke | 323769  (306277, 341911) | -2.14  (-7.32, 2.8) | 33417  (31575, 35433) | -12.27  (-16.74, -7.66) | 23029  (20952, 24147) | -7.87  (-16.83, -3.72) | 523161  (482014, 560734) | -16.65  (-22.67, -11.35) |
| Yemen | Subarachnoid hemorrhage | 36289  (35168, 38260) | 8.35  (4.33, 13.73) | 2900  (2851, 2952) | -1.13  (-2.88, 0.63) | 1605  (1525, 1681) | -7.99  (-13.45, -3.9) | 51676  (45942, 56871) | -10.26  (-19.89, -1.87) |
| Zambia | All stroke | 326145  (311205, 346749) | 22.42  (16.81, 30.15) | 38347  (35390, 41523) | 24.09  (14.52, 34.37) | 20665  (18442, 23467) | 19.22  (6.4, 35.38) | 578121  (491633, 663724) | 10.13  (-6.35, 26.43) |
| Zambia | Intracerebral hemorrhage | 56772  (53982, 59833) | -13.96  (-18.14, -9.55) | 13890  (12618, 15524) | -24.98  (-31.76, -17.86) | 12831  (11124, 15150) | -41.26  (-50.25, -29.69) | 373644  (317576, 422291) | -37.64  (-46.45, -30.17) |
| Zambia | Ischemic stroke | 224937  (207981, 239985) | -12.97  (-18.65, -7.92) | 22582  (20937, 24093) | -17.13  (-22.67, -12.57) | 7162  (6695, 7600) | -10  (-15.56, -4.29) | 183814  (157229, 217185) | -9.17  (-20.03, 3.01) |
| Zambia | Subarachnoid hemorrhage | 22880  (21760, 24229) | -14.16  (-18.12, -10.08) | 1875  (1835, 1906) | -4.05  (-6.46, -2.3) | 672  (622, 717) | -18.96  (-25.59, -12.09) | 20663  (16828, 24248) | -33.64  (-44.34, -23.96) |
| Zimbabwe | All stroke | 217087  (206079, 229568) | 13.04  (7.31, 19.54) | 22283  (20427, 24433) | 17.41  (7.63, 28.73) | 15418  (13710, 17015) | 3.06  (-8.36, 13.73) | 389030  (314413, 464628) | -13.06  (-29.74, 3.83) |
| Zimbabwe | Intracerebral hemorrhage | 22252  (19547, 25540) | -2.16  (-13.23, 10.65) | 4655  (3957, 5680) | 5.19  (-9.58, 24.79) | 9076  (7776, 10277) | -23.96  (-34.31, -12.62) | 226563  (181808, 275897) | -31.67  (-44.33, -18.7) |
| Zimbabwe | Ischemic stroke | 153389  (141855, 164642) | -1.97  (-7.84, 4.43) | 16124  (15004, 17219) | 6.29  (0.66, 12.84) | 5528  (5188, 5882) | -1.32  (-6.44, 6.47) | 137355  (110104, 160692) | -5.87  (-18.17, 8.82) |
| Zimbabwe | Subarachnoid hemorrhage | 19536  (18558, 20916) | -5.37  (-9.85, 0.57) | 1504  (1466, 1533) | -2.65  (-4.74, -0.77) | 814  (746, 856) | -10.07  (-18.26, -5.29) | 25111  (22502, 28039) | -26.49  (-32.75, -18.47) |

Notes: UI=uncertainty interval. DALY=disability adjusted life-year.

# Appendix 4. Burden of all strokes and their pathological types by sex in 2050

**Table S3. Absolute number, crude rates, and age-standardized rates per 100 000 people per year of incident and prevalent strokes, deaths from stroke and DALYs due to stroke in 2050 and percentage change globally for 2021–2050, by sex and pathological type of stroke.**

|  | **Prevalence**  **(95% UI)** | | **Incidence**  **(95% UI)** | | **Deaths**  **(95%UI)** | | **DALYs(95%UI)** | |
| --- | --- | --- | --- | --- | --- | --- | --- | --- |
|  | **2050** | **Percentage change,**  **2021-2050** | **2050** | **Percentage change,**  **2021-2050** | **2050** | **Percentage change,**  **2021-2050** | **2050** | **Percentage change,**  **2021-2050** |
| **Intracerebral haemorrhage** | | | | | | | | |
| **Male** | | | | | | | | |
| Absolute number | 11.24  (11.04, 11.43) | 30.05  (27.71, 32.23) | 1.78  (1.71, 1.84) | 12.74  (8.27, 16.74) | 1.42  (1.33, 1.51) | 0.17  (-6.4, 6.93) | 44.24  (41.49, 46.97) | 8.44  (1.7, 15.15) |
| Crude rate | 304.86  (299.39, 309.98) | 2.47  (0.63, 4.19) | 48.17  (46.26, 49.88) | -11.17  (-14.7, -8.02) | 38.48  (35.96, 41.08) | -21.07  (-26.25, -15.75) | 1199.62  (1125.12, 1273.83) | -14.56  (-19.87, -9.28) |
| Age-standardized rate | 257.18  (253.04, 262.05) | -5.28  (-6.8, -3.48) | 43.50  (42.54, 44.53) | -15.6  (-17.46, -13.6) | 37.47  (35.99, 38.5) | -22.92  (-25.96, -20.8) | 1088.82  (1055.46, 1121.09) | -22.91  (-25.27, -20.62) |
| **Female** | | | | | | | | |
| Absolute number | 8.30  (8.13, 8.47) | 28.3  (25.66, 30.97) | 1.19  (1.14, 1.25) | 11.95  (6.95, 17.13) | 0.97  (0.91, 1.03) | -5.6  (-10.79, 0.71) | 24.62  (22.7, 26.84) | -12.79  (-19.58, -4.9) |
| Crude rate | 222.87  (218.29, 227.52) | 0.67  (-1.4, 2.77) | 31.96  (30.54, 33.44) | -12.16  (-16.08, -8.09) | 25.92  (24.49, 27.65) | -25.93  (-30, -20.97) | 660.86  (609.45, 720.67) | -31.57  (-36.89, -25.38) |
| Age-standardized rate | 188.96  (185.96, 192.68) | -5.06  (-6.57, -3.2) | 26.84  (26.04, 27.92) | -19.5  (-21.9, -16.26) | 26.6  (25.33, 27.53) | -25.28  (-28.85, -22.67) | 731.07  (698.87, 755.94) | -26.41  (-29.65, -23.9) |
| **Ischaemic stroke** | | | | | | | | |
| **Male** | | | | | | | | |
| Absolute number | 48.76  (47.57, 50.11) | 62.55  (58.59, 67.05) | 4.80  (4.63, 4.97) | 47.55  (42.46, 52.89) | 1.3  (1.2, 1.43) | 29.29  (19.56, 41.89) | 40.12  (38.01, 42.37) | 45.15  (37.5, 53.27) |
| Crude rate | 1322.32  (1290.07, 1358.85) | 28.08  (24.96, 31.62) | 130.17  (125.68, 134.89) | 16.25  (12.25, 20.47) | 35.3  (32.64, 38.74) | 1.87  (-5.8, 11.79) | 1088.07  (1030.75, 1148.95) | 14.36  (8.34, 20.76) |
| Age-standardized rate | 992.16  (976.24, 1008.12) | 4.73  (3.05, 6.41) | 92.71  (91.28, 94.11) | -0.69  (-2.22, 0.81) | 26.94  (26.09, 27.77) | -17.39  (-19.99, -14.84) | 786.80  (762.29, 810.19) | -11.00  (-13.77, -8.36) |
| **Female** | | | | | | | | |
| Absolute number | 39.86  (38.82, 41.01) | 41.91  (38.23, 46.03) | 3.42  (3.31, 3.52) | 31.79  (27.44, 35.71) | 0.93  (0.84, 0.99) | 24.99  (13.19, 33.68) | 27.79  (25.86, 30.07) | 32.77  (23.56, 43.68) |
| Crude rate | 1069.98  (1042.19, 1101.03) | 11.36  (8.47, 14.59) | 91.84  (88.81, 94.57) | 3.41  (0, 6.49) | 24.93  (22.58, 26.66) | -1.92  (-11.18, 4.89) | 746.04  (694.33, 807.39) | 4.18  (-3.04, 12.75) |
| Age-standardized rate | 877.98  (863.98, 892.85) | -1.55  (-3.12, 0.11) | 69.24  (68.04, 70.72) | -9.56  (-11.13, -7.63) | 17.66  (17.05, 18.08) | -21.79  (-24.49, -19.93) | 551.94  (526.95, 577.09) | -12.64  (-16.59, -8.66) |
| **Subarachnoid haemorrhage** | | | | | | | | |
| **Male** |  |  |  |  |  |  |  |  |
| Absolute number | 4.68  (4.55, 4.85) | 42.99  (38.97, 48.4) | 0.38  (0.37, 0.39)^#1^ | 30.03  (26.16, 34.96) | 0.19  (0.18, 0.2)^#2^ | 38.25  (30.94, 44.07) | 6.36  (6.12, 6.63) | 29.36  (24.5, 34.83) |
| Crude rate | 126.84  (123.28, 131.64) | 12.66  (9.5, 16.92) | 10.2  (9.9, 10.59) | 2.45  (-0.6, 6.34) | 5.19  (4.92, 5.41) | 8.93  (3.17, 13.52) | 172.42  (165.94, 179.7) | 1.92  (-1.9, 6.23) |
| Age-standardized rate | 123.81  (122.31, 125.29) | -1.23  (-2.43, -0.05) | 9.13  (9.04, 9.21) | -4.99  (-5.93, -4.16) | 3.90  (3.81, 3.97) | -10.34  (-12.41, -8.74) | 154.90  (151.56, 158.60) | -6.33  (-8.35, -4.09) |
| **Female** | | | | | | | | |
| Absolute number | 6.09  (5.91, 6.28) | 58.33  (53.67, 63.28) | 0.38  (0.37, 0.39)^#3^ | 30.34  (27.49, 34.94) | 0.19  (0.18, 0.19)^#4^ | 46.24  (39.16, 52.06) | 6.09  (5.82, 6.37) | 37.29  (31.09, 43.52) |
| Crude rate | 163.4  (158.58, 168.5) | 24.24  (20.58, 28.12) | 10.18  (9.96, 10.54) | 2.28  (0.04, 5.89) | 5.01  (4.77, 5.21) | 14.75  (9.2, 19.32) | 163.63  (156.23, 171.05) | 7.73  (2.86, 12.62) |
| Age-standardized rate | 146.16  (144.37, 148.02) | 5.83  (4.53, 7.18) | 9.47  (9.37, 9.56) | -5.02  (-6.02, -4.11) | 3.52  (3.46, 3.58) | -9.74  (-11.28, -8.21) | 145.70  (142.46, 149.65) | -0.19  (-2.41, 2.51) |
| **Total stroke** | | | | | | | | |
| **Male** | | | | | | | | |
| Absolute number | 76.65  (75.31, 78.11) | 64.1  (61.22, 67.21) | 6.95  (6.71, 7.20) | 35.84  (31.01, 40.75) | 2.91  (2.71, 3.14) | 13.67  (5.82, 22.67) | 90.72  (85.62, 95.97) | 23.67  (16.72, 30.83) |
| Crude rate | 2078.78  (2042.38, 2118.2) | 29.3  (27.03, 31.75) | 188.54  (181.84, 195.36) | 7.03  (3.23, 10.9) | 78.97  (73.52, 85.23) | -10.43  (-16.62, -3.34) | 2460.10  (2321.81, 2602.48) | -2.56  (-8.03, 3.08) |
| Age-standardized rate | 1541.44  (1522.06, 1564.88) | 4.21  (2.9, 5.8) | 145.34  (142.85, 147.84) | -5.93  (-7.54, -4.31) | 68.31  (65.89, 70.24) | -20.17  (-23, -17.92) | 2030.52  (1969.31, 2089.88) | -17.52  (-20.01, -15.11) |
| **Female** | | | | | | | | |
| Absolute number | 67.77  (66.14, 68.95) | 51.45  (47.79, 54.08) | 4.99  (4.82, 5.16) | 26.34  (21.93, 30.65) | 2.08  (1.93, 2.22) | 9.9  (1.99, 17.11) | 58.50  (54.38, 63.29) | 9.15  (1.47, 18.08) |
| Crude rate | 1819.49  (1775.58, 1851.07) | 18.84  (15.97, 20.9) | 133.98  (129.3, 138.55) | -0.86  (-4.32, 2.52) | 55.86  (51.84, 59.53) | -13.76  (-19.97, -8.1) | 1570.53  (1460.02, 1699.11) | -14.35  (-20.38, -7.34) |
| Age-standardized rate | 1392.61  (1373.49, 1416.77) | 1.19  (-0.2, 2.94) | 105.56  (103.45, 108.19) | -11.93  (-13.69, -9.74) | 47.78  (45.84, 49.19) | -23.05  (-26.17, -20.78) | 1428.72  (1368.27, 1482.67) | -19.33  (-22.75, -16.29) |

Notes: Absolute number are in millions. The denominator of crude incidence rate and age standardized incidence rate is per 100 000 people. Absolute numbers in millions, crude rates per 100 000 people, age-standardised rates per 100 000 people, and percentage change are presented to two decimal places. UI=uncertainty interval. DALY=dis ability adjusted life-year.

#1: Absolute number of subarachnoid haemorrhage incident cases in the male 2050 is 376256(365053, 390529);

#2: Absolute number of subarachnoid haemorrhage dead cases in the male in 2050 is 191440(181327, 199511);

#3: Absolute number of subarachnoid haemorrhage incident cases in the female 2050 is 379248(370958, 392629);

#4: Absolute number of subarachnoid haemorrhage dead cases in the female 2050 is 186694(177662, 194123).


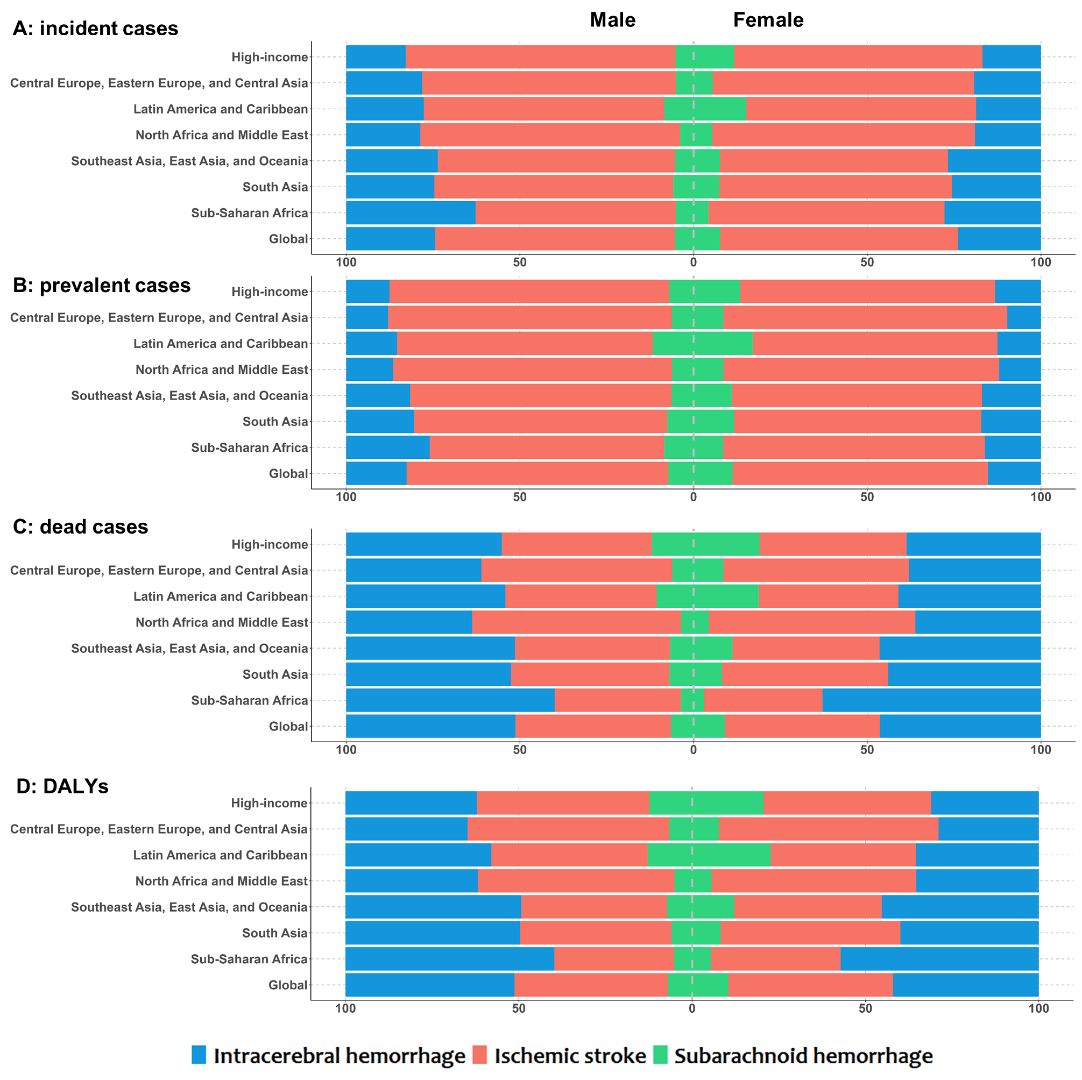


**Fig.S5 Proportion of the number of cases in different pathological types of stroke** **at the global level and the super-region level by sex in 2050.**

# Appendix 5. Burden of all strokes and their pathological types by age in different super-regions in 2050

**Table S4. Absolute number, crude rates, and age-standardised rates per 100 000 people per year of incident and prevalent strokes, deaths from stroke and DALYs due to stroke in 2050 and percentage change globally for 2021–2050, by age and pathological type of stroke.**

|  | **2021** | | | **2050** | | | **Relative change (percentages)** | | |
| --- | --- | --- | --- | --- | --- | --- | --- | --- | --- |
|  | **Number** | **Crude rate** | **Age-standardised Rate** | **Number** | **Crude rate** | **Age-standardised Rate** | **Number** | **Crude rate** | **Age-standardised Rate** |
| **15-59 years** | | | | | | | | | |
| **Incidence** |  |  |  |  |  |  |  |  |  |
| Intracerebral haemorrhage | 1.18 | 24.86 | 22.9 | 0.99  (0.93, 1.04) | 18.40  (17.38, 19.41) | 18.34  (17.65, 19.54) | -16.61  (-21.2, -12.03) | -25.99  (-30.09, -21.92) | -19.91  (-22.93, -14.67) |
| Ischaemic stroke | 1.97 | 41.42 | 39.07 | 1.93  (1.84, 2.02) | 36.04  (34.33, 37.76) | 35.53  (34.16, 37.06) | -1.95  (-6.58, 2.74) | -12.99  (-17.12, -8.84) | -9.06  (-12.57, -5.14) |
| Subarachnoid haemorrhage | 0.37 | 7.87 | 8.19 | 0.39  (0.38, 0.40) | 7.23  (7.12, 7.42) | 7.71  (7.65, 7.78) | 3.59  (2.06, 6.29) | -8.13  (-9.53, -5.72) | -5.86  (-6.59, -5.01) |
| All stroke | 3.53 | 74.14 | 70.16 | 3.31  (3.16, 3.46) | 61.66  (58.84, 64.58) | 61.58  (59.45, 64.39) | -6.23  (-10.48, -1.98) | -16.83  (-20.64, -12.89) | -12.23  (-15.27, -8.22) |
| **Prevalence** |  |  |  |  |  |  |  |  |  |
| Intracerebral haemorrhage | 9.75 | 204.83 | 191.59 | 9.63  (9.42, 9.85) | 179.62  (175.7, 183.75) | 179.40  (176.49, 183.65) | -1.18  (-3.34, 1.09) | -12.31  (-14.22, -10.29) | -6.36  (-7.88, -4.14) |
| Ischaemic stroke | 23.54 | 494.65 | 494.94 | 26.15  (25.31, 27.05) | 487.72  (472, 504.41) | 496.65  (483.52, 510.56) | 11.11  (7.53, 14.92) | -1.4  (-4.58, 1.97) | 0.35  (-2.31, 3.16) |
| Subarachnoid haemorrhage | 4.48 | 94.09 | 104.28 | 5.15  (5.02, 5.3) | 96.12  (93.53, 98.9) | 105.87  (104.43, 107.26) | 15.12  (12.02, 18.45) | 2.16  (-0.6, 5.11) | 1.52  (0.14, 2.86) |
| All stroke | 37.2 | 781.8 | 787.99 | 41.09  (39.92, 42.67) | 766.23  (744.54, 795.71) | 781.22  (761.36, 803.79) | 10.46  (7.31, 14.70) | -1.99  (-4.77, 1.78) | -0.86  (-3.38, 2.01) |
| **Death** |  |  |  |  |  |  |  |  |  |
| Intracerebral haemorrhage | 0.73 | 15.26 | 14.12 | 0.59  (0.56, 0.63) | 11.06  (10.44, 11.7) | 11.54  (11.12, 12.04) | -18.31  (-22.93, -13.64) | -27.52  (-31.59, -23.33) | -18.27  (-21.25, -14.73) |
| Ischaemic stroke | 0.2 | 4.15 | 3.36 | 0.19  (0.17, 0.21) | 3.53  (3.22, 3.91) | 3.17  (2.99, 3.35) | -4.02  (-12.61, 6.08) | -14.94  (-22.41, -5.78) | -5.65  (-11.01, -0.3) |
| Subarachnoid haemorrhage | 0.12 | 2.47 | 2.5 | 0.12  (0.11, 0.13) | 2.25  (2.12, 2.36) | 2.22  (2.17, 2.27) | 2.57  (-3.1, 7.84) | -8.91  (-14.17, -4.45) | -11.2  (-13.2, -9.2) |
| All stroke | 1.04 | 21.88 | 19.99 | 0.90  (0.85, 0.96) | 16.84  (15.78, 17.96) | 16.94  (16.28, 17.66) | -13.46  (-18.27, -7.69) | -23.03  (-27.88, -17.92) | -15.26  (-18.56, -11.66) |
| **DALY** |  |  |  |  |  |  |  |  |  |
| Intracerebral haemorrhage | 30.83 | 647.97 | 611.47 | 22.91  (20.35, 25.28) | 427.31  (379.48, 471.49) | 485.15  (463.26, 510.89) | -25.68  (-34, -18) | -34.05  (-41.44, -27.24) | -20.66  (-24.24, -16.45) |
| Ischaemic stroke | 11.18 | 234.95 | 202.41 | 9.6  (7.92, 11.63) | 179.07  (147.78, 216.8) | 161.41  (139.84, 182.69) | -14.11  (-29.12, 3.99) | -23.78  (-37.1, -7.73) | -20.26  (-30.91, -9.74) |
| Subarachnoid haemorrhage | 5.66 | 119.01 | 123.59 | 5.53  (5.26, 5.79) | 103.05  (98.12, 108) | 117.28  (114.12, 120.96) | -2.42  (-7.09, 2.26) | -13.41  (-17.55, -9.25) | -5.11  (-7.66, -2.13) |
| All stroke | 47.67 | 1001.94 | 937.47 | 38.04  (33.53, 42.7) | 709.43  (625.38, 796.29) | 763.83  (717.22, 814.55) | -20.20  (-29.66, -10.43) | -29.19  (-37.58, -20.53) | -18.52  (-23.49, -13.11) |
| **60+ years** | | | | | | | | | |
| **Incidence** |  |  |  |  |  |  |  |  |  |
| Intracerebral haemorrhage | 1.46 | 136.07 | 143.64 | 1.96  (1.88, 2.06) | 95.83  (91.74, 100.47) | 121.56  (119.34, 124.03) | 34.93  (29.16, 41.46) | -29.57  (-32.58, -26.16) | -15.37  (-16.92, -13.65) |
| Ischaemic stroke | 3.88 | 362.41 | 322.66 | 6.27  (6.09, 6.49) | 305.92  (296.8, 316.69) | 316.52  (313.03, 320.1) | 61.73  (56.9, 67.42) | -15.59  (-18.1, -12.62) | -1.9  (-2.98, -0.79) |
| Subarachnoid haemorrhage | 0.21 | 19.26 | 18.08 | 0.37  (0.35, 0.38) | 17.86  (17.31, 18.73) | 17.48  (17.26, 17.72) | 77.69  (72.23, 86.28) | -7.27  (-10.12, -2.75) | -3.32  (-4.54, -1.99) |
| All stroke | 5.54 | 517.74 | 484.38 | 8.60  (8.32, 8.94) | 419.62  (405.85, 435.89) | 455.55  (449.63, 461.85) | 55.23  (50.18, 61.37) | -18.95  (-21.61, -15.81) | -5.95  (-7.17, -4.65) |
| **Prevalence** |  |  |  |  |  |  |  |  |  |
| Intracerebral haemorrhage | 5.37 | 501.67 | 461.62 | 9.91  (9.73, 10.11) | 483.49  (474.39, 493.27) | 449.43  (443.06, 455.15) | 84.65  (81.17, 88.38) | -3.62  (-5.44, -1.67) | -2.64  (-4.02, -1.4) |
| Ischaemic stroke | 34.54 | 3228.06 | 3119.51 | 62.42  (61.04, 63.94) | 3044.35  (2977.33, 3118.63) | 3204.87  (3174.92, 3241.5) | 80.69  (76.71, 85.09) | -5.69  (-7.77, -3.39) | 2.74  (1.78, 3.91) |
| Subarachnoid haemorrhage | 2.64 | 246.52 | 273.91 | 5.56  (5.36, 5.95) | 271.16  (261.65, 290.17) | 285.44  (282.82, 287.64) | 110.73  (103.34, 125.51) | 10  (6.14, 17.71) | 4.21  (3.25, 5.01) |
| All stroke | 54.26 | 5070.79 | 4741.46 | 102.96  (101.4, 104.81) | 5021.88  (4945.65, 5111.91) | 5026.41  (4980.61, 5066.32) | 89.75  (86.88, 93.16) | -0.96  (-2.47, 0.81) | 6.01  (5.04, 6.85) |
| **Death** |  |  |  |  |  |  |  |  |  |
| Intracerebral haemorrhage | 1.71 | 160.07 | 187.06 | 1.79  (1.67, 1.93) | 87.18  (81.36, 93.94) | 137.39  (132.16, 142.8) | 4.35  (-2.62, 12.43) | -45.54  (-49.17, -41.31) | -26.55  (-29.35, -23.66) |
| Ischaemic stroke | 1.55 | 145.06 | 153.14 | 2.03  (1.91, 2.20) | 99.15  (93.08, 107.08) | 121.26  (117.55, 124.69) | 30.95  (22.93, 41.43) | -31.65  (-35.83, -26.18) | -20.82  (-23.24, -18.58) |
| Subarachnoid haemorrhage | 0.15 | 13.9 | 12.52 | 0.26  (0.24, 0.27) | 12.59  (11.65, 13.2) | 11.42  (11.16, 11.69) | 73.55  (60.64, 82) | -9.42  (-16.19, -5.04) | -8.79  (-10.86, -6.63) |
| All stroke | 3.41 | 319.03 | 352.72 | 4.08  (3.82, 4.39) | 198.92  (186.1, 214.22) | 270.06  (260.87, 279.18) | 19.65  (12.02, 28.74) | -37.65  (-41.67, -32.85) | -23.44  (-26.04, -20.85) |
| **DALY** |  |  |  |  |  |  |  |  |  |
| Intracerebral haemorrhage | 38.19 | 3568.71 | 4266.47 | 46.26  (42.7, 49.66) | 2256.1  (2082.71, 2422.3) | 3095.52  (3004.21, 3182.48) | 21.12  (11.81, 30.04) | -36.78  (-41.64, -32.12) | -27.45  (-29.59, -25.41) |
| Ischaemic stroke | 37.39 | 3494.33 | 3635.64 | 58.34  (55.75, 61.54) | 2845.55  (2719.2, 3001.77) | 3292.39  (3233.15, 3354.3) | 56.02  (49.09, 64.58) | -18.57  (-22.18, -14.1) | -9.44  (-11.07, -7.74) |
| Subarachnoid haemorrhage | 3.69 | 344.95 | 321.87 | 6.93  (6.57, 7.26) | 337.98  (320.23, 354.05) | 321.05  (315.83, 325.81) | 87.72  (77.86, 96.64) | -2.02  (-7.17, 2.64) | -0.25  (-1.88, 1.22) |
| All stroke | 79.27 | 7407.99 | 8223.99 | 111.52  (105.02, 118.46) | 5439.63  (5122.14, 5778.12) | 6708.97  (6553.19, 6862.59) | 40.68  (32.48, 49.44) | -26.57  (-30.86, -22.00) | -18.42  (-20.32, -16.55) |

Notes: Absolute number are in millions. The denominator of crude incidence rate and age standardized incidence rate is per 100 000 people. Absolute numbers in millions, crude rates per 100 000 people, age-standardised rates per 100 000 people, and percentage change are presented to two decimal places. UI=uncertainty interval. DALY=dis ability adjusted life-years

**
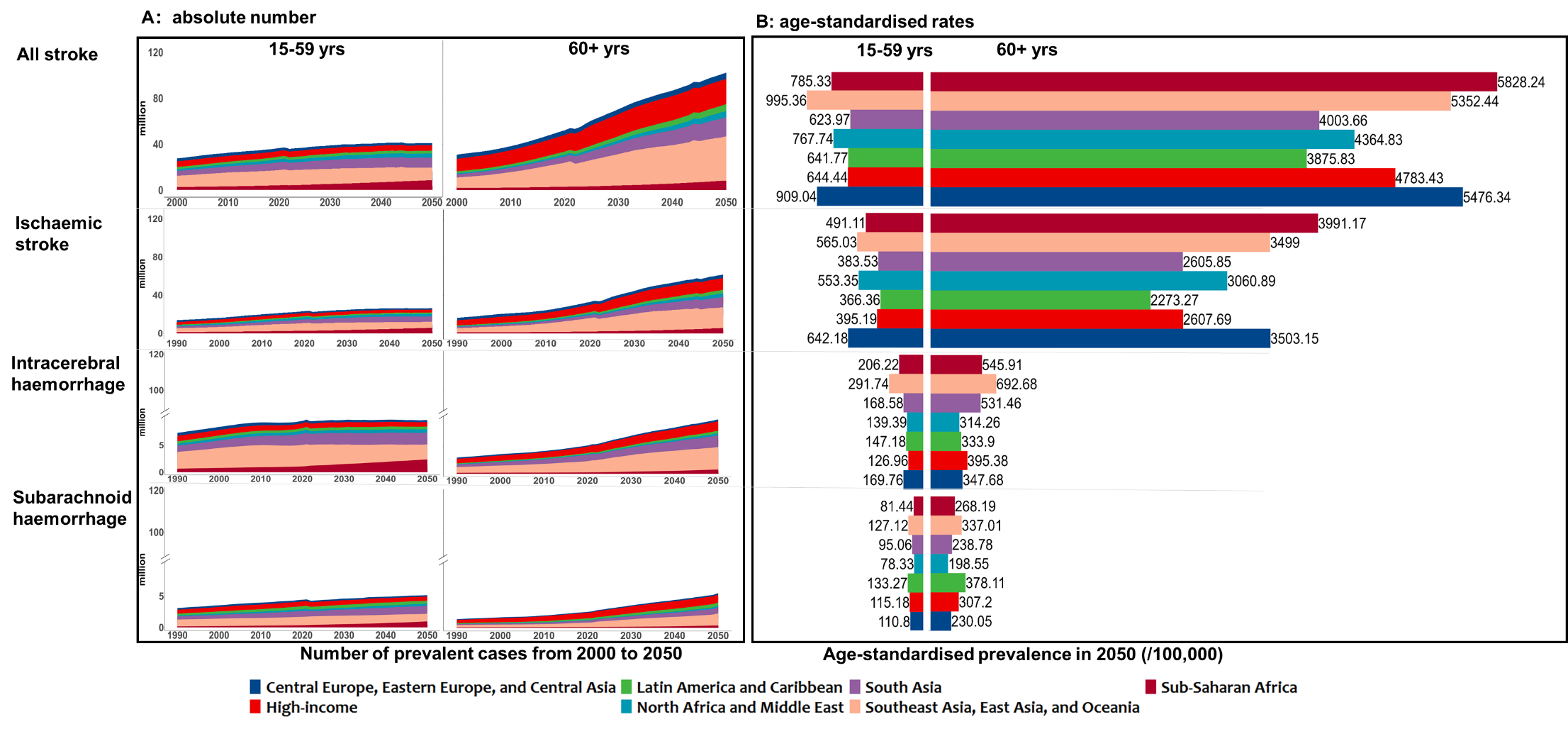
**

**Fig.S6 Predicted prevalent cases and age-standardised prevalence rates for all strokes by age group in seven GBD super-regions. A: Temporal trends in prevalent cases by age group (15-59 and 60+) in seven GBD super-regions from 2000 to 2050. B: Age-standardised prevalence rates in the seven GBD super-regions in 2050.**


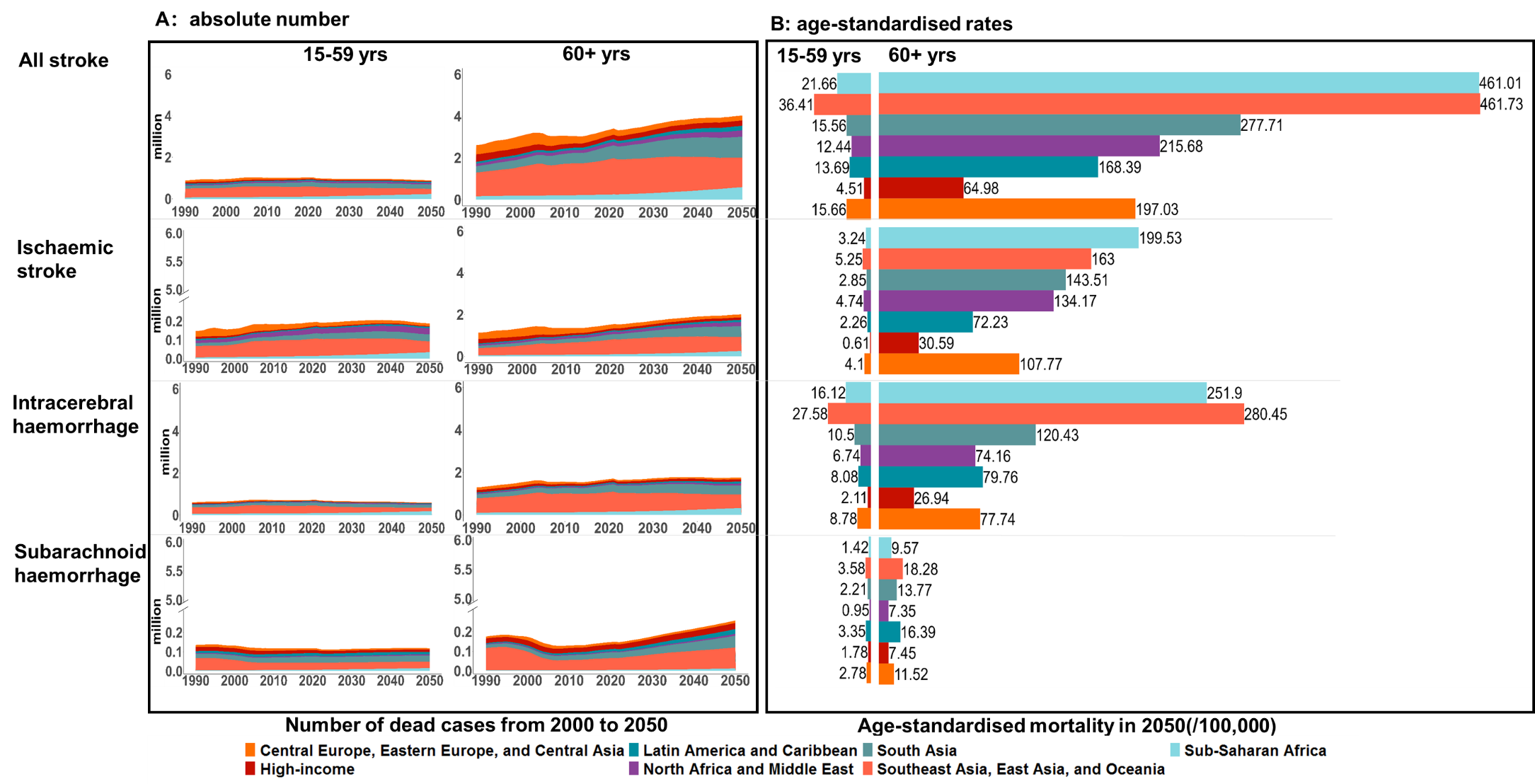


**Fig.S7 Predicted number of deaths and age-standardised death rates for all strokes by age group in seven GBD super-regions. A: Temporal trends in death cases by age group (15-59 and 60+) in seven GBD super-regions from 2000 to 2050. B: Age-standardised death rates in the seven GBD super-regions in 2050.**


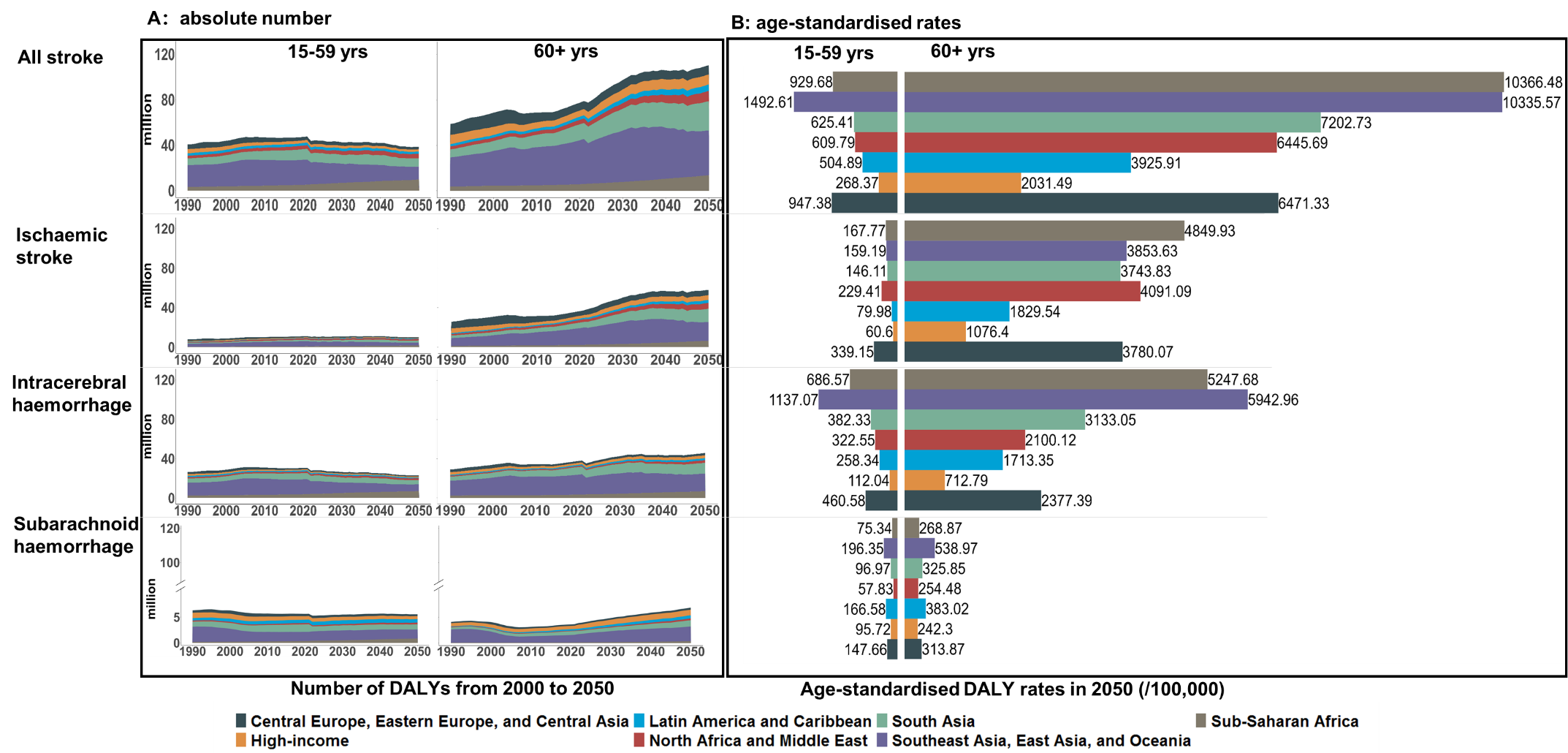


**Fig.S8 Predicted number of DALYs lost and age-standardised DALYs rates for all strokes by age group in seven GBD super-regions. A: Temporal trends in the number of DALYs lost by age group (15-59 and 60+) in seven GBD super-regions from 2000 to 2050. B: Age-standardised DALYs rates in the seven GBD super-regions in 2050.**

# Appendix 6. Burden of all strokes and their pathological types by socio-demographic index level and ages in 2050

**Table S5. Number of cases, crude rates and age-standardised rates per 100 000 population in 2050 and percentage change between 2021 and 2050 for stroke prevalence,** **by SDI quintile, for both sexes (95% uncertainty interval).**

|  | | **Intracerebral haemorrhage** | | **Ischaemic stroke** | | **Subarachnoid haemorrhage** | | **All stroke** | |
| --- | --- | --- | --- | --- | --- | --- | --- | --- | --- |
|  |  | **2050** | **Percentage change,**  **2021-2050** | **2050** | **Percentage change,**  **2021-2050** | **2050** | **Percentage change,**  **2021-2050** | **2050** | **Percentage change,**  **2021-2050** |
| Low SDI | Absolute number | 2.20  (2.15, 2.27) | 124.73  (119.1, 131.54) | 7.34  (7.11, 7.62) | 143.88  (135.98, 153) | 0.94  (0.91, 0.97) | 146.53  (138.11, 154.41) | 11.29  (10.88, 11.66) | 155.98  (146.67, 164.41) |
|  | Crude rate | 226.37  (220.69, 233.22) | 4.17  (1.55, 7.32) | 753.9  (729.49, 782.10) | 13.04  (9.38, 17.27) | 96.51  (93.21, 99.6) | 14.27  (10.36, 17.92) | 1158.79  (1116.62, 1196.95) | 18.65  (14.33, 22.56) |
|  | Age-standardized rate | 275.19  (267.38, 281.25) | -1.05  (-3.85, 1.14) | 976.83  (949.58, 999.57) | 3.35  (0.47, 5.75) | 112.94  (109.99, 115.57) | 4.96  (2.21, 7.39) | 1509.26  (1472.33, 1544.17) | 15.02  (12.2, 17.68) |
| Low-middle SDI | Absolute number | 6.08  (5.88, 6.32) | 38.88  (34.21, 44.33) | 24.38  (23.56, 25.23) | 117.35  (110.04, 124.91) | 3.05  (2.92, 3.18) | 73.17  (65.64, 80.98) | 36.66  (35.6, 37.77) | 99.75  (93.93, 105.76) |
|  | Crude rate | 232.15  (224.35, 241.26) | -3.74  (-6.97, 0.04) | 931.03  (899.7, 963.42) | 50.66  (45.59, 55.9) | 116.37  (111.31, 121.62) | 20.04  (14.81, 25.44) | 1400.08  (1359.3, 1442.2) | 38.46  (34.42, 42.62) |
|  | Age-standardized rate | 270.99  (264.54, 281.17) | -11.48  (-13.59, -8.15) | 1020.93  (997.49, 1041.89) | 4.38  (1.98, 6.52) | 134.88  (132.01, 138.06) | -4.80  (-6.83, -2.56) | 1567.58  (1543.71, 1602.77) | 24.16  (22.27, 26.95) |
| Middle SDI | Absolute number | 3.63  (3.53, 3.74) | 36.88  (32.88, 40.99) | 16.13  (15.77, 16.45) | 67.52  (63.83, 70.8) | 2.22  (2.18, 2.28) | 49.27  (46.64, 53.25) | 25.31  (24.9, 25.84) | 68.30  (65.58, 71.82) |
|  | Crude rate | 269.52  (261.64, 277.61) | 6.52  (3.4, 9.72) | 1196.04  (1169.7, 1219.52) | 30.36  (27.48, 32.91) | 164.68  (161.78, 169.07) | 16.16  (14.11, 19.25) | 1876.72  (1846.39, 1915.98) | 30.97  (28.85, 33.71) |
|  | Age-standardized rate | 212.73  (207.19, 220.14) | 2.55  (-0.12, 6.12) | 867.03  (845.86, 891.19) | 6.07  (3.48, 9.02) | 146.75  (143.9, 149.4) | 13.90  (11.69, 15.96) | 1382.84  (1346.05, 1420.73) | 14.25  (11.21, 17.38) |
| High-middle SDI | Absolute number | 5.27  (5.06, 5.51) | 4.12  (-0.2, 8.71) | 26.16  (25.14, 27.44) | 13.25  (8.81, 18.78) | 2.79  (2.56, 3.15) | 48.77  (36.42, 67.55) | 46.13  (43.99, 47.82) | 31.8  (25.67, 36.62) |
|  | Crude rate | 330.66  (316.95, 345.24) | 5.18  (0.82, 9.82) | 1640.29  (1576.01, 1720.31) | 14.41  (9.93, 19.99) | 175.16  (160.63, 197.28) | 50.29  (37.82, 69.26) | 2892.38  (2757.7, 2997.98) | 33.15  (26.95, 38.01) |
|  | Age-standardized rate | 198.42  (192.42, 203.95) | -14.16  (-16.76, -11.77) | 1000.37  (969.5, 1032.27) | -2.89  (-5.88, 0.21) | 136.26  (134.15, 138.74) | -3.86  (-5.34, -2.1) | 1530.27  (1491.14, 1571.7) | 18.40  (15.37, 21.6) |
| High SDI | Absolute number | 2.35  (2.3, 2.41) | 21.49  (18.55, 24.72) | 14.67  (13.89, 15.4) | 37.08  (29.82, 43.99) | 1.76  (1.71, 1.79) | 13.12  (10.33, 15.1) | 24.87  (24.03, 26.01) | 32.77  (28.31, 38.89) |
|  | Crude rate | 267.68  (261.2, 274.8) | 16.05  (13.24, 19.14) | 1668.84  (1580.43, 1752.98) | 30.94  (24, 37.54) | 199.81  (194.88, 203.31) | 8.06  (5.39, 9.95) | 2829.81  (2734.75, 2960.21) | 26.83  (22.57, 32.67) |
|  | Age-standardized rate | 175.28  (170.36, 181.19) | -2.66  (-5.4, 0.62) | 880.86  (854.52, 907.29) | -4.37  (-7.23, -1.5) | 149.14  (146.83, 151.64) | 1.88  (0.3, 3.59) | 1445.35  (1413.64, 1487.35) | 18.21  (15.62, 21.64) |

Notes: Absolute number are in millions. The denominator of crude incidence rate and age standardized incidence rate is per 100 000 people. Absolute numbers in millions, crude rates per 100 000 people, age-standardised rates per 100 000 people, and percentage change are presented to two decimal places. UI=uncertainty interval. DALY=dis ability adjusted life-year.

**Table S6. Number of cases, crude rates and age-standardised rates per 100 000 population in 2050 and percentage change between 2021 and 2050 for stroke death, by SDI quintile, for both sexes (95% uncertainty interval).**

|  | | **Intracerebral haemorrhage** | | **Ischaemic stroke** | | **Subarachnoid haemorrhage** | | **All stroke** | |
| --- | --- | --- | --- | --- | --- | --- | --- | --- | --- |
|  |  | **2050** | **Percentage change,**  **2021-2050** | **2050** | **Percentage change,**  **2021-2050** | **2050** | **Percentage change,**  **2021-2050** | **2050** | **Percentage change,**  **2021-2050** |
| Low SDI | Absolute number | 0.39  (0.37, 0.41) | 114.31  (102.77, 123.39) | 0.22  (0.21, 0.23) | 160.76  (153.28, 170.33) | 0.03  (0.02, 0.03)^#1^ | 122.72  (112.74, 135.72) | 0.64  (0.61, 0.66) | 128.55  (118.28, 137.94) |
|  | Crude rate | 40.47  (38.29, 42.19) | -0.67  (-6.02, 3.54) | 22.32  (21.68, 23.14) | 20.86  (17.4, 25.3) | 2.61  (2.49, 2.76) | 3.23  (-1.39, 9.26) | 65.4  (62.47, 68.09) | 5.93  (1.18, 10.29) |
|  | Age-standardized rate | 66.78  (64.12, 69.74) | -11.35  (-14.87, -7.41) | 38.46  (37.35, 39.76) | 0.44  (-2.45, 3.83) | 3.94  (3.85, 4.08) | -2.13  (-4.33, 1.43) | 109.17  (105.32, 113.59) | -7.20  (-10.47, -3.44) |
| Low-middle SDI | Absolute number | 0.86  (0.77, 0.96) | 26.03  (13.19, 41.55) | 0.81  (0.77, 0.87) | 91.52  (81.63, 105.08) | 0.11  (0.11, 0.12)^#2^ | 49.82  (39.48, 59.53) | 1.78  (1.64, 1.95) | 51.10  (39.48, 65.55) |
|  | Crude rate | 32.7  (29.37, 36.73) | -12.64  (-21.54, -1.88) | 31.01  (29.4, 33.2) | 32.75  (25.89, 42.15) | 4.31  (4.01, 4.59) | 3.85  (-3.32, 10.58) | 68.02  (62.79, 74.52) | 4.74  (-3.32, 14.75) |
|  | Age-standardized rate | 48.7  (46.44, 50.8) | -26.68  (-30.09, -23.52) | 32.37  (30.72, 33.9) | -8.00  (-12.71, -3.66) | 4.51  (4.37, 4.64) | -16.61  (-19.08, -14.22) | 85.58  (81.53, 89.34) | -20.03  (-23.82, -16.52) |
| Middle SDI | Absolute number | 0.51  (0.47, 0.55) | 5.76  (-0.82, 14.25) | 0.47  (0.44, 0.51) | 60.12  (47.86, 73.39) | 0.08  (0.08, 0.08)^#3^ | 51.93  (45.98, 58.15) | 1.06  (0.99, 1.14) | 28.12  (19.55, 38.17) |
|  | Crude rate | 37.47  (35.14, 40.48) | -17.70  (-22.82, -11.09) | 34.94  (32.27, 37.84) | 24.60  (15.06, 34.93) | 5.99  (5.76, 6.24) | 18.23  (13.6, 23.07) | 78.4  (73.16, 84.56) | -0.30  (-6.97, 7.52) |
|  | Age-standardized rate | 24.92  (23.2, 26.19) | -32.37  (-37.03, -28.92) | 19.34  (18.62, 20.23) | -24.50  (-27.34, -21.04) | 3.87  (3.77, 3.99) | -1.50  (-4.05, 1.56) | 48.13  (45.59, 50.41) | -27.51  (-31.34, -24.07) |
| High-middle SDI | Absolute number | 0.49  (0.42, 0.58) | -50.31  (-57.06, -41.3) | 0.59  (0.51, 0.71) | -28.16  (-38.73, -13.99) | 0.12  (0.1, 0.13) | 31.84  (16.74, 44.4) | 1.20  (1.03, 1.42) | -36.81  (-45.62, -25.39) |
|  | Crude rate | 30.64  (26.47, 36.19) | -49.8  (-56.62, -40.7) | 37.19  (31.72, 44.52) | -27.42  (-38.1, -13.11) | 7.38  (6.54, 8.09) | 33.19  (17.93, 45.88) | 75.21  (64.73, 88.8) | -36.16  (-45.06, -24.62) |
|  | Age-standardized rate | 15.27  (14.55, 16.29) | -48.11  (-50.53, -44.61) | 15.45  (14.65, 16.71) | -47.01  (-49.76, -42.7) | 3.62  (3.51, 3.75) | -19.41  (-21.84, -16.43) | 34.33  (32.71, 36.75) | -45.56  (-48.13, -41.72) |
| High SDI | Absolute number | 0.11  (0.10, 0.12) | 4.06  (-3.28, 10.81) | 0.11  (0.11, 0.13)^#4^ | 2.56  (-3.23, 15.94) | 0.04  (0.04, 0.04)^#5^ | 11.36  (7.98, 15.42) | 0.26  (0.25, 0.29) | 4.42  (-1.68, 13.71) |
|  | Crude rate | 12.5  (11.62, 13.31) | -0.6  (-7.61, 5.84) | 12.86  (12.13, 14.54) | -2.04  (-7.57, 10.75) | 4.46  (4.33, 4.63) | 6.37  (3.14, 10.25) | 29.82  (28.08, 32.48) | -0.25  (-6.08, 8.62) |
|  | Age-standardized rate | 6.64  (6.29, 7.05) | -37.46  (-40.72, -33.53) | 6.65  (6.25, 7.14) | -47.53  (-50.64, -43.66) | 2.73  (2.63, 2.82) | -13.83  (-16.78, -11.03) | 16.01  (15.18, 17.01) | -39.46  (-42.61, -35.69) |

Notes: Absolute number are in millions. The denominator of crude incidence rate and age standardized incidence rate is per 100 000 people. Absolute numbers in millions, crude rates per 100 000 people, age-standardised rates per 100 000 people, and percentage change are presented to two decimal places. UI=uncertainty interval. DALY=dis ability adjusted life-year.

#1: Absolute number of subarachnoid haemorrhage dead cases in low SDI countries in 2050 is 25426(24287, 26910);

#2: Absolute number of subarachnoid haemorrhage dead cases in low-middle SDI countries in 2050 is 112877(105082, 120193);

#3: Absolute number of subarachnoid haemorrhage dead cases in middle SDI countries in 2050 is 80797(77631, 84106);

#4: Absolute number of ischemic haemorrhage dead cases in high SDI countries in 2050 is 113020(106639, 127772);

#5: Absolute number of subarachnoid haemorrhage dead cases in high SDI countries in 2050 is 39220(38030, 40650). **Table S7. Number of cases, crude rates and age-standardised rates per 100 000 population in 2050 and percentage change between 2021 and 2050 for stroke DALYs, by SDI quintile, for both sexes (95% uncertainty interval).**

|  | | **Intracerebral haemorrhage** | | **Ischaemic stroke** | | **Subarachnoid haemorrhage** | | **All stroke** | |
| --- | --- | --- | --- | --- | --- | --- | --- | --- | --- |
|  |  | **2050** | **Percentage change,**  **2021-2050** | **2050** | **Percentage change,**  **2021-2050** | **2050** | **Percentage change,**  **2021-2050** | **2050** | **Percentage change,**  **2021-2050** |
| Low SDI | Absolute number | 10.77  (10.05, 11.53) | 90.14  (77.37, 103.56) | 6.19  (5.65, 6.65) | 160.93  (137.94, 180.31) | 1.09  (1.02, 1.17) | 133.03  (118.6, 149.78) | 18.05  (16.72, 19.35) | 112.26  (96.54, 127.52) |
|  | Crude rate | 1105.79  (1031.53, 1183.79) | -11.87  (-17.79, -5.65) | 636.01  (579.99, 683.25) | 20.94  (10.29, 29.93) | 111.70  (104.79, 119.73) | 8.01  (1.32, 15.77) | 1853.51  (1716.3, 1986.77) | -1.62  (-8.9, 5.46) |
|  | Age-standardized rate | 1571.29  (1510.31, 1627.62) | -24.67  (-27.59, -21.96) | 928.36  (880.18, 971.79) | -5.89  (-10.77, -1.49) | 142.03  (134.85, 148.61) | -3.24  (-8.13, 1.25) | 2641.68  (2525.35, 2748.02) | -17.93  (-21.55, -14.63) |
| Low-middle SDI | Absolute number | 23.89  (20.99, 26.19) | 20.04  (5.47, 31.6) | 22.94  (21.09, 24.61) | 102.13  (85.84, 116.87) | 3.23  (2.87, 3.49) | 16.62  (3.47, 26) | 50.07  (44.95, 54.3) | 47.14  (32.12, 59.59) |
|  | Crude rate | 912.3  (801.61, 1000.18) | -16.8  (-26.89, -8.78) | 876.05  (805.45, 939.93) | 40.11  (28.82, 50.32) | 123.45  (109.53, 133.38) | -19.17  (-28.28, -12.67) | 1911.8  (1716.59, 2073.49) | 1.99  (-8.42, 10.62) |
|  | Age-standardized rate | 1417.71  (1359.05, 1479.71) | -25.56  (-28.64, -22.3) | 871.44  (833.66, 906.46) | -8.02  (-12, -4.32) | 178.76  (171.67, 187.7) | -12.06  (-15.54, -7.66) | 2467.91  (2364.38, 2573.87) | -19.22  (-22.61, -15.75) |
| Middle SDI | Absolute number | 15.73  (14.72, 16.49) | 9.94  (2.85, 15.25) | 14.65  (13.67, 15.6) | 76.35  (64.48, 87.74) | 3.03  (2.92, 3.18) | 52.40  (46.9, 60.23) | 33.41  (31.3, 35.27) | 35.8  (27.22, 43.36) |
|  | Crude rate | 1166.46  (1091.26, 1222.77) | -14.45  (-19.96, -10.32) | 1086.64  (1013.52, 1156.81) | 37.23  (28, 46.09) | 224.33  (216.23, 235.87) | 18.59  (14.31, 24.69) | 2477.43  (2321.01, 2615.45) | 5.67  (-1, 11.56) |
|  | Age-standardized rate | 756.35  (710.76, 797.24) | -28.73  (-33.03, -24.88) | 559.64  (519.3, 602.49) | -20.41  (-26.14, -14.31) | 159.82  (152.96, 166.25) | 7.80  (3.18, 12.14) | 1475.8  (1383.02, 1565.97) | -22.84  (-27.69, -18.13) |
| High-middle SDI | Absolute number | 15.33  (12.69, 17.9) | -40.94  (-51.09, -31.04) | 18.75  (16.31, 20.91) | -16.25  (-27.17, -6.6) | 3.62  (3.26, 3.95) | 27.87  (15.44, 39.76) | 37.70  (32.26, 42.76) | -26.33  (-36.95, -16.44) |
|  | Crude rate | 961.04  (795.89, 1122.04) | -40.33  (-50.59, -30.34) | 1175.6  (1022.32, 1310.98) | -15.39  (-26.42, -5.65) | 226.67  (204.63, 247.74) | 29.18  (16.63, 41.19) | 2363.30  (2022.84, 2680.76) | -25.58  (-36.3, -15.58) |
|  | Age-standardized rate | 618.44  (583.57, 655.63) | -28.52  (-32.55, -24.22) | 660.78  (613.67, 709.82) | -19.19  (-24.95, -13.19) | 156.57  (152.49, 160.21) | -8.18  (-10.58, -6.05) | 1435.79  (1349.73, 1525.65) | -22.53  (-27.17, -17.68) |
| High SDI | Absolute number | 3.59  (3.26, 4.02) | 22.38  (11.04, 37.01) | 5.34  (4.67, 6.68) | 39.55  (22.05, 74.47) | 1.55  (1.46, 1.65) | 24.50  (17.82, 32.55) | 10.49  (9.4, 12.35) | 30.91  (17.36, 54.22) |
|  | Crude rate | 409.09  (371.20, 457.99) | 16.9  (6.07, 30.87) | 607.97  (531.74, 760.11) | 33.30  (16.58, 66.65) | 176.14  (166.7, 187.52) | 18.92  (12.55, 26.61) | 1193.20  (1069.63, 1405.62) | 25.05  (12.10, 47.31) |
|  | Age-standardized rate | 265.41  (242.69, 283.97) | -16.02  (-23.21, -10.15) | 361.38  (332.68, 383.75) | -12.05  (-19.04, -6.61) | 128.49  (124.76, 132.03) | 4.58  (1.54, 7.46) | 755.28  (700.13, 799.75) | -11.12  (-17.61, -5.89) |

Notes: Absolute number are in millions. The denominator of crude incidence rate and age standardized incidence rate is per 100 000 people. Absolute numbers in millions, crude rates per 100 000 people, age-standardised rates per 100 000 people, and percentage change are presented to two decimal places. UI=uncertainty interval. DALY=dis ability adjusted life-year.

**Table S8. Number of cases, crude and age-standardised rates per 100 000 population in 2050 and percentage change between 2021 and 2050 for stroke incidence, by SDI quintile,** **for both sexes in people aged 15-59 years (95% uncertainty interval).**

|  | | **Intracerebral haemorrhage** | | **Ischaemic stroke** | | **Subarachnoid haemorrhage** | | **All stroke** | |
| --- | --- | --- | --- | --- | --- | --- | --- | --- | --- |
|  |  | **2050** | **Percentage change,**  **2021-2050** | **2050** | **Percentage change,**  **2021-2050** | **2050** | **Percentage change,**  **2021-2050** | **2050** | **Percentage change,**  **2021-2050** |
| Low SDI | Absolute number | 0.21  (0.20, 0.22) | 101.32  (88.02, 114.82) | 0.30  (0.28, 0.32) | 118.92  (102.33, 133.49) | 0.04  (0.04, 0.05)^#1^ | 93.32  (89.33, 96.81) | 0.56  (0.52, 0.59) | 107.41  (92.59, 118.52) |
|  | Crude rate | 24.10  (22.51, 25.72) | -3.91  (-10.25, 2.55) | 35.02  (32.37, 37.35) | 4.51  (-3.4, 11.46) | 5.12  (5.01, 5.21) | -7.75  (-9.73, -6.13) | 64.24  (59.89, 68.28) | 0.16  (-6.63, 6.45) |
|  | Age-standardized rate | 26.31  (25.05, 27.75) | -11.98  (-16.19, -7.16) | 34.45  (32.60, 36.47) | -2.57  (-7.81, 3.14) | 5.88  (5.77, 5.99) | -2.97  (-4.79, -1.16) | 66.63  (63.42, 70.21) | -6.56  (-11.06, -1.54) |
| Low-middle SDI | Absolute number | 0.38  (0.34, 0.42) | -5.75  (-15.31, 4.24) | 0.72  (0.65, 0.80) | 49.17  (32.79, 64.74) | 0.14  (0.14, 0.14)^#2^ | 16.64  (13.67, 18.97) | 1.25  (1.13, 1.37) | 23.76  (11.88, 35.64) |
|  | Crude rate | 18.54  (16.66, 20.5) | -27.8  (-35.12, -20.17) | 35.03  (31.18, 38.69) | 14.25  (1.7, 26.19) | 6.75  (6.58, 6.89) | -10.71  (-12.96, -8.86) | 60.32  (54.42, 66.08) | -5.62  (-14.85, 3.4) |
|  | Age-standardized rate | 28.55  (26.99, 30.34) | -15.96  (-20.55, -10.69) | 39.31  (37.30, 41.5) | -0.25  (-5.35, 5.3) | 7.50  (7.43, 7.58) | -15.73  (-16.52, -14.83) | 75.36  (71.72, 79.42) | -8.40  (-12.82, -3.46) |
| Middle SDI | Absolute number | 0.20  (0.18, 0.22) | -18.61  (-24.55, -11.27) | 0.37  (0.35, 0.4) | -0.68  (-8.06, 6.56) | 0.09  (0.09, 0.09) ^#3^ | 6.49  (4.29, 10.57) | 0.66  (0.62, 0.71) | -5.71  (-11.43, 1.43) |
|  | Crude rate | 20.49  (19, 22.34) | -25.65  (-31.06, -18.94) | 38.55  (35.69, 41.36) | -9.27  (-16, -2.66) | 9.28  (9.09, 9.63) | -2.73  (-4.72, 0.94) | 68.32  (63.77, 73.33) | -14.16  (-19.88, -7.87) |
|  | Age-standardized rate | 16.05  (14.41, 17.59) | -21.63  (-29.64, -14.11) | 31.76  (29.14, 34.41) | -8.29  (-15.85, -0.64) | 8.45  (8.34, 8.58) | 6.56  (5.17, 8.2) | 56.27  (51.89, 60.57) | -10.74  (-17.69, -3.92) |
| High-middle SDI | Absolute number | 0.16  (0.12, 0.19) | -55.97  (-66.42, -47.93) | 0.37  (0.32, 0.41) | -49.60  (-55.5, -44.16) | 0.07  (0.07, 0.08)^#4^ | -22.40  (-25.4, -15.76) | 0.6  (0.51, 0.67) | -49.15  (-56.78, -43.22) |
|  | Crude rate | 17.15  (13.08, 20.28) | -41.00  (-55.01, -30.24) | 39.80  (35.14, 44.1) | -32.47  (-40.38, -25.18) | 7.64  (7.35, 8.29) | 3.95  (0.00, 12.79) | 64.59  (55.56, 72.67) | -32.27  (-41.74, -23.79) |
|  | Age-standardized rate | 14.85  (13.35, 16.57) | -30.02  (-37.09, -21.91) | 40.63  (37.55, 43.73) | -13.77  (-20.31, -7.19) | 8.6  (8.41, 8.84) | -6.83  (-8.88, -4.23) | 64.08  (59.31, 69.14) | -17.39  (-23.54, -10.87) |
| High SDI | Absolute number | 0.04  (0.03, 0.05) | -36.27  (-46.12, -25.78) | 0.17  (0.15, 0.18) | -26.49  (-33.47, -19.43) | 0.04  (0.04, 0.04)^#5^ | -19.5  (-21.75, -17.99) | 0.25  (0.23, 0.27) | -26.47  (-32.35, -20.59) |
|  | Crude rate | 7.58  (6.41, 8.83) | -30.14  (-40.92, -18.62) | 31.14  (28.18, 34.13) | -19.45  (-27.11, -11.72) | 8.07  (7.85, 8.23) | -11.8  (-14.21, -10.05) | 46.79  (42.44, 51.18) | -20.25  (-27.66, -12.77) |
|  | Age-standardized rate | 8.13  (7.29, 9.23) | -29.12  (-36.44, -19.53) | 33.75  (30.97, 36.5) | -16.95  (-23.79, -10.19) | 8.41  (8.19, 8.6) | -7.68  (-10.10, -5.60) | 50.28  (46.44, 54.33) | -17.88  (-24.15, -11.27) |

Notes: Absolute number are in millions. The denominator of crude incidence rate and age standardized incidence rate is per 100 000 people. Absolute numbers in millions, crude rates per 100 000 people, age-standardised rates per 100 000 people, and percentage change are presented to two decimal places. UI=uncertainty interval. DALY=dis ability adjusted life-year.

#1: Absolute number of subarachnoid haemorrhage incident cases among 15-59 years old population in low SDI countries in 2050 is 44512(43593, 45316);

#2: Absolute number of subarachnoid haemorrhage incident cases among 15-59 years old population in low-middle SDI countries in 2050 is 139671(136115, 142472);

#3: Absolute number of subarachnoid haemorrhage incident cases among 15-59 years old population in middle SDI countries in 2050 is 89724(87871, 93159);

#4: Absolute number of subarachnoid haemorrhage incident cases among 15-59 years old population in high-middle SDI countries in 2050 is 70514(67789, 76549);

#5: Absolute number of subarachnoid haemorrhage incident cases among 15-59 years old population in high SDI countries in 2050 is 43270(42060, 44086).

**Table S9. Number of cases, crude rates and age-standardised rates per 100 000 population in 2050 and percentage change between 2021 and 2050 for stroke prevalence, by SDI quintile, for both sexes in people aged 15-59 years (95% uncertainty interval).**

|  | | **Intracerebral haemorrhage** | | **Ischaemic stroke** | | **Subarachnoid haemorrhage** | | **All stroke** | |
| --- | --- | --- | --- | --- | --- | --- | --- | --- | --- |
|  |  | **2050** | **Percentage change,**  **2021-2050** | **2050** | **Percentage change,**  **2021-2050** | **2050** | **Percentage change,**  **2021-2050** | **2050** | **Percentage change,**  **2021-2050** |
| Low SDI | Absolute number | 1.68  (1.62, 1.74) | 112.87  (105.74, 120.57) | 3.72  (3.44, 3.92) | 110.67  (94.65, 121.94) | 0.67  (0.65, 0.70) | 137.12  (127.47, 146.39) | 5.91  (5.60, 6.24) | 119.7  (108.18, 131.97) |
|  | Crude rate | 192.71  (186.26, 199.69) | 1.61  (-1.79, 5.29) | 428.43  (395.86, 451.36) | 0.57  (-7.08, 5.95) | 77.44  (74.29, 80.47) | 13.2  (8.6, 17.63) | 679.40  (644.01, 718.25) | 4.76  (-0.7, 10.75) |
|  | Age-standardized rate | 217.18  (210.54, 223.67) | 0.26  (-2.8, 3.26) | 457.85  (432.44, 482.74) | 1.55  (-4.09, 7.07) | 82.45  (79.45, 85.25) | 10.08  (6.07, 13.82) | 762.15  (732.43, 796.03) | 13.54  (9.11, 18.59) |
| Low-middle SDI | Absolute number | 3.43  (3.25, 3.61) | 15.55  (9.38, 21.59) | 9.40  (8.82, 10.06) | 53.57  (44.03, 64.23) | 1.84  (1.72, 1.92) | 42.07  (33.16, 47.91) | 14.02  (13.30, 14.92) | 35.46  (28.5, 44.15) |
|  | Crude rate | 165.90  (157.05, 174.58) | -11.51  (-16.23, -6.88) | 454.6  (426.37, 486.16) | 17.61  (10.31, 25.78) | 88.94  (83.37, 92.60) | 8.8  (1.98, 13.27) | 677.90  (642.94, 721.37) | 3.73  (-1.62, 10.39) |
|  | Age-standardized rate | 220.51  (213.92, 229.81) | -9.91  (-12.6, -6.11) | 523.09  (501.34, 544.26) | 5.68  (1.29, 9.96) | 103.65  (100.95, 107.34) | -2.94  (-5.47, 0.52) | 846.67  (817.57, 882.88) | 27.06  (22.69, 32.49) |
| Middle SDI | Absolute number | 1.98  (1.92, 2.06) | 3.75  (0.28, 7.69) | 5.02  (4.84, 5.27) | 11.03  (7.04, 16.57) | 1.08  (1.05, 1.11) | 8.8  (5.68, 11.64) | 8.29  (8.04, 8.59) | 12.03  (8.65, 16.08) |
|  | Crude rate | 205.20  (198.33, 213.00) | -5.24  (-8.41, -1.63) | 519.51  (500.81, 545.39) | 1.42  (-2.23, 6.47) | 111.77  (108.57, 114.68) | -0.62  (-3.47, 1.96) | 857.07  (831.2, 888.09) | 2.3  (-0.79, 6) |
|  | Age-standardized rate | 176.10  (170.08, 183.20) | 2.65  (-0.86, 6.79) | 475.63  (452.75, 498.96) | 8.87  (3.64, 14.22) | 116.72  (113.92, 119.44) | 15.26  (12.49, 17.94) | 773.93  (739.11, 811.71) | 14.82  (9.65, 20.42) |
| High-middle SDI | Absolute number | 1.76  (1.63, 1.88) | -43.82  (-47.89, -39.93) | 5.09  (4.65, 5.46) | -32.45  (-38.36, -27.55) | 0.96  (0.89, 1.03) | -17.23  (-23.25, -11.21) | 8.31  (7.56, 9.15) | -29.46  (-35.82, -22.33) |
|  | Crude rate | 190.77  (176.93, 203.97) | -24.73  (-30.19, -19.52) | 551.64  (503.37, 591.69) | -9.51  (-17.43, -2.94) | 103.9  (96.34, 111.46) | 10.89  (2.82, 18.95) | 900.01  (819.12, 991.97) | -5.56  (-14.05, 4.09) |
|  | Age-standardized rate | 164.16  (158.1, 169.3) | -17.36  (-20.41, -14.77) | 580.66  (548.87, 618.06) | -2.56  (-7.9, 3.71) | 111.64  (109.4, 114.05) | -7.9  (-9.75, -5.91) | 841.24  (804.38, 885.45) | 13.61  (8.63, 19.58) |
| High SDI | Absolute number | 0.77  (0.74, 0.81) | -11.19  (-15.42, -7.34) | 3.02  (2.84, 3.16) | -10.43  (-15.65, -6.22) | 0.61  (0.59, 0.63) | -12.93  (-15.75, -10.1) | 4.53  (4.33, 4.74) | -8.11  (-12.17, -3.85) |
|  | Crude rate | 144.51  (137.62, 150.78) | -2.69  (-7.33, 1.53) | 562.66  (529.88, 589.15) | -1.86  (-7.58, 2.76) | 114.59  (110.88, 118.31) | -4.6  (-7.69, -1.51) | 845.26  (807.39, 885.27) | 0.71  (-3.8, 5.48) |
|  | Age-standardized rate | 134.24  (129.15, 140.36) | -7.4  (-10.91, -3.18) | 487.25  (461.62, 513.31) | -9.94  (-14.68, -5.12) | 119.89  (116.96, 122.41) | -1.41  (-3.82, 0.66) | 732.62  (693, 767.72) | 10.49  (4.52, 15.78) |

Notes: Absolute number are in millions. The denominator of crude incidence rate and age standardized incidence rate is per 100 000 people. Absolute numbers in millions, crude rates per 100 000 people, age-standardised rates per 100 000 people, and percentage change are presented to two decimal places. UI=uncertainty interval. DALY=dis ability adjusted life-year.

**Table S10. Number of cases, crude rates and age-standardised rates per 100 000 population in 2050 and percentage change between 2021 and 2050 for stroke deaths, by SDI quintile, for both sexes in people aged 15-59 years (95% uncertainty interval).**

|  | | **Intracerebral haemorrhage** | | **Ischaemic stroke** | | **Subarachnoid haemorrhage** | | **All stroke** | |
| --- | --- | --- | --- | --- | --- | --- | --- | --- | --- |
|  |  | **2050** | **Percentage change,**  **2021-2050** | **2050** | **Percentage change,**  **2021-2050** | **2050** | **Percentage change,**  **2021-2050** | **2050** | **Percentage change,**  **2021-2050** |
| Low SDI | Absolute number | 0.14  (0.13, 0.15) | 94.9  (83.9, 109.08) | 0.03  (0.02, 0.03) | 111.51  (97.94, 129.81) | 0.01  (0.01, 0.01)^#1^ | 96.36  (85.34, 118.05) | 0.18  (0.17, 0.19) | 100.00  (88.89, 111.11) |
|  | Crude rate | 15.63  (14.75, 16.77) | -6.96  (-12.2, -0.18) | 2.96  (2.77, 3.22) | 1.02  (-5.46, 9.9) | 1.55  (1.46, 1.72) | -6.06  (-11.52, 4.24) | 20.14  (18.98, 21.71) | -5.84  (-11.27, 1.5) |
|  | Age-standardized rate | 20.38  (19.49, 21.71) | -1.45  (-5.75, 4.98) | 3.96  (3.64, 4.31) | 18.56  (8.98, 29.04) | 2.05  (1.97, 2.15) | -1.91  (-5.74, 2.87) | 26.39  (25.11, 28.17) | 1.11  (-3.79, 7.93) |
| Low-middle SDI | Absolute number | 0.23  (0.21, 0.25) | 4.49  (-3.38, 14.63) | 0.07  (0.07, 0.08)^#2^ | 42.77  (28.24, 58.98) | 0.04  (0.04, 0.04)^#3^ | 5.66  (-6.12, 13.08) | 0.35  (0.32, 0.38) | 12.9  (3.23, 22.58) |
|  | Crude rate | 11.23  (10.39, 12.32) | -20.01  (-26, -12.25) | 3.54  (3.18, 3.94) | 9.26  (-1.85, 21.6) | 2.02  (1.79, 2.16) | -18.88  (-28.11, -13.25) | 16.79  (15.36, 18.42) | -15.07  (-22.31, -6.83) |
|  | Age-standardized rate | 17.44  (16.58, 18.46) | -21.34  (-25.21, -16.73) | 4.31  (3.97, 4.6) | 6.95  (-1.49, 14.14) | 2.45  (2.38, 2.53) | -22.71  (-24.92, -20.19) | 24.2  (22.93, 25.59) | -17.60  (-21.93, -12.87) |
| Middle SDI | Absolute number | 0.13  (0.13, 0.15) | -21.97  (-27.68, -14.8) | 0.05  (0.04, 0.05)^#4^ | 16.37  (6.4, 30.74) | 0.03  (0.03, 0.03^)#5^ | 7.88  (2.79, 15.9) | 0.21  (0.2, 0.23) | -12.50  (-16.67, -4.17) |
|  | Crude rate | 13.96  (12.93, 15.24) | -28.70  (-33.96, -22.17) | 4.89  (4.47, 5.49) | 6.30  (-2.83, 19.35) | 3.12  (2.98, 3.36) | -1.58  (-5.99, 5.99) | 21.97  (20.38, 24.09) | -19.67  (-25.48, -11.92) |
|  | Age-standardized rate | 10.75  (10.07, 11.55) | -17.88  (-23.07, -11.76) | 3.58  (3.23, 3.85) | 4.68  (-5.56, 12.57) | 2.45  (2.38, 2.54) | 2.51  (-0.42, 6.28) | 16.78  (15.68, 17.94) | -11.17  (-16.99, -5.03) |
| High-middle SDI | Absolute number | 0.07  (0.06, 0.08) | -69.92  (-75.17, -65.32) | 0.04  (0.03, 0.05) | -56.54  (-67.69, -42.78) | 0.02  (0.02, 0.03)^#6^ | -24.29  (-29.31, -4.76) | 0.13  (0.11, 0.16) | -62.86  (-68.57, -54.29) |
|  | Crude rate | 7.65  (6.32, 8.83) | -59.74  (-66.74, -53.53) | 3.92  (2.91, 5.16) | -41.75  (-56.76, -23.33) | 2.39  (2.23, 3.01) | 1.27  (-5.51, 27.54) | 13.96  (11.46, 16.99) | -50.28  (-59.19, -39.49) |
|  | Age-standardized rate | 6.93  (6.44, 7.66) | -43.61  (-47.6, -37.67) | 3  (2.67, 3.38) | -30.39  (-38.05, -21.58) | 2.45  (2.35, 2.55) | -17.51  (-20.88, -14.14) | 12.38  (11.46, 13.58) | -36.71  (-41.41, -30.57) |
| High SDI | Absolute number | 0.01  (0.01, 0.02)^#7^ | -39.50  (-48.1, -31.82) | 0.00  (0.00, 0.01)^#8^ | -50.85  (-58.53, -43.25) | 0.01  (0.01, 0.01)^#9^ | -18.07  (-20.52, -15.86) | 0.03  (0.03, 0.03)^#10^ | -40.00  (-40.00, -40.00^)#11^ |
|  | Crude rate | 2.7  (2.32, 3.04) | -33.66  (-43, -25.31) | 0.82  (0.69, 0.95) | -46.05  (-54.61, -37.5) | 2.02  (1.96, 2.07) | -10.22  (-12.89, -8) | 5.54  (4.97, 6.06) | -29.43  (-36.69, -22.8) |
|  | Age-standardized rate | 2.33  (2.14, 2.62) | -44.79  (-49.29, -37.91) | 0.88  (0.77, 1) | -52.69  (-58.6, -46.24) | 1.76  (1.67, 1.86) | -15.38  (-19.71, -10.58) | 4.97  (4.58, 5.49) | -39.09  (-43.87, -32.72) |

Notes: Absolute number are in millions. The denominator of crude incidence rate and age standardized incidence rate is per 100 000 people. Absolute numbers in millions, crude rates per 100 000 people, age-standardised rates per 100 000 people, and percentage change are presented to two decimal places. UI=uncertainty interval. DALY=dis ability adjusted life-year.

#1: Absolute number of subarachnoid haemorrhage dead cases among 15-59 years old population in low SDI countries in 2050 is 13459(12704, 14946);

#2: Absolute number of ischaemic haemorrhage dead cases among 15-59 years old population in low-middle SDI countries in 2050 is 73211(65759, 81527);

#3: Absolute number of subarachnoid haemorrhage dead cases among 15-59 years old population in low-middle SDI countries in 2050 is 41741(37088, 44674);

#4: Absolute number of ischaemic haemorrhage dead cases among 15-59 years old population in middle SDI countries in 2050 is 47276(43224, 53114);

#5: Absolute number of subarachnoid haemorrhage dead cases among 15-59 years old population in middle SDI countries in 2050 is 30216(28792, 32462);

#6: Absolute number of subarachnoid haemorrhage dead cases among 15-59 years old population in high-middle SDI countries in 2050 is 22048(20586, 27735);

#7: Absolute number of intracerebral haemorrhage dead cases among 15-59 years old population in high SDI countries in 2050 is 144766(12418, 16313);

#8: Absolute number of Ischaemic haemorrhage dead cases among 15-59 years old population in high SDI countries in 2050 is 4399(3712, 5079);

#9: Absolute number of subarachnoid haemorrhage dead cases among 15-59 years old population in high SDI countries in 2050 is 10815(10491, 11106);

#10: Absolute number of all stroke dead cases among 15-59 years old population in high SDI countries in 2050 is 29690(26620, 32497);

#11: The relative change of absolute number of all stroke dead cases among 15-59 years old population in high SDI countries in 2050 is -35.56(-42.22, -29.47).

**Table S11. Number of cases, crude rates and age-standardised rates per 100 000 population in 2050 and percentage change between 2021 and 2050 for stroke DALYs, by SDI quintile, for both sexes in people aged 15-59 years (95% uncertainty interval).**

|  | | **Intracerebral haemorrhage** | | **Ischaemic stroke** | | **Subarachnoid haemorrhage** | | **All stroke** | |
| --- | --- | --- | --- | --- | --- | --- | --- | --- | --- |
|  |  | **2050** | **Percentage change,**  **2021-2050** | **2050** | **Percentage change,**  **2021-2050** | **2050** | **Percentage change,**  **2021-2050** | **2050** | **Percentage change,**  **2021-2050** |
| Low SDI | Absolute number | 5.48  (4.81, 6.04) | 78.92  (57.13, 97.39) | 1.39  (0.98, 1.77) | 92.92  (36.01, 144.88) | 0.77  (0.7, 0.84) | 120.11  (101.5, 139.7) | 7.64  (6.49, 8.64) | 84.99  (57.14, 109.2) |
|  | Crude rate | 629.89  (553.18, 694.91) | -14.59  (-24.99, -5.77) | 160.08  (112.86, 203.2) | -7.91  (-35.07, 16.9) | 88.31  (80.85, 96.18) | 5.07  (-3.81, 14.43) | 878.28  (746.89, 994.29) | -11.76  (-24.96, -0.11) |
|  | Age-standardized rate | 710.44  (654.24, 764.06) | -21.39  (-27.61, -15.45) | 154.74  (111.44, 193.79) | -20.43  (-42.69, -0.34) | 101.26  (93.78, 108.02) | -3.55  (-10.68, 2.89) | 966.43  (859.46, 1065.87) | -19.68  (-28.57, -11.41) |
| Low-middle SDI | Absolute number | 7.96  (6.17, 9.75) | -15.63  (-34.62, 3.34) | 3.70  (2.46, 4.81) | 30.2  (-13.54, 69.23) | 1.56  (1.34, 1.78) | -16.97  (-28.67, -5.58) | 13.22  (9.97, 16.33) | -6.64  (-29.59, 15.32) |
|  | Crude rate | 384.7  (298.08, 471.2) | -35.38  (-49.93, -20.85) | 178.77  (118.71, 232.36) | -0.28  (-33.79, 29.61) | 75.63  (64.96, 86) | -36.41  (-45.38, -27.69) | 639.09  (481.75, 789.55) | -28.48  (-46.09, -11.64) |
|  | Age-standardized rate | 765.36  (702.05, 826.08) | -20.4  (-26.99, -14.09) | 163.74  (126.32, 203.44) | -29.6  (-45.69, -12.53) | 138.23  (131.33, 147.99) | -12.79  (-17.14, -6.63) | 1067.33  (959.7, 1177.52) | -21.09  (-29.05, -12.95) |
| Middle SDI | Absolute number | 6.02  (5.40, 6.56) | -17.87  (-26.3, -10.47) | 1.92  (1.44, 2.4) | -15.09  (-36.36, 5.82) | 1.45  (1.37, 1.53) | 7.68  (1.67, 13.69) | 9.39  (8.21, 10.49) | -14.17  (-24.95, -4.11) |
|  | Crude rate | 622.14  (558.33, 678.2) | -24.98  (-32.68, -18.23) | 198.74  (148.96, 247.68) | -22.44  (-41.86, -3.34) | 149.98  (141.61, 158.35) | -1.64  (-7.13, 3.85) | 970.85  (848.9, 1084.23) | -21.58  (-31.43, -12.43) |
|  | Age-standardized rate | 432.51  (391.99, 479.24) | -23.16  (-30.36, -14.86) | 120.33  (78.85, 157.47) | -38.4  (-59.63, -19.38) | 125.93  (118.77, 132.15) | 6.79  (0.72, 12.07) | 678.77  (589.6, 768.87) | -22.53  (-32.71, -12.24) |
| High-middle SDI | Absolute number | 2.89  (1.96, 3.73) | -70.60  (-80.04, -61.98) | 2.25  (1.55, 3.08) | -49.82  (-65.44, -31.24) | 1.24  (1.06, 1.41) | -11.78  (-24.73, 0.65) | 6.37  (4.56, 8.23) | -59.43  (-70.96, -47.58) |
|  | Crude rate | 312.89  (212.4, 404.56) | -60.61  (-73.26, -49.06) | 243.53  (167.71, 333.67) | -32.77  (-53.7, -7.89) | 134.13  (114.44, 153.04) | 18.19  (0.84, 34.85) | 690.55  (494.55, 891.27) | -45.62  (-61.06, -29.82) |
|  | Age-standardized rate | 401.73  (366.6, 443.81) | -23.04  (-29.77, -14.98) | 251.14  (202.13, 299.39) | -1.77  (-20.94, 17.1) | 129.42  (124.99, 133.85) | -9.53  (-12.62, -6.43) | 782.29  (693.72, 877.04) | -15.03  (-24.65, -4.74) |
| High SDI | Absolute number | 0.87  (0.72, 1.03) | -19.64  (-33.57, -4.88) | 0.61  (0.47, 0.8) | -23.27  (-41.02, -0.06) | 0.54  (0.5, 0.57) | -16.16  (-21.81, -11.83) | 2.02  (1.69, 2.4) | -20.16  (-33.2, -5.14) |
|  | Crude rate | 162.35  (134.21, 192.17) | -11.95  (-27.21, 4.22) | 114.56  (88.06, 149.21) | -15.93  (-35.38, 9.5) | 100.6  (93.82, 105.8) | -8.14  (-14.33, -3.39) | 377.51  (316.09, 447.18) | -12.24  (-26.52, 3.95) |
|  | Age-standardized rate | 155.47  (137.4, 174.24) | -17.14  (-26.77, -7.13) | 138.80  (109.44, 164.64) | -4.1  (-24.38, 13.76) | 104.11  (100.16, 108.29) | 2.11  (-1.77, 6.21) | 398.39  (347.01, 447.17) | -8.27  (-20.10, 2.96) |

Notes: Absolute number are in millions. The denominator of crude incidence rate and age standardized incidence rate is per 100 000 people. Absolute numbers in millions, crude rates per 100 000 people, age-standardised rates per 100 000 people, and percentage change are presented to two decimal places. UI=uncertainty interval. DALY=dis ability adjusted life-year.

**Table S12. Number of cases, crude rates and age-standardised rates per 100 000 population in 2050 and percentage change between 2021 and 2050 for stroke incidence, by SDI quintile,** **for both sexes in people aged ≥60 years (95% uncertainty interval).**

|  | | **Intracerebral haemorrhage** | | **Ischaemic stroke** | | **Subarachnoid haemorrhage** | | **All stroke** | |
| --- | --- | --- | --- | --- | --- | --- | --- | --- | --- |
|  |  | **2050** | **Percentage change,**  **2021-2050** | **2050** | **Percentage change,**  **2021-2050** | **2050** | **Percentage change,**  **2021-2050** | **2050** | **Percentage change,**  **2021-2050** |
| Low SDI | Absolute number | 0.19  (0.18, 0.20) | 149.13  (139.5, 159.18) | 0.39  (0.37, 0.4) | 200.89  (191.78, 207.76) | 0.02  (0.02, 0.02) ^#1^ | 166.39  (161.81, 171.2) | 0.59  (0.58, 0.61) | 180.95  (176.19, 190.48) |
|  | Crude rate | 182.94  (175.87, 190.32) | -13.21  (-16.56, -9.71) | 369.4  (358.22, 377.84) | 4.82  (1.65, 7.22) | 16.12  (15.84, 16.41) | -7.20  (-8.81, -5.53) | 568.46  (549.93, 584.57) | -2.08  (-5.27, 0.69) |
|  | Age-standardized rate | 207.90  (201.40, 213.01) | -12.18  (-14.92, -10.02) | 373.24  (365.96, 381.2) | 5.81  (3.74, 8.06) | 17.64  (17.35, 17.93) | -0.28  (-1.92, 1.36) | 598.79  (584.71, 612.14) | -1.38  (-3.7, 0.82) |
| Low-middle SDI | Absolute number | 0.61  (0.58, 0.66) | 54.63  (45.21, 66.02) | 1.85  (1.74, 1.94) | 224.93  (206.32, 240.26) | 0.09  (0.09, 0.09)^#2^ | 122.75  (112.94, 128.93) | 2.55  (2.41, 2.69) | 152.48  (138.61, 166.34) |
|  | Crude rate | 111.48  (104.69, 119.69) | -35.1  (-39.05, -30.32) | 336.1  (316.85, 351.95) | 36.38  (28.57, 42.81) | 16.62  (15.89, 17.09) | -6.52  (-10.63, -3.88) | 464.2  (437.43, 488.73) | 6.47  (0.33, 12.1) |
|  | Age-standardized rate | 169.48  (165.5, 173.32) | -23.03  (-24.84, -21.29) | 385.65  (380.93, 391.64) | 2.51  (1.26, 4.11) | 20.73  (20.34, 21.3) | -7.62  (-9.36, -5.08) | 575.86  (566.77, 586.26) | -6.94  (-8.41, -5.26) |
| Middle SDI | Absolute number | 0.39  (0.38, 0.42) | 71.74  (64.31, 81.04) | 1.18  (1.14, 1.21) | 117.63  (111.57, 124.14) | 0.08  (0.07, 0.08)^#3^ | 113.95  (109.12, 119.16) | 1.65  (1.60, 1.71) | 103.70  (97.53, 111.11) |
|  | Crude rate | 103.27  (98.8, 108.86) | -25.24  (-28.48, -21.2) | 308.74  (300.15, 317.98) | -5.27  (-7.9, -2.43) | 19.82  (19.37, 20.3) | -6.86  (-8.98, -4.61) | 431.83  (418.32, 447.14) | -11.02  (-13.81, -7.87) |
|  | Age-standardized rate | 103.66  (100.52, 107) | -15.90  (-18.45, -13.19) | 292.36  (286.82, 299.6) | -1.54  (-3.41, 0.9) | 18.35  (18, 18.68) | 3.50  (1.52, 5.36) | 414.36  (405.34, 425.28) | -5.38  (-7.44, -2.89) |
| High-middle SDI | Absolute number | 0.59  (0.52, 0.66) | -7.00  (-17.84, 3.44) | 2.05  (1.93, 2.2) | -1.51  (-7.37, 5.69) | 0.13  (0.12, 0.14)^#4^ | 69.75  (55.6, 83.42) | 2.78  (2.57, 3) | -0.71  (-8.21, 7.14) |
|  | Crude rate | 88.24  (77.95, 98.14) | -48.12  (-54.17, -42.3) | 305.02  (286.87, 327.33) | -45.06  (-48.33, -41.04) | 19.68  (18.04, 21.27) | -5.34  (-13.23, 2.31) | 412.95  (382.87, 446.75) | -44.65  (-48.68, -40.12) |
|  | Age-standardized rate | 83.09  (79.35, 87.01) | -22.56  (-26.04, -18.9) | 298.29  (292.61, 304.35) | -15.02  (-16.64, -13.29) | 16.93  (16.44, 17.47) | -6.88  (-9.57, -3.91) | 398.31  (388.41, 408.83) | -16.41  (-18.48, -14.2) |
| High SDI | Absolute number | 0.17  (0.17, 0.18) ^#5^ | 59.26  (53.36, 67.45) | 0.82  (0.75, 0.89) | 53.3  (41.2, 66.96) | 0.05  (0.05, 0.05)^#6^ | 21.61  (17.77, 24.06) | 1.04  (0.97, 1.13) | 50.72  (40.58, 63.77) |
|  | Crude rate | 50.53  (48.66, 53.13) | 17.16  (12.82, 23.19) | 238.16  (219.37, 259.39) | 12.78  (3.88, 22.83) | 15.72  (15.23, 16.04) | -10.53  (-13.32, -8.71) | 304.42  (283.26, 328.56) | 11.96  (4.18, 20.84) |
|  | Age-standardized rate | 56.03  (54.61, 57.57) | 0.59  (-1.96, 3.36) | 242.16  (236.46, 248.8) | -5.61  (-7.83, -3.02) | 14.92  (14.6, 15.14) | -6.05  (-8.06, -4.66) | 313.11  (305.66, 321.52) | -4.58  (-6.85, -2.02) |

Notes: Absolute number are in millions. The denominator of crude incidence rate and age standardized incidence rate is per 100 000 people. Absolute numbers in millions, crude rates per 100 000 people, age-standardised rates per 100 000 people, and percentage change are presented to two decimal places. UI=uncertainty interval. DALY=dis ability adjusted life-year.

#1: Absolute number of subarachnoid haemorrhage incident cases among 60+ years old population in low SDI countries in 2050 is 16868(16578, 17172);

#2: Absolute number of subarachnoid haemorrhage incident cases among 60+ years old population in low-middle SDI countries in 2050 is 91451(87424, 93988);

#3: Absolute number of intracerebral haemorrhage incident cases among 60+ years old population in middle SDI countries in 2050 is 75600(73893, 77443);

#4: Absolute number of intracerebral haemorrhage incident cases among 60+ years old population in high-middle SDI countries in 2050 is 132298(121266, 142952);

#5: Absolute number of intracerebral haemorrhage incident cases among 60+ years old population in high SDI countries in 2050 is 173262(166849, 182174);

#6: Absolute number of subarachnoid haemorrhage incident cases among 60+ years old population in high SDI countries in 2050 is 53903(52202, 54991).

**Table S13. Number of cases, crude rates and age-standardised rates per 100 000 population in 2050 and percentage change between 2021 and 2050 for stroke prevalence, by SDI quintile, for both sexes in people aged ≥60 years (95% uncertainty interval).**

|  | | **Intracerebral haemorrhage** | | **Ischaemic stroke** | | **Subarachnoid haemorrhage** | | **All stroke** | |
| --- | --- | --- | --- | --- | --- | --- | --- | --- | --- |
|  |  | **2050** | **Percentage change,**  **2021-2050** | **2050** | **Percentage change,**  **2021-2050** | **2050** | **Percentage change,**  **2021-2050** | **2050** | **Percentage change,**  **2021-2050** |
| Low SDI | Absolute number | 0.53  (0.52, 0.55) | 173.49  (165.61, 181.75) | 3.66  (3.57, 3.75) | 194.44  (187.27, 201.45) | 0.27  (0.26, 0.27) # | 172.71  (167.71, 179.2) | 5.35  (5.18, 5.51) | 211.05  (201.16, 220.35) |
|  | Crude rate | 507.14  (492.53, 522.45) | -4.72  (-7.47, -1.84) | 3497.56  (3412.38, 3580.77) | 2.58  (0.08, 5.02) | 253.8  (249.15, 259.84) | -4.99  (-6.73, -2.73) | 5110.1  (4953.91, 5264.42) | 8.45  (5.13, 11.72) |
|  | Age-standardized rate | 570.79  (556.73, 584.62) | -4.32  (-6.68, -2) | 3668.39  (3599.28, 3729.69) | 4.63  (2.66, 6.38) | 270.96  (266.39, 275.36) | -2.19  (-3.84, -0.6) | 5377.41  (5246.5, 5482.08) | 16.08  (13.25, 18.34) |
| Low-middle SDI | Absolute number | 2.65  (2.54, 2.76) | 88.07  (80.45, 96.24) | 14.93  (14.35, 15.55) | 193.06  (181.62, 205.3) | 1.22  (1.18, 1.27) | 162.79  (153.3, 172.78) | 22.67  (21.92, 23.45) | 183.38  (174, 193.12) |
|  | Crude rate | 481.14  (461.66, 502.06) | -21.06  (-24.26, -17.63) | 2713.66  (2607.68, 2826.97) | 23  (18.2, 28.14) | 222.00  (213.98, 230.43) | 10.3  (6.31, 14.49) | 4121.70  (3984.2, 4263.22) | 18.93  (14.96, 23.01) |
|  | Age-standardized rate | 536.72  (524.19, 548.93) | -13.99  (-16, -12.04) | 3596.24  (3551.78, 3640.14) | 3.32  (2.04, 4.58) | 296.31  (291.59, 300.23) | -8.1  (-9.57, -6.89) | 5303.32  (5241.52, 5373.85) | 21.88  (20.46, 23.51) |
| Middle SDI | Absolute number | 1.65  (1.59, 1.71) | 121.69  (114.35, 129.67) | 11.04  (10.86, 11.28) | 116.33  (112.86, 121) | 1.14  (1.11, 1.18) | 131.08  (124.86, 138.86) | 17.03  (16.74, 17.36) | 122.91  (119.11, 127.23) |
|  | Crude rate | 431.5  (417.22, 447.04) | -3.5  (-6.7, -0.03) | 2894.33  (2848.03, 2956.87) | -5.84  (-7.34, -3.8) | 299.44  (291.38, 309.53) | 0.59  (-2.12, 3.98) | 4465.36  (4388.96, 4550.42) | -2.91  (-4.57, -1.06) |
|  | Age-standardized rate | 404.94  (395.28, 415.01) | 2.93  (0.48, 5.49) | 2898.84  (2842, 2946.51) | 3.94  (1.9, 5.65) | 301.69  (297.36, 306.89) | 11.05  (9.46, 12.96) | 4537.17  (4476.43, 4611.24) | 13.75  (12.23, 15.61) |
| High-middle SDI | Absolute number | 3.51  (3.37, 3.67) | 81.84  (74.48, 90.23) | 21.14  (20.25, 22.01) | 35.84  (30.07, 41.43) | 1.81  (1.63, 2.12) | 151.06  (126.51, 195.1) | 37.59  (36.07, 39.05) | 61.89  (55.34, 68.17) |
|  | Crude rate | 522.64  (501.47, 546.74) | 1.44  (-2.67, 6.11) | 3145.76  (3012.1, 3275.15) | -24.22  (-27.44, -21.11) | 268.76  (242.49, 315.91) | 40.04  (26.36, 64.61) | 5592.94  (5366.78, 5810.25) | -9.69  (-13.34, -6.18) |
|  | Age-standardized rate | 376.64  (369.01, 384.53) | -5.74  (-7.65, -3.77) | 3165.72  (3106.69, 3212.47) | -3.46  (-5.26, -2.03) | 263.64  (259.76, 267.53) | 6.33  (4.76, 7.9) | 5097.94  (5030.16, 5164.87) | 22.78  (21.15, 24.39) |
| High SDI | Absolute number | 1.57  (1.53, 1.62) | 47.78  (43.68, 52.49) | 11.63  (10.88, 12.44) | 58.60  (48.33, 69.7) | 1.14  (1.11, 1.17) | 34.32  (30.62, 37.65) | 20.39  (19.49, 21.36) | 47.75  (41.23, 54.78) |
|  | Crude rate | 458.67  (445.96, 473.29) | 8.72  (5.7, 12.18) | 3391.57  (3171.86, 3629) | 16.68  (9.12, 24.85) | 331.79  (322.64, 340.02) | -1.18  (-3.91, 1.27) | 5948.39  (5683.87, 6231.28) | 8.71  (3.88, 13.88) |
|  | Age-standardized rate | 387.52  (380.9, 396.8) | 7.06  (5.24, 9.63) | 2919.85  (2861.87, 2982.03) | 1.04  (-0.97, 3.19) | 301.52  (297.03, 305.96) | 9.74  (8.1, 11.35) | 5167.53  (5073.87, 5256.14) | 25.37  (23.1, 27.52) |

Notes: Absolute number are in millions. The denominator of crude incidence rate and age standardized incidence rate is per 100 000 people. Absolute numbers in millions, crude rates per 100 000 people, age-standardised rates per 100 000 people, and percentage change are presented to two decimal places. UI=uncertainty interval. DALY=dis ability adjusted life-year.

#: Absolute number of subarachnoid haemorrhage prevalent cases among 60+ years old population in low SDI countries in 2050 is 265570(260704, 271895).

**Table S14. Number of cases, crude rates and age-standardised rates per 100 000 population in 2050 and percentage change between 2021 and 2050 for stroke deaths, by SDI quintile, for both sexes in people aged ≥60 years (95% uncertainty interval).**

|  | | **Intracerebral haemorrhage** | | **Ischaemic stroke** | | **Subarachnoid haemorrhage** | | **All stroke** | |
| --- | --- | --- | --- | --- | --- | --- | --- | --- | --- |
|  |  | **2050** | **Percentage change,**  **2021-2050** | **2050** | **Percentage change,**  **2021-2050** | **2050** | **Percentage change,**  **2021-2050** | **2050** | **Percentage change,**  **2021-2050** |
| Low SDI | Absolute number | 0.26  (0.24, 0.27) | 125.12  (111.82, 137.12) | 0.19  (0.19, 0.20)^#1^ | 168.65  (161.01, 178.01) | 0.01  (0.01, 0.01)^#2^ | 158.54  (149.49, 165.32) | 0.46  (0.44, 0.48) | 142.11  (131.58, 152.63) |
|  | Crude rate | 245.74  (231.22, 258.83) | -21.57  (-26.21, -17.39) | 182.8  (177.6, 189.17) | -6.41  (-9.07, -3.14) | 11.27  (10.88, 11.57) | -9.91  (-13.03, -7.51) | 439.81  (419.7, 459.56) | -15.61  (-19.47, -11.82) |
|  | Age-standardized rate | 306.19  (291.64, 322.38) | -14.56  (-18.62, -10.04) | 217.08  (211.03, 224.84) | -1.04  (-3.8, 2.5) | 13.76  (13.35, 14.19) | -2.2  (-5.12, 0.85) | 537.03  (516.03, 561.41) | -9.25  (-12.8, -5.14) |
| Low-middle SDI | Absolute number | 0.62  (0.54, 0.72) | 34.82  (18.41, 56.85) | 0.74  (0.69, 0.79) | 97.38  (84.49, 113.09) | 0.07  (0.07, 0.08)^#3^ | 101.87  (83.12, 116.69) | 1.42  (1.29, 1.59) | 63.22  (48.28, 82.76) |
|  | Crude rate | 112.03  (98.4, 130.34) | -43.42  (-50.3, -34.17) | 133.72  (124.99, 144.37) | -17.16  (-22.56, -10.56) | 13.15  (11.93, 14.12) | -15.27  (-23.13, -9.02) | 258.9  (235.32, 288.82) | -30.95  (-37.23, -22.96) |
|  | Age-standardized rate | 209.77  (199.79, 221.54) | -29.06  (-32.43, -25.07) | 178.51  (167.31, 186.74) | -9.2  (-14.9, -5.02) | 15.06  (14.48, 15.84) | -11.31  (-14.72, -6.71) | 403.33  (381.58, 424.12) | -20.80  (-25.07, -16.72) |
| Middle SDI | Absolute number | 0.37  (0.34, 0.41) | 20.6  (11.35, 32.87) | 0.42  (0.39, 0.46) | 66.89  (52.18, 80.64) | 0.05  (0.05, 0.05)^#4^ | 98.95  (88.76, 108.37) | 0.84  (0.77, 0.92) | 44.83  (32.76, 58.62) |
|  | Crude rate | 96.37  (88.98, 106.18) | -47.51  (-51.53, -42.16) | 110.99  (101.21, 120.14) | -27.36  (-33.76, -21.37) | 13.13  (12.46, 13.75) | -13.39  (-17.81, -9.3) | 220.49  (202.65, 240.07) | -37.28  (-42.35, -31.71) |
|  | Age-standardized rate | 96.41  (89.77, 103.43) | -39.71  (-43.86, -35.32) | 101.15  (96.12, 106.15) | -28.07  (-31.65, -24.51) | 11.13  (10.73, 11.62) | -6.55  (-9.91, -2.43) | 208.7  (196.62, 221.2) | -33.2  (-37.07, -29.2) |
| High-middle SDI | Absolute number | 0.41  (0.36, 0.5) | -44.97  (-52.14, -33.45) | 0.56  (0.47, 0.67) | -24.89  (-36.53, -9.82) | 0.09  (0.08, 0.1) | 56.57  (34.59, 72.8) | 1.06  (0.91, 1.27) | -31.61  (-41.29, -18.06) |
|  | Crude rate | 61.28  (53.3, 74.1) | -69.3  (-73.3, -62.88) | 82.97  (70.11, 99.62) | -58.1  (-64.6, -49.69) | 14.02  (12.05, 15.47) | -12.65  (-24.92, -3.61) | 158.27  (135.46, 189.2) | -61.74  (-67.26, -54.27) |
|  | Age-standardized rate | 57.95  (55.14, 62.14) | -50.96  (-53.33, -47.41) | 80.42  (73.93, 87.67) | -49.06  (-53.17, -44.47) | 9.75  (9.23, 10.15) | -21.12  (-25.32, -17.88) | 148.12  (138.29, 159.96) | -48.64  (-52.05, -44.53) |
| High SDI | Absolute number | 0.10  (0.09, 0.10)^#5^ | 16.39  (9.05, 24.57) | 0.11  (0.10, 0.12) | 6.87  (0.96, 21.21) | 0.03  (0.03, 0.03)^#6^ | 29.12  (24.72, 35.82) | 0.23  (0.22, 0.25) | 15.00  (10.00, 25.00) |
|  | Crude rate | 27.72  (25.97, 29.66) | -14.37  (-19.77, -8.37) | 31.56  (29.81, 35.8) | -21.38  (-25.73, -10.81) | 8.29  (8.01, 8.72) | -5.04  (-8.25, -0.11) | 67.57  (63.79, 74.18) | -16.83  (-21.48, -8.69) |
|  | Age-standardized rate | 28.26  (27.04, 30.21) | -35.33  (-38.12, -30.87) | 36.37  (34.02, 39.12) | -47.04  (-50.46, -43.03) | 7.69  (7.4, 7.98) | -12.71  (-16, -9.42) | 72.32  (68.46, 77.3) | -40.32  (-43.51, -36.21) |

Notes: Absolute number are in millions. The denominator of crude incidence rate and age standardized incidence rate is per 100 000 people. Absolute numbers in millions, crude rates per 100 000 people, age-standardised rates per 100 000 people, and percentage change are presented to two decimal places. UI=uncertainty interval. DALY=dis ability adjusted life-year.

#1: Absolute number of Ischaemic haemorrhage dead cases among 60+ years old population in low SDI countries in 2050 is 191279(185841, 197942);

#2: Absolute number of subarachnoid haemorrhage dead cases among 60+ years old population in low SDI countries in 2050 is 11795(11382, 12104);

#3: Absolute number of intracerebral haemorrhage dead cases among 60+ years old population in low-middle SDI countries in 2050 is 72340 (65621, 77654);

#4: Absolute number of intracerebral haemorrhage dead cases among 60+ years old population in middle SDI countries in 2050 is 50078 (47514, 52450);

#5: Absolute number of intracerebral haemorrhage dead cases among 60+ years old population in high-middle SDI countries in 2050 is 95027 (89033, 101705);

#6: Absolute number of subarachnoid haemorrhage dead cases among 60+ years old population in high SDI countries in 2050 is 28433 (27463, 29908).

**Table S15. Number of cases, crude rates and age-standardised rates per 100 000 population in 2050 and percentage change between 2021 and 2050 for stroke DALYs, by SDI quintile, for both sexes in people aged ≥60 years (95% uncertainty interval).**

|  | | **Intracerebral haemorrhage** | | **Ischaemic stroke** | | **Subarachnoid haemorrhage** | | **All stroke** | |
| --- | --- | --- | --- | --- | --- | --- | --- | --- | --- |
|  |  | **2050** | **Percentage change,**  **2021-2050** | **2050** | **Percentage change,**  **2021-2050** | **2050** | **Percentage change,**  **2021-2050** | **2050** | **Percentage change,**  **2021-2050** |
| Low SDI | Absolute number | 5.31  (5.05, 5.6) | 103.84  (93.97, 115.13) | 4.84  (4.63, 5) | 192.65  (180.15, 202.26) | 0.32  (0.31, 0.34) | 172.36  (161.9, 184.94) | 10.47  (9.99, 10.93) | 139.59  (128.6, 150.11) |
|  | Crude rate | 5072.27  (4826.47, 5353.18) | -28.98  (-32.43, -25.05) | 4622.33  (4424.97, 4774.07) | 1.95  (-2.4, 5.3) | 307.35  (295.54, 321.54) | -5.12  (-8.76, -0.73) | 10001.95  (9546.98, 10448.78) | -16.65  (-20.44, -12.93) |
|  | Age-standardized rate | 6041.78  (5792.72, 6229.6) | -26.40  (-29.43, -24.11) | 4925.37  (4782.33, 5050.12) | -3.22  (-6.03, -0.77) | 352.73  (340.57, 362.93) | -2.90  (-6.25, -0.09) | 11319.89  (10915.62, 11642.66) | -17.14  (-20.10, -14.78) |
| Low-middle SDI | Absolute number | 15.78  (14.18, 17.16) | 50.65  (35.44, 63.93) | 19.35  (18.14, 20.53) | 127.38  (113.2, 141.29) | 1.61  (1.46, 1.78) | 80.78  (64.57, 100) | 36.73  (33.79, 39.47) | 84.85  (70.06, 98.64) |
|  | Crude rate | 2867.61  (2578.04, 3120.24) | -36.77  (-43.15, -31.2) | 3517.31  (3298, 3732.61) | -4.57  (-10.52, 1.28) | 291.83  (265.67, 322.86) | -24.12  (-30.92, -16.06) | 6676.75  (6141.71, 7175.71) | -22.41  (-28.63, -16.61) |
|  | Age-standardized rate | 4781.55  (4606.73, 4929.58) | -29.56  (-32.14, -27.38) | 4539.24  (4453.14, 4614.77) | -2.39  (-4.24, -0.76) | 388.68  (377.96, 400.31) | -10.69  (-13.15, -8.02) | 9709.47  (9437.83, 9944.66) | -18.23  (-20.52, -16.25) |
| Middle SDI | Absolute number | 9.65  (9.11, 10.13) | 38.17  (30.44, 45.16) | 12.71  (11.91, 13.67) | 110.27  (96.96, 126.12) | 1.57  (1.5, 1.67) | 145.80  (134.84, 162.07) | 23.93  (22.51, 25.48) | 75.05  (64.67, 86.39) |
|  | Crude rate | 2529.04  (2387.51, 2657.01) | -39.86  (-43.22, -36.81) | 3332.85  (3121.82, 3584.08) | -8.47  (-14.27, -1.57) | 411.19  (392.85, 438.41) | 6.99  (2.22, 14.08) | 6273.09  (5902.18, 6679.5) | -23.78  (-28.29, -18.85) |
|  | Age-standardized rate | 2417.11  (2330.8, 2534.47) | -33.65  (-36.02, -30.43) | 2877.92  (2738.12, 3004.33) | -13.67  (-17.86, -9.88) | 335.76  (325.65, 345.83) | 9.96  (6.64, 13.25) | 5630.79  (5394.57, 5884.63) | -22.67  (-25.92, -19.19) |
| High-middle SDI | Absolute number | 12.44  (10.35, 14.93) | -22.89  (-35.84, -7.44) | 16.44  (14.27, 18.44) | -8.23  (-20.3, 2.94) | 2.36  (2.14, 2.62) | 65.86  (49.93, 83.7) | 31.24  (26.76, 35.99) | -11.93  (-24.56, 1.47) |
|  | Crude rate | 1850.97  (1540.16, 2221.81) | -56.99  (-64.21, -48.37) | 2445.34  (2123.59, 2743.07) | -48.81  (-55.54, -42.58) | 351.42  (317.67, 389.22) | -7.48  (-16.37, 2.47) | 4647.73  (3981.41, 5354.09) | -50.87  (-57.91, -43.4) |
|  | Age-standardized rate | 1746.62  (1670.89, 1833.2) | -33.91  (-36.77, -30.63) | 2775.45  (2654.61, 2889.07) | -25.57  (-28.81, -22.53) | 296.84  (290.11, 304.58) | -5.12  (-7.27, -2.65) | 4818.91  (4615.61, 5026.85) | -27.91  (-30.95, -24.8) |
| High SDI | Absolute number | 2.73  (2.49, 2.97) | 47.25  (34.18, 60.27) | 4.78  (4.09, 5.98) | 57.77  (34.9, 97.37) | 1.01  (0.95, 1.1) | 67.92  (57.53, 82.77) | 8.52  (7.52, 10.05) | 55.47  (37.23, 83.39) |
|  | Crude rate | 796.59  (725.91, 867.07) | 8.32  (-1.29, 17.91) | 1393.6  (1191.61, 1743.37) | 16.07  (-0.76, 45.2) | 293.98  (275.78, 319.97) | 23.54  (15.89, 34.46) | 2484.17  (2193.29, 2930.41) | 14.26  (0.88, 34.79) |
|  | Age-standardized rate | 817.86  (777.72, 864.58) | -16.66  (-20.75, -11.9) | 1507.5  (1440.26, 1578.48) | -15.77  (-19.53, -11.81) | 254.79  (247.74, 261.5) | 10.23  (7.18, 13.13) | 2580.15  (2465.72, 2704.56) | -14.06  (-17.87, -9.92) |

Notes: Absolute number are in millions. The denominator of crude incidence rate and age standardized incidence rate is per 100 000 people. Absolute numbers in millions, crude rates per 100 000 people, age-standardised rates per 100 000 people, and percentage change are presented to two decimal places. UI=uncertainty interval. DALY=dis ability adjusted life-year.
